# Enantioselective Synthesis of *vic*-Aminoalcohol Derivatives by Nickel-Catalyzed Reductive Coupling of Aldehydes with Protected Amino-pentadienoates

**DOI:** 10.1021/jacs.4c12002

**Published:** 2024-11-22

**Authors:** Thilo Bender, Alois Fürstner

**Affiliations:** Max-Planck-Institut für Kohlenforschung, 45470 Mülheim/Ruhr, Germany

## Abstract

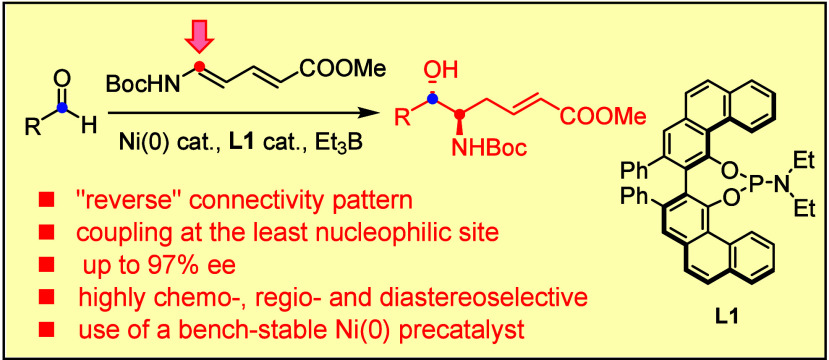

A VAPOL-derived phosphoramidite ligand is uniquely effective at reverting the regiochemical course of nickel-catalyzed reactions of aldehydes with carbamate-protected 5-amino-2,4-pentadienoates as “push/pull” dienes; the ensuing carbonyl α-amino-homoallylation reaction affords *anti*-configured *vic*-aminoalcohol derivatives in good yields with high optical purity. The reductive coupling is conveniently performed with a bench-stable Ni(0) precatalyst and Et_3_B as the promoter.

Our group has demonstrated that the VAPOL-derived phosphoramidite ligand **L1** is uniquely able to revert the course of nickel-catalyzed reductive coupling reactions of functionalized dienes with aldehydes and imines (Scheme 1).^1−7^ While dienyl (silyl)ethers such as **1** had long been known to react at the terminus to give adducts of type **2**,^8−11^ the addition of **L1** redirects the formation of the C–C bond to the least nucleophilic and sterically most hindered C atom carrying the oxygen substituent such that pre-differentiated 1,2-diols **3** are obtained.^1^ Electron-deficient dienes such as sorbate esters **5** also follow an “inverted” path: rather than reacting at the π-bond adjacent to the ester to give aldol-type products **6**, as documented in the literature,^10^ the combination Ni(0)/**L1** engages the distal olefin and furnishes deoxypropionates **7** terminated by an enoate moiety.^2^ Importantly, the products of types **3** and **7** were obtained with impeccable diastereoselectivity and high optical purity. This fact is all the more noteworthy if one considers that asymmetric variants of the traditional nickel-catalyzed diene/carbonyl coupling reactions had long been elusive or limited to special cases,^12−15^ and the challenge that they pose has been explicitly mentioned;^16^ only very recently have relevant examples of broader scope been disclosed.^17−19^

**Scheme 1:**
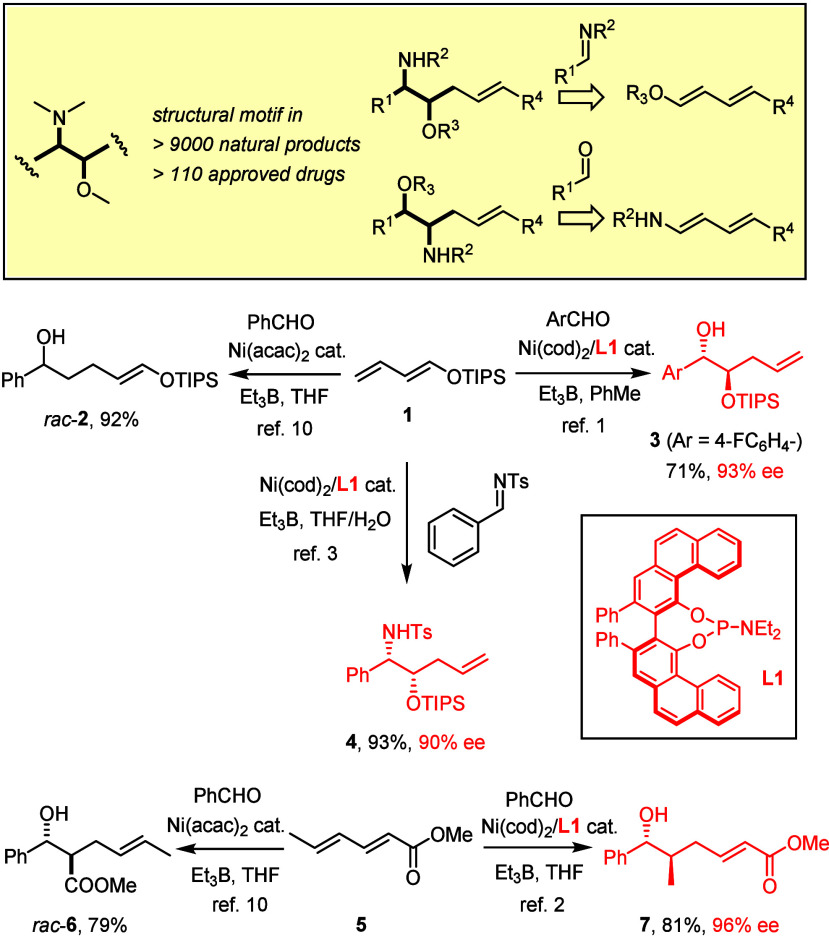
Conceived Formation of *vic*-Aminoalcohols and Summary of Relevant Prior Work

Although the exact reasons why the use of **L1** entails such massive consequences remain unknown at this stage of development, further investigations into this promising new reactivity mode are warranted. Specifically, an extension to the catalytic asymmetric formation of *vic*-aminoalcohols seems particularly desirable,^20^ given the prevalence of this motif in natural products, approved drugs, and drug candidates.^21^ Such compounds might be obtained by two regio-complementary routes that engage either an imine or a substituted amino-diene into an “inverted” coupling process (Scheme 1, top). If successful, this approach nicely complements the arsenal of catalytic asymmetric coupling reactions using metals other than nickel for the formation of 1,2-aminoalcohols by various mechanistic pathways.^22^

In a first foray, we have previously shown that Ni(0)/**L1** in combination with Et_3_B as the promoter does indeed effect the coupling of dienyl silyl ethers **1** with *N*-tosylimines to furnish *syn*-configured 1,2-aminoalcohol derivatives **4**, provided water is added to the reaction mixture (Scheme 1).^3^ The need for a rather stable *N*-tosyl group, however, is deemed a certain disadvantage, and access to the *anti*-series has not been gained.

Therefore, we explored the second approach in which a protected amino group is placed on the diene partner to be coupled with an aldehyde. Yet, the simple extrapolation from the 1,3-dienyl silyl ether **1** to 1,3-diene-carbamate **8** was unrewarding due to low reactivity, insufficient regiocontrol, and poor asymmetric induction (Scheme 2).^23^ During our previous study on sorbate esters, however, we had observed the favorable effects that electron-withdrawing substituents can entail.^24^ Therefore, we resorted to “push–pull” dienes *E,E*-**13**; although rarely used before, they are easy to make on a gram scale in differently protected format and can be stored in a freezer under Ar for extended periods of time.^25^ In the absence of any ligand, the nickel-catalyzed coupling of **13a** with benzaldehyde occurs, as expected, adjacent to the ester, to give the aldol derivative *rac*-**14** exclusively; the reaction can be performed with the air-stable Ni(0) stilbene complex **16** (10 mol %)^26^ as precatalyst and Et_3_B as promoter in THF. If the mixture is complemented with the phosphoramidite ligand **L1** (10 mol %), the course is reprogrammed and the *anti*-configured *vic*-aminoalcohol derivative **15a** formed virtually as the only isomer (rr >20:1, dr >20:1) in good yield with 91% ee; upon single recrystallization, optically pure material (99% ee) was attained. The relative and absolute configuration of **15a** was determined by single-crystal X-ray diffraction analysis (Figure 1). The nature of the carbamate plays a minor role, since **13b**–**d** reacted similarly well (Scheme 2);^27^ this provides synthetic flexibility as the various protecting groups can be cleaved under very different conditions. *N*-Alkylated aminodienes such as **17a**,**b** are equally suitable, whereas the phthalimide derivative **19** reacted poorly; the fact that products **18a** (R = Me) and **18b** (R = Bn) were both obtained in good yield and similar optical purity suggests that there is ample scope (Scheme 3).^28^ This notion is further corroborated by the excellent ee’s observed with aminodienes **17c**,**d** terminated by amide rather than ester groups; the compatibility of the highly versatile Weinreb amide function with its reducible N–O bond with the [Ni(0)] catalyst is particularly noteworthy.^29^

**Scheme 2:**
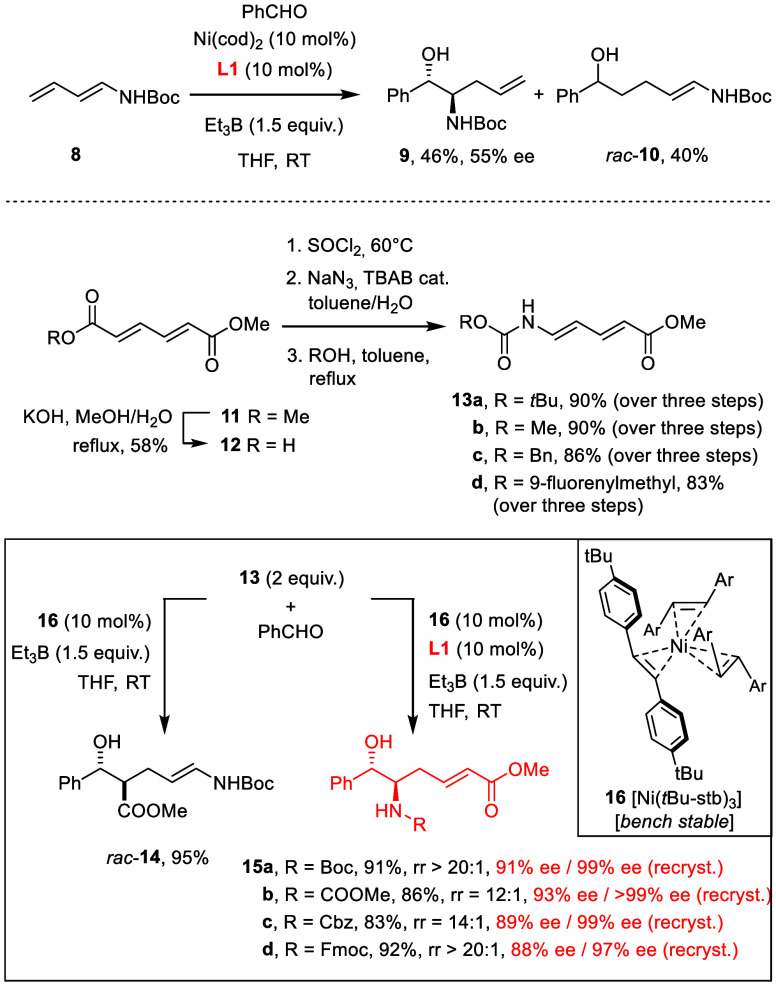
Nickel-Catalyzed Reductive Formation of *vic*-Aminoalcohol Derivatives

**Figure 1:**
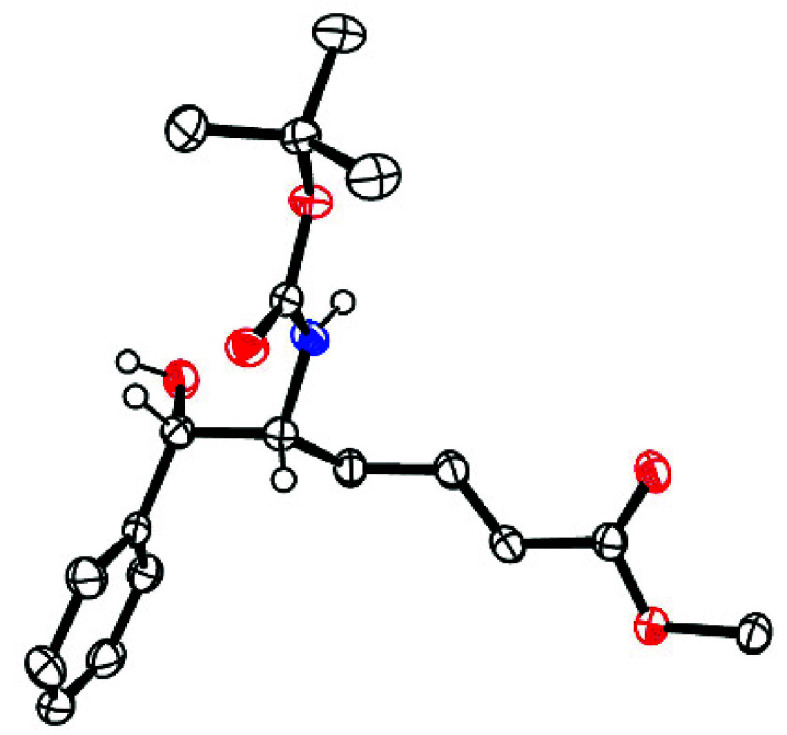
Structure of **15a** in the solid state.

**Scheme 3:**
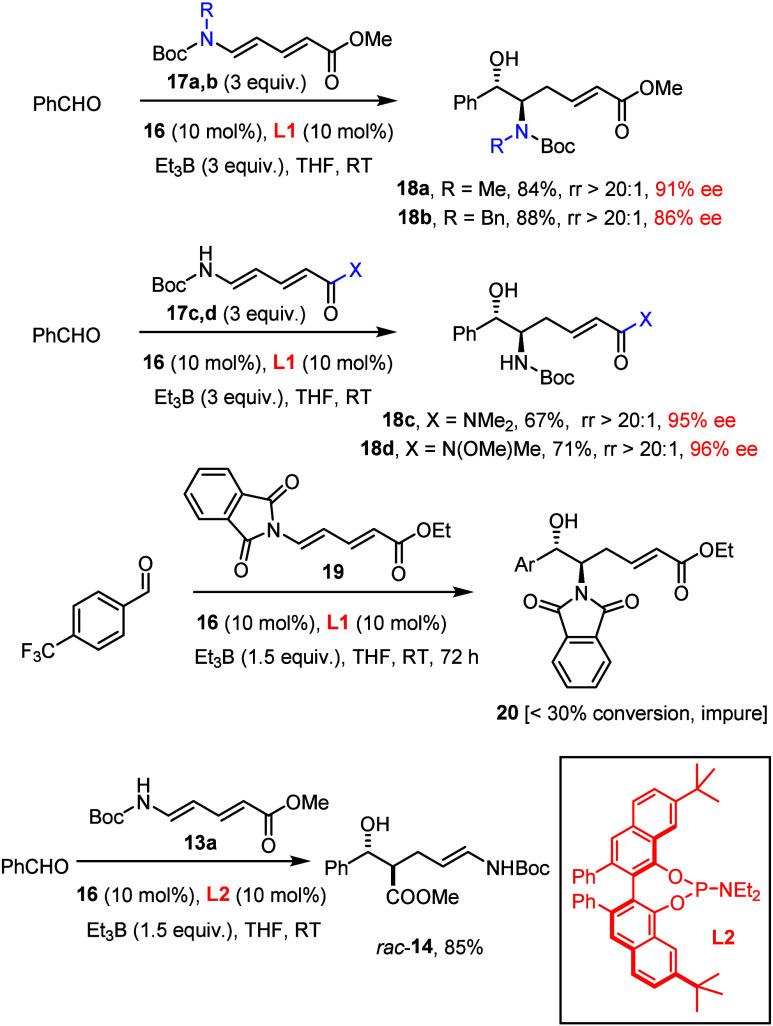
Control Experiments

Since the literature knows of various cases in which 7,7′-disubstituted VANOL ligands serve as favorable substitutes for VAPOL-derived catalyst systems,^7,30,31^ phosphoramidite **L2** was prepared and tested. Yet, this particular ligand is completely inadequate in the present context (Scheme 3); only the racemic background reaction seems to be operative. Since **L1** and **L2** are similarly vaulted biaryl derivatives, the disparity is striking and currently unexplained; **L1** thus remains, for the time being, the only ligand able to “invert” the course of nickel-catalyzed reductive coupling reactions, while rendering them highly enantioselective.^32^

Some additional observations made during a careful optimization exercise deserve mentioning (for details, see SI). Specifically, the reaction leading to *rac*-**14** is considerably faster than the asymmetric reaction controlled by **L1** affording product **15a**. Therefore, it is essential to preserve the integrity of the catalyst throughout the course of the coupling process; even small amounts of unligated Ni(0) or colloidal nickel^33^ will afford substantial amounts of the undesired regioisomer and/or might cause racemic background reactions. It proved beneficial to use the diene in excess (≥2 equiv), which drives the conversion and, at the same time, seems to stabilize the nickel complex in the catalyst resting state. Under these conditions, good reproducibility was ensured and the formation of the undesired regioisomer **14** largely suppressed; if desirable, the excess diene can be recovered during workup (see below).

Only Et_3_B was found suitable as promoter, whereas Et_2_Zn gave no more than traces of product even though this reagent has a good track record in ordinary nickel-catalyzed reductive coupling reactions.^8−11,34^ This striking divergence had already been observed in the inverse coupling reactions shown in Scheme 1 but has no good explanation. The same is true for the role of water (1–3 equiv), which was instrumental for the nickel-catalyzed coupling of dienyl silyl ethers **1** with *N*-tosylimines to give *syn*-configured aminoalcohol derivatives **4** (Scheme 1)^3,35^ but proved detrimental in the present case.^36^

An assortment of (hetero)aromatic aldehydes were reacted under the optimized conditions with diene **13a** in THF as the solvent (Chart 1). All reactions were performed with the bench-stable Ni(0) complex **16** as precatalyst,^26^ after a control experiment had shown that the much more sensitive Ni(cod)_2_ brings no advantage.^37^ In line with our expectations, aldehydes of greatly different electronic character and steric demand all reacted well, affording the corresponding *anti*-configured *vic*-aminoalcohol derivatives (dr >20:1) with generally good yields, high rr’s, and good to excellent ee’s. Most compounds are nicely crystalline materials, and a single recrystallization afforded optically pure product (≥99% ee) in almost all cases. The compatibility with numerous functional groups is noteworthy; specifically, the aryl chloride in **25** does not get activated by the Ni(0) catalyst. However, 4-bromobenzaldehyde was not suitable, although aryl bromides had passed uncompromised in “inverse” coupling reactions with the sorbate esters **5**;^2^ the difference is likely kinetic in origin, as sorbates react more quickly than the push–pull diene **13a** and hence give oxidative addition of Ni(0) into a C–Br bond less of a chance. The *ortho*-methyl substituent in **27** constitutes no serious steric impediment for the coupling reaction, although the rr was lower than that in the other cases. *p*-Trifluoromethylbenzaldehyde had been one of the more challenging substrates in the nickel-catalyzed reductive diol synthesis with **1**(1) but was fully compliant in the present setting. Likewise, the compatibility of the −B(pin) substituent is deemed an asset, as this group provides ample opportunity for downstream functionalization. *N*-Boc-pyrrole-2-carbaldehyde and furan-2-carbaldehyde also gave excellent results. A still appreciable outcome was observed for the corresponding thiophene derivative, despite the thiophilicity of low-valent nickel. 4-Cyanobenzaldehyde, however, resulted in incomplete conversion and an ee of only 68%. We presume that the sulfur-containing heterocycle and the −CN group, respectively, compete with **L1** for the nickel center and hence afford achiral systems resulting in racemic background reactions. Tight binding renders pyridine-4-carbaldehyde unsuitable; moreover, cinnamaldehyde, myrtenal, and pivaldehyde essentially failed to react.

**Chart 1:**
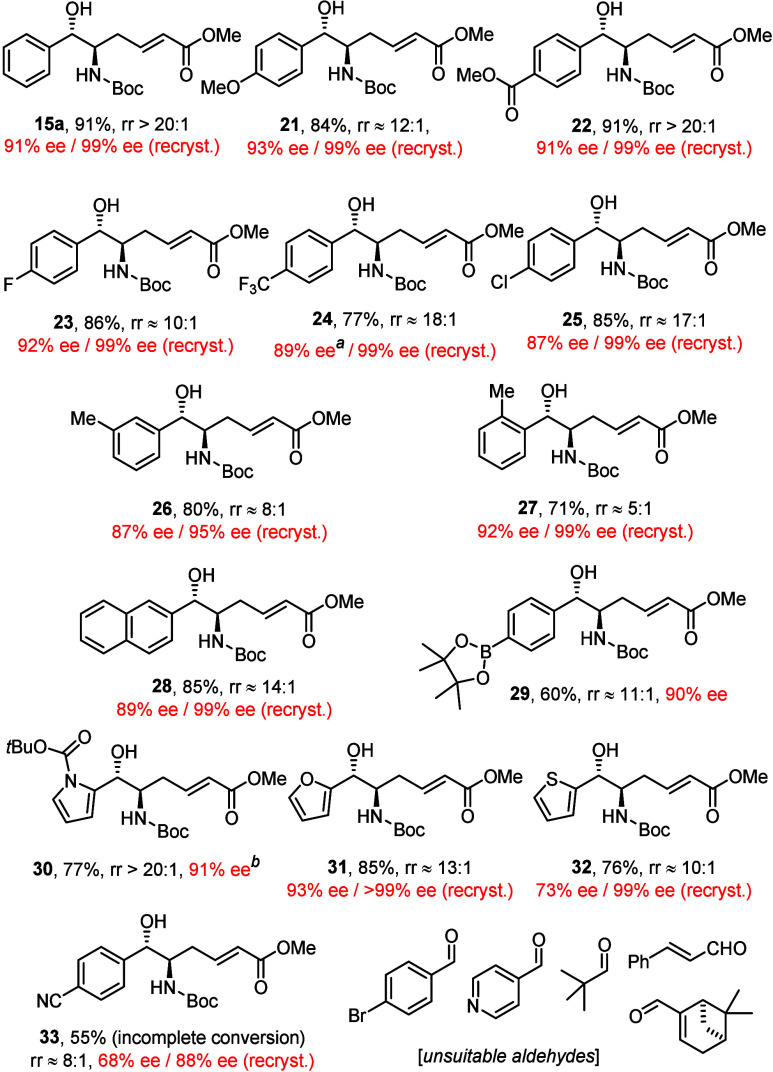
Reductive Coupling of Aromatic Aldehydes under the Optimized Conditions Shown in Scheme 2 (Unless Stated Otherwise); the rr’s (^1^H NMR) Refer to the Crude Material, the Yields to Analytically Pure Isolated Material

a At −10°C. b Using 3 equiv of **13a**.

For aliphatic aldehydes, the regiocontrol exerted by the phosphoramidite ligand **L1** is less stringent, and the isolated yields of product were invariably poor under the standard conditions (≤35%). Gratifyingly, replacement of THF by toluene had a positive effect on conversion, regioselectivity, and ee, which was reinforced when a larger excess of Et_3_B (also as a solution in toluene) was used. Since toluene is not optimal for aromatic aldehydes, this pronounced solvent effect is striking. Even though mixtures of both regioisomers were still formed, they proved separable by flash chromatography; the targeted *anti*-configured *vic*-aminoalcohol derivatives were obtained in respectable yields with excellent ee’s (Scheme 4); their configuration was assigned in analogy to the aromatic series. The lower level of regiocontrol suggests that aliphatic substrates lack some favorable secondary interactions with the extended π-system of **L1** when ligated to the nickel center in the regio-determining transition state, from which the aromatic aldehydes seem to benefit. In an attempt to improve the outcome, it was tested whether the placement of aromatic substituents in either reaction partner is helpful. However, neither use of the corresponding benzyl ester (cf. **43b**) nor arene-containing protecting groups (−OTBDPS, −OPMP) nor the use of aminodiene **15c** with a benzyloxy carbamate group led to any significant improvement. A more rational approach to addressing the issue must await a better understanding of the binding poses within the loaded catalyst.

**Scheme 4:**
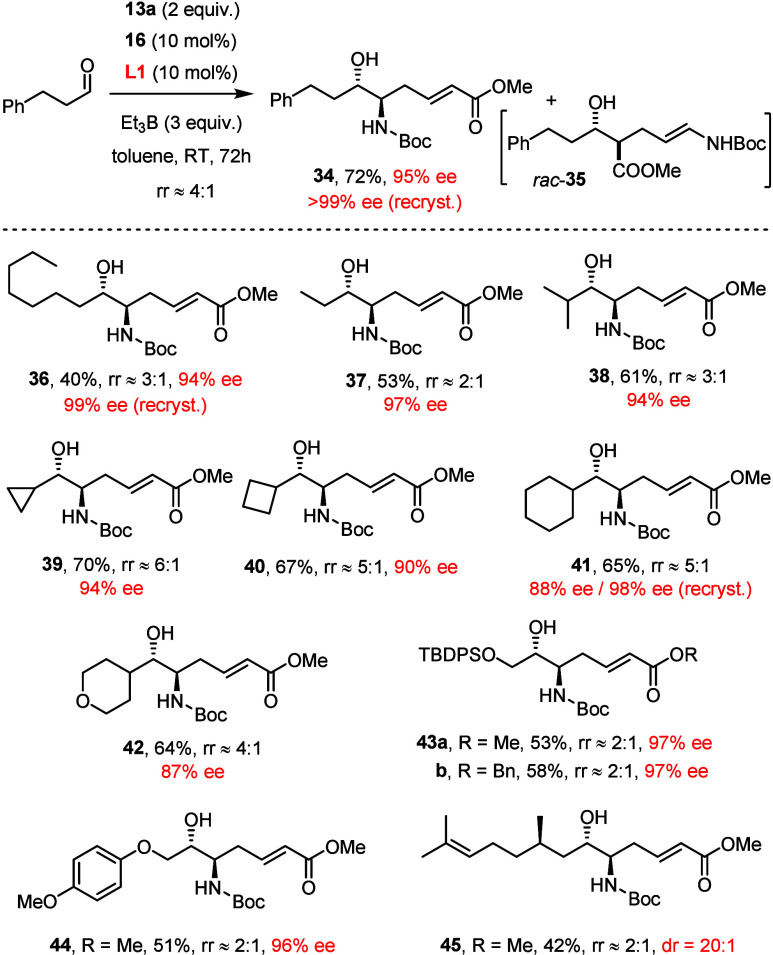
Nickel-Catalyzed Reductive Coupling of Aliphatic Aldehydes The rr’s (^1^H NMR) refer to the crude material; the yields to the shown products after separation of the regioisomers.

The reaction was well-scaled (Scheme 5). An oxidative workup with buffered aqueous H_2_O_2_ proved convenient, allowing for the isolation of compound **15a** on a gram scale in virtually unchanged yield and optical purity; the excess diene was largely recovered by flash chromatography. Although the relevance of *vic*-aminoalcohols as a privileged structural motif does not need any particular illustration, it is worth mentioning that the ester or (Weinreb) amide termini of the products prepared herein provide an additional handle for downstream functionalization. This notion is illustrated by the straightforward conversion of **15a** into lactam **46** and the derived piperidine **47**; heterocycles of this type are endowed with diverse bioactivity.^38^ Another heterocyclic motif of high interest represented by **49** is attained via an essentially quantitative oxa-Michael addition of the hydroxy group of **15a** to the enoate.^39^ Advantage can also be taken from the −NHBoc substituent by neighboring group participation; thus, treatment of **15a** with SOCl_2_ entailed inversion of the secondary −OH group with formation of the cyclic carbamate **48** as a protected *syn*-configured aminoalcohol derivative.^40,41^ This maneuver fills a current gap in coverage since direct access to the *syn*-series via *E,Z*-**13a** as coupling partner, though possible, came along with a modest ee of product **50** before recrystallization (Scheme 5).

**Scheme 5:**
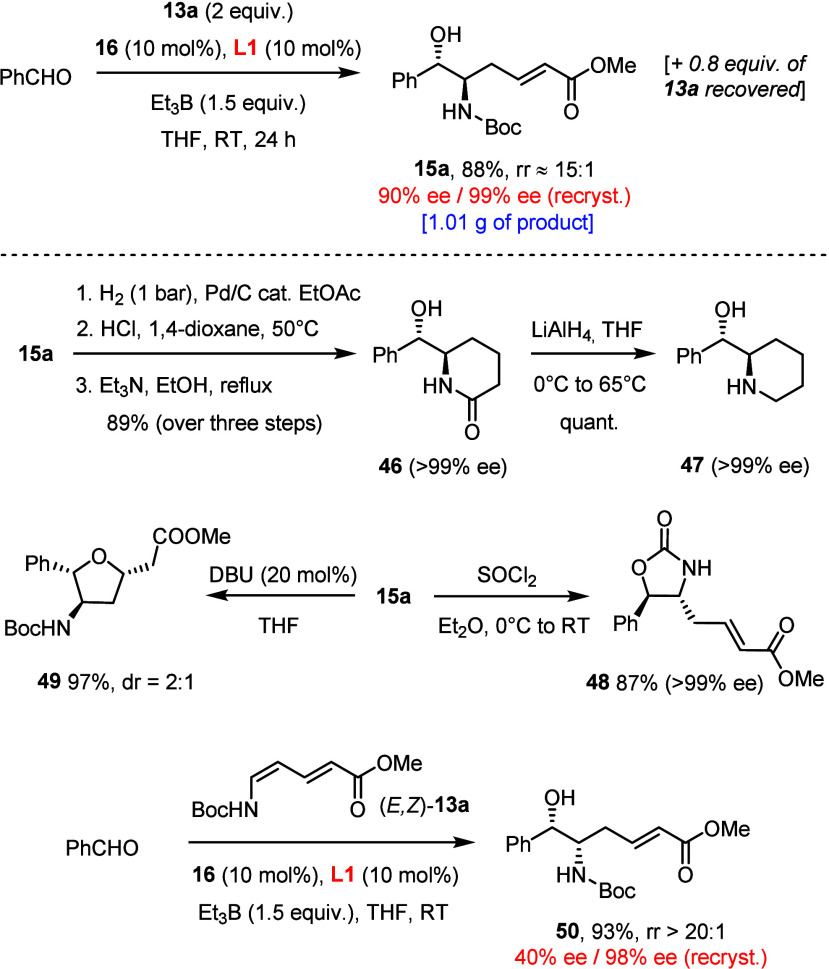
Gram-Scale Experiment and Downstream Functionalization

The results summarized above add another important entry to the growing list of asymmetric transformations, the regiochemical course of which is fundamentally changed by the VAPOL-derived phosphoramidite **L1** as ligand to a Ni(0) catalyst.^1−5^ Strong evidence in the literature suggests that reactions of dienes (or other unsaturated hydrocarbons) with carbonyl derivatives commence with an oxidative cyclometalation,^42,43^ and we have currently no indication that the reductive coupling leading to *vic*-aminoalcohols is triggered otherwise; it likely follows the generic catalytic cycle shown in Scheme 6. Yet, the reasons why **L1** is uniquely effective at altering the outcome and exerting enantiocontrol among a set of ≈50 different ligands tested up to now,^32^ including vaulted siblings such as **L2**, remain unknown. Mechanistic studies prove taxing for a number of reasons;^44^ therefore, we deliberately refrain from drawing a more detailed picture until our ongoing investigations have yielded solid experimental and computational evidence. This desideratum notwithstanding, the method disclosed herein is arguably a valuable complement to the arsenal of catalytic asymmetric transformations that allow precious aminoalcohol derivatives to be formed from readily available substrates by creating both stereocenters while fashioning the interconnecting C–C bond.^22,45^ At the same time, it becomes increasingly apparent that “inverted” nickel-catalyzed coupling reactions are enabling, and their scope is wide.

**Scheme 6:**
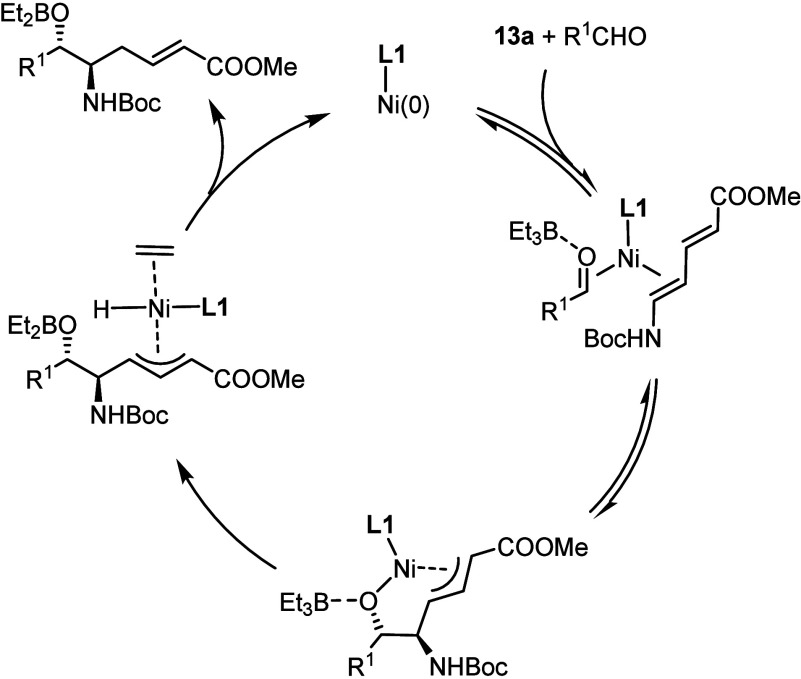
Presumed Catalytic Cycle

## Supporting Information Available

Experimental Section including characterization data, NMR spectra of new compounds, and supporting crystallographic information (PDF)
