# Supplementary material for: Enantioselective Synthesis of vic-Aminoalcohol Derivatives by Nickel-Catalyzed Reductive Coupling of Aldehydes with Protected Amino-pentadienoates
Source: J Am Chem Soc. 2024 Nov 22;146(49):33295–301. doi: 10.1021/jacs.4c12002 (PMC11638964; doi:10.1021/jacs.4c12002)

SUPPORTING INFORMATION

Enantioselective Synthesis of *vic*-Aminoalcohol Derivatives by Nickel-Catalyzed Reductive Coupling of Aldehydes with Protected Amino-pentadienoates

Thilo Bender and Alois Fürstner*

Max-Planck-Institut für Kohlenforschung, 45470 Mülheim/Ruhr, Germany

Email: fuerstner@kofo.mpg.de

Table of Content

Supporting Crystallographic Information S2
Experimental S15
General Information S15
Procedures and Characterization Data S16
Preparation of Dienes and Ligand L2 ..... S16
Ni-catalyzed Reductive Coupling S23
Reaction Screening for Aromatic Aldehydes S23
Reaction Screening for Aliphatic Aldehydes S23
General Procedures S24
Work-up Procedures S24
Notes S25
Control Experiments S27
Determination of the Regioisomer Ratio (rr) S33
Characterization Data and ee-Determination S36
References S79
NMR Spectra of New Compounds S80

Supporting Crystallographic Information

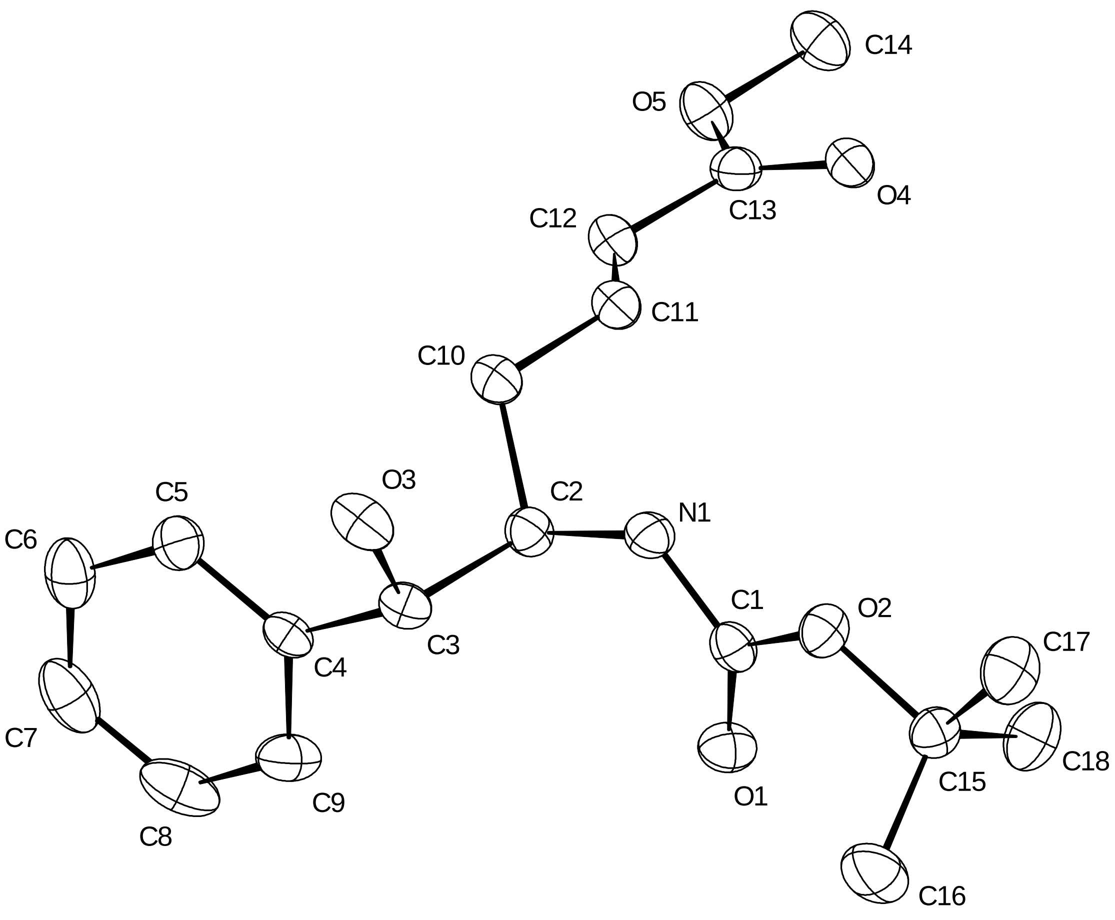

Figure S1. The structure of compound **15a** in the solid state; H atoms have been removed for clarity.

X-ray Crystal Structure Analysis of Compound 15a. C_{18} H_{25} N O_5, M_r = 335.39 g · mol^{-1}, colorless prism, crystal size 0.131 x 0.066 x 0.051 mm^3, orthorhombic, space group $P2_12_12_1$[19], a = 9.0951(3) Å, b = 9.5582(3) Å, c = 18.4316(7) Å, V = 1548.82(11) Å^3, T = 100(2) K, Z = 4, D_{calc} = 1.218 g · cm^3, λ = 1.54178 Å, $\mu(Cu\text{-}K_\alpha)$ = 0.728 mm^{-1}, analytical absorption correction ($T_{\min}$ = 0.95, $T_{\max}$ = 0.98), Bruker AXS Enraf-Nonius KappaCCD diffractometer with a FR591 rotating Cu-anode X-ray source, 4.204 < θ < 71.807°, 62197 measured reflections, 3520 independent reflections, 3214 reflections with $I > 2\sigma(I)$, R_{int} = 0.0626, 222 parameters, S = 1.062, absolute structure parameter = 0.00(7), residual electron density +0.1 (0.70 Å from H1) / -0.2 e · Å^{-3} (0.78 Å from C1). The structure was solved by *SHELXT* and refined by full-matrix least-squares (*SHELXL*) against F^2 to R_1 = 0.030 [$I > 2\sigma(I)$], wR_2 = 0.072. **CCDC-2377029**

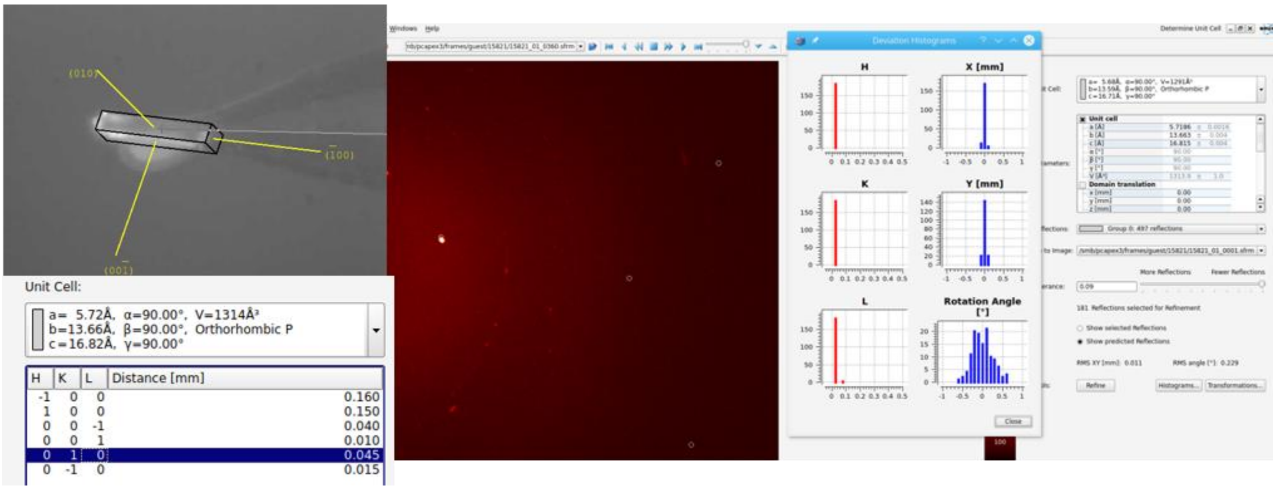

Figure S2. Crystal faces and unit cell determination/refinement of compound **15a**

INTENSITY STATISTICS FOR DATASET # 1

Resolution	#Data	#Theory	%Complete	Redundancy	Mean I	Mean I/s	Rmerge	Rsigma
Inf - 3.29	57	57	100.0	18.49	164.00	105.36	0.0278	0.0087
3.29 - 2.22	124	124	100.0	18.13	50.84	92.96	0.0284	0.0093
2.22 - 1.77	174	174	100.0	19.90	38.69	76.01	0.0367	0.0105
1.77 - 1.54	180	180	100.0	18.68	20.85	56.53	0.0458	0.0134
1.54 - 1.40	177	177	100.0	16.94	12.63	40.70	0.0632	0.0196
1.40 - 1.30	176	176	100.0	13.48	12.74	33.11	0.0666	0.0240
1.30 - 1.22	185	185	100.0	22.07	12.74	39.40	0.0728	0.0224
1.22 - 1.16	166	166	100.0	27.81	10.52	39.20	0.0854	0.0214
1.16 - 1.11	182	182	100.0	26.92	9.67	35.57	0.0897	0.0234
1.11 - 1.06	205	205	100.0	26.39	10.62	37.30	0.0867	0.0223
1.06 - 1.03	143	143	100.0	23.64	7.45	28.30	0.1124	0.0306
1.03 - 0.99	223	223	100.0	22.26	5.41	22.58	0.1341	0.0390
0.99 - 0.97	139	139	100.0	21.35	6.23	26.34	0.1046	0.0332
0.97 - 0.94	190	190	100.0	19.72	3.91	18.90	0.1507	0.0511
0.94 - 0.91	239	239	100.0	18.32	3.28	16.90	0.1699	0.0553
0.91 - 0.90	94	94	100.0	18.64	3.66	19.44	0.1378	0.0467
0.90 - 0.88	176	176	100.0	18.69	2.97	17.06	0.1846	0.0537
0.88 - 0.86	205	210	97.6	9.32	2.38	10.46	0.1998	0.0992
0.86 - 0.84	217	220	98.6	3.05	2.00	6.51	0.1633	0.1542
0.84 - 0.82	221	229	96.5	2.87	2.26	7.71	0.1367	0.1247
0.82 - 0.81	71	121	58.7	1.12	1.61	4.42	0.1979	0.2182
0.91 - 0.81	984	1050	93.7	8.06	2.44	10.58	0.1697	0.1027
Inf - 0.81	3544	3610	98.2	17.29	12.90	31.79	0.0622	0.0213

Table S1. Crystal data and structure refinement of compound **15a**

Identification code	15500	
Empirical formula	$C_{18} H_{25} N O_5$	
Color	colorless	
Formula weight	335.39 g·mol^{-1}	
Temperature	100(2) K	
Wavelength	1.54178 Å	
Crystal system	Orthorhombic	
Space group	$P2_12_12_1$, (no. 19)	
Unit cell dimensions	a = 9.0951(3) Å	α= 90°.
	b = 9.5582(3) Å	β= 90°.
	c = 21.0340(7) Å	γ = 90°.
Volume	1828.54(10) Å^3	
Z	4	
Density (calculated)	1.218 Mg·m^{-3}	
Absorption coefficient	0.728 mm^{-1}	
F(000)	720 e	
Crystal size	0.131 x 0.066 x 0.051 mm^3	
θ range for data collection	4.204 to 71.807°.	
Index ranges	$-11 \le h \le 11$, $-10 \le k \le 11$, $-25 \le l \le 25$	
Reflections collected	62197	
Independent reflections	3520 [R_{int} = 0.0626]	
Reflections with I>2σ(I)	3214	
Completeness to θ = 67.679°	99.6 %	
Absorption correction	Gaussian	
Max. and min. transmission	0.98055 and 0.94823	
Refinement method	Full-matrix least-squares on F^2	
Data / restraints / parameters	3520 / 0 / 222	
Goodness-of-fit on F^2	1.062	
Final R indices [I>2σ(I)]	R_1 = 0.0300	wR^2 = 0.0706
R indices (all data)	R_1 = 0.0350	wR^2 = 0.0724
Absolute structure parameter	0.00(7)	
Extinction coefficient	n/a	
Largest diff. peak and hole	0.115 and -0.148 e·Å^{-3}	

Table S2. Bond lengths [Å] and angles [°] of compound **15a**

O(1)-C(1)	1.223(2)	O(2)-C(1)	1.349(2)
O(2)-C(15)	1.468(2)	O(3)-H(3)	0.8400
O(3)-C(3)	1.421(2)	O(4)-C(13)	1.215(2)
O(5)-C(13)	1.340(2)	O(5)-C(14)	1.447(2)
N(1)-H(1)	0.8800	N(1)-C(1)	1.347(2)
N(1)-C(2)	1.458(2)	C(2)-H(2)	1.0000
C(2)-C(3)	1.538(3)	C(2)-C(10)	1.538(3)
C(3)-H(3A)	1.0000	C(3)-C(4)	1.517(2)
C(4)-C(5)	1.388(3)	C(4)-C(9)	1.392(3)
C(5)-H(5)	0.9500	C(5)-C(6)	1.384(3)
C(6)-H(6)	0.9500	C(6)-C(7)	1.374(4)
C(7)-H(7)	0.9500	C(7)-C(8)	1.387(3)
C(8)-H(8)	0.9500	C(8)-C(9)	1.387(3)
C(9)-H(9)	0.9500	C(10)-H(10A)	0.9900
C(10)-H(10B)	0.9900	C(10)-C(11)	1.497(3)
C(11)-H(11)	0.9500	C(11)-C(12)	1.320(3)
C(12)-H(12)	0.9500	C(12)-C(13)	1.476(3)
C(14)-H(14A)	0.9800	C(14)-H(14B)	0.9800
C(14)-H(14C)	0.9800	C(15)-C(16)	1.512(3)
C(15)-C(17)	1.515(3)	C(15)-C(18)	1.517(3)
C(16)-H(16A)	0.9800	C(16)-H(16B)	0.9800
C(16)-H(16C)	0.9800	C(17)-H(17A)	0.9800
C(17)-H(17B)	0.9800	C(17)-H(17C)	0.9800
C(18)-H(18A)	0.9800	C(18)-H(18B)	0.9800
C(18)-H(18C)	0.9800		
C(1)-O(2)-C(15)	121.06(14)	C(3)-O(3)-H(3)	109.5
C(13)-O(5)-C(14)	115.86(14)	C(1)-N(1)-H(1)	119.2
C(1)-N(1)-C(2)	121.60(15)	C(2)-N(1)-H(1)	119.2
O(1)-C(1)-O(2)	125.74(17)	O(1)-C(1)-N(1)	124.81(17)
N(1)-C(1)-O(2)	109.45(15)	N(1)-C(2)-H(2)	108.6
N(1)-C(2)-C(3)	110.13(15)	N(1)-C(2)-C(10)	108.73(14)
C(3)-C(2)-H(2)	108.6	C(10)-C(2)-H(2)	108.6
C(10)-C(2)-C(3)	112.23(15)	O(3)-C(3)-C(2)	107.07(14)
O(3)-C(3)-H(3A)	108.1	O(3)-C(3)-C(4)	112.84(15)
C(2)-C(3)-H(3A)	108.1	C(4)-C(3)-C(2)	112.58(15)
C(4)-C(3)-H(3A)	108.1	C(5)-C(4)-C(3)	121.64(16)

C(5)-C(4)-C(9)	118.48(18)	C(9)-C(4)-C(3)	119.87(17)
C(4)-C(5)-H(5)	119.5	C(6)-C(5)-C(4)	120.94(19)
C(6)-C(5)-H(5)	119.5	C(5)-C(6)-H(6)	119.8
C(7)-C(6)-C(5)	120.4(2)	C(7)-C(6)-H(6)	119.8
C(6)-C(7)-H(7)	120.3	C(6)-C(7)-C(8)	119.4(2)
C(8)-C(7)-H(7)	120.3	C(7)-C(8)-H(8)	119.8
C(7)-C(8)-C(9)	120.4(2)	C(9)-C(8)-H(8)	119.8
C(4)-C(9)-H(9)	119.8	C(8)-C(9)-C(4)	120.3(2)
C(8)-C(9)-H(9)	119.8	C(2)-C(10)-H(10A)	108.9
C(2)-C(10)-H(10B)	108.9	H(10A)-C(10)-H(10B)	107.7
C(11)-C(10)-C(2)	113.24(16)	C(11)-C(10)-H(10A)	108.9
C(11)-C(10)-H(10B)	108.9	C(10)-C(11)-H(11)	117.4
C(12)-C(11)-C(10)	125.17(18)	C(12)-C(11)-H(11)	117.4
C(11)-C(12)-H(12)	119.0	C(11)-C(12)-C(13)	122.04(18)
C(13)-C(12)-H(12)	119.0	O(4)-C(13)-O(5)	123.67(17)
O(4)-C(13)-C(12)	125.54(18)	O(5)-C(13)-C(12)	110.79(15)
O(5)-C(14)-H(14A)	109.5	O(5)-C(14)-H(14B)	109.5
O(5)-C(14)-H(14C)	109.5	H(14A)-C(14)-H(14B)	109.5
H(14A)-C(14)-H(14C)	109.5	H(14B)-C(14)-H(14C)	109.5
O(2)-C(15)-C(16)	110.83(17)	O(2)-C(15)-C(17)	102.29(15)
O(2)-C(15)-C(18)	109.52(16)	C(16)-C(15)-C(17)	110.77(17)
C(16)-C(15)-C(18)	113.20(19)	C(17)-C(15)-C(18)	109.69(18)
C(15)-C(16)-H(16A)	109.5	C(15)-C(16)-H(16B)	109.5
C(15)-C(16)-H(16C)	109.5	H(16A)-C(16)-H(16B)	109.5
H(16A)-C(16)-H(16C)	109.5	H(16B)-C(16)-H(16C)	109.5
C(15)-C(17)-H(17A)	109.5	C(15)-C(17)-H(17B)	109.5
C(15)-C(17)-H(17C)	109.5	H(17A)-C(17)-H(17B)	109.5
H(17A)-C(17)-H(17C)	109.5	H(17B)-C(17)-H(17C)	109.5
C(15)-C(18)-H(18A)	109.5	C(15)-C(18)-H(18B)	109.5
C(15)-C(18)-H(18C)	109.5	H(18A)-C(18)-H(18B)	109.5
H(18A)-C(18)-H(18C)	109.5	H(18B)-C(18)-H(18C)	109.5

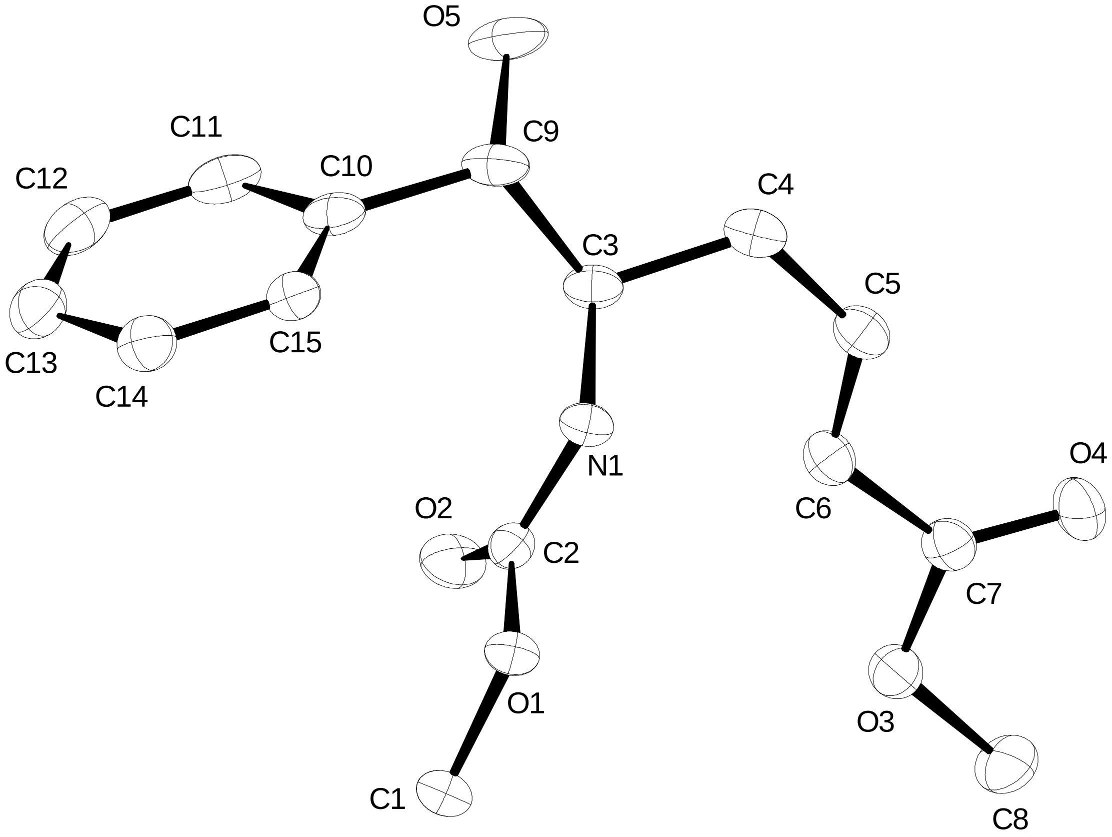

Figure S3. The structure of compound **15b** in the solid state; H atoms have been removed for clarity.

X-ray Crystal Structure Analysis of Compound 15b. $C_{15}\,H_{19}\,N\,O_5$, M_r = 293.31 g · mol^{-1}, colorless prism, crystal size 0.28 x 0.24 x 0.05 mm^3, orthorhombic, space group $P2_12_12_1$[19], a = 5.0555(2) Å, b = 16.6216(7) Å, c = 21.0340(7) Å, V = 1828.54(10) Å^3, T = 100(2) K, Z = 4, D_{calc} = 1.258 g · cm^3, λ = 1.54178 Å, $\mu(Cu\text{-}K_\alpha)$ = 0.789 mm^{-1}, analytical absorption correction ($T_{\min}$ = 0.84, $T_{\max}$ = 0.96), Bruker AXS Enraf-Nonius KappaCCD diffractometer with a FR591 rotating Cu-anode X-ray source, 3.581 < θ < 71.542°, 54998 measured reflections, 2912 independent reflections, 2848 reflections with $I > 2\sigma(I)$, R_{int} = 0.0294, 213 parameters, S = 1.173, absolute structure parameter = 0.05(4), residual electron density +0.2 (2.22 Å from H1A) / -0.2 e · Å^{-3} (0.93 Å from N1). The structure was solved by *SHELXT* and refined by full-matrix least-squares (*SHELXL*) against F^2 to R_1 = 0.035 [$I > 2\sigma(I)$], wR_2 = 0.087. **CCDC-2377028**

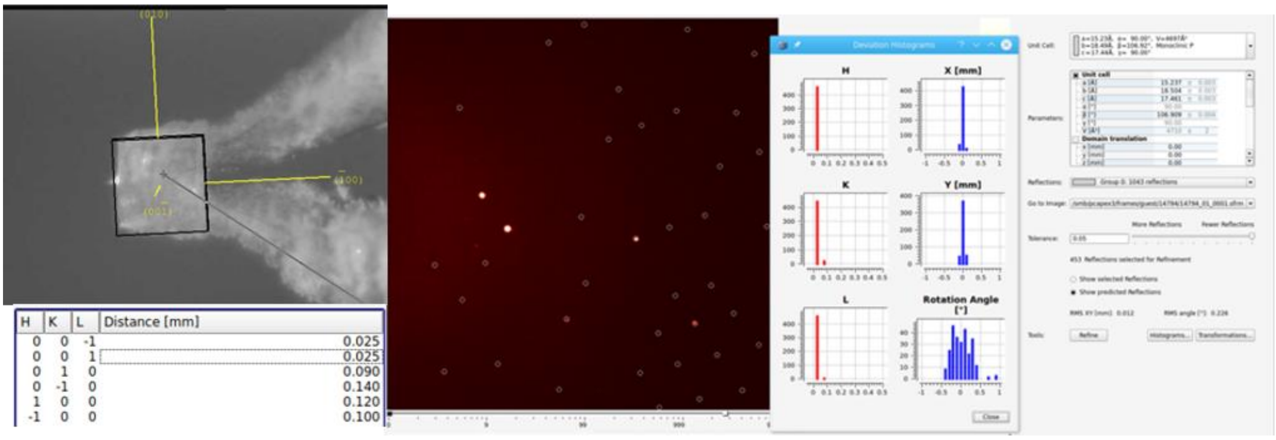

Figure S4. Crystal faces and unit cell determination/refinement of compound **15b**

INTENSITY STATISTICS FOR DATASET # 1

Resolution	#Data	#Theory	%Complete	Redundancy	Mean I	Mean I/s	Rmerge	Rsigma
Inf - 4.05	27	27	100.0	17.22	139.86	100.07	0.0258	0.0086
4.05 - 2.46	60	60	100.0	27.00	87.95	133.13	0.0260	0.0069
2.46 - 1.90	89	89	100.0	29.25	43.25	136.23	0.0263	0.0070
1.90 - 1.63	86	86	100.0	31.99	26.51	123.94	0.0267	0.0069
1.63 - 1.47	91	91	100.0	29.57	19.19	111.92	0.0257	0.0077
1.47 - 1.35	89	89	100.0	28.81	13.17	101.98	0.0302	0.0094
1.35 - 1.26	82	85	96.5	25.33	10.69	96.37	0.0341	0.0098
1.26 - 1.19	91	95	95.8	45.48	12.55	141.74	0.0310	0.0075
1.19 - 1.14	86	86	100.0	48.05	12.57	148.68	0.0315	0.0058
1.14 - 1.09	89	89	100.0	52.19	11.52	150.77	0.0308	0.0057
1.09 - 1.05	83	83	100.0	50.59	8.52	138.84	0.0315	0.0060
1.05 - 1.01	92	93	98.9	48.67	7.39	133.44	0.0347	0.0065
1.01 - 0.97	101	125	80.8	32.82	4.74	102.61	0.0407	0.0082
0.97 - 0.95	70	70	100.0	34.20	5.92	100.31	0.0363	0.0120
0.95 - 0.92	113	113	100.0	33.81	4.82	90.26	0.0407	0.0104
0.92 - 0.90	85	85	100.0	32.36	3.74	92.02	0.0403	0.0091
0.90 - 0.88	81	81	100.0	33.59	3.27	92.07	0.0469	0.0099
0.88 - 0.86	96	96	100.0	14.78	2.67	42.78	0.0834	0.0340
0.86 - 0.84	91	95	95.8	5.80	2.60	14.00	0.1635	0.0568
0.84 - 0.83	52	62	83.9	5.32	1.45	11.81	0.1882	0.0712
0.83 - 0.81	95	116	81.9	3.62	1.82	11.37	0.2445	0.0784
0.91 - 0.81	465	500	93.0	14.19	2.55	40.79	0.0679	0.0383
Inf - 0.81	1749	1816	96.3	30.38	15.08	99.19	0.0301	0.0089

Table S3. Crystal data and structure refinement of compound **15b**

Identification code	14794	
Empirical formula	$C_{15} H_{19} N O_5$	
Color	colorless	
Formula weight	293.31 g · mol^{-1}	
Temperature	100(2) K	
Wavelength	1.54178 Å	
Crystal system	ORTHORHOMBIC	
Space group	**$P2_12_12_1$, (no. 19)**	
Unit cell dimensions	a = 5.0555(2) Å	α= 90°.
	b = 16.6216(7) Å	β= 90°.
	c = 18.4316(7) Å	γ = 90°.
Volume	1548.82(11) Å^3	
Z	4	
Density (calculated)	1.258 Mg · m^{-3}	
Absorption coefficient	0.789 mm^{-1}	
F(000)	624 e	
Crystal size	0.28 x 0.24 x 0.05 mm^3	
θ range for data collection	3.581 to 71.542°.	
Index ranges	-4 ≤ h ≤ 5, -20≤ k ≤ 20, -22 ≤ l ≤ 22	
Reflections collected	54998	
Independent reflections	2912 [R_{int} = 0.0294]	
Reflections with I>2σ(I)	2848	
Completeness to θ = 67.679°	97.5 %	
Absorption correction	Gaussian	
Max. and min. transmission	0.96 and 0.84	
Refinement method	Full-matrix least-squares on F^2	
Data / restraints / parameters	2912 / 0 / 213	
Goodness-of-fit on F^2	1.173	
Final R indices [I>2σ(I)]	R_1 = 0.0353	wR^2 = 0.0865
R indices (all data)	R_1 = 0.0361	wR^2 = 0.0868
Absolute structure parameter	0.05(4)	
Largest diff. peak and hole	0.2 and -0.2 e · Å^{-3}	

Table S4. Bond lengths [Å] and angles [°] of compound **15b**

O(1)-C(1)	1.440(3)	O(1)-C(2)	1.347(3)
O(2)-C(2)	1.223(3)	O(3)-C(7)	1.339(3)
O(3)-C(8)	1.447(3)	O(4)-C(7)	1.209(3)
O(5)-C(9)	1.420(3)	N(1)-H(1)	0.83(4)
N(1)-C(2)	1.341(3)	N(1)-C(3)	1.455(3)
C(3)-H(3)	1.03(3)	C(3)-C(4)	1.528(3)
C(3)-C(9)	1.539(3)	C(4)-C(5)	1.491(3)
C(5)-H(5A)	0.95(3)	C(5)-C(6)	1.327(4)
C(6)-H(6)	0.94(3)	C(6)-C(7)	1.477(3)
C(9)-H(9)	1.04(3)	C(9)-C(10)	1.517(4)
C(10)-C(11)	1.386(4)	C(10)-C(15)	1.398(3)
C(11)-C(12)	1.394(4)	C(12)-C(13)	1.384(4)
C(13)-C(14)	1.384(4)	C(14)-C(15)	1.387(4)
C(2)-O(1)-C(1)	115.3(2)	C(7)-O(3)-C(8)	115.1(2)
C(2)-N(1)-H(1)	119(2)	C(2)-N(1)-C(3)	122.7(2)
C(3)-N(1)-H(1)	118(2)	O(2)-C(2)-O(1)	123.7(2)
O(2)-C(2)-N(1)	125.5(2)	N(1)-C(2)-O(1)	110.8(2)
N(1)-C(3)-H(3)	106.9(13)	N(1)-C(3)-C(4)	110.10(19)
N(1)-C(3)-C(9)	110.33(18)	C(4)-C(3)-H(3)	111.5(14)
C(4)-C(3)-C(9)	110.3(2)	C(9)-C(3)-H(3)	107.7(14)
C(5)-C(4)-C(3)	116.8(2)	C(4)-C(5)-H(5A)	116.5(17)
C(6)-C(5)-C(4)	127.3(2)	C(6)-C(5)-H(5A)	116.2(17)
C(5)-C(6)-H(6)	124.3(18)	C(5)-C(6)-C(7)	120.5(2)
C(7)-C(6)-H(6)	115.3(18)	O(3)-C(7)-C(6)	111.6(2)
O(4)-C(7)-O(3)	122.8(2)	O(4)-C(7)-C(6)	125.7(2)
O(5)-C(9)-C(3)	105.00(18)	O(5)-C(9)-H(9)	110.9(16)
O(5)-C(9)-C(10)	112.3(2)	C(3)-C(9)-H(9)	107.8(16)
C(10)-C(9)-C(3)	112.8(2)	C(10)-C(9)-H(9)	108.0(16)
C(11)-C(10)-C(9)	121.4(2)	C(11)-C(10)-C(15)	118.6(2)
C(15)-C(10)-C(9)	119.9(2)	C(10)-C(11)-C(12)	120.7(2)
C(13)-C(12)-C(11)	120.2(3)	C(12)-C(13)-C(14)	119.5(3)
C(13)-C(14)-C(15)	120.4(2)	C(14)-C(15)-C(10)	120.5(2)

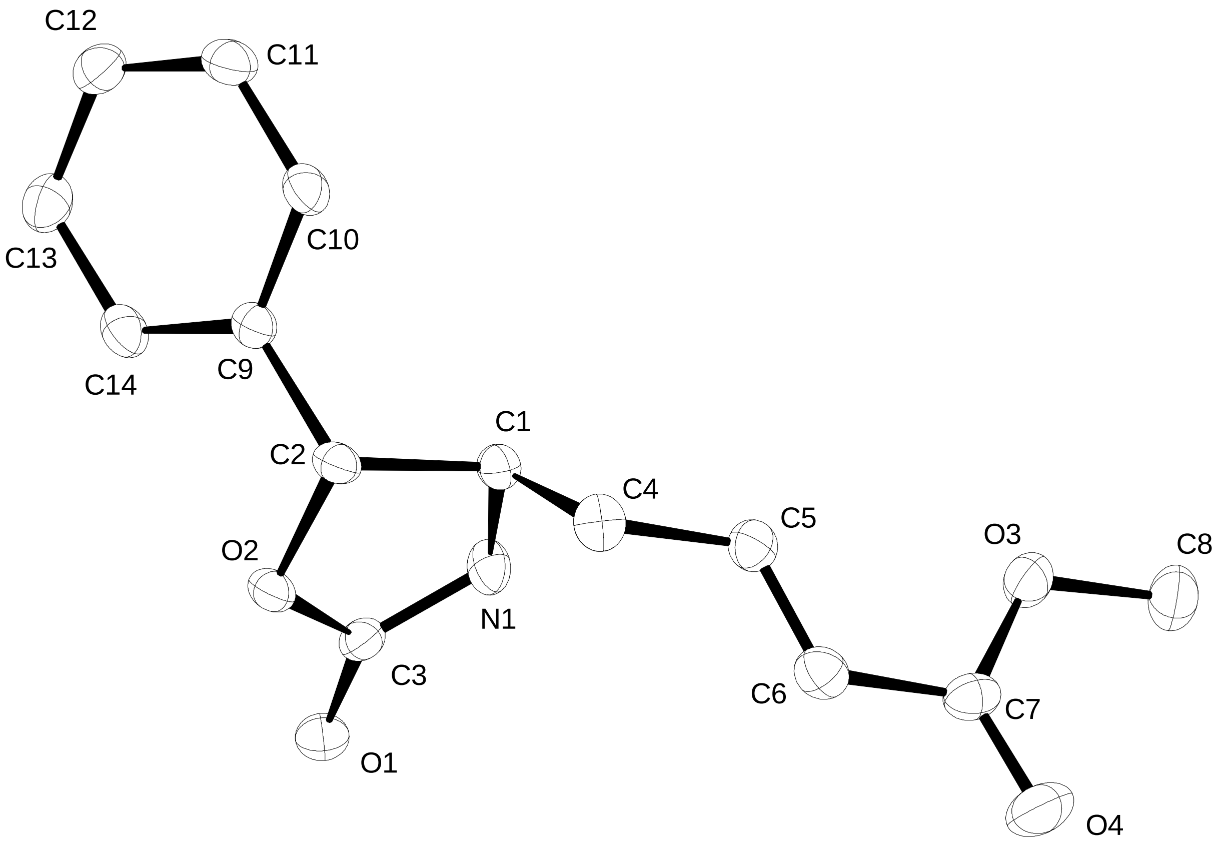

Figure S5. The structure of compound **48** in the solid state; H atoms have been removed for clarity

X-ray Crystal Structure Analysis of Compound 48. $C_{14} H_{15} N O_4$, M_r = 261.27 g · mol^{-1}, colorless needles, crystal size 0.401 x 0.087 x 0.07 mm^3, orthorhombic, space group $P2_12_12_1$[19], a = 5.6941(6) Å, b = 13.5841(14) Å, c = 16.7511(18) Å, V = 1295.7(2) Å^3, T = 100(2) K, Z = 4, D_{calc} = 1.339 g · cm^3, λ = 1.54178 Å, $\mu(Cu\text{-}K_\alpha)$ = 0.820 mm^{-1}, analytical absorption correction ($T_{\min}$ = 0.82, $T_{\max}$ = 0.96), Bruker AXS Enraf-Nonius KappaCCD diffractometer with a FR591 rotating Cu-anode X-ray source, 4.190 < θ < 71.897°, 46340 measured reflections, 2506 independent reflections, 2338 reflections with $I > 2\sigma(I)$, R_{int} = 0.0494, 173 parameters, S = 1.283, absolute structure parameter = -0.18(5), residual electron density +0.2 (0.88 Å from O4) / -0.2 e · Å^{-3} (0.85 Å from C2). The structure was solved by *SHELXT* and refined by full-matrix least-squares (*SHELXL*) against F^2 to R_1 = 0.032 [$I > 2\sigma(I)$], wR_2 = 0.08. **CCDC-2377038**.

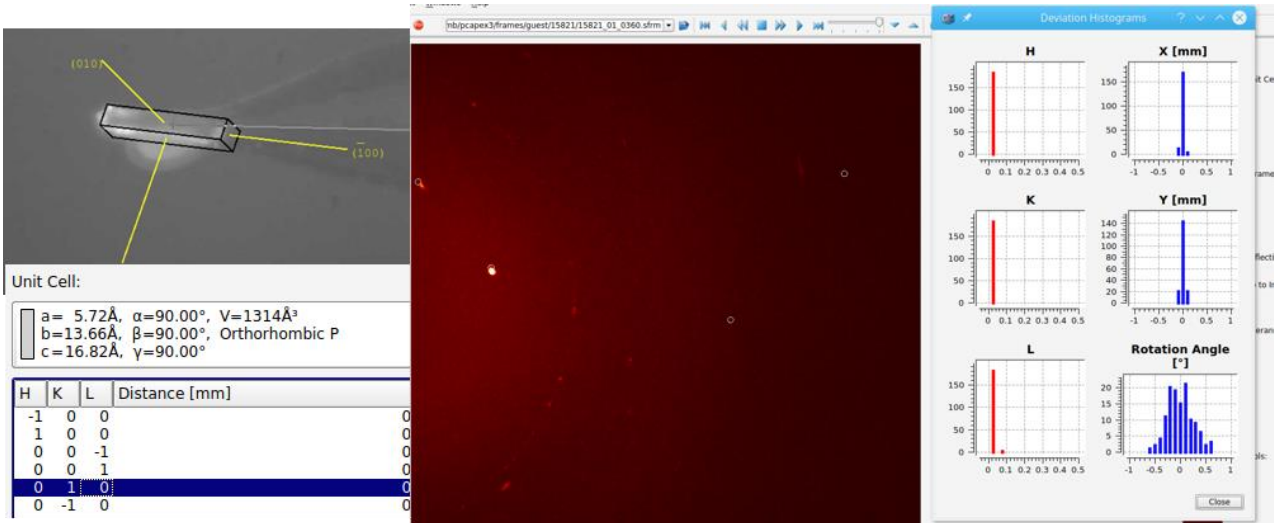

Figure S6. Crystal faces and unit cell determination/refinement of compound **48**

INTENSITY STATISTICS FOR DATASET # 1

Resolution	#Data	#Theory	%Complete	Redundancy	Mean I	Mean I/s	Rmerge	Rsigma
Inf - 4.00	23	23	100.0	17.30	69.62	77.92	0.0302	0.0229
4.00 - 2.45	52	52	100.0	27.10	84.48	117.37	0.0317	0.0077
2.45 - 1.87	75	75	100.0	28.97	31.40	110.72	0.0305	0.0076
1.87 - 1.62	76	76	100.0	31.72	20.44	110.12	0.0324	0.0074
1.62 - 1.45	75	75	100.0	30.07	15.30	91.37	0.0327	0.0087
1.45 - 1.34	76	76	100.0	27.18	9.57	81.43	0.0356	0.0099
1.34 - 1.25	80	80	100.0	26.76	9.06	74.35	0.0525	0.0112
1.25 - 1.18	74	74	100.0	49.50	10.37	100.44	0.0574	0.0079
1.18 - 1.12	82	82	100.0	47.50	10.39	97.13	0.0529	0.0081
1.12 - 1.08	72	72	100.0	46.71	9.22	92.05	0.0603	0.0086
1.08 - 1.04	73	73	100.0	45.90	9.12	92.76	0.0587	0.0085
1.04 - 1.01	66	66	100.0	43.20	5.93	75.71	0.0733	0.0106
1.01 - 0.97	93	93	100.0	37.01	5.26	62.82	0.1094	0.0130
0.97 - 0.94	81	81	100.0	37.33	3.51	54.03	0.1063	0.0152
0.94 - 0.92	67	67	100.0	36.30	3.52	45.04	0.1029	0.0154
0.92 - 0.90	72	72	100.0	34.11	5.12	60.19	0.0854	0.0131
0.90 - 0.88	80	80	100.0	33.20	2.55	45.64	0.1384	0.0176
0.88 - 0.86	77	77	100.0	17.03	2.65	28.84	0.1518	0.0392
0.86 - 0.84	79	79	100.0	7.11	1.86	9.97	0.2613	0.1179
0.84 - 0.82	98	98	100.0	6.42	1.60	9.45	0.2248	0.1145
0.82 - 0.81	37	47	78.7	2.38	1.95	7.29	0.1877	0.2323
0.91 - 0.81	406	416	97.6	15.44	2.42	25.26	0.1307	0.0670
Inf - 0.81	1508	1518	99.3	30.70	11.94	68.15	0.0490	0.0131

Table S5. Crystal data and structure refinement of compound **48**

Identification code	15834	
Empirical formula	$C_{14} H_{15} N O_4$	
Color	colorless	
Formula weight	261.27 g · mol^{-1}	
Temperature	100(2) K	
Wavelength	1.54178 Å	
Crystal system	ORTHORHOMBIC	
Space group	**$P2_12_12_1$, (no. 19)**	
Unit cell dimensions	a = 5.6941(6) Å	α= 90°.
	b = 13.5841(14) Å	β= 90°.
	c = 16.7511(18) Å	γ = 90°.
Volume	1295.7(2) Å^3	
Z	4	
Density (calculated)	1.339 Mg · m^{-3}	
Absorption coefficient	0.820 mm^{-1}	
F(000)	552 e	
Crystal size	0.401 x 0.087 x 0.07 mm^3	
θ range for data collection	4.190 to 71.897°.	
Index ranges	$-7 \le h \le 6$, $-16 \le k \le 16$, $-20 \le l \le 20$	
Reflections collected	46340	
Independent reflections	2506 [R_{int} = 0.0494]	
Reflections with I>2σ(I)	2338	
Completeness to θ = 67.679°	99.9 %	
Absorption correction	Gaussian	
Max. and min. transmission	0.96 and 0.82	
Refinement method	Full-matrix least-squares on F^2	
Data / restraints / parameters	2506 / 0 / 173	
Goodness-of-fit on F^2	1.109	
Final R indices [I>2σ(I)]	R_1 = 0.0316	wR^2 = 0.0747
R indices (all data)	R_1 = 0.0417	wR^2 = 0.0785
Absolute structure parameter	0.18(5)	
Largest diff. peak and hole	0.2 and -0.2 e · Å^{-3}	

Table S6. Bond lengths [Å] and angles [°] of compound **48**

O(1)-C(3)	1.218(3)	O(2)-C(2)	1.444(3)
O(2)-C(3)	1.367(2)	O(3)-C(7)	1.342(3)
O(3)-C(8)	1.441(3)	O(4)-C(7)	1.212(3)
N(1)-C(1)	1.452(3)	N(1)-C(3)	1.332(3)
C(1)-C(2)	1.544(3)	C(1)-C(4)	1.531(3)
C(2)-C(9)	1.519(3)	C(4)-C(5)	1.493(3)
C(5)-C(6)	1.321(3)	C(6)-C(7)	1.469(3)
C(9)-C(10)	1.393(3)	C(9)-C(14)	1.385(3)
C(10)-C(11)	1.387(3)	C(11)-C(12)	1.384(3)
C(12)-C(13)	1.380(3)	C(13)-C(14)	1.392(3)
C(3)-O(2)-C(2)	108.99(15)	C(7)-O(3)-C(8)	116.17(17)
C(3)-N(1)-C(1)	113.15(17)	N(1)-C(1)-C(2)	100.04(16)
N(1)-C(1)-C(4)	112.93(17)	C(4)-C(1)-C(2)	112.20(17)
O(2)-C(2)-C(1)	104.70(15)	O(2)-C(2)-C(9)	110.53(17)
C(9)-C(2)-C(1)	115.08(16)	O(1)-C(3)-O(2)	120.32(19)
O(1)-C(3)-N(1)	129.94(19)	N(1)-C(3)-O(2)	109.73(18)
C(5)-C(4)-C(1)	112.07(17)	C(6)-C(5)-C(4)	124.5(2)
C(5)-C(6)-C(7)	124.8(2)	O(3)-C(7)-C(6)	113.31(17)
O(4)-C(7)-O(3)	122.8(2)	O(4)-C(7)-C(6)	123.9(2)
C(10)-C(9)-C(2)	119.16(18)	C(14)-C(9)-C(2)	121.97(19)
C(14)-C(9)-C(10)	118.81(19)	C(11)-C(10)-C(9)	120.7(2)
C(12)-C(11)-C(10)	120.0(2)	C(13)-C(12)-C(11)	119.7(2)
C(12)-C(13)-C(14)	120.4(2)	C(9)-C(14)-C(13)	120.4(2)

Experimental

General Information

Unless stated otherwise, all reactions were carried out under argon atmosphere in flame-dried Schlenk glassware. Solvents were purified by distillation over the indicated drying agents under argon: toluene ($NaAlEt_4$), THF (Mg/anthracene), Et_2O (Mg/anthracene), *tert*-butyl methyl ether (CaH_2), pentane (Na/K), CH_2Cl_2 (CaH_2), $CHCl_3$ ($CaCl_2$), methanol (Mg). DMF and NEt_3 were dried by an absorption solvent purification system based on molecular sieves. *tert*-Butanol was dried over 3 Å molecular sieves. Dry benzyl alcohol was purchased from Sigma Aldrich and used as received.

Flash chromatography: Merck silica gel 60 (40-63 µm or 15-40 µm). Thin layer chromatography (TLC): Macherey-Nagel precoated plates (POLYGRAM®SIL/UV254); visualization by UV light (254 nm) or by staining with solutions of *p*-anisaldeyhde, ninhydrin, ceric ammonium nitrate, or $KMnO_4$.

NMR: Spectra were recorded on Bruker Avance III 300, 400 MHz or an Avance Neo 600 MHz NMR (equipped with a Bruker BBO CryoProbe) spectrometer in the solvents indicated; chemical shifts are given in ppm relative to TMS, coupling constants (*J*) in Hz. The solvent signals were used as references and the chemical shifts converted to the TMS scale ($CDCl_3$: δ_C = 77.16 ppm; residual $CHCl_3$ in $CDCl_3$: δ_H = 7.26 ppm; [D$_6$]-DMSO: δ_C = 39.52 ppm; residual D_2HSOCD_3 in [D$_6$]-DMSO: δ_H = 2.50 ppm; CD_2Cl_2: δ_C = 53.84 ppm; residual $CDHCl_2$ in CD_2Cl_2: δ_H = 5.32 ppm). All spectra were recorded at 298 K unless stated otherwise. Peak multiplicities are indicated by the following abbreviations: s: singlet, d: doublet, t: triplet, q: quartet, p: pentet, h: hextet, hept: heptet, m: multiplet, dd: double doublet, dt: double triplet, and so on; “br” indicates a broad peak, where the width is large enough to eclipse small coupling constants, which might otherwise be expected.

IR: Spectra were recorded on an Alpha Platinum ATR instrument (Bruker); wavenumbers ($\tilde{\nu}$) are reported in cm^{-1}.

MS (EI): Finnigan MAT 8200 (70 eV), ESI-MS: ESQ3000 (Bruker), Thermo Scientific LTQ-FT, or Thermo Scientific Exactive. HRMS: Bruker APEX III FT-MS (7T magnet), MAT 95 (Finnigan), Thermo Scientific LTQ-FT, or Thermo Scientific Exactive. GC-MS: Shimadzu GCMS-QP2010 Ultra instrument, GC-MS (CI): Q Exactive GC Orbitrap.

HPLC analyses for the determination of the enantiomeric excess (ee) of the individual compounds were conducted on a Shimadzu LC 2020 instrument equipped with a Shimadzu SPD-M20A UV/VIS detector. Solvents (HPLC grade) were purchased and used as received. The exact conditions are stated separately for each compound.

Optical rotations were measured with an A-Krüss Otronic Model P8000-t polarimeter at a wavelength of 589 nm. The values are given as specific optical rotation with exact temperature, concentration (c (10 mg/mL)) and solvent.

Melting points were determined using a BÜCHI B-540.

Aldehydes were purchased from commercial suppliers with the exception of TBDPS- and PMP-protected 2-hydroxyacetaldehyde, which were prepared following literature procedures.[1,2] Liquid aldehydes were distilled and degassed before use, solid aldehydes were recrystallized depending on the purity which was determined by ^{1}H NMR. (*E,E*)-Muconic acid was purchased from Fisher Scientific, (Ni(^{t}Bu-stb)$_3$) was purchased from STREM chemicals. The ligand precursor VAPOL[3] and the phosphoramidite ligand **L1**[4] were prepared following previously established synthetic routes. Triethylborane solutions were prepared from neat triethylborane and the respective solvent. Unless stated otherwise, all other commercially available compounds (TCI Chemicals, Fisher Scientific, Sigma Aldrich, abcr, BLDpharm) were used as received.

Procedures and Characterization Data

Preparation of Dienes and Ligand L2

Dimethyl (2*E*,4*E*)-hexa-2,4-dienedioate (11). HCl (4 M in dioxane, 19.0 mL, 67.8 mmol) was added to a stirred suspension of (*E,E*)-muconic acid (2.41 g, 17.0 mmol) in methanol (60 mL). The suspension was stirred at room temperature for 48 h. The white precipitate was filtered off and dissolved in ethyl acetate (400 mL). The solution was washed with sat. aq. $NaHCO_3$ (150 mL) and brine (150 mL). The organic layer was separated, dried over $MgSO_4$ and filtered. The solvent was removed *in vacuo* and to give the title compound as a colorless crystalline solid (2.70 g, 94%). ^{1}H NMR (400 MHz, $CDCl_3$): δ = 7.38 – 7.27 (m, 1H), 6.27 – 6.12 (m, 1H), 3.78 (s, 3H). ^{1}H NMR data matched those reported in the literature.[5]

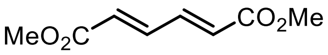

(2*E*,4*E*)-6-Methoxy-6-oxohexa-2,4-dienoic acid (12). A solution of KOH (977 mg, 17.4 mmol) in methanol (9 mL) and water (3 mL) was added dropwise over 2 h to a solution of dimethyl ester **11** (2.51 g, 14.8 mmol) in methanol (150 mL) at 60 °C (bath temperature). Once the addition was complete, stirring was continued for another 2 h at 60 °C. The solvent was evaporated and the residue was dissolved in sat. aq. $NaHCO_3$ (100 mL). The aqueous layer was washed with ethyl acetate (3 x 50 mL) before it was acidified with HCl (6 M) until the product precipitated. Ethyl acetate (100 mL) was added until the precipitate was fully dissolved, the organic layer was separated and the aqueous phase extracted with ethyl acetate (100 mL). The combined organic layers were dried over $MgSO_4$, filtered, and the solvent was removed *in vacuo*. The crude material was purified by recrystallization from *iso*-hexane/ethyl acetate to give the title compound as a crystalline colorless solid (1.35 g, 58%). mp: 162-163 °C; ^{1}H NMR (400 MHz, CD_2Cl_2): δ = 7.49 – 7.24 (m, 2H), 6.24 (td, *J* = 14.8, 1.2 Hz, 2H), 3.76 (s, 3H) ppm; ^{13}C NMR (101 MHz, CD_2Cl_2): δ = 170.6, 166.4, 143.4, 140.8, 129.4, 127.6, 52.3 ppm; IR (ATR): ṽ = 2845, 1724, 1668, 1643, 1428, 1309, 1226, 1171, 1139, 997, 856, 692, 566 cm^{-1}; HRMS (ESI$^-$): *m/z*: calcd. for $C_7H_7O_4$ [M-H]$^-$: 155.03498, found: 155.03509.

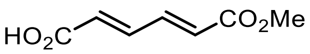

Methyl (2*E*,4*E*)-5-((*tert*-butoxycarbonyl)amino)penta-2,4-dienoate (13a). A flame dried Schlenk flask was charged with carboxylic acid **12** (1.24 g, 7.94 mmol) and $SOCl_2$ (5.75 mL, 79.4 mmol) and the resulting mixture was stirred at 60 °C (bath temperature) for 3 h. The excess $SOCl_2$ was removed *in vacuo* to leave the acid chloride as a colorless solid, which was directly used in the next step.

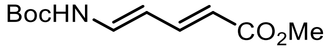

This crude material was dissolved in toluene (18 mL) and a solution of NaN_3 (1.55 g, 23.8 mmol) and tetrabutylammonium bromide (128 mg, 0.40 mmol) in water (18 mL) was added. The mixture was vigorously stirred for 30 min at room temperature. The organic phase was separated and the aqueous layer was extracted with toluene (3 x 15 mL). The combined organic phases were washed with sat. aq. $NaHCO_3$ (20 mL) and brine (20 mL) and were then dried over $MgSO_4$ and filtered.

The volume of the solution was reduced to ca. 20 mL (***Note***: *do not dry completely*!). Dry *tert*-butanol (7.60 mL, 79.4 mmol) was added and the mixture stirred at 100 °C (bath temperature) for 2 h. The solvent was evaporated and the residue was purified by flash chromatography (SiO_2, *iso*-hexane/ethyl acetate, 4:1) to give title compound as a pale yellow solid (1.62 g, 90%). mp = 116-117 °C; ^{1}H NMR (400 MHz, CD_2Cl_2): δ = 7.28 (dd, *J* = 15.2, 11.4 Hz, 1H), 7.06 (t, *J* = 12.6 Hz, 1H), 6.80 (br, 1H), 5.78 (t, *J* = 12.6 Hz, 1H), 5.70 (dd, *J* = 15.2, 0.7 Hz, 1H), 3.68 (s, 3H), 1.47 (s, 9H); ^{13}C NMR (101 MHz, CD_2Cl_2): δ = 168.1, 152.3, 144.5, 135.6, 116.1, 107.8, 82.0, 51.5, 28.3 (3C); IR (ATR): ṽ = 3312, 2972, 1720, 1709, 1637, 1611, 1498, 1258, 1234, 1133, 992, 860, 681 cm^{-1}; HRMS (EI): *m/z*: calcd. for $C_{11}H_{17}NO_4$ [M]$^{+\cdot}$: 227.11521, found: 227.11478.

Methyl (2*E*,4*E*)-5-((methoxycarbonyl)amino)penta-2,4-dienoate (13b). A flame dried Schlenk flask was charged with carboxylic acid **12** (250 mg, 1.60 mmol) and $SOCl_2$ (1.20 mL, 16.5 mmol) and the resulting mixture stirred at 60 °C (bath temperature) for 3 h. The excess $SOCl_2$ was removed *in vacuo* to leave the acid chloride as a colorless solid, which was directly used in the next step.

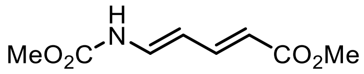

This crude material was dissolved in toluene (5 mL) and a solution of NaN_3 (312 mg, 4.80 mmol) and tetrabutylammonium bromide (25.8 mg, 0.08 mmol) in water (5 mL) was added. The mixture was vigorously stirred for 30 min at room temperature. The organic phase was separated and the aqueous layer was extracted with toluene (3 x 10 mL). The combined organic phases were washed with sat. aq. $NaHCO_3$ (10 mL) and brine (10 mL), dried over $MgSO_4$ and filtered.

The volume of the solution was reduced to ca. 10 mL (***Note***: *do not dry completely*!). Dry methanol (2.60 mL, 64.2 mmol) was added and the mixture was stirred at 100 °C (bath temperature) for 2 h. The solvent was evaporated and the crude product was purified by flash chromatography (SiO_2, *iso*-hexane/ethyl acetate, 2:1 → 1:1) to give the title compound as a colorless solid (267 mg, 90%). mp = 169-171 °C; ^{1}H NMR (400 MHz, [D$_6$]-DMSO): δ = 10.09 (s, 1H), 7.32 (dd, *J* = 15.2, 11.5 Hz, 1H), 7.17 (dd, *J* = 13.7, 10.6 Hz, 1H), 5.89 (t, *J* = 12.7 Hz, 1H), 5.73 (d, *J* = 15.2 Hz, 1H), 3.67 (s, 3H), 3.62 (s, 3H); ^{13}C NMR (101 MHz, [D$_6$]-DMSO): δ = 167.0, 153.9, 145.0, 136.9, 114.7, 108.0, 52.5, 50.9; IR (ATR): ṽ = 3225, 1728, 1680, 1625, 1513, 1434, 1231, 1169, 1014, 737, 603 cm^{-1}; HRMS (EI): *m/z*: calcd. for $C_8H_{11}NO_4$ $[M]^{+\cdot}$: 185.06826, found: 185.06858.

Methyl (2*E*,4*E*)-5-(((benzyloxy)carbonyl)amino)penta-2,4-dienoate (13c). A flame dried Schlenk flask was charged with carboxylic acid **12** (782 mg, 5.01 mmol) and $SOCl_2$ (3.63 mL, 50.1 mmol) and the resulting mixture was stirred at 60 °C (bath temperature) for 3 h. The excess $SOCl_2$ was removed *in vacuo* to leave the acid chloride as a colorless solid, which was directly used in the next step.

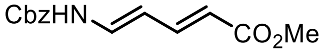

This crude material was dissolved in toluene (12 mL) and a solution of NaN_3 (979 mg, 15.1 mmol) and tetrabutylammonium bromide (83.0 mg, 0.26 mmol) in water (12 mL) was added. The mixture was vigorously stirred for 30 min at room temperature. The organic phase was separated and the aqueous layer was extracted with toluene (3 x 10 mL). The combined organic phases were washed with sat. aq. $NaHCO_3$ (15 mL) and brine (15 mL) and were then dried over $MgSO_4$ and filtered.

The volume of the solution was reduced to ca. 15 mL (***Note***: *do not dry completely*!). Dry benzyl alcohol (1.56 mL, 15.0 mmol) was added and the mixture stirred at 100 °C (bath temperature) for 3 h. The solvent was evaporated and the residue purified by recrystallization from *iso*-hexane/ethyl acetate to give title compound as colorless needles (1.12 g, 86%). mp = 132-133 °C, ^{1}H NMR (400 MHz, CD_2Cl_2): δ = 7.39 – 7.32 (m, 5H), 7.28 (dd, *J* = 15.2, 11.3 Hz, 1H), 7.12 – 7.06 (m, 1H), 7.00 (br, 1H), 5.84 (t, *J* = 12.6 Hz, 1H), 5.73 (dt, *J* = 15.2, 0.7 Hz, 1H), 5.18 (s, 2H), 3.69 (s, 3H); ^{13}C NMR (101 MHz, CD_2Cl_2): δ = 167.9, 153.3, 143.9, 136.1, 134.8, 129.0 (2C), 128.9, 128.6 (2C), 117.0, 109.0, 68.2, 51.6; IR (ATR): ṽ = 3264, 1729, 1696, 1636, 1611, 1521, 1256, 1217, 1148, 993, 694 cm^{-1}; HRMS (EI): *m/z*: calcd. for $C_{14}H_{15}NO_4$ $[M]^{+\cdot}$: 261.09956, found: 261.09958.

Methyl (2*E*,4*E*)-5-((((9*H*-fluoren-9-yl)methoxy)carbonyl)amino)penta-2,4-dienoate (13d). A flame dried Schlenk flask was charged with carboxylic acid **12** (1.20 g, 7.69 mmol) and $SOCl_2$ (5.58 mL, 76.9 mmol) and the mixture stirred at 60 °C (bath temperature) for 3 h. The excess $SOCl_2$ was removed *in vacuo* to leave the acid chloride as a colorless solid, which was directly used in the next step.

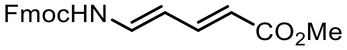

This crude material was dissolved in toluene (18 mL) and a solution of NaN_3 (1.50 g, 23.1 mmol) and tetrabutylammonium bromide (124 mg, 0.38 mmol) in water (18 mL) was added. The mixture was vigorously stirred for 30 min at room temperature until TLC indicated complete conversion. The organic phase was separated and the aqueous layer was extracted with toluene (3 x 15 mL). The combined organic phases were washed with sat. aq. $NaHCO_3$ solution (20 mL) and brine (20 mL) and were then dried over $MgSO_4$ and filtered.

The volume of the solution was reduced to ca. 20 mL (***Note***: *do not dry completely*!). 9-Fluorenylmethanol (1.37 g, 6.99 mmol) was added and the mixture was stirred at 100 °C (bath temperature) for 2 h. The solvent was evaporated and the residue was purified by recrystallization from toluene to give the title compound as a pale yellow solid (2.02 g, 83%). mp = 177-178 °C, ^{1}H NMR (400 MHz, CD_2Cl_2): δ = 7.79 (dt, *J* = 7.6, 1.0 Hz, 2H), 7.60 (dd, *J* = 7.5, 1.0 Hz, 2H), 7.42 (tt, *J* = 7.4, 1.0 Hz, 2H), 7.33 (td, *J* = 7.5, 1.2 Hz, 2H), 7.27 (dd, *J* = 14.9, 11.1 Hz, 1H), 7.06 (t, *J* = 12.5 Hz, 1H), 6.93 (br, 1H), 5.83 (t, *J* = 12.5 Hz, 1H), 5.74 (dt, *J* = 15.2, 0.7 Hz, 1H), 4.52 (d, *J* = 6.6 Hz, 2H), 4.26 (t, *J* = 6.6 Hz, 1H), 3.69 (s, 3H); ^{13}C NMR (101 MHz, CD_2Cl_2): δ = 167.9, 153.2, 144.0, 143.9 (2C), 141.7 (2C), 134.6, 128.2 (2C), 127.5 (2C), 125.3 (2C), 120.4 (2C), 117.1, 109.1, 67.9, 51.6, 47.4; IR (ATR): ṽ = 3295, 1710, 1645, 1620, 1520, 1448, 1264, 1144, 986, 736 cm^{-1}; HRMS (ESI^+): *m/z*: calcd. for $C_{21}H_{19}NO_4Na$ $[M+Na]^+$: 372.12063, found: 372.12064.

Dibenzyl (2*E*,4*E*)-hexa-2,4-dienedioate (S1). Benzyl bromide (1.52 mL, 12.8 mmol) was added to a mixture of (*E*,*E*)-muconic acid (825 mg, 5.81 mmol), KOH (1.49 g, 26.6 mmol) and Bu_4NHSO_4 (4.34 g, 12.8 mmol) in dichloroethane (25 mL) and water (25 mL). The mixture was stirred at 50 °C (bath temperature) overnight. After cooling to room temperature, the organic phase was separated and the aqueous layer extracted with dichloromethane (3 x 50 mL). The combined organic phases were washed with water (100 mL), sat. aq. $NaHCO_3$ (100 mL) and brine (100 mL), and were then dried over $MgSO_4$. The the solvent was removed under reduced pressure and the residue purified by flash chromatography (SiO_2, *iso*-hexane/ethyl acetate, 6:1) to give the title compound as a colorless solid (898 mg, 48%). mp = 103-104 °C; ^{1}H NMR (400 MHz, $CDCl_3$): δ = 7.40 – 7.30 (m, 12H), 6.29 – 6.20 (m, 2H), 5.22 (s, 4H); ^{13}C NMR (101 MHz, $CDCl_3$): δ = 165.8 (2C), 141.3 (2C), 135.7 (2C), 128.8 (4C), 128.6 (2C), 128.5 (4C), 128.4 (2C), 66.9 (2C); IR (ATR): ṽ = 1702, 1612, 1453, 1310, 1236, 1151, 1022, 859, 732, 692 cm^{-1}; HRMS (ESI^+): *m/z*: calcd. for $C_{20}H_{18}O_4Na$ $[M+Na]^+$: 345.10973, found: 345.10984.

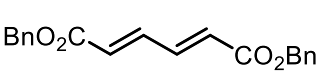

(2*E*,4*E*)-6-(Benzyloxy)-6-oxohexa-2,4-dienoic acid (S2). A solution of KOH (138 mg, 2.46 mmol) in water (5 mL) was added dropwise over 5 h to a solution of dibenzyl (2*E*,4*E*)-hexa-2,4-dienedioate (**S1**, 754 mg, 2.34 mmol) in THF (20 mL) at room temperature and the resulting mixture was stirred overnight. THF was removed under reduced pressure and the remaining aqueous solution was diluted with sat. aq. $NaHCO_3$ (20 mL). The aqueous phase was washed with ethyl acetate (3 x 20 mL) before it was acidified with HCl (6 M) until a solid precipited. Ethyl acetate (ca. 30 mL) was added until the precipitate was fully dissolved, the organic layer was separated and the aqueous phase extracted with ethyl acetate (100 mL). The organic phase was washed with brine (15 mL), dried over $MgSO_4$, and the solvent was removed under reduced pressure. The residue was purified by flash chromatography (SiO_2, *iso*-hexane/ethyl acetate 2:1 + 1% AcOH) to yield the title compound as a pale yellow solid (105 mg, 19%). ^{1}H NMR (400 MHz, $CDCl_3$): δ = 7.46 – 7.32 (m, 7H), 6.36 – 6.13 (m, 2H), 5.23 (s, 2H); ^{13}C NMR (101 MHz, $CDCl_3$): δ = 170.5, 165.7, 143.1, 140.9, 135.7, 129.2, 128.8 (2C), 128.6, 128.5 (2C), 127.6, 67.0; IR (ATR): ṽ = 2938, 1710, 1389, 1240, 1024, 989, 825, 608 cm^{-1}; HRMS (ESI^-): *m/z*: calcd. for $C_{13}H_{11}O_4$ $[M-H]^-$: 231.06629, found: 231.06634.

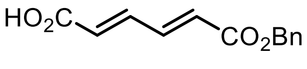

Benzyl (2*E*,4*E*)-5-((*tert*-butoxycarbonyl)amino)penta-2,4-dienoate (S3). A flame dried Schlenk flask was charged with carboxylic acid **S2** (120 mg, 0.52 mmol) and $SOCl_2$ (1 mL, 13.8 mmol) and the resulting mixture was stirred at 60 °C (bath temperature) for 3 h. The excess $SOCl_2$ was removed *in vacuo* to leave the acid chloride as a colorless solid, which was directly used in the next step.

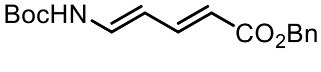

This crude material was dissolved in toluene (3 mL) and a solution of NaN_3 (100.8 mg, 1.55 mmol) and tetrabutylammonium bromide (8.3 mg, 0.03 mmol) in water (3 mL) was added. The mixture was vigorously stirred for 1 h at room temperature. The organic phase was separated and the aqueous phase was extracted with toluene (3 x 5 mL). The combined organic phases were washed with sat. aq. $NaHCO_3$ (5 mL) and brine (5 mL) and were then dried over $MgSO_4$.

The volume of the solution was reduced to ca. 3 mL (***Note***: *do not dry completely*!). Dry *tert*-butanol (1.00 mL, 10.7 mmol) was added and the mixture was stirred at 100 °C (bath temperature) for 2 h. When TLC indicated full conversion, the solvent was evaporated and the residue purified by flash chromatography (SiO_2, *iso*-hexane/ethyl acetate, 7:1) to give the title compound as an off-white solid (125 mg, 80%). ^{1}H NMR (400 MHz, CD_2Cl_2): δ = 7.37 – 7.30 (m, 6H), 7.08 (t, *J* = 12.7 Hz, 1H), 6.78 (d, *J* = 10.4 Hz, 1H), 5.81 – 5.73 (m, 2H), 5.15 (s, 2H), 1.47 (s, 9H); ^{13}C NMR (101 MHz, CD_2Cl_2): δ = 167.3, 152.2, 144.8, 137.1, 135.6, 128.9 (2C), 128.4 (2C), 128.4, 116.2, 107.8, 82.1, 66.1, 28.2 (3C); IR (ATR): ṽ = 3304, 1709, 1636, 1502, 1255, 1125, 998, 863 cm^{-1}; HRMS (ESI$^+$): *m/z*: calcd. for $C_{17}H_{21}NO_4Na$ $[M+Na]^+$: 326.13628, found: 326.13617.

***tert*-Butyl formylcarbamate (S4).**[6] A flame dried pressure Schlenk was charged with *tert*-butyl carbamate (4.11 g, 35.1 mmol), toluene (35 mL) and dimethylformamide dimethylacetal (14.0 mL, 105 mmol). The mixture was stirred at 90 °C (bath temperature) for 3 h. After cooling to room temperature, the solvent was removed under reduced pressure to give a pale yellow oil, which solidified on standing. The solid was dissolved in water (6 mL) and acetic acid (17 mL) and the mixture stirred at room temperature overnight. The mixture was diluted with *tert*-butyl methyl ether (50 mL), washed with water (6 x 25 mL) and brine (25 mL), dried over $MgSO_4$, and concentrated under reduced pressure. The obtained colorless oil was re-dissolved in the minimum amount of *n*-heptane (20 mL), which was then evaporated to give *tert*-butyl formylcarbamate as a colorless solid (4.55 g, 89%). ^{1}H NMR (400 MHz, $CDCl_3$): δ = 8.91 (d, J = 10.4 Hz, 1H), 7.30 (br, 1H), 1.53 (s, 9H). ^{1}H NMR data matched those reported in the literature.[6]

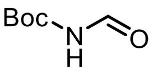

(*E*)-3-Methoxycarbonyl-2-propenylidenetriphenylphosphorane (S5). A flame dried Schlenk flask was charged with triphenylphosphine (5.36 g, 20.4 mmol), toluene (30 mL) and methyl-*trans*-4-brom-2-butenoate (3.13 g, 18.5 mmol). The solution was stirred at room temperature for 24 h leading to the precipitation of a white solid. The solid was filtered off, washed with *tert*-butyl methyl ether (100 mL) and dried in air for 1 h. The solid was finely ground to give a powder that was further dried *in vacuo* to yield the corresponding triphenylphosphonium bromide (8.16 g, quant.).

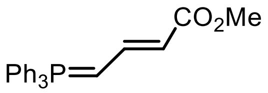

This compound was suspended in water (800 mL) and NaOH (1 M, 18.6 mL, 18.6 mmol) was added dropwise, causing the formation of a yellow-orange precipitate. After stirring the suspension at room temperature for 20 min, the solid was filtered off and dissolved in dichloromethane (150 mL). The organic phase was washed with brine (50 mL), dried over $MgSO_4$, filtered and the solvent was removed under reduced pressure to afford the title compound as an orange solid, which was directly used in the next step (5.77 g, 87%).

Methyl (2*E*,4*Z*)-5-((*tert*-butoxycarbonyl)amino)penta-2,4-dienoate ((*E*,*Z*)-13a). A flame dried pressure Schlenk flask was charged with *tert*-butyl formylcarbamate (**S4**, 649 mg, 4.47 mmol), (*E*)-3-methoxycarbonyl-2-propenylidenetriphenylphosphorane (**S5**, 4.84 g, 13.4 mmol) and chloroform (40 mL). The mixture was stirred at reflux temperature for 17 h. The solvent was removed under reduced pressure and the residue was purified by flash chromatography (SiO_2, *iso*-hexane/ethyl acetate, 7:1 + 1% NEt_3 → *iso*-hexane/ethyl acetate, 5:1 + 1% NEt_3) to afford the title compound as a colorless viscous oil (410 mg, 40%). ^{1}H NMR (400 MHz, CD_2Cl_2): δ = 7.56 – 7.49 (m, 1H), 7.29 (d, *J* = 11.3 Hz, 1H), 6.75 (t, *J* = 10.6 Hz, 1H), 5.81 (dt, *J* = 14.9, 0.8 Hz, 1H), 5.37 (t, *J* = 10.8 Hz, 1H), 3.71 (s, 3H), 1.47 (s, 9H); ^{13}C NMR (101 MHz, CD_2Cl_2): δ = 168.1, 152.4, 137.6, 130.8, 118.2, 104.3, 81.8, 51.8, 28.3 (3C); IR (ATR): ṽ = 3307, 2979, 1695, 1631, 1492, 1233, 1139, 1006, 857 cm^{-1}; HRMS (EI): *m/z*: calcd. for $C_{11}H_{17}NO_4$ $[M]^{+\cdot}$: 227.11521, found: 227.11501.

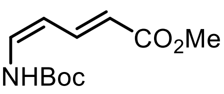

(*E*)-3-(1,3-Dioxoisoindolin-2-yl)acrylaldehyde (S6). A flame dried Schlenk flask was charged with DMF (4 mL). $POCl_3$ (0.46 mL, 4.91 mmol) was added dropwise at 0 °C before the resulting mixture was allowed to warm to room temperature. N-Vinylphtalimide (500 mg, 2.89 mmol) in DMF (2 mL) was added and the resulting solution was stirred at 70 °C (bath temperature) for 3 h. The mixture was

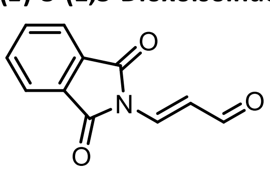

cooled to 0 °C for 2 h, causing the precipitation of a yellow solid. The solvent was decanted and the residual solid was washed with cold chloroform (3 x 1 mL). The obtained pale-yellow solid was dried *in vacuo* before it was used in the next step.

A solution of sodium acetate (31.3 mg, 0.38 mmol) in water (2 mL) was added to the solid material and the resulting mixture was stirred at 50 °C (bath temperature) for 1 h. After cooling to room temperature, chloroform (10 mL) was added to the suspension and the mixture was stirred until a clear two-phase system had formed. The organic layer was separated and washed with water (2 mL), dried over $MgSO_4$ and filtered. The solvent was removed under reduced pressure to afford a pale-yellow solid, which was purified by flash chromatography (SiO_2, toluene/ *tert*-butyl methyl ether, 10:1) to give the title compound as an off-white solid (169 mg, 29%). mp = 151-152 °C, ^{1}H NMR (400 MHz, $CDCl_3$): δ = 9.61 (d, *J* = 7.9 Hz, 1H), 8.01 – 7.96 (m, 2H), 7.88 – 7.84 (m, 2H), 7.72 (d, *J* = 14.8 Hz, 1H), 7.22 (dd, *J* = 14.8, 7.9 Hz, 1H); ^{13}C NMR (101 MHz, $CDCl_3$): δ = 193.1, 165.1 (2C), 138.2, 135.7 (2C), 131.5 (2C), 124.7 (2C), 119.0; IR (ATR): ṽ = 1728, 1676, 1629, 1364, 1139, 1065, 976, 715 cm^{-1}; HRMS (ESI$^+$): *m/z*: calcd. for $C_{11}H_7NO_3Na$ $[M+Na]^+$: 224.03181, found: 224.03188.

Methyl (2*E*,4*E*)-5-((*tert*-butoxycarbonyl)(methyl)amino)penta-2,4-dienoate (17a). In a flame dried Schlenk flask, sodium hydride (41.2 mg, 1.72 mmol) was suspended in THF (10 mL). A solution of diene **13a** (300 mg, 1.32 mmol) in THF (5 mL) was added dropwise at 0°C, causing a color change to intense yellow. Methyl iodide (0.41 mL, 6.60 mmol) was added and the mixture was allowed to warm to room temperature and stirred for 1 h. The mixture was diluted with ethyl acetate (10 mL) and the reaction carefully quenched with sat. aq. $NaHCO_3$ solution (5 mL). The aqueous phase was extracted with ethyl acetate (3 x 10 mL). The combined organic phases were washed with brine (15 mL), dried over $MgSO_4$, filtered, and the solvent was removed under reduced pressure to furnish the title compound as a yellow oil (308 mg, 97%). ^{1}H NMR (600 MHz, CD_2Cl_2): δ = 7.54 (d, *J* = 14.0 Hz, 1H), 7.34 (ddd, *J* = 15.1, 11.2, 0.7 Hz, 1H), 5.74 (d, *J* = 15.1 Hz, 1H), 5.62 (dd, *J* = 13.8, 11.3 Hz, 1H), 3.68 (s, 3H), 3.06 (s, 3H), 1.50 (s, 9H); ^{13}C NMR (151 MHz, CD_2Cl_2): δ = 168.1, 152.6, 145.3, 139.9, 115.8, 106.4, 82.7, 51.4, 31.1, 28.2 (3C); *Note: the signals at 152.6 ppm and 31.1 ppm have very low intensity.* IR (ATR): ṽ = 2978, 1707, 1618, 1432, 1370, 1321, 1239, 1121, 1051, 991, 860 cm^{-1}; HRMS (EI): *m/z*: calcd. for $C_{12}H_{19}NO_4$ $[M]^{+\cdot}$ 241.13086, found: 241.13086.

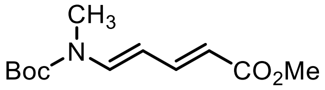

Methyl (2*E*,4*E*)-5-(benzyl(*tert*-butoxycarbonyl)amino)penta-2,4-dienoate (17b). In a flame dried Schlenk flask, sodium hydride (37.0 mg, 1.54 mmol) was suspended in THF (10 mL). A solution of diene **13a** (250 mg, 1.10 mmol) in THF (5 mL) was added dropwise at 0 °C, causing a color change to intense yellow. Benzyl bromide (0.26 mL, 2.20 mmol) was added and stirring continued at room temperature for 24 h. The mixture was diluted with ethyl acetate (10 mL) and the reaction carefully quenched with sat. aq. $NaHCO_3$ solution (5 mL). The organic phase was separated and the aqueous layer extracted with ethyl acetate (3 x 10 mL). The combined organic phases were washed with brine (15 mL), dried over $MgSO_4$, filtered and the solvent was removed under reduced pressure. The residue was purified by flash chromatography (pentane/ethyl acetate, 10:1 → 1:1) to give the product as a pale yellow solid (297 mg, 85%). mp = 94-96 °C; ^{1}H NMR (400 MHz, CD_2Cl_2): δ = 7.58 (d, *J* = 14.1 Hz, 1H), 7.36 – 7.24 (m, 4H), 7.20 – 7.17 (m, 2H), 5.63 (d, *J* = 15.1 Hz, 1H) 5.59 (t, *J* = 13.2 Hz, 1H) , 4.78 (s, 2H), 3.66 (s, 3H), 1.50 (s, 9H); ^{13}C NMR (151 MHz, CD_2Cl_2): δ = 168.0, 152.9, 145.0, 138.9, 137.2, 129.0 (2C), 127.6, 126.7 (2C), 116.1, 107.4, 83.2, 51.5, 48.0, 28.2 (3C); IR (ATR): ṽ = 1711, 1622, 1386, 1240, 1133 cm^{-1}; HRMS (EI): *m/z*: calcd. for $C_{18}H_{23}NO_4$ $[M]^{+\cdot}$: 317.16216, found: 317.16173.

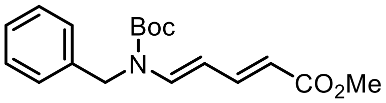

***tert*-Butyl ((1*E*,3*E*)-5-(dimethylamino)-5-oxopenta-1,3-dien-1-yl)carbamate (17c)**. Trimethyl-

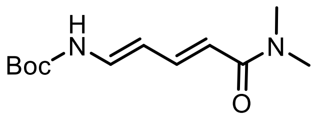

aluminium (2.0 M in hexane, 1.45 mL, 2.90 mmol) was added dropwise to a solution of dimethylamine hydrochloride (236 mg, 2.90 mmol) in dichloromethane (5 mL) at 0 °C (***Caution***: CH_4 formation!). The mixture was allowed to warm to room temperature and stirred for 30 min. A solution of diene **13a** (219 mg, 0.96 mmol) in dichloromethane (5 mL) was added and the resulting orange mixture was stirred at room temperature for 48 h. The mixture was cooled to 0 °C before the reaction was carefully quenched by dropwise addition of sat. aq. NH_4Cl solution (10 mL) followed by sat. aq. Rochelle's salt solution (15 mL). The mixture was diluted with dichloromethane (20 mL) and vigorously stirred for 1 h. The organic phase was separated and the aqueous layer extracted with dichloromethane (3 x 30 mL). The combined organic phases were washed with water (30 mL), dried over $MgSO_4$, filtered, and the solvent was removed under reduced pressure. The residue was purified by flash chromatography (pentane/ethyl acetate, 1:1 → ethyl acetate) to furnish the title compound as a pale yellow solid (102 mg, 44%). mp = 199-201 °C, ^{1}H NMR (400 MHz, $CDCl_3$): δ = 7.28 (dd, *J* = 14.6, 11.4 Hz, 1H), 6.99 (t, *J* = 12.2 Hz, 1H), 6.69 (br, 1H), 6.15 (d, *J* = 14.6 Hz, 1H), 5.79 (t, *J* = 11.8 Hz, 1H), 3.03 (s, 6H), 1.47 (s, 9H); ^{13}C NMR (101 MHz, $CDCl_3$): δ = 167.3, 152.1, 141.7, 133.5, 115.9, 108.5, 81.6, 36.5 (2C), 28.3 (3C); IR (ATR): ṽ = 3203, 2979, 1725, 1646, 1504, 1255, 1158, 1124 cm^{-1}; HRMS (EI): *m/z*: calcd. for $C_{12}H_{20}N_2O_3$ $[M]^{+\cdot}$: 240.14684, found: 240.14684.

***tert*-Butyl ((1*E*,3*E*)-5-(methoxy(methyl)amino)-5-oxopenta-1,3-dien-1-yl)carbamate** (**17d**). Trimethyl

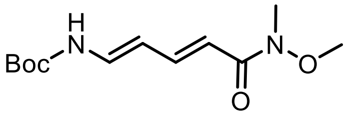

aluminium (2.0 M in hexane, 1.65 mL, 3.30 mmol) was added dropwise to a solution of *N*,*O*-dimethylhydroxylamine hydrochloride (322 mg, 3.30 mmol) in anhydrous dichloromethane (6 mL) at 0 °C (***Caution***: CH_4 formation!). The mixture was warmed to room temperature and stirred for 30 min. A solution of diene **13a** (250 mg, 1.10 mmol) in dichloromethane (6 mL) was added and the resulting yellow solution was stirred at room temperature for 48 h. The mixture was cooled to 0 °C before the reaction was carefully quenched by dropwise addition of sat. aq. NH_4Cl solution (10 mL), followed by the addition of sat. aq. Rochelle's salt solution (15 mL). The mixture was diluted with dichloromethane (20 mL) and vigorously stirred for 1 h. The organic layer was separated and the aqueous phase extracted with dichloromethane (3 x 30 mL). The combined organic phases were washed with water (30 mL), dried over $MgSO_4$, filtered, and the solvent was removed under reduced pressure. The residue was purified by flash chromatography (pentane/methyl-*tert*-butyl ether, 3:1 + 1% NEt_3 → 1:3 + 1% NEt_3) to give the title compound as a colorless solid (215 mg, 76%). mp = 178-180 °C, ^{1}H NMR (400 MHz, CD_2Cl_2): δ = 7.27 (ddd, *J* = 15.0, 11.4, 0.7 Hz, 1H), 7.05 (dd, *J* = 13.8, 11.5 Hz, 1H), 6.84 (br, 1H), 6.31 (d, *J* = 15.0 Hz, 1H), 5.85 (t, *J* = 12.7 Hz, 1H), 3.68 (s, 3H), 3.19 (s, 3H), 1.47 (s, 9H); ^{13}C NMR (101 MHz, CD_2Cl_2): δ = 167.9, 152.3, 142.6, 134.7, 114.8, 108.6, 81.8, 62.0, 32.6, 28.3 (3C); IR (ATR): ṽ = 3241, 2976, 1726, 1648, 1586, 1503, 1251, 1147, 1090, 999 cm^{-1}; HRMS (ESI$^+$): *m/z*: calcd. for $C_{12}H_{20}N_2O_4Na$ $[M+Na]^+$: 279.13153, found: 279.13157.

Ethyl (2*E*,4*E*)-5-(1,3-dioxoisoindolin-2-yl)penta-2,4-dienoate (19). A flame dried Schlenk flask was

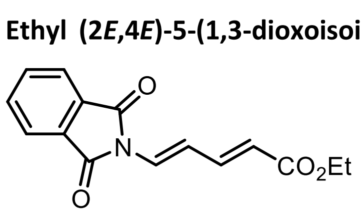

charged with sodium hydride (25.5 mg, 1.06 mmol) and THF (1 mL). The suspension was cooled to 0 °C and triethyl phosphonoacetate (0.23 mL, 1.16 mmol) was added dropwise. After stirring at 0 °C for 10 min, a solution of (*E*)-3-(1,3-dioxoisoindolin-2-yl)acrylaldehyde **S6** (120 mg, 0.60 mmol) in THF (3 mL) was added dropwise, causing an immediate color change to yellow. Upon complete addition of the aldehyde, the mixture was allowed to warm to room temperature and stirring was continued for 18 h. The mixture was diluted with *tert*-butyl methyl ether (20 mL) and the reaction quenched upon addition of sat. aq. NH_4Cl (20 mL). The aqueous layer was extracted with *tert*-butyl methyl ether (2 x 30 mL). The combined organic phases were washed with brine (20 mL), dried over $MgSO_4$, filtered, and the solvent was removed under

reduced pressure. The residue was purified by flash chromatography (SiO_2, *iso*-hexane/ethyl acetate, 3:1 → 2:1) to furnish the title compound as a neon yellow solid (119 mg, 73%). mp = 149-150 °C, ^{1}H NMR (400 MHz, $CDCl_3$): δ = 7.95 – 7.88 (m, 2H), 7.81 – 7.76 (m, 2H), 7.43 – 7.31 (m, 2H), 7.23 – 7.15 (m, 1H), 6.07 – 5.99 (m, 1H), 4.23 (q, *J* = 7.1 Hz, 2H), 1.31 (t, *J* = 7.1 Hz, 3H); ^{13}C NMR (101 MHz, $CDCl_3$): δ = 167.0, 165.9 (2C), 143.3, 135.1 (2C), 131.7 (2C), 126.4, 124.1 (2C), 121.9, 117.5, 60.5, 14.5; IR (ATR): ṽ = 1717, 1698, 1622, 1367, 1203, 1130, 1075, 1015, 866, 711 cm^{-1}; HRMS (EI): *m/z*: calcd. for $C_{15}H_{13}NO_4$ $[M]^{+\cdot}$: 271.08391, found: 271.08422.

Phosphoramidite (*R*)-7,7'-(*t*-Bu)$_2$-VANPhos-NEt$_2$ (L2). An oven-dried pressure Schlenk flask was charged with (*R*)-7,7'-(*t*-Bu)$_2$-VANOL (200 mg, 0.36 mmol)[7] and toluene (2.0 mL). Tris(diethylamino)phosphine (111 µL, 0.40 mmol) was added and the mixture stirred at 110 °C (bath temperature) for 24 h. After cooling to room temperature, the solution was concentrated *in vacuo* and the residue was purified by flash chromatography (SiO_2, pentane/*tert*-butyl methyl ether 100:1 → 30:1) to give phosphoramidite **L2** as a colorless solid (215 mg, 91%). $[\alpha]_D^{20}$ = +87.6 (c = 0.50, $CHCl_3$); ^{1}H NMR (400 MHz, C_6D_6): δ = 8.73 (d, *J* = 1.9 Hz, 1H), 8.63 (d, *J* = 2.0 Hz, 1H), 7.65 (d, *J* = 8.6 Hz, 1H), 7.60 (d, *J* = 8.7 Hz, 1H), 7.51 – 7.44 (m, 3H), 7.41 (s, 1H), 6.94 – 6.86 (m, 2H), 6.83 – 6.68 (m, 8H), 3.19 (ddq, *J* = 14.1, 9.5, 7.1 Hz, 2H), 2.87 (ddq, *J* = 14.0, 10.7, 7.0 Hz, 2H), 1.39 (d, *J* = 4.8 Hz, 18H), 0.88 (t, *J* = 7.0 Hz, 6H); ^{13}C NMR (101 MHz, C_6D_6): δ = 149.4, 149.0, 148.9, 148.8, 148.6, 141.4, 141.3, 140.9, 140.7, 133.3, 133.0, 129.7, 129.3, 128.4, 128.2, 127.42, 127.40, 127.3, 126.5, 126.4, 126.10, 126.07, 125.91, 125.86, 125.8, 124.5, 124.41, 124.38, 118.5 (2C), 117.6 (2C), 39.5, 39.3, 35.3, 35.2, 31.5 (3C), 31.3 (3C), 15.27, 15.25; ^{31}P NMR (162 MHz, C_6D_6): δ = 151.1; IR (ATR): ṽ = 2962, 2867, 2279, 1595, 1495, 1460, 1370, 1319, 1204, 1177, 1112, 1022, 929, 911, 824, 764 cm^{-1}; HRMS (CI): *m/z*: calcd. for $C_{44}H_{47}NO_2P$ $[M+H]^+$: 652.33389, found: 652.33408.

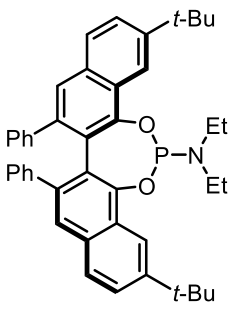

Ni-catalyzed Reductive Coupling

Reaction Screening for Aromatic Aldehydes

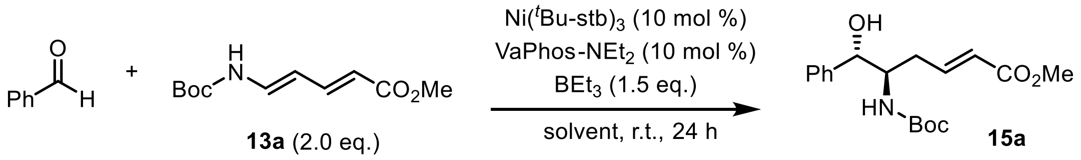

Entry	Solvent	rr	Isolated Yield	ee
**1**	**THF**	**> 20:1**	**91%**	**91%**
2	MTBE	> 20:1	84%	86%
3	Et_2O	> 20:1	81%	85%
4	1,4-dioxane	> 20:1	88%	87%
5	PhMe	> 20:1	67%[a]	41%

[a] 85% conversion only; MTBE = *tert*-butyl methyl ether

Reaction Screening for Aliphatic Aldehydes

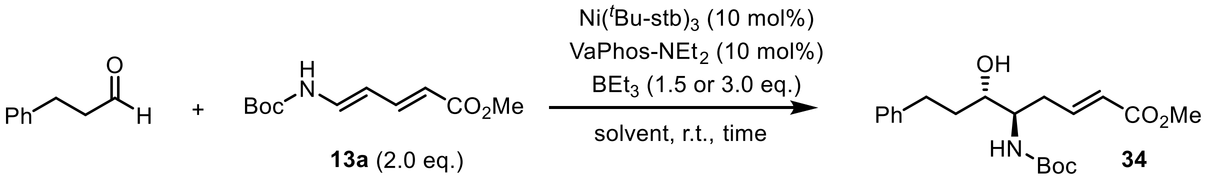

Entry	Solvent	time	Conversion	rr	Isolated Yield	ee
1	THF[a]	18 h	~ 50%	1 : 2	18%	80%
2	PhMe[a,b]	72 h	~ 70%	4 : 1	46%	-
3	$C_6H_5CF_3$[b]	48 h	~ 30%	3 : 1	-	-
4	PhMe[c]	18 h	< 5%	-	-	-
5	PhMe[b]	72 h	~ 75%	4 : 1	51%	92%
6	PhMe[d]	72 h	~ 70%	4 : 1	-	-
**7**	**PhMe[e]**	**72 h**	**100%**	**4 : 1**	**72%**	**95%**

[a] 1.1 eq. of the diene was used; [b] BEt_3 (1 M in hexane, 1.5 eq.); [c] $ZnEt_2$ (1 M in hexane) used as reducing agent instead of BEt_3; [d] 20 mol% catalyst loading; [e] BEt_3 (1 M in toluene, 3 eq.) used

We observed a significant improvement in terms of regioselectivity and enantioselectivity when changing the solvent from THF to toluene (compare entry 1 and 2), but the reaction initially suffered from incomplete conversion. The use of $ZnEt_2$ as the ultimate reducing agent shut the reaction down,

despite its track record in nickel-catalyzed reductive couplings of aldehydes and dienes.[8] Addition of a higher excess of diene (entry 5) could partly address the conversion issue; even when using an increased catalyst loading (entry 6) the conversion remained incomplete. The use of an increased amount of BEt_3 in hexane caused precipitation of the diene and hence affected the reaction. Therefore, BEt_3 in toluene was used, which led to quantitative conversion (entry 7).

General Procedures

General Procedure A (For Aromatic Aldehydes). A flame-dried Schlenk flask was charged with tris(*trans*-1,2-bis(4-*tert*-butylphenyl)ethene)nickel(0) (Ni(^{t}Bu-stb)$_3$, **16**) (18.7 mg, 0.02 mmol), phosphoramidite **L1** (12.8 mg, 0.02 mmol) and the respective diene (0.40 mmol or 0.60 mmol). The Schlenk flask was evacuated and refilled with argon (3 cycles). THF (0.35 mL) and triethylborane (1 M in THF, 0.30 mL, 0.30 mmol) were added and the resulting solution was stirred for 5 min. The respective aldehyde (0.20 mmol) was added under an argon counterflow, the flask was sealed and the mixture stirred at room temperature for the indicated time. The reaction was quenched *via* the indicated work-up procedure **I** or **II**. Purification of the crude material by flash chromatography furnished the analytically pure *vic*-aminoalcohol product.

General Procedure B (For Aliphatic Aldehydes). A flame-dried Schlenk flask was charged with tris(*trans*-1,2-bis(4-*tert*-butylphenyl)ethene)nickel(0) (Ni(^{t}Bu-stb)$_3$, **16**) (18.7 mg, 0.02 mmol), phosphoramidite **L1** (12.8 mg, 0.02 mmol) and the respective diene (0.40 mmol). The Schlenk flask was evacuated and refilled with argon (3 cycles). Toluene (1.4 mL) and triethylborane (1 M in toluene, 0.60 mL, 0.60 mmol) were added and the resulting solution was stirred for 5 min. The respective aldehyde (0.20 mmol) was added under an argon counterflow, the flask was sealed and the mixture stirred at room temperature for 72 h. The reaction was quenched *via* the indicated work-up procedure **I** or **II**. Purification of the crude material by flash chromatography furnished the analytically pure *vic*-aminoalcohol product.

Work-up Procedures

The product initially formed is the corresponding -$OBEt_2$ adduct. The O-B bond is fairly stable towards hydrolysis but can be cleaved under oxidative or basic conditions as described below to give the desired free alcohol. In general, the **oxidative work-up (I)** is preferred, except when functionality sensitive to oxidation is present (e. g. the pinacolboronate in product **29**).

The **basic work-up (II)** tends to be less efficient and an extended period of stirring is required to achieve complete cleavage of the O-B bond. It is recommended only for substrates that are sensitive to oxidants.

Oxidative Work-up Procedure (I): The reaction mixture was cooled to 0 °C and pH 7 phosphate buffer solution (1.5 mL) and aq. H_2O_2 (30% *w/w* in water, ≈0.3 mL) were added. The mixture was diluted with ethyl acetate (2 mL) and vigorously stirred for 1 h at 0 °C. Additional ethyl acetate (10 mL) and water (5 mL) were added, the organic phase was separated, and the aqueous layer was extracted with ethyl acetate (2 × 10 mL). The combined organic layers were washed with sat. aq. $Na_2S_2O_3$ (15 mL) and brine (15 mL), dried over $MgSO_4$, filtered and concentrated *in vacuo*.

Basic Work-up Procedure (II): Sat. aq. $NaHCO_3$ (3 mL) and ethyl acetate (2 mL) were added to the reaction mixture and vigorous stirring continued for 3 h at room temperature. The mixture was diluted with ethyl acetate (10 mL) and water (5 mL) and the aqueous phase was extracted with ethyl acetate (2 × 10 mL). The combined organic layers were washed with brine (15 mL), dried over $MgSO_4$, filtered and concentrated *in vacuo*.

Notes

1. THF and toluene were distilled under argon and stored in Schlenk flasks under argon for long-term use. Karl-Fischer-titration was used to check that the water content did not exceed 20 ppm.
2. The presence of residual chlorinated solvents ($CHCl_3$, CH_2Cl_2 etc.) in any of the reaction partners can lead to catalyst deactivation.
3. Liquid aldehydes were distilled under vacuum and stored in the freezer (–18 °C) under argon. Carboxylic acid impurities have a deleterious effect; therefore the purity of the aldehyde was checked by ^{1}H NMR spectroscopy prior to use.
4. Solid aldehydes were purified by recrystallization prior to use.
5. Triethylborane solutions in THF and toluene were prepared from neat triethylborane and the respective solvent. ***Note***: *Triethylborane is pyrophoric and must be handled carefully under rigorously oxygen-free and water-free conditions*.
6. The diene-carbamates showed good stability and were handled and weighed in air. However, partial decomposition was observed when kept in air for extended periods of time (> 3 days). When stored in Schlenk flasks under argon in the freezer (–18 °C), they could be used over extended periods of time (> 6 months) without any notable decomposition.
7. Tris(*trans*-1,2-bis(4-*tert*-butylphenyl)ethene)nickel(0) (**16**) can be weighed in air, yet is stored in the freezer at –18 °C. The same batch was used for > 1 year without any observable loss of catalytic activity.
8. Although tris(*trans*-1,2-bis(4-*tert*-butylphenyl)ethene)nickel(0) (**16**) is air-stable in the solid state, care must be taken once it has been dissolved. Therefore, the ligand was always added *before* the solvent as it helps stabilize the Ni(0) complex should residual oxygen be present in

solution. Likewise, the diene seems to stabilize the nickel species, while triethylborane quenches any remaining oxygen.

9. Small-scale reactions were carried out in sealed Schlenk flasks. However, ethylene gas is released from reduction of the nickel catalyst by triethylborane and care must be taken to use reaction vessels that can withstand the increase in pressure caused by this gas evolution. ***Note***: *For larger scale reactions, it is essential to use a gas bubbler to prevent build-up of overpressure and possible explosion of the flask*.
10. If the reactions are performed without the addition of any ligand, the mixture immediately turns black, likely because of the formation of nickel black / nickel nanoparticles. The reaction of an aldehyde with dienecarbamate **13** then proceeds exclusively at the π-bond proximal to the ester to give the racemic aldol-type product such as *rac*-**14**.
11. In the presence of the VaPhos-NEt_2 ligand **L1**, the color of the reaction mixture is usually intense orange to yellow. Formation of a dark color or black particles indicates catalyst decomposition and will cause a drop in conversion, regioselectivity and yield presumably due to a competing background reaction.
12. The regioisomeric ratio was determined by ^{1}H NMR analysis of the crude materal after the aqueous workup. Unless stated otherwise, the dr was > 20:1.
13. The use of toluene/*tert*-butyl methyl ether mixtures as the eluent for flash chromatography allowed any regioisomers to be separated from the desired *vic*-aminoalcohol products.
14. Recrystallization was performed to increase the ee of the products. On small scale, the product was simply dissolved by portionwise addition of a solvent and ultrasonication of the sample. An anti-solvent was then added and the vial stored in the freezer at −18 °C until the product had crystallized (at least 1 day).
    For experiments on larger scale, the product was recrystallized by allowing a hot solution of the *vic*-aminoalcohol derivative to slowly cool to room temperature (see picture below, recrystallization of ca. 900 mg of compound **15a**).

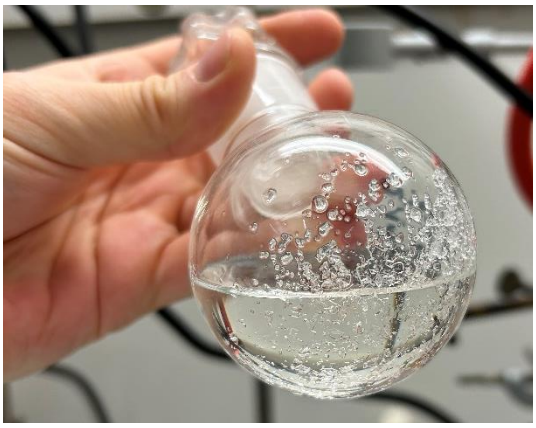
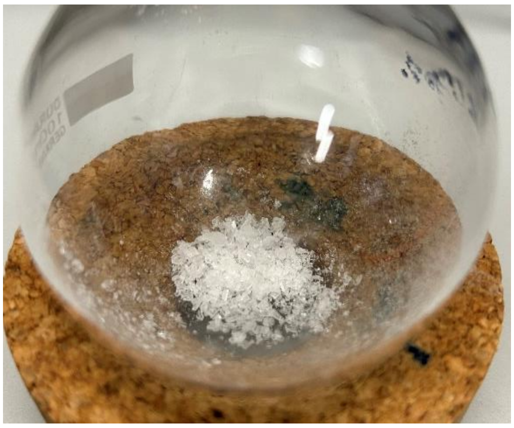

Figure S7. Recrystallization of compound **15a** on 900 mg scale.

Control Experiments

1) VANOL-derived phosphoramidite ligands had shown inferior reactivity and selectivity when compared to their VAPOL analogues in our initial ligand screening on nickel-catalyzed reductive couplings between aldehydes and dienol ethers.[4] However, other applications are known in which di-substituted VANOL-derivatives bearing *tert*-butyl groups in the 7,7'-position surpass VAPOL in terms of enantioselectivity.[9] Inspired by this precedent, the corresponding phosphoramidite ligand **L2** was tested in the reductive coupling between benzaldehyde and **13a**.

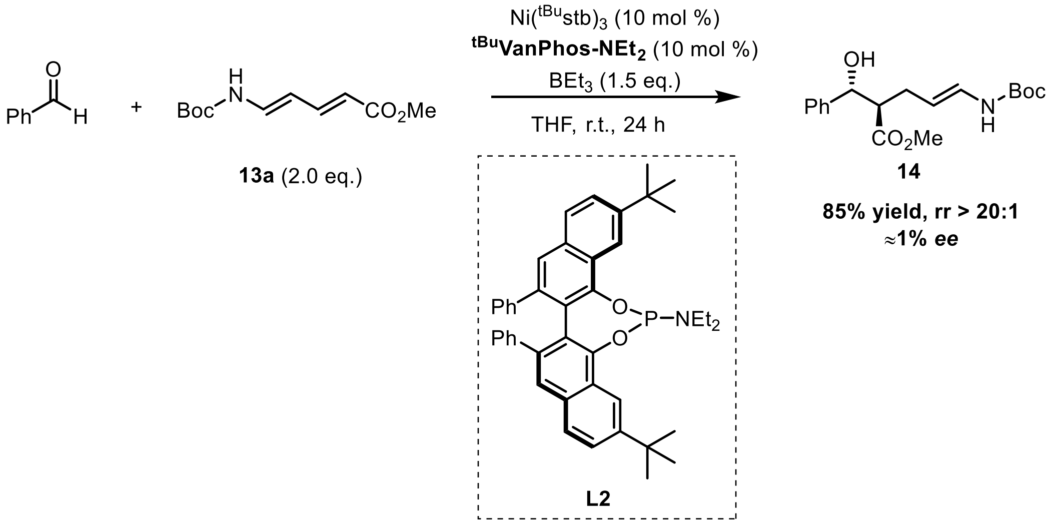

Scheme S1. The phosphoramidite ligand **L2** derived from a 7,7'-disubstituted VANOL derivative was found incapable of reverting the course of the addition reaction and did not impose any enantiocontrol on the C-C bond formation either.

The undesired regioisomer **14** was formed almost exclusively and in virtually racemic form; only traces of the aminoalcohol derivative **15a** were observed in the crude NMR (Scheme S1).

2) In the original publication on the nickel catalyzed asymmetric inverse reductive coupling chemistry, a set of ≈ 50 different ligands was screened, of which only the VaPhos derivative **L1** was able to effect the reaction with high regio- and enantioselectivity.[4] To further test if this unique qualification also pertains to the formation of the *vic*-aminoalcohol derivatives reported in the present publication, three representative examples of the previously unsuccessful ligands featuring a Binol-, Spinol- or Taddol-ligand framework were screened. Whereas **L3** and **L4** furnished the Mori-Tamaru product **14** almost exclusively rather than the

targeted *vic*-aminoalcohol **15a**, the Taddol-derived ligand **L5** gave **15a** predominantly but with very poor ee. The VaPhos derived ligand **L1** is clearly superior and was therefore used throughout this investigation.

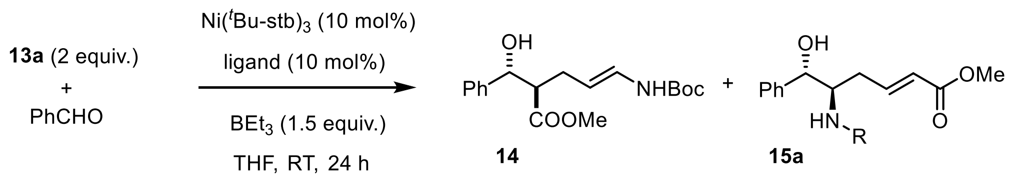

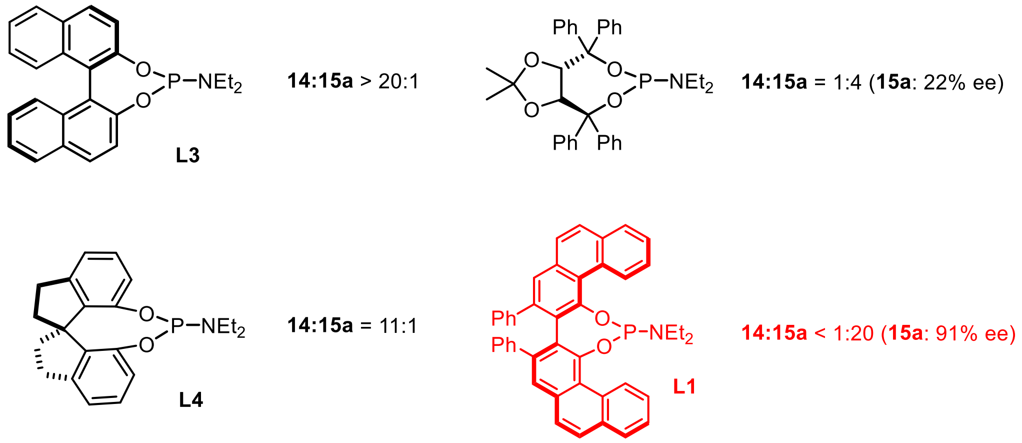

3) Performing the reductive coupling in the presence of 3.0 eq. of degassed water drastically changed the outcome of the reaction.

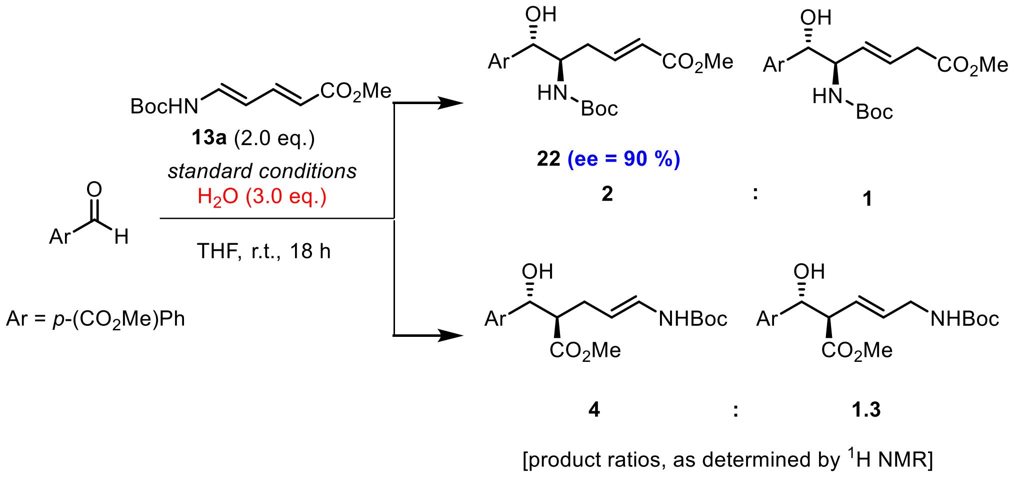

Scheme S2. Water has a deleterious effect on the product distribution

Apart from a loss of regioselectivity, partial isomerization of the double bond out of conjugation was observed for both the *vic*-aminoalcohol product and its regioisomer (Scheme S2). It is of note, however, that the ee of the *vic*-aminoalcohol product **22** present in the mixture remained basically unchanged. In the absence of water, no signs of double bond isomerization were detected.

4) In addition to Ni(^{t}Bu-stb)$_3$ (**16**), Ni(cod)$_2$ can be used as a precatalyst. While there appears to be no significant effect on enantioselectivity, Ni(cod)$_2$ led to a lower regioselectivity and hence a lower isolated yield of product **22** (Scheme S3).

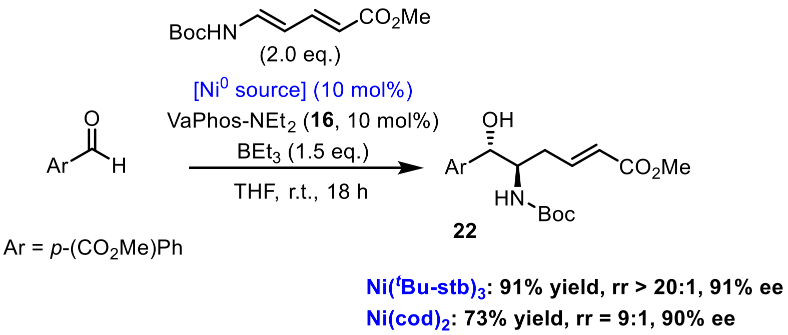

Scheme S3. Comparison of two different Ni(0) precatalysts

5) A control experiment showing the need to use "push/pull" dienes.

***tert*-Butyl ((1*S*,2*R*)-1-hydroxy-1-phenylpent-4-en-2-yl)carbamate (9).** A flame dried Schlenk flask under argon was charged with $Ni(cod)_2$ (8.0 mg, 0.03 mmol), phosphoramidite ligand **L1** (18.6 mg, 0.03 mmol), and *tert*-butyl (*E*)-buta-1,3-dien-1-yl carbamate (63.9 mg, 0.38 mmol).[10] The Schlenk flask was evacuated and refilled with argon (3 cycles). THF (0.60 mL), triethylborane (1 M in THF, 0.44 mL, 0.44 mmol) and benzaldehyde (29.6 µL, 0.29 mmol) were added under argon counterflow, the flask was sealed and the mixture was stirred at room temperature for 24 h. The reaction was quenched by addition of sat. aq. $NaHCO_3$ solution (3 mL) and the resulting mixture was stirred for 1 h. The mixture was diluted with ethyl acetate (3 mL) and the organic layer extracted with ethyl acetate (3 x 3 mL). The combined extracts were dried over $MgSO_4$, filtered, and the solvent was removed under reduced pressure. The crude product was purified by flash chromatography (SiO_2, *iso*-hexane/ethyl acetate, 20:1 → 4:1) to give the title compound as an off-white solid (37.0 mg, 46% yield, > 20:1 dr, 55% ee). mp = 73-75 °C, ^1H NMR (400 MHz, $CDCl_3$): δ = 7.38 – 7.27 (m, 5H), 5.79 – 5.69 (m, 1H), 5.09 – 5.02 (m, 2H), 4.90 (s, 1H), 4.54 (br, 1H), 3.96 (br, 1H), 3.36 (s, 1H), 2.24 – 2.17 (m, 1H), 2.10 – 2.02 (m, 1H), 1.44 (s, 9H); ^{13}C NMR (151 MHz, $CDCl_3$): δ = 156.9, 140.8, 134.8, 128.3 (2C), 127.7, 126.6 (2C), 117.9, 80.0, 76.4, 56.1, 33.9, 28.5 (3C); IR (ATR): ṽ = 3417, 2978, 1687, 1503, 1366, 1251, 1168, 1023, 702 cm^{-1}; HRMS (ESI$^+$): m/z: calcd. for $C_{16}H_{23}NO_3Na$ $[M+Na]^+$: 300.15701, found: 300.15705.

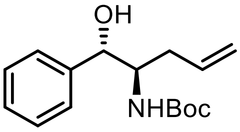

The ee was determined by 2D-HPLC analysis. First dimension = 100 mm Zorbax RX-SIL, 4.6 mm Ø, *n*-heptane/*iso*-propanol = 99:1, v = 1.0 mL/min, λ = 220 nm. Second Dimension = 150 mm Chiralcel OD-3, 4.6 mm Ø, *n*-heptane/*iso*-propanol = 90:10, v = 1.0 ml/min, λ = 220 nm.

First dimension racemic sample:

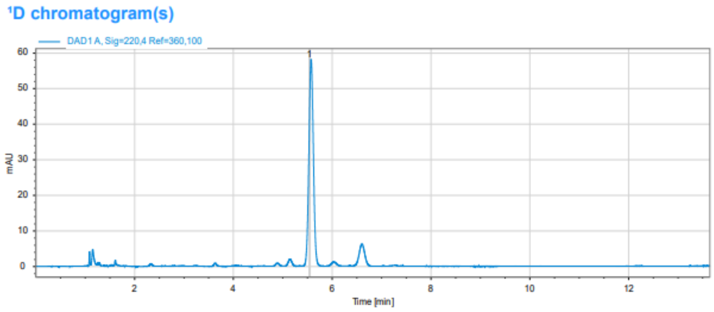

Sampling table (¹D)

Cut group	Cut #	¹D Cut start [min]	¹D Ret. time [min]	¹D Duration [min]	Trigger	²D Run start [min]
	1	5.53	***	0.04	Peak	5.58

Second dimension racemic sample:

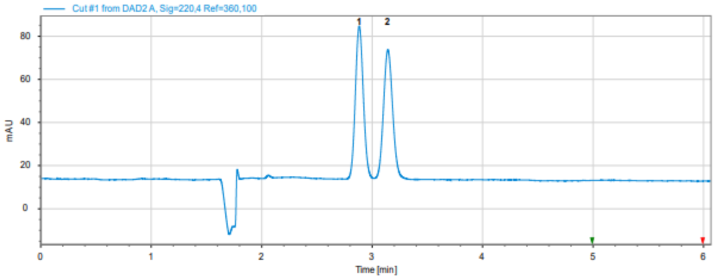

Signal: DAD2 A, Sig=220,4 Ref=360,100

Component table

Signal: DAD2 A, Sig=220,4 Ref=360,100

Component	¹D Sampling range [min]	Ret.Time ²D [min]	Area	Area%
1	5.53 - 5.57	2.883	357.898	50.715 1. enantiomer
2	5.53 - 5.57	3.142	347.806	49.285 2. enantiomer

First dimension enantioenriched sample:

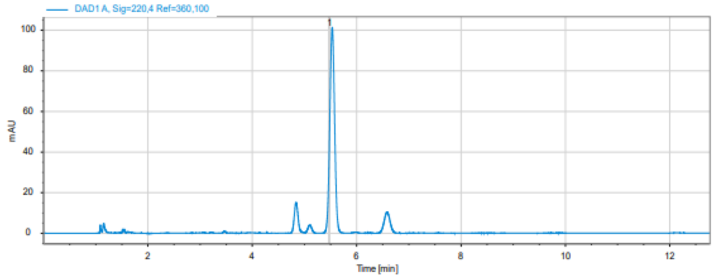

Sampling table (¹D)

Cut group	Cut #	¹D Cut start [min]	¹D Ret. time [min]	¹D Duration [min]	Trigger	²D Run start [min]
	1	5.47	***	0.04	Peak	5.52

Second dimension enantioenriched sample:

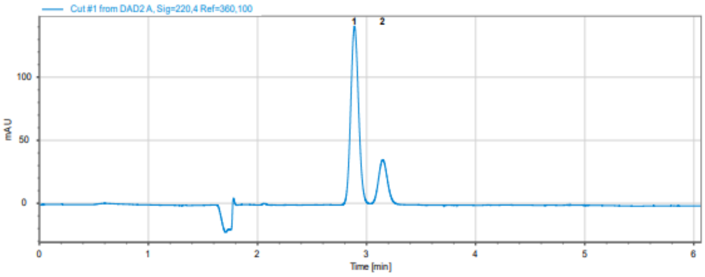

Signal: DAD2 A, Sig=220,4 Ref=360,100

Component table

Signal: DAD2 A, Sig=220,4 Ref=360,100

Component	¹D Sampling range [min]	Ret.Time ²D [min]	Area	Area%
1	5.47 - 5.51	2.889	712.401	77.262 1. enantiomer
2	5.47 - 5.51	3.148	209.658	22.738 2. enantiomer

ee = 54.5%

5) A control experiment under ligand-free conditions led exclusively to C–C coupling adjacent to the ester of diene **13a** with formation of *rac*-**14**.

Methyl (*E*)-5-((*tert*-butoxycarbonyl)amino)-2-(hydroxy(phenyl)methyl)pent-4-enoate (*rac*-14). A flame-dried Schlenk flask under argon was charged with tris(*trans*-1,2-bis(4-*tert*-butylphenyl)ethene)nickel(0) (Ni(^{t}Bu-stb)$_3$, **16**) (18.7 mg, 0.02 mmol) and diene **13a** (90.9 mg, 0.40 mmol). The Schlenk flask was evacuated and refilled with argon (3 cycles). THF (0.35 mL) and triethylborane (1 M in THF, 0.30 mL, 0.30 mmol) were added and the resulting solution was stirred for 5 min. Benzaldehyde (20.2 µL, 0.20 mmol) was added under an argon counterflow, the flask was sealed and the mixture stirred at room temperature for 16 h. The reaction was quenched at 0 °C by addition of pH 7 phosphate buffer solution (1.5 mL) and aq. H_2O_2 (30% *w/w* in water, 0.3 mL). Ethyl acetate (2.0 mL) was added and the resulting mixture was vigorously stirred for 1 h. The mixture was diluted with ethyl acetate (10 mL) and water (5 mL) and the aqueous phase was extracted with ethyl acetate (2 x 10 mL). The combined organic layers were washed with sat. aq. $Na_2S_2O_3$ solution (15 mL) and brine (15 mL), dried over $MgSO_4$, filtered, and the solvent was removed under reduced pressure. The residue was purified by flash chromatography (SiO_2, *iso*-hexane + 1% NEt_3 →*iso*-hexane/ethyl acetate 4:1 + 1% NEt_3) to give the title compound as a colorless oil (63.8 mg, 95%). ^1H NMR (400 MHz, CD_2Cl_2): δ = 7.36 – 7.25 (m, 5H), 6.40 (t, *J* = 12.4 Hz, 1H), 6.25 (br, 1H), 4.94 (dd, *J* = 6.1, 2.3 Hz, 1H), 4.89 – 4.81 (m, 1H), 3.55 (s, 3H), 2.85 (s, 1H), 2.77 – 2.72 (m, 1H), 2.43 – 2.27 (m, 2H), 1.43 (s, 9H); ^{13}C NMR (101 MHz, CD_2Cl_2): δ = 174.6, 152.9, 142.2, 128.7 (2C), 128.1, 126.6 (2C), 126.0, 105.9, 80.5, 74.1, 54.3, 51.9, 28.4 (3C), 28.2; IR (ATR): ṽ = 3336, 2978, 1701, 1676, 1516, 1367, 1246, 1163, 1019, 702 cm^{-1}; HRMS (ESI$^+$): *m/z*: calcd. for $C_{18}H_{25}NO_5Na$ $[M+Na]^+$: 358.16249, found: 358.16249.

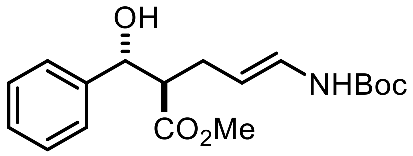

Determination of the Regioisomer Ratio (rr)

The regioisomer ratios (rr) were determined by analysis of the ^{1}H NMR spectra of the crude products after the aqueous workup. Figure S8 shows the ^{1}H NMR spectrum of the crude mixture of the reductive coupling of benzaldehyde with diene **13a** catalyzed by [Ni(*t*Bu-stb)$_3$]/**L1** as well as the ^{1}H NMR spectra of the purified aminoalcohol derivative **15a** and its regioisomer *rac*-**14**. As evident from Figure S9, the ester groups of **15a** and *rac*-**14** show significant shift differences and hence allow for accurate determination of the rr; this effect is fairly general, see Figures S10 and S11. Likewise, the olefinic protons of the regioisomers are usually well separated (see Figure S12) and therefore also suitable for the determination of the rr.

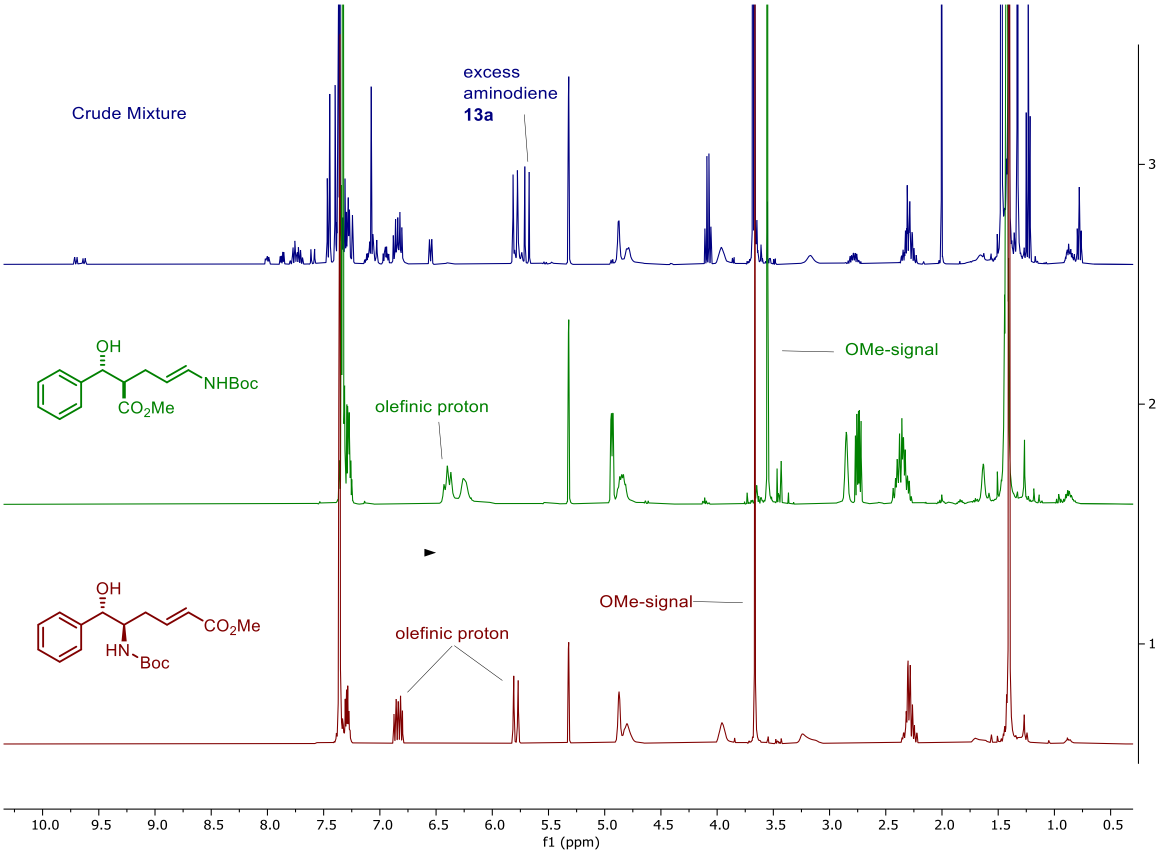

Figure S8. Stacked spectra of the crude mixture of the ligand-controlled reductive coupling of aminodiene **13a** and benzalydehyde (blue), the Tamaru/Mori isomer *rac*-**14** (green), and the *vic*-aminoalcohol derivative **15a** (red).

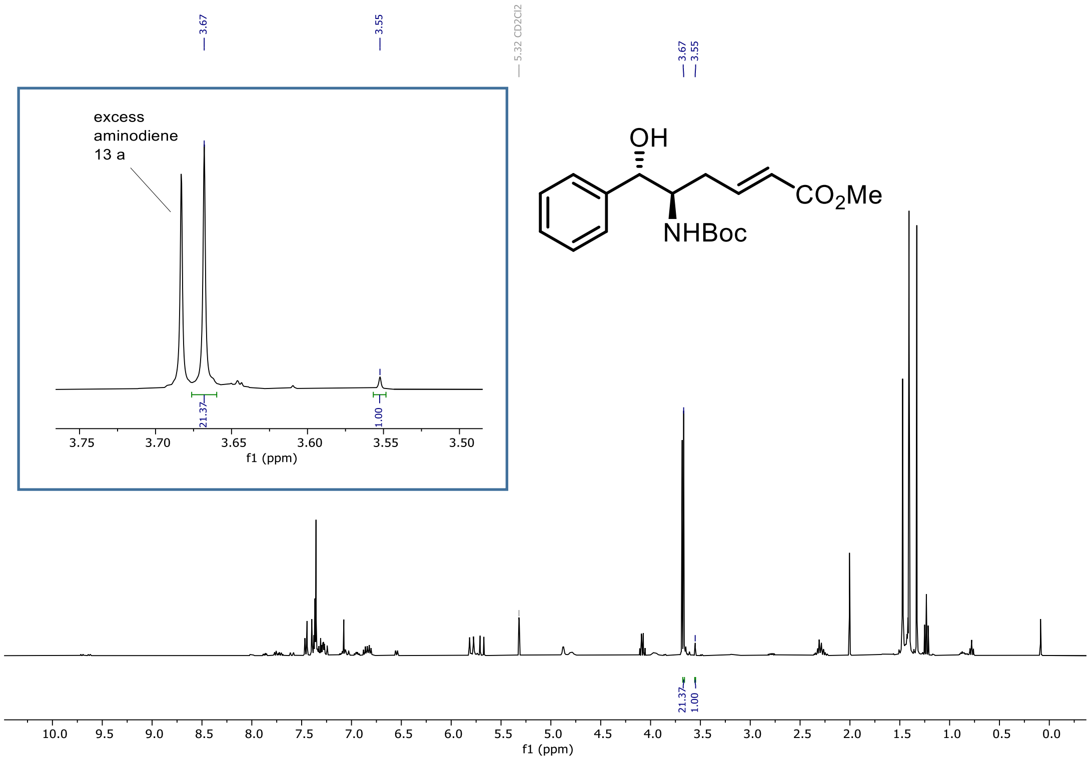

Figure S9. Determination of the rr from the ^{1}H NMR of the crude product of aminoalcohol derivative **15a**; as can be seen from the Insert, the rr was > 20:1

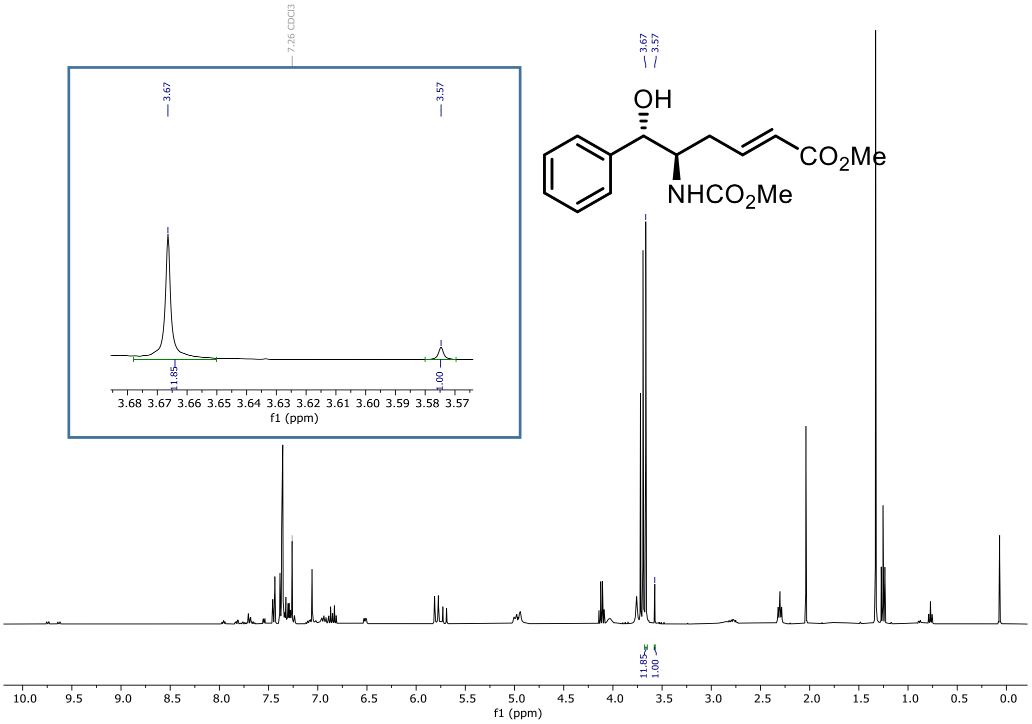

Figure S10. Determination of the rr from the ^{1}H NMR of the crude product of *vic*-aminoalcohol derivative **15b**; as can be seen from the Insert, the rr was ≈ 12:1

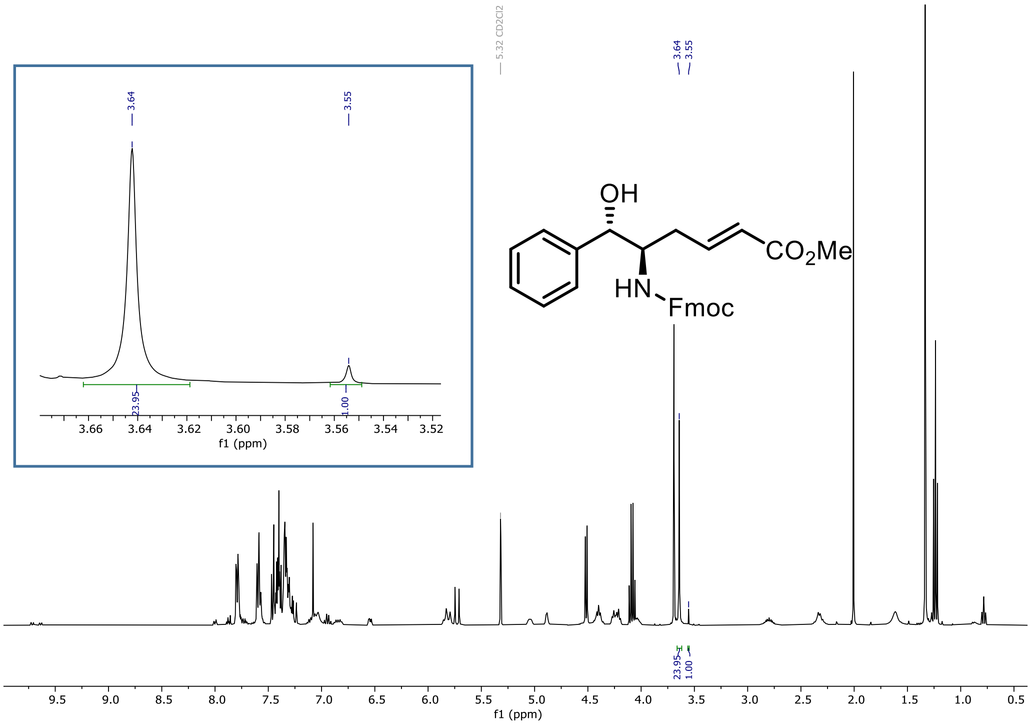

Figure S11. Determination of the rr from the ^{1}H NMR of the crude product of *vic*-aminoalcohol derivative **15d**; as can be seen from the Insert, the rr was > 20:1

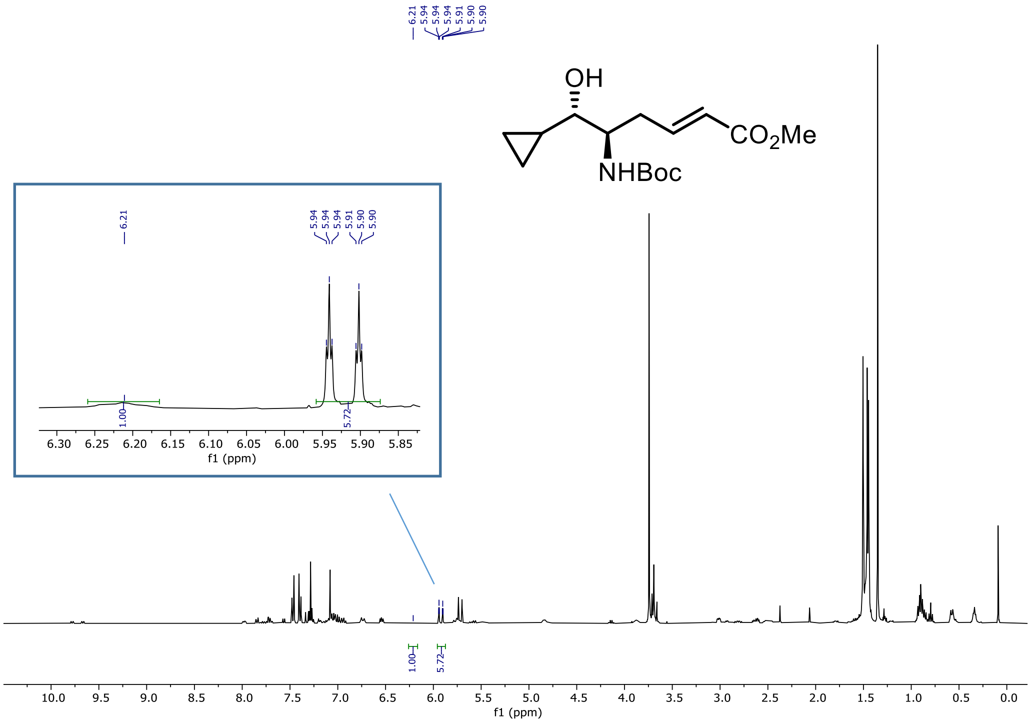

Figure S12. Determination of the rr from the ^{1}H NMR of the crude product of *vic*-aminoalcohol derivative **39** using the olefinic signals of the isomers; as can be seen from the Insert, the rr was ≈ 6:1

Characterization Data and ee-Determination

Methyl (5*R*,6*S*,*E*)-5-((*tert*-butoxycarbonyl)amino)-6-hydroxy-6-phenylhex-2-enoate (15a). The title compound was prepared according to the general procedure **A** and oxidative workup **I**, using benzaldehyde (20.3 µL, 0.20 mmol) and diene **13a** (90.9 mg, 0.40 mmol); the reaction time was 24 h. The crude material (rr > 20:1) was purified by flash chromatography (SiO_2, toluene/ *tert*-butyl methyl ether, 4:1) and the product was obtained as an off-white solid (61.0 mg, 91% yield, > 20:1 dr, 91% ee). mp = 112-113 °C; $[\alpha]_D^{20}$ (99% ee, sample after recrystallization) = +14.0 (c = 0.50, $CHCl_3$); ^1H NMR (400 MHz, CD_2Cl_2): δ = 7.36 – 7.26 (m, 5H), 6.87 – 6.80 (m, 1H), 5.79 (dt, *J* = 15.6, 1.4 Hz, 1H), 4.87 (s, 1H), 4.80 (s, 1H), 3.95 (s, 1H), 3.66 (s, 3H), 3.24 (br, 1H), 2.36 – 2.22 (m, 2H), 1.40 (s, 9H); ^{13}C NMR (101 MHz, CD_2Cl_2): δ = 166.8, 156.5, 145.9, 141.4, 128.7 (2C), 128.1, 126.6 (2C), 123.5, 80.1, 76.4, 55.9, 51.7, 32.4, 28.4 (3C); IR (ATR): ṽ = 3453, 3358, 1689, 1651, 1525, 1323, 1228, 1165, 1016, 701 cm^{-1}; HRMS (ESI^+): *m/z*: calcd. for $C_{18}H_{25}NO_5Na$ $[M+Na]^+$: 358.16249, found: 358.16239.

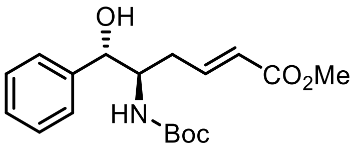

34.0 mg of the product were recrystallized from *tert*-butyl methyl ether/*iso*-hexane to give the enantioenriched product in 99% ee (28.6 mg).

The ee was determined by HPLC analysis: Chiralpak 150 mm IB-N-3, 3 µm, 4.6 mm ∅, *n*-heptane/*iso*-propanol = 95:5, v = 1.0 mL/min, λ = 220 nm, t(major) = 9.15 min, t(minor) = 10.68 min (racemate: top left, enantioenriched sample: top right, recrystallized enantioenriched sample: bottom left).

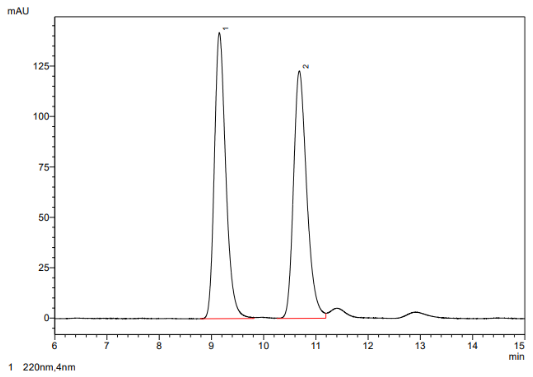

PDA Ch1 220nm

Peak #	Ret. Time	Area %	Name
1	9,15	50,03	1. Enantiomer
2	10,68	49,97	2. Enantiomer
Total		100,00	

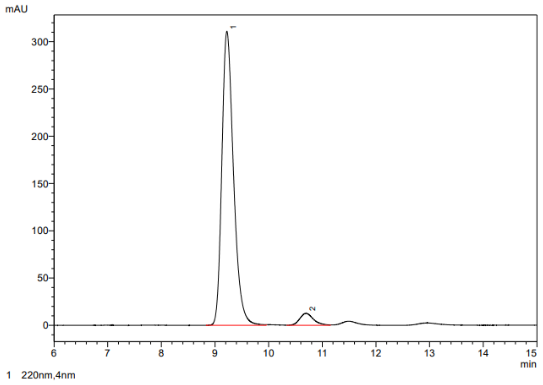

PDA Ch1 220nm

Peak #	Ret. Time	Area %	Name
1	9,22	95,40	1. Enantiomer 90.8 % ee
2	10,69	4,60	2. Enantiomer
Total		100,00	

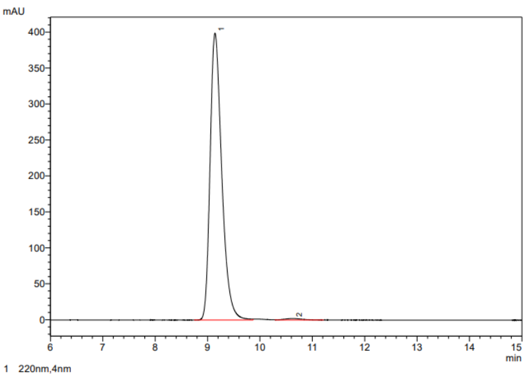

PDA Ch1 220nm

Peak #	Ret. Time	Area %	Name
1	9,14	99,24	1. Enantiomer 98.5 % ee
2	10,63	0,76	2. Enantiomer
Total		100,00	

Gram-Scale Reaction. A flame-dried Schlenk flask under argon was charged with tris(*trans*-1,2-bis(4-*tert*-butylphenyl)ethene)nickel(0) (Ni(^{t}Bu-stb)$_3$) (**16**, 318 mg, 0.34 mmol), phosphoramidite **L1** (217.5 mg, 0.34 mmol) and diene **13a** (1.55 g, 6.80 mmol). The Schlenk flask was evacuated and refilled with argon (3 cycles). THF (6.0 mL) and triethylborane (1 M in THF, 5.10 mL, 5.10 mmol) were added

and the resulting red solution was stirred for 5 minutes. Benzaldehyde (344 µL, 3.40 mmol) was added under an argon counterflow, causing a color change of the mixture to intense orange. After addition of the aldehyde, a gas bubbler was connected to the Schlenk flask and the mixture was stirred under a very gentle flow of argon at room temperature for 24 h. At this point, TLC indicated full conversion of the aldehyde. The mixture was cooled to 0 °C and the reaction was quenched by addition of pH 7 phosphate buffer solution (10 mL) and aq. H_2O_2 (30% *w/w* in water, 4 mL). Ethyl acetate (25 mL) was added and the resulting mixture was vigorously stirred for 1 h. The organic layer was separated and the aqueous phase was extracted with ethyl acetate (3 x 40 mL). The combined organic layers were washed with sat. aq. $Na_2S_2O_3$ (25 mL) and brine (25 mL), dried over $MgSO_4$, filtered, and the solvent was removed under reduced pressure. The residue (rr = 15:1) was purified by flash chromatography (SiO_2, toluene/ *tert*-butyl methyl ether, 4:1) to give product **15a** as an off-white solid (1.01 g, 88%, dr > 20:1, 90% ee). For the analytical and spectral data, see above.

A second fraction contained unreacted diene **13a** partly contaminated by the ligand. When this material was subjected to a second flash chromatographic purification using a different eluent (SiO_2, *iso*-hexane/ethyl acetate, 4:1), pure diene **13a** was recovered as a colorless solid (589 mg, 0.76 eq.).

900 mg of **15a** were recrystallized from *tert*-butyl methyl ether/*iso*-hexane to give the enantioenriched product in 99% ee (707 mg), see Figure S7.

Note: Single crystals suitable for X-ray diffraction analysis were obtained in the process of recrystallization and were used to determine the relative and absolute configuration of the *vic*-aminoalcohol product **15a**, see Figure S1.

The ee was determined by HPLC analysis: Chiralpak 150 mm IB-N-3, 3 µm, 4.6 mm ∅, *n*-heptane/*iso*-propanol = 95:5, v = 1.0 mL/min, λ = 220 nm, t(major) = 9.15 min, t(minor) = 10.68 min (racemate: top left, enantioenriched sample: top right, recrystallized enantioenriched sample: bottom left).

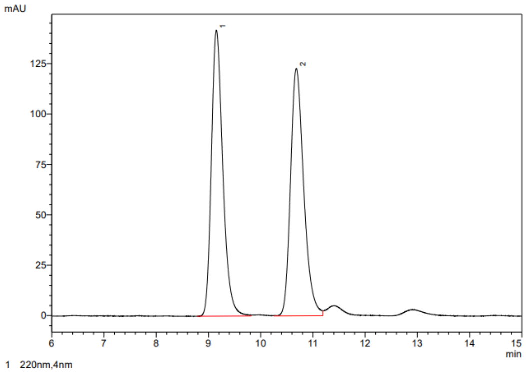

PDA Ch1 220nm

Peak #	Ret. Time	Area %	Name
1	9,15	50,03	1. Enantiomer
2	10,68	49,97	2. Enantiomer
Total		100,00	

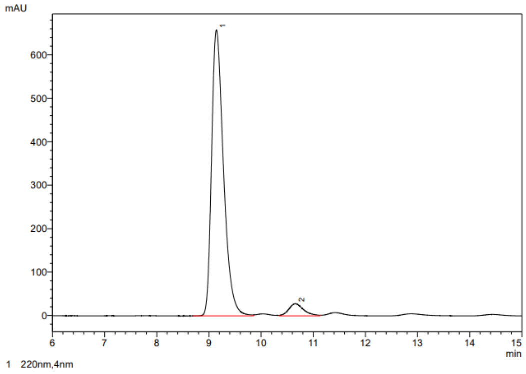

PDA Ch1 220nm

Peak #	Ret. Time	Area %	Name
1	9,14	94,93	1. Enantiomer 89.9 % ee
2	10,66	5,07	2. Enantiomer
Total		100,00	

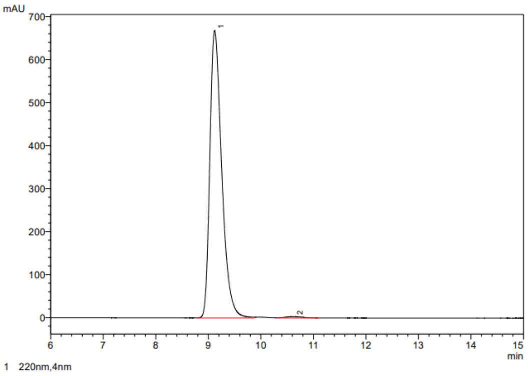

PDA Ch1 220nm

Peak #	Ret. Time	Area %	Name
1	9,12	99,37	1. Enantiomer 98.7 % ee
2	10,62	0,63	2. Enantiomer
Total		100,00	

Methyl (5*R*,6*S*,*E*)-6-hydroxy-5-((methoxycarbonyl)amino)-6-phenylhex-2-enoate (15b). The title compound was prepared according to the general procedure **A** and oxidative workup **I**, using benzaldehyde (20.3 µL, 0.20 mmol) and diene **13b** (74.1 mg, 0.40 mmol); the reaction time was 24 h. The crude material (rr = 12:1) was purified by flash chromatography (SiO_2, toluene/*tert*-butyl methyl ether, 1.5:1) and the product was obtained as an off-white solid (50.5 mg, 86% yield, > 20:1 dr, 93% ee). $[\alpha]_D^{20}$ (> 99% ee, sample after recrystallization) = +7.6 (c = 0.50, $CHCl_3$); ^1H NMR (400 MHz, CD_2Cl_2): δ = 7.47 – 7.22 (m, 5H), 6.82 (ddd, *J* = 15.6, 7.6, 6.9 Hz, 1H), 5.79 (dt, *J* = 15.6, 1.5 Hz, 1H), 5.06 (d, *J* = 9.1 Hz, 1H), 4.90 (s, 1H), 3.99 (s, 1H), 3.66 (s, 3H), 3.62 (s, 3H), 3.01 (s, 1H), 2.56 – 2.14 (m, 2H); ^{13}C NMR (101 MHz, CD_2Cl_2): δ = 166.8, 157.4, 145.8, 141.2, 128.8 (2C), 128.1, 126.5 (2C), 123.6, 76.0, 56.3, 52.6, 51.7, 32.0; IR (ATR): ṽ = 3496, 3331, 1705, 1687, 1544, 1319, 1229, 1042, 706, 521 cm^{-1}; HRMS (CI): *m/z*: calcd. for $C_{15}H_{20}NO_5$ $[M+H]^+$: 294.13360, found: 294.13363.

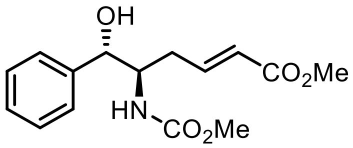

19.0 mg of the product were recrystallized from *tert*-butyl methyl ether/*iso*-hexane to give the enantioenriched product in >99% ee (14.6 mg), mp = 101-102 °C.

Note: Single crystals suitable for X-ray diffraction analysis were obtained in the process of recrystallization and were used to determine the relative and absolute configuration of the vic-aminoalcohol product **15b** (see Figure S3).

The ee was determined by HPLC analysis: Chiralcel 150 mm IG-3, 3 µm, 4.6 mm ∅, CH_3CN/water = 20:80 → CH_3CN/water = 50:50 (over 10 minutes), v = 1.0 mL/min, λ = 220 nm, t(minor) = 9.12 min, t(major) = 9.87 min (racemate: top left, enantioenriched sample: top right, recrystallized enantioenriched sample: bottom left).

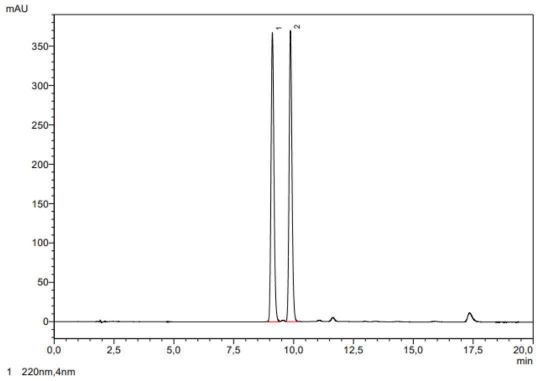

PDA Ch1 220nm

Peak #	Ret. Time	Area %	Name
1	9,12	49,98	1. Enantiomer
2	9,87	50,02	2. Enantiomer
Total		100,00	

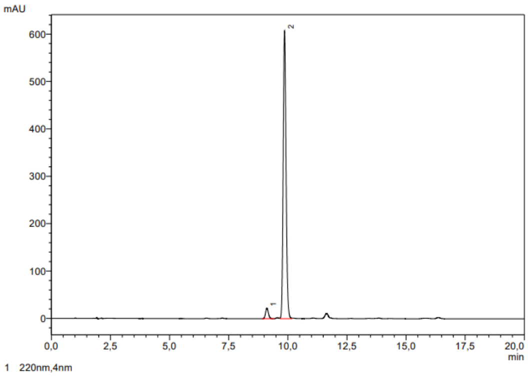

PDA Ch1 220nm

Peak #	Ret. Time	Area %	Name
1	9,12	3,55	1. Enantiomer
2	9,86	96,45	2. Enantiomer 92.9 % ee
Total		100,00	

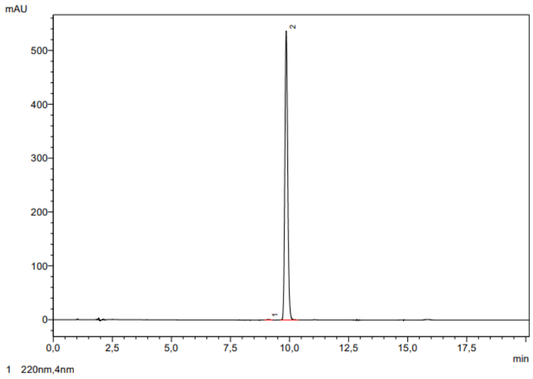

PDA Ch1 220nm

Peak #	Ret. Time	Area %	Name
1	9,11	0,17	1. Enantiomer
2	9,86	99,83	2. Enantiomer 99.7 % ee
Total		100,00	

Methyl (5*R*,6*S*,*E*)-5-(((benzyloxy)carbonyl)amino)-6-hydroxy-6-phenylhex-2-enoate (15c). The title compound was prepared according to the general procedure **A** and oxidative workup **I**, using benzaldehyde (20.3 µL, 0.20 mmol) and diene **13c** (104.5 mg, 0.40 mmol); the reaction time was 16 h. The crude material (rr = 14:1) was purified by flash chromatography (SiO_2, toluene/ *tert*-butyl methyl ether, 3:1) and the product was obtained as an off-white solid (61.0 mg, 83% yield, dr > 20:1, 89% ee). mp = 116-117 °C ; $[\alpha]_D^{20}$ (99% ee¸ sample after recrystallization) = +16.8 (c = 0.50, $CHCl_3$); ^1H NMR (400 MHz, CD_2Cl_2): δ = 7.38 – 7.27 (m, 10H), 6.88 – 6.80 (m, 1H), 5.80 (dt, *J* = 15.6, 1.5 Hz, 1H), 5.10 (br, 1H), 5.07 (s, 2H), 4.91 (s, 1H), 4.07 – 4.00 (m, 1H), 3.67 (s, 3H), 2.86 (s, 1H), 2.37 – 2.26 (m, 2H); ^{13}C NMR (101 MHz, CD_2Cl_2): δ = 166.8, 156.7, 145.8, 141.2, 137.1, 128.9 (2C), 128.8 (2C), 128.4, 128.2 (2C), 128.1, 126.6 (2C), 123.6, 76.0, 67.1, 56.3, 51.7, 32.1; IR (ATR): ṽ = 3354, 1694, 1653, 1537, 1439, 1321, 1228, 1024, 986, 695 cm^{-1}; HRMS (ESI$^+$): *m/z*: calcd. for $C_{21}H_{23}NO_5Na$ $[M+Na]^+$: 392.14684, found: 392.14717.

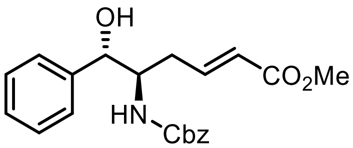

18.0 mg of the product were recrystallized from dichloromethane/*iso*-hexane to give the enantioenriched product with 99% ee (13.7 mg).

The ee was determined by HPLC analysis: Chiralpak 150 mm IG-3, 3 µm, 4.6 mm ∅, *n*-heptane/*iso*-propanol = 80:20; v = 1.0 mL/min, λ = 220 nm, t(minor) = 8.28 min, t(major) = 17.11 min (racemate: top left, enantioenriched sample: top right, recrystallized enantioenriched sample: bottom left).

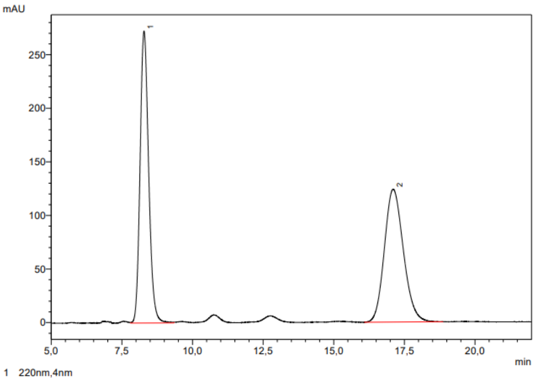

PDA Ch1 220nm

Peak #	Ret. Time	Area %	Name
1	8,28	49,77	1. Enantiomer
2	17,11	50,23	2. Enantiomer
Total		100,00	

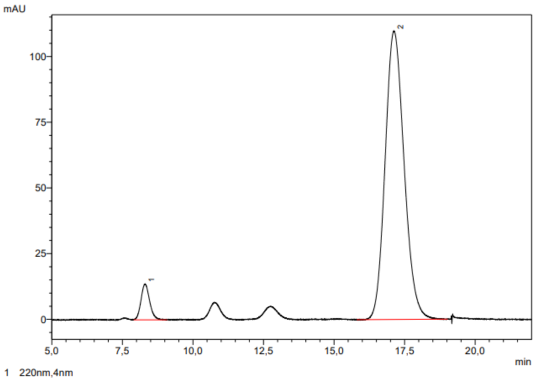

PDA Ch1 220nm

Peak #	Ret. Time	Area %	Name
1	8,30	5,36	1. Enantiomer
2	17,12	94,64	2. Enantiomer 89.3 % ee
Total		100,00	

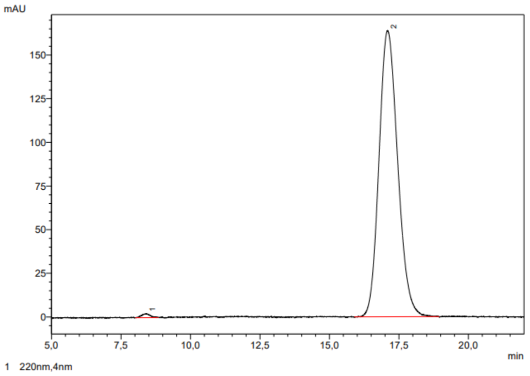

PDA Ch1 220nm

Peak #	Ret. Time	Area %	Name
1	8,40	0,62	1. Enantiomer
2	17,09	99,38	2. Enantiomer 98.8 % ee
Total		100,00	

Methyl (5*R*,6*S*,*E*)-5-((((9*H*-fluoren-9-yl)methoxy)carbonyl)amino)-6-hydroxy-6-phenylhex-2-enoate (15d).

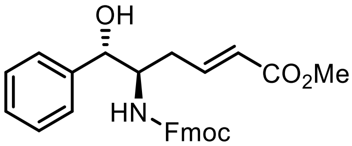

The title compound was prepared according to the general procedure **A** and oxidative workup **I**, using benzaldehyde (20.3 µL, 0.20 mmol) and diene **13d** (139.7 mg, 0.40 mmol). *Note: because of the low solubility of the diene, the volume of THF was increased to 1.2 mL.* The reaction time was 65 h. The crude material (rr > 20:1) was purified by flash chromatography (SiO_2, toluene/*tert*-butyl methyl ether, 3:1) and the product was obtained as an off-white solid (83.9 mg, 92% yield, dr > 20:1, 88% ee). mp = 157-158 °C; $[\alpha]_D^{20}$ (88% ee, sample before recrystallization) = +5.0 (c = 0.50, $CHCl_3$); ^{1}H NMR (600 MHz, CD_2Cl_2): δ = 7.79 (d, *J* = 7.5 Hz, 2H), 7.58 (d, *J* = 6.7 Hz, 2H), 7.44 – 7.39 (m, 2H), 7.39 – 7.26 (m, 7H), 6.84 (dt, *J* = 15.1, 7.2 Hz, 1H), 5.81 (d, *J* = 15.6 Hz, 1H), 4.99 (d, *J* = 9.1 Hz, 1H), 4.89 (s, 1H), 4.44 – 4.36 (m, 2H), 4.21 (t, *J* = 6.9 Hz, 1H), 4.05 – 4.02 (m, 1H), 3.64 (s, 3H), 2.71 (d, *J* = 3.7 Hz, 1H), 2.37 – 2.27 (m, 2H); ^{13}C NMR (151 MHz, CD_2Cl_2): δ = 166.7, 156.7, 145.5, 144.5, 144.3, 141.7, 141.7, 141.0, 128.8 (2C), 128.2, 128.1 (2C), 127.5 (2C), 126.6 (2C), 125.5, 125.4, 123.8, 120.3, 120.3, 76.1, 67.0, 56.3, 51.7, 47.7, 32.1; IR (ATR): ṽ = 3446, 1720, 1687, 1523, 1442, 1333, 1231, 1023, 988, 737 cm^{-1}; HRMS (ESI$^+$): *m/z*: calcd. for $C_{28}H_{28}NO_5Na$ [M+Na]$^+$: 458.19620, found: 458.19665.

20.0 mg of the product were recrystallized from dichloromethane/*iso*-hexane to give the enantioenriched product in 97% ee (14.6 mg).

The ee was determined by HPLC analysis: Chiralpak 150 mm IB-N-3, 3 µm, 4.6 mm ∅, CH_3CN/water = 70:30, v = 1.0 mL/min, λ = 220 nm, t(minor) = 6.20 min, t(major) = 8.84 min (racemate: top left, enantioenriched sample: top right, recrystallized enantioenriched sample: bottom left).

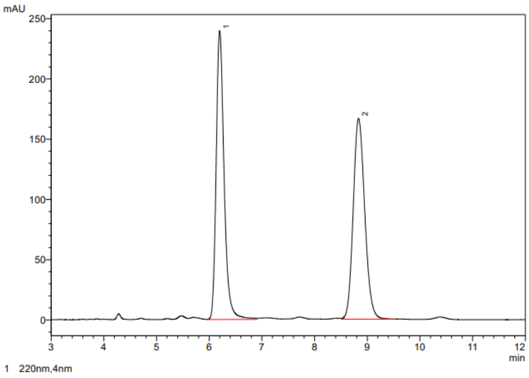

1 220nm,4nm

PDA Ch1 220nm

Peak #	Ret. Time	Area %	Name
1	6,20	49,73	1. Enantiomer
2	8,84	50,27	2. Enantiomer
Total		100,00	

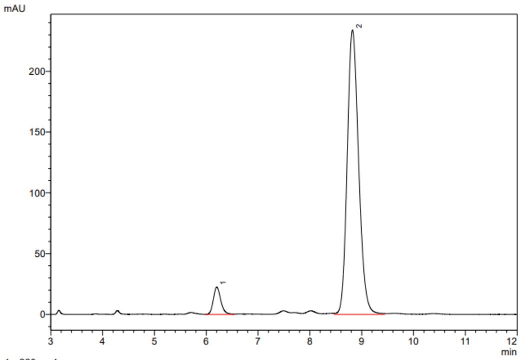

1 220nm,4nm

PDA Ch1 220nm

Peak #	Ret. Time	Area %	Name
1	6,20	5,88	1. Enantiomer
2	8,82	94,12	2. Enantiomer 88.2 % ee
Total		100,00	

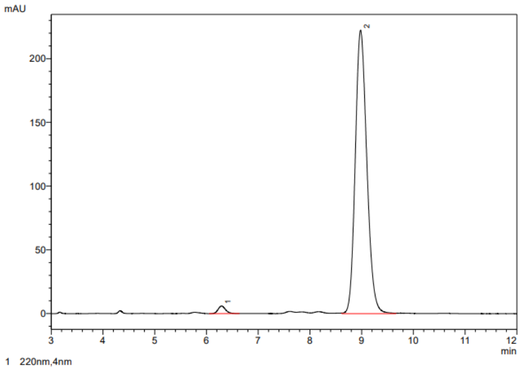

1 220nm,4nm

PDA Ch1 220nm

Peak #	Ret. Time	Area %	Name
1	6,29	1,70	1. Enantiomer
2	8,98	98,30	2. Enantiomer 96.6 % ee
Total		100,00	

Methyl (5*R*,6*S*,*E*)-5-((*tert*-butoxycarbonyl)(methyl)amino)-6-hydroxy-6-phenylhex-2-enoate (18a). A flame-dried Schlenk flask under argon was charged with tris(*trans*-1,2-bis(4-*tert*-butylphenyl)ethene)nickel(0) (Ni(^{t}Bu-stb)$_3$) (14.0 mg, 0.015 mmol) and phosphoramidite ligand **L1** (9.6 mg, 0.015 mmol). The Schlenk flask was evacuated and refilled with argon (3 cycles). A solution of diene **17a** (109 mg, 0.45 mmol) in THF (0.10 mL) and triethylborane (1 M in THF, 0.45 mL, 0.45 mmol) were successively added and the resulting solution was stirred for 5 min before benzaldehyde (15.2 µL, 0.15 mmol) was added under an argon counterflow. The flask was sealed and the mixture stirred at room temperature for 40 h. The reaction was quenched at 0 °C by addition of pH 7 phosphate buffer solution (1.5 mL) followed by aq. H_2O_2 (30% *w/w* in water, 0.3 mL). Ethyl acetate (2 mL) was added and the resulting mixture was vigorously stirred for 1 h. The mixture was diluted with ethyl acetate (10 mL) and water (5 mL) and the organic phase was separated. The aqueous layer was extracted with ethyl acetate (2 x 10 mL), the combined organic phases were washed with sat. aq. $Na_2S_2O_3$ solution (15 mL) and brine (15 mL), dried over $MgSO_4$, filtered, and the solvent was removed under reduced pressure. The residue (rr > 20:1) was purified by flash chromatography (SiO_2, toluene/methyl-*tert*-butyl ether, 4:1) to give the title compound as a colorless solid (44.1 mg, 84% yield, > 20:1 dr, 91% ee). mp = 106-107 °C, $[\alpha]_D^{20}$ (91% ee) = +5.9 (c = 0.65, $CHCl_3$); ***Note:*** *Rotamers were observed at RT, hence characterization by NMR had to be performed at elevated temperature.* ^{1}H NMR (600 MHz, [D$_6$]-DMSO, 393 K): δ = 7.35 – 7.33 (m, 2H), 7.29 (dd, *J* = 8.4, 6.8 Hz, 2H), 7.24 – 7.21 (m, 1H), 6.83 (ddd, *J* = 15.7, 7.9, 6.2 Hz, 1H), 5.87 (dt, *J* = 15.7, 1.6 Hz, 1H), 5.15 (d, *J* = 5.2 Hz, 1H), 4.64 – 4.62 (m, 1H), 4.17 – 4.13 (m, 1H), 3.65 (s, 3H), 2.77 – 2.73 (m, 1H), 2.66 – 2.60 (m, 1H), 2.57 (s, 3H), 1.30 (s, 9H); ^{13}C NMR (151 MHz, [D$_6$]-DMSO, 393 K): δ = 165.2, 154.0, 145.9, 142.7, 127.0 (2C), 126.3, 125.9 (2C), 121.4, 77.9, 73.6, 60.3, 50.1, 30.1, 29.5, 27.4 (3C); IR (ATR): ṽ = 3438, 2972, 1725, 1694, 1667, 1438, 1393, 1325, 1160, 1043 cm^{-1}; HRMS (ESI$^+$): m/z: calcd. for $C_{19}H_{27}NO_5Na$ [M+Na]$^+$: 372.17814, found: 372.17834.

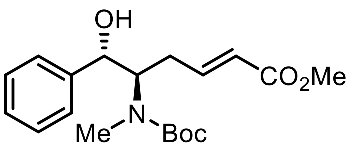

The ee was determined by HPLC analysis: Chiralpak 150 mm IG-3, 3 µm, Ø = 4.6 mm, CH_3CN/water = 40:60, v = 1.0 mL/min, v = 1.0 mL/min, λ = 210 nm, t(minor) = 8.26 min, t(major) = 10.13 min (racemate left, enantioenriched sample right).

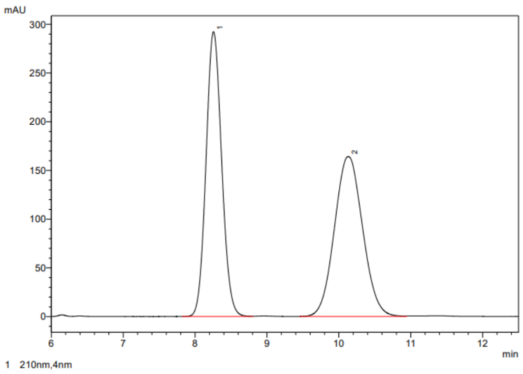

1 210nm,4nm

PDA Ch1 210nm

Peak #	Ret. Time	Area %	Name
1	8,26	49,74	1. Enantiomer
2	10,13	50,26	2. Enantiomer
Total		100,00	

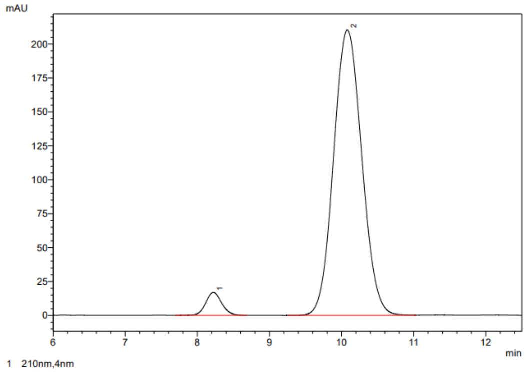

1 210nm,4nm

PDA Ch1 210nm

Peak #	Ret. Time	Area %	Name
1	8,22	4,37	1. Enantiomer
2	10,08	95,63	2. Enantiomer 91.3 % ee
Total		100,00	

Methyl (5*R*,6*S*,*E*)-5-(benzyl(*tert*-butoxycarbonyl)amino)-6-hydroxy-6-phenylhex-2-enoate (18b). A flame-dried Schlenk flask under argon was charged with tris(*trans*-1,2-bis(4-*tert*-butylphenyl)ethene)nickel(0) (**16**) (14.0 mg, 0.015 mmol), phosphoramidite ligand **L1** (9.6 mg, 0.015 mmol) and diene **17b** (142.8 mg, 0.45 mmol). The Schlenk flask was evacuated and refilled with argon (3 cycles). Triethylborane (1 M in THF, 0.45 mL, 0.45 mmol) was added and the resulting solution was stirred for 5 min before benzaldehyde (15.2 µL, 0.15 mmol) was added under an argon counterflow. The flask was sealed and the mixture stirred at room temperature for 42 h. The mixture was cooled to 0 °C before the reaction was quenched by addition of pH 7 phosphate buffer solution (1.5 mL) followed by aq. H_2O_2 (30% *w/w* in water, 0.3 mL). Ethyl acetate (2 mL) was introduced and the mixture was vigorously stirred for 1 h. The mixture was diluted with additional ethyl acetate (10 mL) and water (5 mL) and the organic phase was separated. The aqueous layer was extracted with ethyl acetate (2 x 10 mL), the combined organic phases were washed with sat. aq. $Na_2S_2O_3$ solution (15 mL) and brine (15 mL), dried over $MgSO_4$, filtered, and the solvent was removed under reduced pressure. The residue (rr > 20:1) was purified by flash chromatography (SiO_2, toluene/methyl-*tert*-butyl ether, 15:1 → 10:1) to furnish the title compound as a colorless oil (56.4 mg, 88% yield, > 20:1 dr, 86% ee). $[\alpha]_D^{20}$ (86% ee) = +4.4 (c = 0.55, $CHCl_3$); ***Note:*** *Rotamers were observed at RT, hence characterization by NMR had to be performed at elevated temperature.* ^{1}H NMR (600 MHz, [d^6]-DMSO, 393 K): δ = 7.34 – 7.28 (m, 4H), 7.26 – 7.23 (m, 1H), 7.22 – 7.16 (m, 3H), 7.10 – 7.08 (m, 2H), 6.62 (ddd, *J* = 15.7, 7.8, 6.6 Hz, 1H), 5.63 (dt, *J* = 15.7, 1.5 Hz, 1H), 5.18 (s, 1H), 4.84 (d, *J* = 7.6 Hz, 1H), 4.24 (d, *J* = 15.6 Hz, 1H), 3.93 – 3.90 (m, 1H), 3.87 (d, *J* = 15.7 Hz, 1H), 3.62 (s, 3H), 2.75 – 2.71 (m, 1H), 2.69 – 2.63 (m, 1H), 1.35 (s, 9H); ^{13}C NMR (151 MHz, [d^6]-DMSO, 393 K): δ = 165.0, 154.4, 146.1, 142.8, 138.3, 127.2 (2C), 127.1 (2C), 127.0 (2C), 126.4, 126.0 (2C), 125.9, 121.2, 78.7, 73.7, 62.5, 50.0, 49.3, 31.3, 27.4 (3C); *Note: the signal at 49.3 ppm shows very low intensity.* IR (ATR): ṽ = 3462, 2974, 1724, 1691, 1663, 1454, 1366, 1163, 701 cm^{-1}; HRMS (ESI$^+$): m/z: calcd. for $C_{25}H_{31}NO_5Na$ [M+Na]$^+$: 448.20944, found: 448.20897.

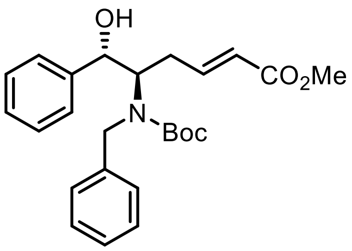

The ee was determined by HPLC analysis: Chiralpak 150 mm IG-3, 3 µm, Ø = 4.6 mm, CH_3CN/water = 50:50, v = 1.0 mL/min, v = 1.0 mL/min, λ = 210 nm, t(minor) = 8.95 min, t(major) = 9.90 min (racemate left, enantioenriched sample right).

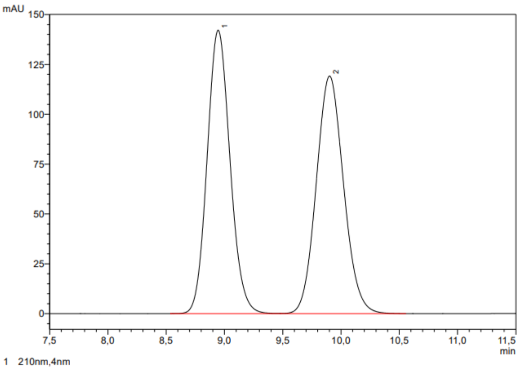

PDA Ch1 210nm

Peak #	Ret. Time	Area %	Name
1	8,95	50,00	1. Enantiomer
2	9,90	50,00	2. Enantiomer
Total		100,00	

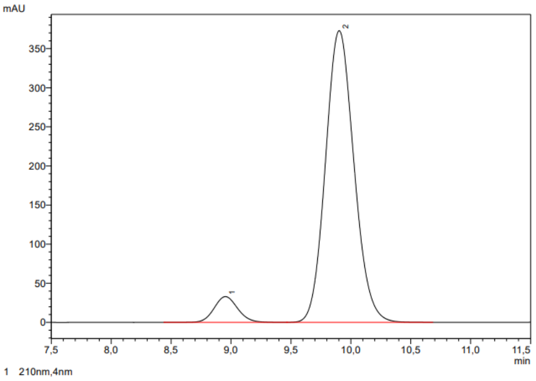

PDA Ch1 210nm

Peak #	Ret. Time	Area %	Name
1	8,95	6,82	1. Enantiomer
2	9,90	93,18	2. Enantiomer 86.4 % ee
Total		100,00	

tert-Butyl ((1S,2R,E)-6-(dimethylamino)-1-hydroxy-6-oxo-1-phenylhex-4-en-2-yl)carbamate (18c). A

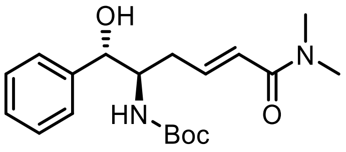

flame-dried Schlenk flask under argon was charged with tris(*trans*-1,2-bis(4-*tert*-butylphenyl)ethene)nickel(0) (**16**) (9.4 mg, 0.01 mmol), phosphoramidite ligand **L1** (6.4 mg, 0.01 mmol) and diene **17c** (48.1 mg, 0.20 mmol). The Schlenk flask was evacuated and refilled with argon (3 cycles). THF (0.7 mL) and triethylborane (1 M in THF, 0.30 mL, 0.30 mmol) were added and the resulting mixture was stirred for 5 min before benzaldehyde (10.1 µL, 0.10 mmol) was added under argon counterflow. The flask was sealed and the mixture stirred at room temperature for 48 h. The mixture was cooled to 0 °C before the reaction was quenched by addition of pH 7 phosphate buffer solution (1.5 mL) followed by aq. H_2O_2 (30% *w/w* in water, 0.2 mL). Ethyl acetate (2 mL) was introduced and the resulting mixture was vigorously stirred for 1 h. The mixture was diluted with additional ethyl acetate (5 mL) and water (5 mL) and the organic phase was separated. The aqueous layer was extracted with ethyl acetate (3 x 5 mL), the combined organic phases were washed with sat. aq. $Na_2S_2O_3$ solution (5 mL) and brine (5 mL), dried over $MgSO_4$, filtered, and the solvent was removed under reduced pressure. The residue was purified by flash chromatography (SiO_2, toluene/acetone, 2.5:1) to give the title compound as a colorless oil (23.4 mg, 67% yield, ≈ 14:1 dr, 95% ee). $[\alpha]_D^{20}$ (95% ee) = +29.9 (c = 0.72, $CHCl_3$); ^{1}H NMR (400 MHz, CD_2Cl_2): δ = 7.38 – 7.32 (m, 4H), 7.29 – 7.25 (m, 1H), 6.71 (dt, *J* = 15.2, 7.1 Hz, 1H), 6.25 (d, *J* = 15.1 Hz, 1H), 4.99 (d, *J* = 8.9 Hz, 1H), 4.85 (d, *J* = 4.0 Hz, 1H), 3.95 (br, 1H), 3.72 (br, 1H), 3.00 (s, 3H), 2.90 (s, 3H), 2.34 – 2.29 (m, 2H), 1.39 (s, 9H); ^{13}C NMR (101 MHz, CD_2Cl_2): δ = 166.6, 156.5, 142.0, 141.7, 128.6 (2C), 127.8, 126.8 (2C), 123.1, 79.8, 76.5, 56.1, 37.5, 35.7, 32.3, 28.5 (3C); IR (ATR): ṽ = 3338, 2929, 1699, 1659, 1603, 1496, 1394, 1251, 1168, 703 cm^{-1}; HRMS (ESI$^+$): m/z: calcd. for $C_{19}H_{28}N_2O_4Na$ [M+Na]$^+$: 371.19413, found: 371.19411.

The ee was determined by HPLC analysis: Chiralpak 150 mm IG-3, 3 µm, Ø = 4.6 mm, CH_3CN/H_2O = 30:70, v = 1.0 mL/min, λ = 220 nm, t(minor) = 11.34 min, t(major) = 19.71 min (racemate left, enantioenriched sample right). The diastereomeric ratio of the enantioenriched sample was determined as dr ≈ 13.8 : 1.0.

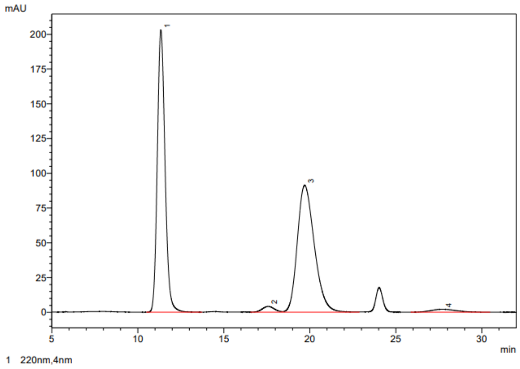

PDA Ch1 220nm

Peak #	Ret. Time	Area %	Name
1	11,34	48,58	1. enantiomer major diastereomer
2	17,58	1,60	1. enantiomer minor diastereomer
3	19,71	48,16	2. enantiomer major diastereomer
4	27,71	1,65	2. enantiomer minor diastereomer
Total		100,00	

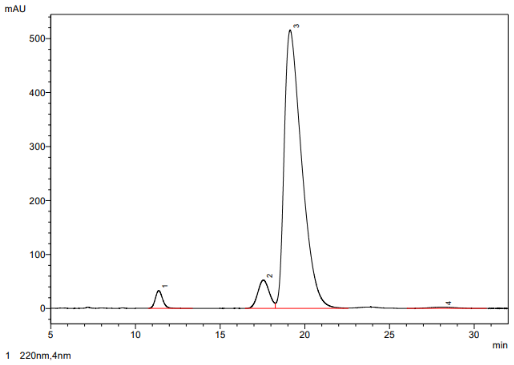

PDA Ch1 220nm

Peak #	Ret. Time	Area %	Name
1	11,38	2,44	1. enantiomer major diastereomer
2	17,55	6,07	1. enantiomer minor diastereomer 79.9 % ee
3	19,14	90,81	2. enantiomer major diastereomer 94.8 % ee
4	28,12	0,68	2. enantiomer minor diastereomer
Total		100,00	

***tert*-Butyl ((1*S*,2*R*,*E*)-1-hydroxy-6-(methoxy(methyl)amino)-6-oxo-1-phenylhex-4-en-2-yl)carbamate (18d).**

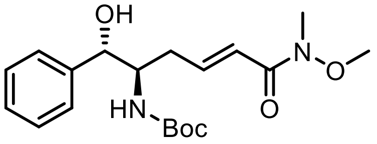

A flame-dried Schlenk flask under argon was charged with tris(*trans*-1,2-bis(4-*tert*-butylphenyl)ethene)nickel(0) (**16**) (9.4 mg, 0.01 mmol), phosphoramidite ligand **L1** (6.4 mg, 0.01 mmol) and diene **17d** (51.3 mg, 0.20 mmol). The Schlenk flask was evacuated and refilled with argon (3 cycles). Triethylborane (1 M in THF, 0.30 mL, 0.30 mmol) was added and the resulting solution was stirred for 5 min before benzaldehyde (10.1 µL, 0.10 mmol) was added under an argon counterflow. The flask was sealed and the mixture stirred at room temperature for 72 h. The mixture was cooled to 0 °C before the reaction was quenched by addition of pH 7 phosphate buffer solution (1.5 mL) followed by aq. H_2O_2 (30% *w/w* in water, 0.2 mL). Ethyl acetate (2 mL) was introduced and the resulting mixture vigorously stirred for 1 h. The mixture was diluted with additional ethyl acetate (5 mL) and water (5 mL) and the organic phase was separated. The aqueous layer was extracted with ethyl acetate (3 x 5 mL), the combined organic phases were washed with sat. aq. $Na_2S_2O_3$ solution (5 mL) and brine (5 mL), dried over $MgSO_4$, filtered, and the solvent was removed under reduced pressure. The residue was purified by flash chromatography (SiO_2, toluene/methyl-*tert*-butyl ether, 2:1 → 1:1.5) to give the title compound as a colorless oil (26.0 mg, 71% yield, > 20:1 dr, 96% ee). $[\alpha]_D^{20}$ (96% ee) = +35.2 (c = 0.65, $CHCl_3$); ^1H NMR (400 MHz, CD_2Cl_2): δ = 7.38 – 7.26 (m, 5H), 6.81 (dt, *J* = 15.4, 7.2 Hz, 1H), 6.40 (dt, *J* = 15.5, 1.5 Hz, 1H), 4.87 (t, *J* = 3.9 Hz, 1H), 4.79 (d, *J* = 8.6 Hz, 1H), 3.97 (br, 1H), 3.66 (s, 3H), 3.37 (s, 1H), 3.17 (s, 3H), 1.40 (s, 9H); ^{13}C NMR (101 MHz, CD_2Cl_2): δ = 166.6, 156.6, 143.5, 141.5, 128.6 (2C), 128.0, 126.8 (2C), 121.7, 80.0, 76.6, 62.1, 56.2, 32.5, 28.4 (3C); IR (ATR): ṽ = 3338, 2929, 1699, 1659, 1603, 1496, 1394, 1365, 1251, 1168, 703 cm^{-1}; HRMS (ESI^+): m/z: calcd. for $C_{19}H_{28}N_2O_4Na$ $[M+Na]^+$: 371.19413, found: 371.19411.

The ee was determined by HPLC analysis: Chiralpak 150 mm IG-3, 3 µm, Ø = 4.6 mm, CH_3CN/20mM NH_4CO_3 (pH = 9) = 30:70, v = 1.0 mL/min, λ = 210 nm, t(minor) = 16.01 min, t(major) = 18.96 min (racemate left, enantioenriched sample right).

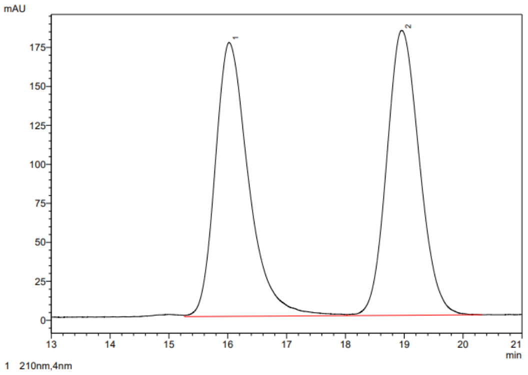

PDA Ch1 210nm

Peak #	Ret. Time	Area %	Name
1	16,02	49,70	1. Enantiomer
2	18,96	50,30	2. Enantiomer
Total		100,00	

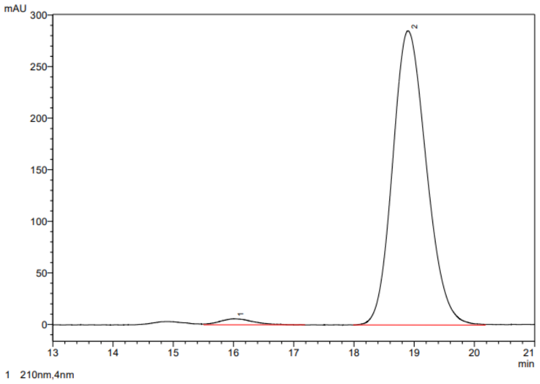

PDA Ch1 210nm

Peak #	Ret. Time	Area %	Name
1	16,01	2,04	1. Enantiomer
2	18,90	97,96	2. Enantiomer 95.9 % ee
Total		100,00	

Methyl (5*R*,6*S*,*E*)-5-((*tert*-butoxycarbonyl)amino)-6-hydroxy-6-(4-methoxyphenyl)hex-2-enoate

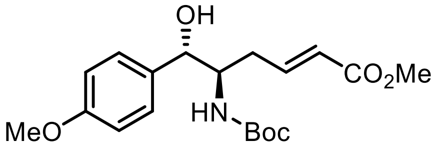

(21). The title compound was prepared according to the general procedure **A** and oxidative workup **I**, using 4-methoxy benzaldehyde (24.3 µL, 0.20 mmol) and diene **13a** (136.4 mg, 0.60 mmol); the reaction time was 48 h. The crude material (rr = 12:1) was purified by flash chromatography (SiO_2, toluene/*tert*-butyl methyl ether, 3:1) and the product was obtained as an off-white solid (61.5 mg, 84% yield, dr > 20:1, 93% ee). mp = 108-109 °C; $[\alpha]_D^{20}$ (99% ee, sample after recrystallization) = +9.6 (c = 0.50, $CHCl_3$); ^1H NMR (400 MHz, CD_2Cl_2): δ = 7.28 – 7.25 (m, 2H), 6.91 – 6.81 (m, 3H), 5.80 (dt, *J* = 15.6, 1.5 Hz, 1H), 4.81 (s, 1H), 4.72 (d, *J* = 8.8 Hz, 1H), 3.92 (s, 1H), 3.79 (s, 3H), 3.67 (s, 3H), 2.98 (s, 1H), 2.38 – 2.20 (m, 2H), 1.40 (s, 9H); ^{13}C NMR (101 MHz, CD_2Cl_2): δ = 166.8, 159.7, 156.5, 145.9, 133.3, 127.8 (2C), 123.5, 114.1 (2C), 80.0, 76.0, 55.9, 55.6, 51.7, 32.6, 28.4 (3C); IR (ATR): ṽ = 3363, 1722, 1679, 1512, 1434, 1324, 1248, 1160, 980, 827 cm^{-1}; HRMS (ESI^+): *m/z*: calcd. for $C_{19}H_{27}NO_6Na$ $[M+Na]^+$: 388.17306, found: 388.17338.

24.0 mg of the product were recrystallized from *tert*-butyl methyl ether/*iso*-hexane to give the enantioenriched product in 99% ee (21.3 mg).

The ee was determined by HPLC analysis: Chiralpak 150 mm IC-3, 3 µm, 4.6 mm ∅, *n*-heptane/ethanol = 90:10, v = 1.0 mL/min, λ = 225 nm, t(minor) = 16.04 min, t(major) = 18.62 min (racemate: top left, enantioenriched sample: top right, recrystallized enantioenriched sample: bottom left).

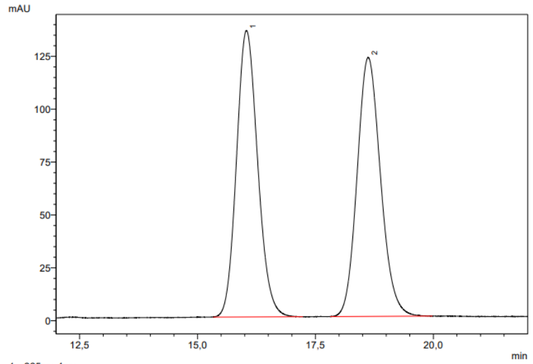

1 225nm,4nm

PDA Ch1 225nm

Peak #	Ret. Time	Area %	Name
1	16,04	50,00	1. Enantiomer
2	18,62	50,00	2. Enantiomer
Total		100,00	

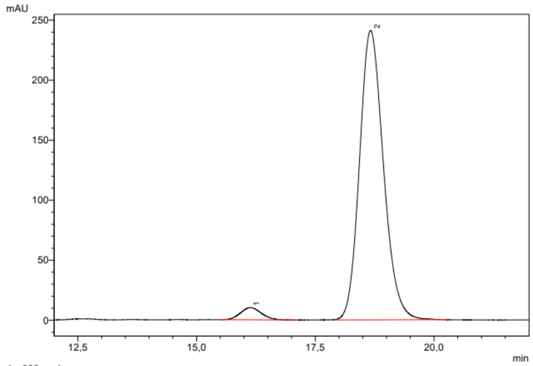

1 225nm,4nm

PDA Ch1 225nm

Peak #	Ret. Time	Area %	Name
1	16,13	3,58	1. Enantiomer
2	18,66	96,42	2. Enantiomer 93.0 % ee
Total		100,00	

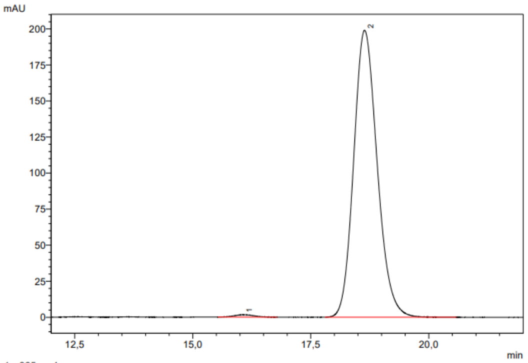

1 225nm,4nm

PDA Ch1 225nm

Peak #	Ret. Time	Area %	Name
1	16,06	0,71	1. Enantiomer
2	18,64	99,29	2. Enantiomer 98.6 % ee
Total		100,00	

Methyl 4-((1*S*,2*R*,*E*)-2-((*tert*-butoxycarbonyl)amino)-1-hydroxy-6-methoxy-6-oxohex-4-en-1-yl)-benzoate (22).

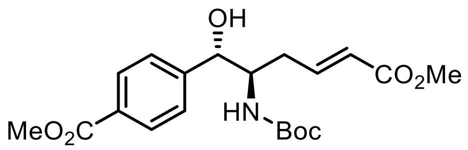

The title compound was prepared according to the general procedure **A** and oxidative workup **I**, using methyl 4-formylbenzoate (32.8 mg, 0.20 mmol) and diene **13a** (90.9 mg, 0.40 mmol); the reaction time was 18 h. The crude material (rr > 20:1) was purified by flash chromatography (SiO_2, toluene/*tert*-butyl methyl ether, 3:1) and the product was obtained as an off-white solid (71.5 mg, 91% yield, dr > 20:1, 91% ee). mp = 128-129 °C; $[\alpha]_D^{20}$ (99% ee, sample after recrystallization) = +7.4 (c = 0.50, $CHCl_3$); ^1H NMR (400 MHz, CD_2Cl_2): δ = 8.02 – 7.99 (m, 2H), 7.47 – 7.43 (m, 2H), 6.82 (m, 1H), 5.79 (dt, *J* = 15.6, 1.5 Hz, 1H), 4.96 (t, *J* = 3.9 Hz, 1H), 4.80 (s, 1H), 3.96 (s, 1H), 3.89 (s, 3H), 3.66 (s, 3H), 3.40 (s, 1H), 2.30 – 2.26 (m, 2H), 1.41 (s, 9H); ^{13}C NMR (101 MHz, CD_2Cl_2): δ = 167.1, 166.7, 156.5, 146.5, 145.5, 130.1, 129.9 (2C), 126.7 (2C), 123.7, 80.3, 76.2, 56.0, 52.4, 51.7, 32.1, 28.4 (3C); IR (ATR): ṽ = 3368, 1720, 1700, 1679, 1519, 1439, 1276, 1164, 1018, 775 cm^{-1}; HRMS (ESI^+): *m/z*: calcd. for $C_{20}H_{27}NO_7Na$ $[M+Na]^+$: 416.16797, found: 416.16840.

62.5 mg of the product were recrystallized from *tert*-butyl methyl ether/*iso*-hexane to give the enantioenriched product in 99% ee (49.5 mg).

The ee was determined by HPLC analysis: Chiralpak 150 mm IC-3, 3 µm, 4.6 mm ∅, *n*-heptane/ethanol = 75:25, v = 1.0 mL/min, λ = 220 nm, t(minor) = 5.44 min, t(major) = 10.98 min (racemate: top left, enantioenriched sample: top right, recrystallized enantioenriched sample: bottom left).

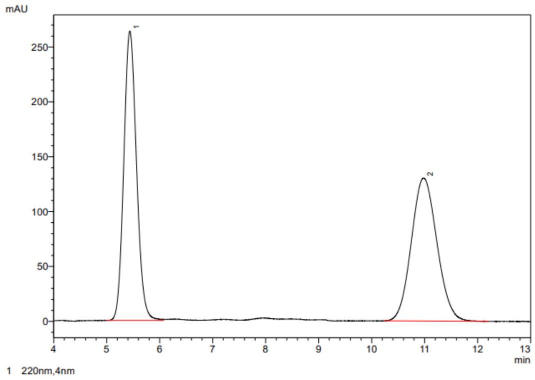

PDA Ch1 220nm

Peak #	Ret. Time	Area %	Name
1	5,44	50,11	1. Enantiomer
2	10,98	49,89	2. Enantiomer
Total		100,00	

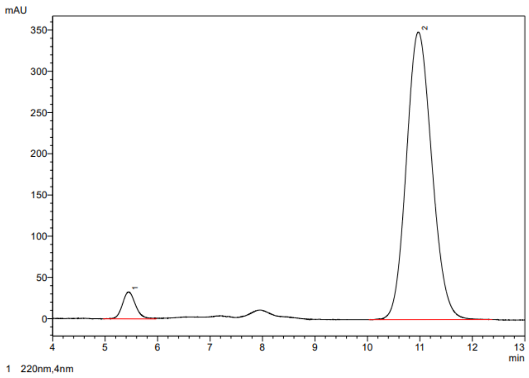

PDA Ch1 220nm

Peak #	Ret. Time	Area %	Name
1	5,45	4,59	1. Enantiomer
2	10,97	95,41	2. Enantiomer 90.8 % ee
Total		100,00	

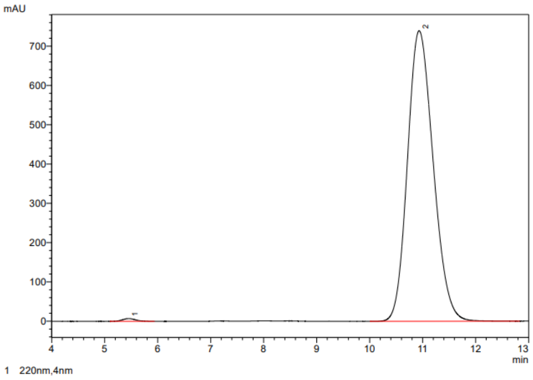

PDA Ch1 220nm

Peak #	Ret. Time	Area %	Name
1	5,45	0,50	1. Enantiomer
2	10,93	99,50	2. Enantiomer 99.0 % ee
Total		100,00	

Methyl (5*R*,6*S*,*E*)-5-((*tert*-butoxycarbonyl)amino)-6-(4-fluorophenyl)-6-hydroxyhex-2-enoate (23).

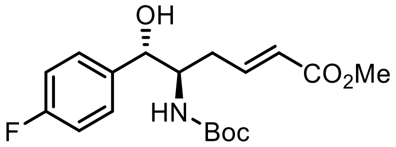

The title compound was prepared according to the general procedure **A** and oxidative workup **I**, using 4-fluorobenzaldehyde (21.5 µL, 0.20 mmol) and diene **13a** (90.9 mg, 0.40 mmol); the reaction time was 24 h. The crude material (rr = 10:1) was purified by flash chromatography (SiO_2, toluene/*tert*-butyl methyl ether, 4:1) and the product was obtained as a colorless solid (60.7 mg, 86% yield, dr > 20:1, 92% ee). mp = 127-128 °C; $[\alpha]_D^{20}$ (99% ee, sample after recrystallization) = +17.6 (c = 0.50, $CHCl_3$); ^{1}H NMR (400 MHz, CD_2Cl_2): δ = 7.37 – 7.32 (m, 2H), 7.09 – 7.03 (m, 2H), 6.87 – 6.79 (m, 1H), 5.80 (dt, *J* = 15.6, 1.5 Hz, 1H), 4.86 (t, *J* = 4.0 Hz, 1H), 4.74 (d, *J* = 8.8 Hz, 1H), 3.92 (s, 1H), 3.67 (s, 3H), 3.23 (s, 1H), 2.36 – 2.22 (m, 2H), 1.40 (s, 9H); ^{13}C NMR (101 MHz, CD_2Cl_2): δ = 166.8, 162.7 (d, $^1J_{CF}$ = 245.0 Hz), 156.5, 145.6, 137.2, 128.4 (d, $^3J_{CF}$ = 8.0 Hz, 2C), 123.7, 115.5 (d, $^2J_{CF}$ = 21.5 Hz, 2C), 80.2, 75.9, 56.0, 51.7, 32.3, 28.4 (3C); ^{19}F NMR (282 MHz, CD_2Cl_2): δ = -115.7; IR (ATR): ṽ = 3372, 2951, 1713, 1679, 1523, 1504, 1324, 1217, 1164, 1026, 851 cm^{-1}; HRMS (ESI$^+$): *m/z*: calcd. for $C_{18}H_{24}NO_5FNa$ [M+Na]$^+$: 376.15307, found: 376.15298.

25.0 mg of the product were recrystallized from dichloromethane/*iso*-hexane to give the enantioenriched product in 99% ee (15.3 mg).

The ee was determined by HPLC analysis: Chiralpak 150 mm IC-3, 3 µm, 4.6 mm ∅, *n*-heptane/ethanol = 95:5, v = 1.0 mL/min, λ = 220 nm, t(minor) = 7.09 min, t(major) = 18.20 min (racemate: top left, enantioenriched sample: top right, recrystallized enantioenriched sample: bottom left).

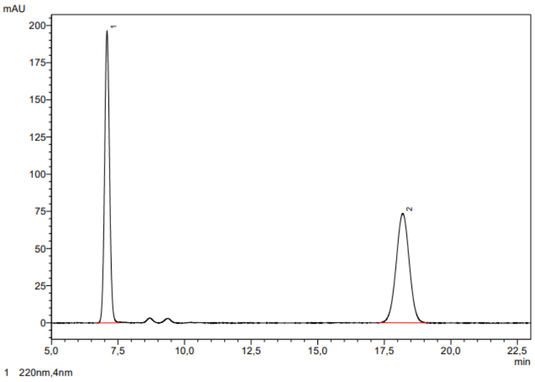

PDA Ch1 220nm

Peak #	Ret. Time	Area %	Name
1	7,09	50,01	1. Enantiomer
2	18,20	49,99	2. Enantiomer
Total		100,00	

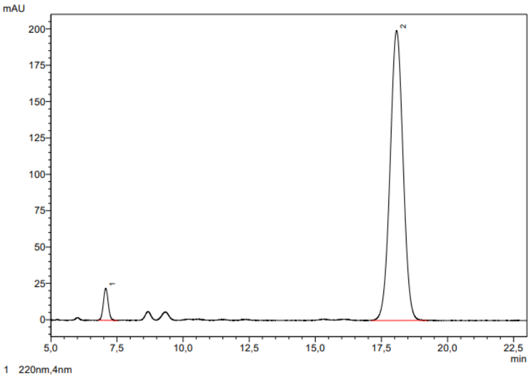

PDA Ch1 220nm

Peak #	Ret. Time	Area %	Name
1	7,07	3,98	1. Enantiomer
2	18,07	96,02	2. Enantiomer 92.0 % ee
Total		100,00	

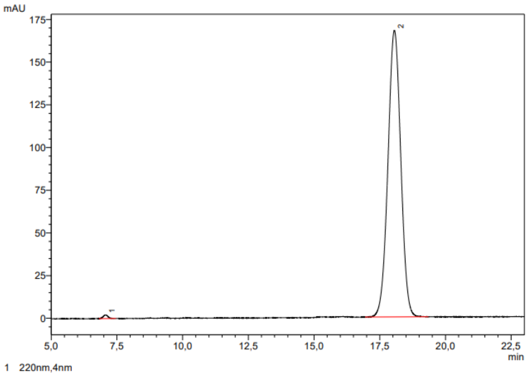

PDA Ch1 220nm

Peak #	Ret. Time	Area %	Name
1	7,07	0,49	1. Enantiomer
2	18,06	99,51	2. Enantiomer 99.0 % ee
Total		100,00	

Methyl (5*R*,6*S*,*E*)-5-((*tert*-butoxycarbonyl)amino)-6-hydroxy-6-(4-(trifluoromethyl)phenyl)hex-2-enoate (24). The title compound was prepared according to the general procedure **A** and oxidative workup **I**, using 4-(trifluoromethyl)benzaldehyde (27.3 µL, 0.20 mmol) and diene **13a** (90.9 mg, 0.40 mmol); the reaction time was 17 h. The crude material (rr = 18:1) was purified by flash chromatography (SiO_2, toluene/*tert*-butyl methyl ether, 4:1) and the product was obtained as an off-white solid (67.5 mg, 84% yield, dr > 20:1, 84% ee). Analytical data see below.

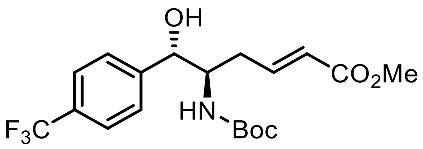

The ee was determined by HPLC analysis: Chiralpak 150 mm IC-3, 3 µm, 4.6 mm ∅, *n*-heptane/ethanol = 95:5, v = 1.0 mL/min, λ = 220 nm, t(minor) = 4.40 min, t(major) = 9.34 min (racemate: left, enantioenriched sample: right).

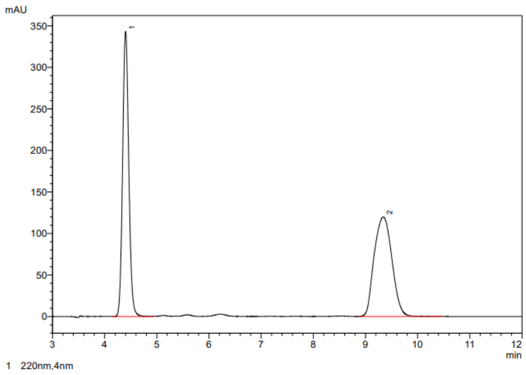

PDA Ch1 220nm

Peak #	Ret. Time	Area %	Name
1	4,40	49,76	1. Enantiomer
2	9,34	50,24	2. Enantiomer
Total		100,00	

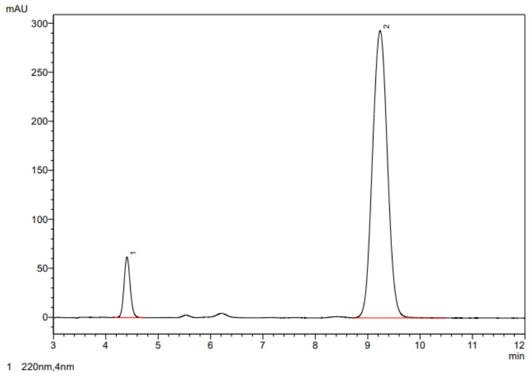

PDA Ch1 220nm

Peak #	Ret. Time	Area %	Name
1	4,40	7,99	1. Enantiomer
2	9,24	92,01	2. Enantiomer 84.0 % ee
Total		100,00	

Reaction at –10 °C. The reaction was performed analogously but the reaction mixture was cooled to –10 °C before addition of the aldehyde. The mixture was then stirred at –10°C for 72 h. After workup, the crude product (rr = 18:1) was purified by flash chromatography (SiO_2, toluene/*tert*-butyl methyl ether, 4:1) and the product was obtained as an off-white solid (61.5 mg, 77% yield, dr > 20:1, 89% ee). mp = 95-96 °C; $[\alpha]_D^{20}$ (99% ee, sample after recrystallization) = +33.4 (c = 0.50, $CHCl_3$); ^1H NMR (400 MHz, CD_2Cl_2): δ = 7.63 (d, *J* = 8.2 Hz, 2H), 7.51 (d, *J* = 8.1 Hz, 2H), 6.85 – 6.78 (m, 1H), 5.79 (dt, *J* = 15.6, 1.5 Hz, 1H), 4.95 (t, *J* = 4.0 Hz, 1H), 4.84 (d, *J* = 8.8 Hz, 1H), 3.96 (s, 1H), 3.66 (s, 3H), 3.59 (s, 1H), 2.32 – 2.28 (m, 2H), 1.39 (s, 9H); ^{13}C NMR (101 MHz, CD_2Cl_2): δ = 166.8, 156.5, 145.7, 145.4, 130.0 (q, $^2J_{CF}$ = 32.2 Hz), 127.2 (2C), 125.6 (q, $^3J_{CF}$ = 3.9 Hz, 2C), 124.7 (q, $^1J_{CF}$ = 271.8 Hz), 123.8, 80.4, 75.9, 56.0, 51.7, 32.1, 28.4 (3C); ^{19}F NMR (282 MHz, CD_2Cl_2): δ = -62.8; IR (ATR): ṽ = 3361, 1705, 1678, 1521, 1325, 1161, 1115, 1066, 836 cm^{-1}; HRMS (ESI$^+$): *m/z*: calcd. for $C_{19}H_{24}NO_5F_3Na$ $[M+Na]^+$: 426.14988, found: 426.14999.

37.0 mg of the product were recrystallized from ethyl acetate/*iso*-hexane to give the enantioenriched product in 99% ee (26.9 mg).

The ee was determined by HPLC analysis: Chiralpak 150 mm IC-3, 3 µm, 4.6 mm ∅, *n*-heptane/ethanol = 95:5, v = 1.0 mL/min, λ = 220 nm, t(minor) = 4.40 min, t(major) = 9.34 min (racemate: top left, enantioenriched sample: top right, recrystallized enantioenriched sample: bottom left).

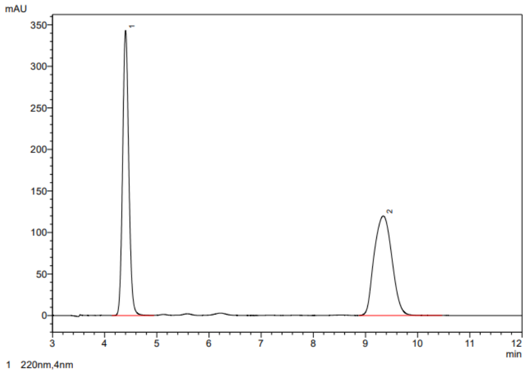

1 220nm,4nm

PDA Ch1 220nm

Peak #	Ret. Time	Area %	Name
1	4,40	49,76	1. Enantiomer
2	9,34	50,24	2. Enantiomer
Total		100,00	

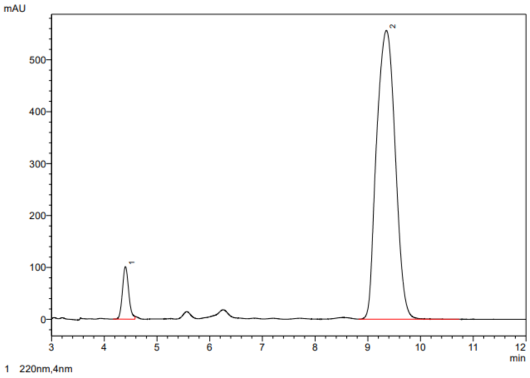

1 220nm,4nm

PDA Ch1 220nm

Peak #	Ret. Time	Area %	Name
1	4,40	5,71	1. Enantiomer
2	9,35	94,29	2. Enantiomer 88.6 % ee
Total		100,00	

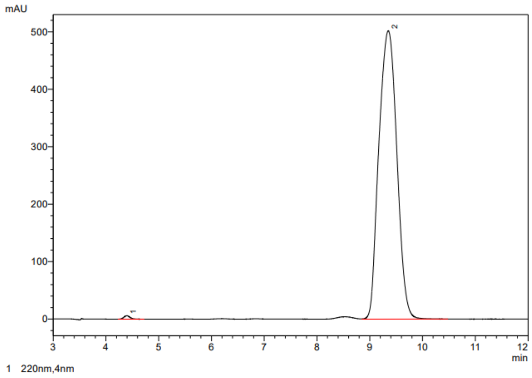

1 220nm,4nm

PDA Ch1 220nm

Peak #	Ret. Time	Area %	Name
1	4,39	0,42	1. Enantiomer
2	9,35	99,58	2. Enantiomer 99.2 % ee
Total		100,00	

Methyl (5*R*,6*S*,*E*)-5-((*tert*-butoxycarbonyl)amino)-6-(4-chlorophenyl)-6-hydroxyhex-2-enoate (25).

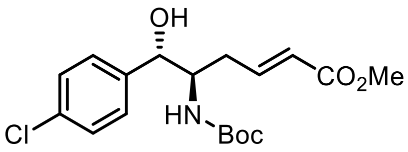

The title compound was prepared according to the general procedure **A** and oxidative workup **I**, using 4-chlorobenzaldehyde (28.1 mg, 0.20 mmol) and diene **13a** (90.9 mg, 0.40 mmol); the reaction time was 20 h. The crude material (rr = 17:1) was purified by flash chromatography (SiO_2, toluene/*tert*-butyl methyl ether, 3:1) and the product was obtained as an off-white solid (62.6 mg, 85% yield, dr > 20:1, 87% ee). mp = 130-131 °C; $[\alpha]_D^{20}$ (99% ee, sample after recrystallization) = +20.8 (c = 0.50, $CHCl_3$); ^1H NMR (400 MHz, CD_2Cl_2): δ = 7.37 – 7.30 (m, 4H), 6.86 – 6.78 (m, 1H), 5.80 (dt, *J* = 15.6, 1.5 Hz, 1H), 4.87 (t, *J* = 3.9 Hz, 1H), 4.73 (d, *J* = 8.6 Hz, 1H), 3.92 (s, 1H), 3.67 (s, 3H), 3.23 (s, 1H), 2.35 – 2.22 (m, 2H), 1.40 (s, 9H); ^{13}C NMR (101 MHz, CD_2Cl_2): δ = 166.9, 156.5, 145.7, 140.1, 133.6, 128.8 (2C), 128.2 (2C), 123.6, 80.2, 75.7, 55.9, 51.7, 32.2, 28.4 (3C); IR (ATR): ṽ = 3451, 1682, 1526, 1439, 1327, 1282, 1166, 1012, 978, 825 cm^{-1}; HRMS (ESI$^+$): *m/z*: calcd. for $C_{18}H_{24}NO_5ClNa$ $[M+Na]^+$: 392.12352, found: 392.12347.

24.0 mg of the product were recrystallized from dichloromethane/*iso*-hexane to give the enantioenriched product in 99% ee (16.1 mg).

The ee was determined by HPLC analysis: Chiralcel 150 mm IG-3, 3 µm, 4.6 mm ∅, CH_3CN/water = 40:60; v = 1.0 mL/min, λ = 220 nm, t(minor) = 7.82 min, t(major) = 8.82 min (racemate: top left, enantioenriched sample: top right, recrystallized enantioenriched sample: bottom left).

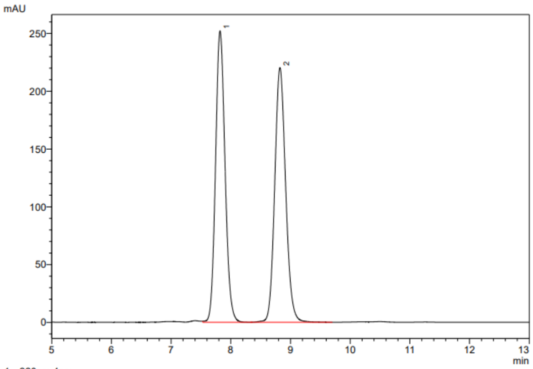

1 220nm,4nm

PDA Ch1 220nm

Peak #	Ret. Time	Area %	Name
1	7,82	49,85	1. Enantiomer
2	8,82	50,15	2. Enantiomer
Total		100,00	

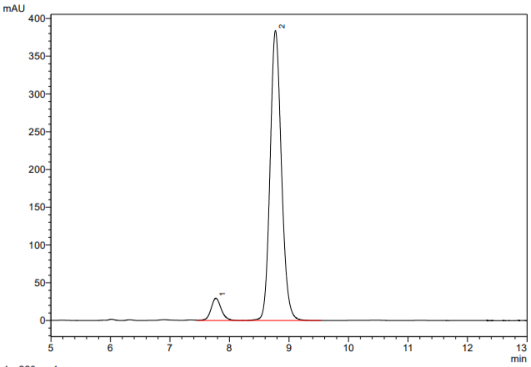

1 220nm,4nm

PDA Ch1 220nm

Peak #	Ret. Time	Area %	Name
1	7,77	6,48	1. Enantiomer
2	8,77	93,52	2. Enantiomer 87.0 % ee
Total		100,00	

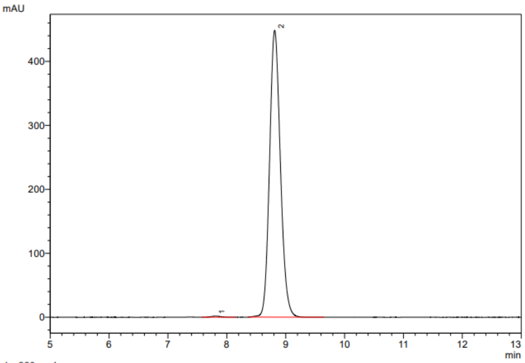

1 220nm,4nm

PDA Ch1 220nm

Peak #	Ret. Time	Area %	Name
1	7,81	0,34	1. Enantiomer
2	8,81	99,66	2. Enantiomer 99.3 % ee
Total		100,00	

Methyl (5*R*,6*S*,*E*)-5-((*tert*-butoxycarbonyl)amino)-6-hydroxy-6-(m-tolyl)hex-2-enoate (26). The title compound was prepared according to the general procedure **A** and oxidative workup **I**, using 3-methylbenzaldehyde (23.5 µL, 0.20 mmol) and diene **13a** (90.9 mg, 0.40 mmol); the reaction time was 40 h. The crude material (rr = 8:1) was purified by flash chromatography (SiO_2, toluene/*tert*-butyl methyl ether, 4:1) and the product was obtained as an off-white solid (56.0 mg, 80% yield, dr > 20:1, 87% ee). mp = 95-96 °C; $[\alpha]_D^{20}$ (95% ee, sample after recrystallization) = +17.0 (c = 0.50, $CHCl_3$); ^{1}H NMR (400 MHz, CD_2Cl_2): δ = 7.26 – 7.22 (m, 1H), 7.18 – 7.09 (m, 3H), 6.87 – 6.80 (m, 1H), 5.79 (dt, *J* = 15.6, 1.5 Hz, 1H), 4.85 – 4.81 (m, 2H), 3.94 (s, 1H), 3.66 (s, 3H), 3.18 (s, 1H), 2.35 (s, 3H), 2.33 – 2.21 (m, 2H), 1.41 (s, 9H); ^{13}C NMR (101 MHz, CD_2Cl_2): δ = 166.9, 156.5, 146.1, 141.3, 138.5, 128.7, 128.6, 127.3, 123.6, 123.5, 80.0, 76.3, 56.0, 51.7, 32.3, 28.4 (3C), 21.6; IR (ATR): ṽ = 3352, 2934, 1723, 1680, 1526, 1433, 1309, 1159, 1013, 974 cm^{-1}; HRMS (ESI$^+$): *m/z*: calcd. for $C_{19}H_{27}NO_5Na$ [M+Na]$^+$: 372.17814, found: 372.17811.

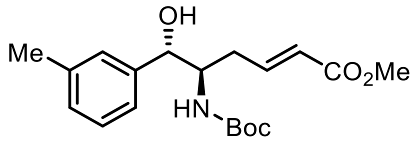

40 mg of the product were recrystallized from *tert*-butyl methyl ether/*iso*-hexane to give the enantioenriched product in 95% ee (19.9 mg).

The ee was determined by HPLC analysis: Chiralpak 150 mm IC-3, 3 µm, 4.6 mm ∅, *n*-heptane/ethanol = 90:10, v = 1.0 mL/min, λ = 220 nm, t(minor) = 4.90 min, t(major) = 13.32 min (racemate: top left, enantioenriched sample: top right, recrystallized enantioenriched sample: bottom left).

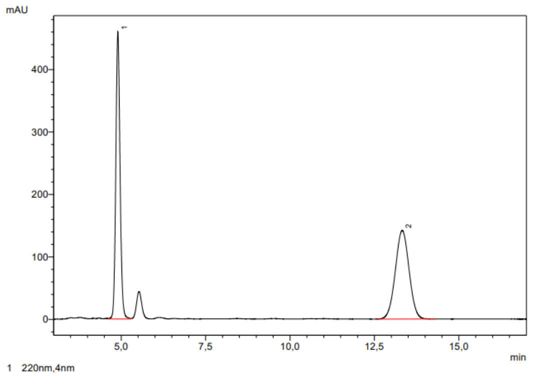

PDA Ch1 220nm

Peak #	Ret. Time	Area %	Name
1	4,90	49,99	1. Enantiomer
2	13,32	50,01	2. Enantiomer
Total		100,00	

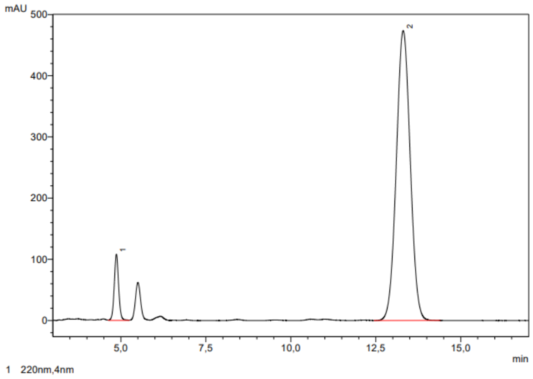

PDA Ch1 220nm

Peak #	Ret. Time	Area %	Name
1	4,87	6,71	1. Enantiomer
2	13,31	93,29	2. Enantiomer 86.6 % ee
Total		100,00	

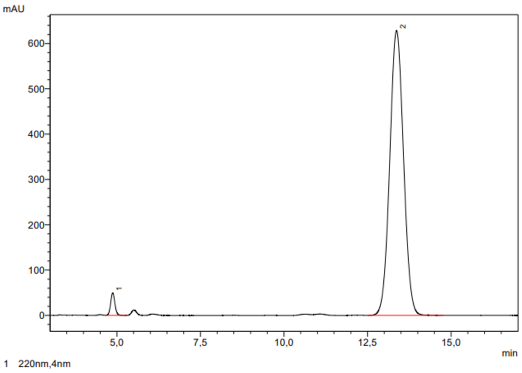

PDA Ch1 220nm

Peak #	Ret. Time	Area %	Name
1	4,88	2,39	1. Enantiomer
2	13,37	97,61	2. Enantiomer 95.2 % ee
Total		100,00	

Methyl (5*R*,6*S*,*E*)-5-((*tert*-butoxycarbonyl)amino)-6-hydroxy-6-(o-tolyl)hex-2-enoate (27). The title compound was prepared according to the general procedure **A** and oxidative workup **I**, using 2-methylbenzaldehyde (23.1 µL, 0.20 mmol) and diene **13a** (90.9 mg, 0.40 mmol); the reaction time was 30 h. The crude material (rr = 5:1) was purified by flash chromatography (SiO_2, toluene/*tert*-butyl methyl ether, 4:1) and the product was obtained as an off-white solid (49.3 mg, 71% yield, dr > 20:1, 92% ee). mp = 122-123 °C; $[\alpha]_D^{20}$ (99% ee, sample after recrystallization) = +51.8 (c = 0.50, $CHCl_3$); ^1H NMR (400 MHz, CD_2Cl_2): δ = 7.51 – 7.49 (m, 1H), 7.24 – 7.14 (m, 3H), 6.85 – 6.78 (m, 1H), 5.78 (dt, *J* = 15.6, 1.5 Hz, 1H), 5.12 (s, 1H), 4.97 (d, *J* = 8.7 Hz, 1H), 3.92 – 3.84 (m, 1H), 3.66 (s, 3H), 2.75 (s, 1H), 2.43 – 2.27 (m, 2H), 2.37 (s, 3H), 1.41 (s, 9H); ^{13}C NMR (101 MHz, CD_2Cl_2): δ = 166.9, 156.1, 146.5, 139.6, 135.3, 130.9, 127.8, 126.4, 126.3, 123.4, 79.9, 73.3, 54.0, 51.6, 31.2, 28.4 (3C), 19.2; IR (ATR): ṽ = 3359, 1718, 1683, 1657, 1530, 1436, 1317, 1241, 1161, 1023 cm^{-1}; HRMS (ESI^+): *m/z*: calcd. for $C_{19}H_{27}NO_5Na$ $[M+Na]^+$: 372.17814, found: 372.17827.

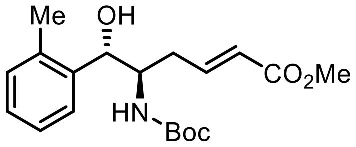

20.5 mg of the product were recrystallized from *tert*-butyl methyl ether/*iso*-hexane to give the enantioenriched product in 99% ee (17.5 mg).

The ee was determined by HPLC analysis: Chiralpak 150 mm IC-3, 3 µm, 4.6 mm ∅, *n*-heptane/ethanol = 95:5, v = 1.0 mL/min, λ = 220 nm, t(minor) = 8.99 min, t(major) = 20.58 min (racemate: top left, enantioenriched sample: top right, recrystallized enantioenriched sample: bottom left).

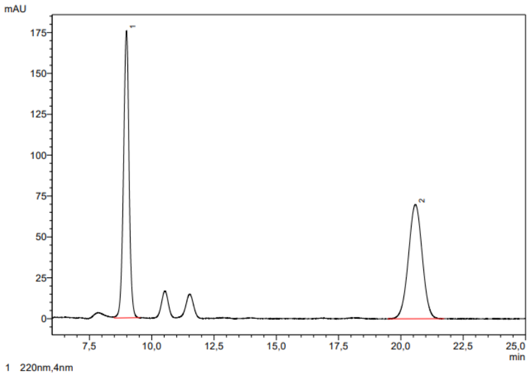

1 220nm,4nm

PDA Ch1 220nm

Peak #	Ret. Time	Area %	Name
1	8,99	50,19	1. Enantiomer
2	20,58	49,81	2. Enantiomer
Total		100,00	

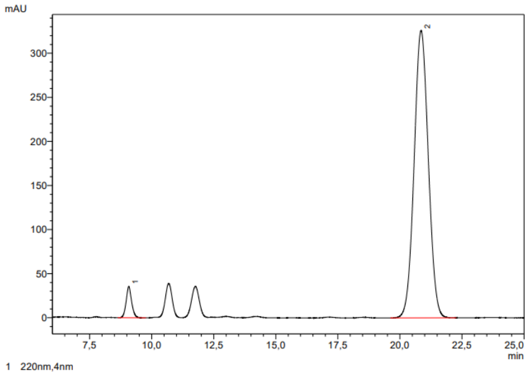

1 220nm,4nm

PDA Ch1 220nm

Peak #	Ret. Time	Area %	Name
1	9,08	4,04	1. Enantiomer
2	20,85	95,96	2. Enantiomer 91.9 % ee
Total		100,00	

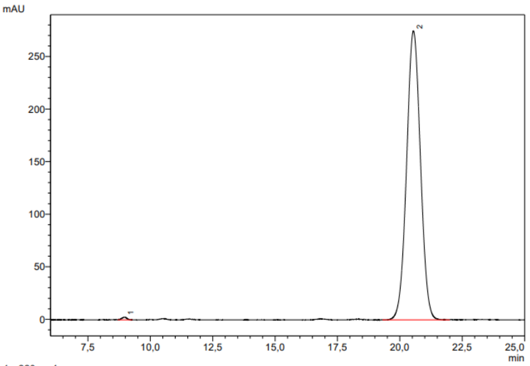

1 220nm,4nm

PDA Ch1 220nm

Peak #	Ret. Time	Area %	Name
1	8,96	0,34	1. Enantiomer
2	20,54	99,66	2. Enantiomer 99.3 % ee
Total		100,00	

Methyl (5*R*,6*S*,*E*)-5-((*tert*-butoxycarbonyl)amino)-6-hydroxy-6-(naphthalen-2-yl)hex-2-enoate (28).

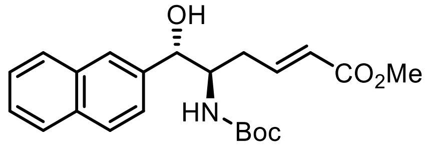

The title compound was prepared according to the general procedure **A** and oxidative workup **I**, using 2-naphthaldehyde (31.2 mg, 0.20 mmol) and diene **13a** (90.9 mg, 0.40 mmol); the reaction time was 23 h. The crude material (rr = 14:1) was purified by flash chromatography (SiO_2, toluene/*tert*-butyl methyl ether, 4:1) and the product was obtained as an off-white solid (65.6 mg, 85% yield, dr > 20:1, 89% ee). mp = 132-133 °C; $[\alpha]_D^{20}$ (99% ee, sample after recrystallization) = +4.2 (c = 0.50, $CHCl_3$); ^{1}H NMR (400 MHz, CD_2Cl_2): δ = 7.86 – 7.83 (m, 4H), 7.52 – 7.46 (m, 3H), 6.87 – 6.79 (m, 1H), 5.77 (dt, *J* = 15.6, 1.5 Hz, 1H), 5.05 (s, 1H), 4.86 (d, *J* = 8.9 Hz, 1H), 4.05 (s, 1H), 3.64 (s, 3H), 3.39 (s, 1H), 2.38 – 2.26 (m, 2H), 1.40 (s, 9H); ^{13}C NMR (101 MHz, CD_2Cl_2): δ =166.8, 156.5, 145.9, 138.9, 133.6, 133.4, 128.4, 128.3, 128.0, 126.6, 126.4, 125.5, 124.7, 123.5, 80.1, 76.5, 56.0, 51.6, 32.3, 28.4 (3C); IR (ATR): ṽ = 3348, 2926, 1714, 1679, 1503, 1353, 1217, 1153, 1121, 819 cm^{-1}; HRMS (ESI$^+$): *m/z*: calcd. for $C_{22}H_{27}NO_5Na$ $[M+Na]^+$: 408.17814, found: 408.17812.

31.0 mg of the product were recrystallized from *tert*-butyl methyl ether/*iso*-hexane to give the enantioenriched product in 99% ee (23.7 mg).

The ee was determined by HPLC analysis: Chiralpak 150 mm IA-3, 3 µm, 4.6 mm ∅, *n*-heptane/ethanol = 95:5, v = 1.0 mL/min, λ = 225 nm, t(minor) = 13.23 min, t(major) = 16.91 min (racemate: top left, enantioenriched sample: top right, recrystallized enantioenriched sample: bottom left).

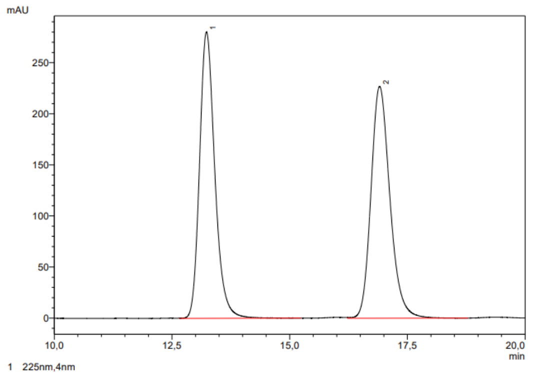

PDA Ch1 225nm

Peak #	Ret. Time	Area %	Name
1	13,23	49,90	1. Enantiomer
2	16,91	50,10	2. Enantiomer
Total		100,00	

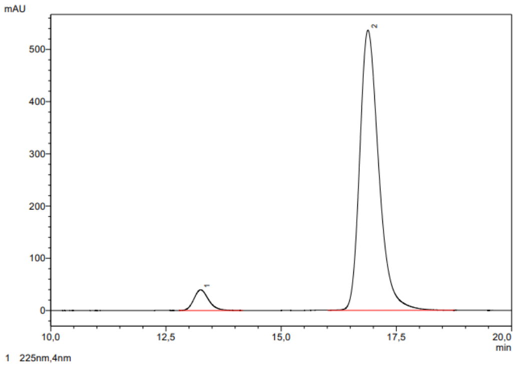

PDA Ch1 225nm

Peak #	Ret. Time	Area %	Name
1	13,25	5,43	1. Enantiomer
2	16,88	94,57	2. Enantiomer 89.1 % ee
Total		100,00	

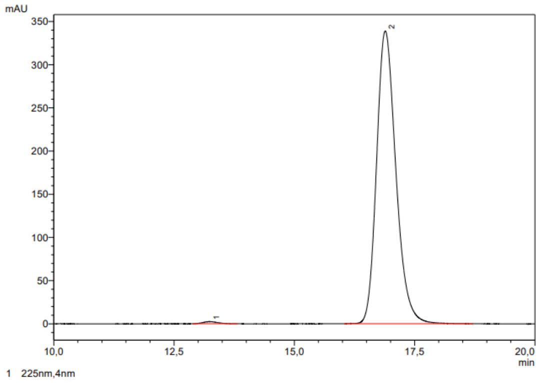

PDA Ch1 225nm

Peak #	Ret. Time	Area %	Name
1	13,24	0,56	1. Enantiomer
2	16,89	99,44	2. Enantiomer 98.9 % ee
Total		100,00	

Methyl (5*R*,6*S*,*E*)-5-((*tert*-butoxycarbonyl)amino)-6-hydroxy-6-(4-(4,4,5,5-tetramethyl-1,3,2-dioxaborolan-2-yl)phenyl)hex-2-enoate (29). The title compound was prepared according to the general procedure **A** and basic workup **II**, using 4-(4,4,5,5-tetramethyl-1,3,2-dioxaborolan-2-yl)benzaldehyde (46.4 mg, 0.20 mmol) and diene **13a** (90.9 mg, 0.40 mmol); the reaction time was 24 h. The crude material (rr = 11:1) was purified by flash chromatography (SiO_2, toluene/*tert*-butyl methyl ether, 4:1) and the product was obtained as an off-white solid (55.7 mg, 0.12 mmol, 60% yield, dr > 20:1, 90% ee). mp = 90 °C; $[\alpha]_D^{20}$ (90% ee) = +10.6 (c = 0.50, $CHCl_3$); ^1H NMR (400 MHz, CD_2Cl_2): δ = 7.76 – 7.74 (m, 2H), 7.37 – 7.35 (m, 2H), 6.86 – 6.79 (m, 1H), 5.78 (dt, *J* = 15.6, 1.5 Hz, 1H), 4.91 (t, *J* = 3.8 Hz, 1H), 4.76 (d, *J* = 8.9 Hz, 1H), 3.96 (s, 1H), 3.66 (s, 3H), 3.20 (s, 1H), 2.28 – 2.20 (m, 2H), 1.41 (s, 9H), 1.33 (s, 12H); ^{13}C NMR (101 MHz, CD_2Cl_2): δ = 166.8, 156.5, 145.8, 144.4, 135.1 (2C), 126.0 (3C), 123.6, 84.3 (2C), 80.1, 76.4, 56.0, 51.7, 32.3, 28.4 (3C), 25.1 (4C); IR (ATR): ṽ = 3360, 1698, 1514, 1358, 1319, 1269, 1143, 1087, 1019, 859 cm^{-1}; HRMS (ESI$^+$): *m/z*: calcd. for $C_{24}H_{36}BNO_7Na$ $[M+Na]^+$: 484.24770, found: 484.24792.

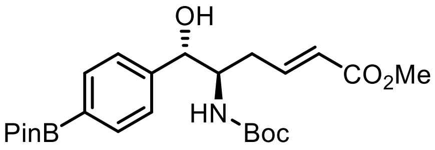

The ee was determined by HPLC analysis: Chiralpak 150 mm IC-3, 3 µm, 4.6 mm ∅, *n*-heptane/ethanol = 95:5, v = 1.0 mL/min, λ = 230 nm, t(minor) = 10.10 min, t(major) = 21.35 min (racemate left, enantioenriched sample right).

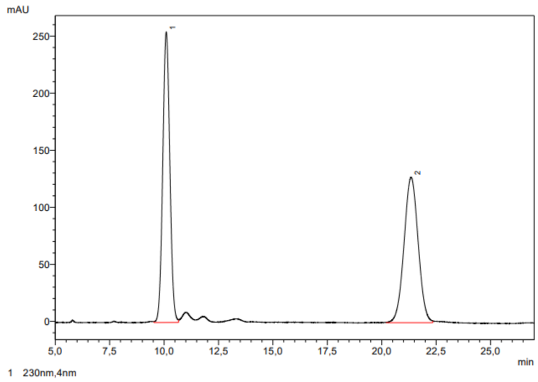

PDA Ch1 230nm

Peak #	Ret. Time	Area %	Name
1	10,10	49,99	1. Enantiomer
2	21,35	50,01	2. Enantiomer
Total		100,00	

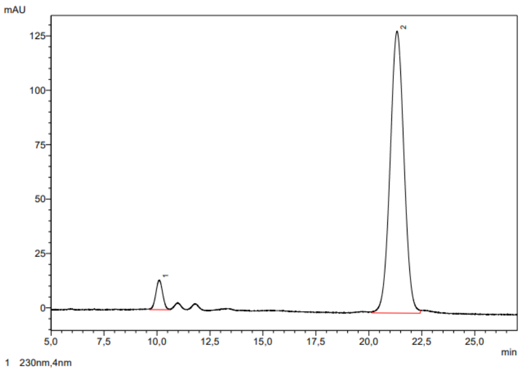

PDA Ch1 230nm

Peak #	Ret. Time	Area %	Name
1	10,11	4,92	1. Enantiomer
2	21,33	95,08	2. Enantiomer 90.2 % ee
Total		100,00	

***tert*-Butyl 2-((1*R*,2*R*,*E*)-2-((*tert*-butoxycarbonyl)amino)-1-hydroxy-6-methoxy-6-oxohex-4-en-1-yl)-1*H*-pyrrole-1-carboxylate (30).** The title compound was prepared according to the general procedure **A** and oxidative workup **I**, using N-Boc-pyrrole-2-carboxaldehyde (39.0 mg, 0.20 mmol) and diene **13a** (136.4 mg, 0.60 mmol); the reaction time was 48 h. The crude material (rr > 20:1) was purified by flash chromatography (SiO_2, toluene/*tert*-butyl methyl ether, 6:1) and the product was obtained as a viscous yellowish oil (65.1 mg, 77% yield, dr > 20:1, 91% ee). $[\alpha]_D^{20}$ (91% ee) = –7.2 (c = 0.50, $CHCl_3$); ^1H NMR (400 MHz, CD_2Cl_2): δ = 7.19 (s, 1H), 6.99 – 6.92 (m, 1H), 6.24 – 6.22 (m, 1H), 6.10 (t, *J* = 3.4 Hz, 1H), 5.89 (dt, *J* = 15.6, 1.5 Hz, 1H), 4.76 (t, *J* = 7.2 Hz, 1H), 4.62 (d, *J* = 9.9 Hz, 1H), 4.18 – 4.08 (m, 2H), 3.69 (s, 3H), 2.76 – 2.69 (m, 1H), 2.48 – 2.40 (m, 1H), 1.60 (s, 9H), 1.32 (s, 9H); ^{13}C NMR (101 MHz, CD_2Cl_2): δ = 166.9, 155.5, 150.9, 146.1, 135.1, 123.6, 122.9, 113.9, 110.5, 85.4, 79.5, 70.9, 52.9, 51.6, 34.5, 28.4 (3C), 28.1 (3C); IR (ATR): $\tilde{\nu}$ = 3370, 2977, 1710, 1493, 1327, 1248, 1161, 1125, 1042, 725 cm^{-1}; HRMS (ESI^+): *m/z*: calcd. for $C_{21}H_{32}N_2O_7Na$ $[M+Na]^+$: 447.21017, found: 447.21050.

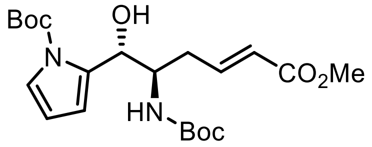

The ee was determined by HPLC analysis: Chiralpak 150 mm IC-3, 3 µm, 4.6 mm ∅, *n*-heptane/ethanol = 95:5, v = 1.0 mL/min, λ = 230 nm, t(minor) = 10.69 min, t(major) = 18.45 min (racemate: left, enantioenriched sample: right).

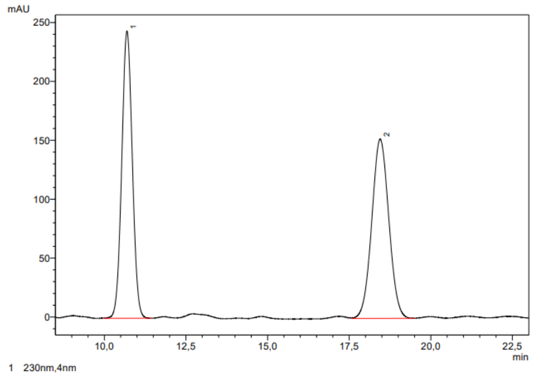

PDA Ch1 230nm

Peak #	Ret. Time	Area %	Name
1	10,69	49,78	1. Enantiomer
2	18,45	50,22	2. Enantiomer
Total		100,00	

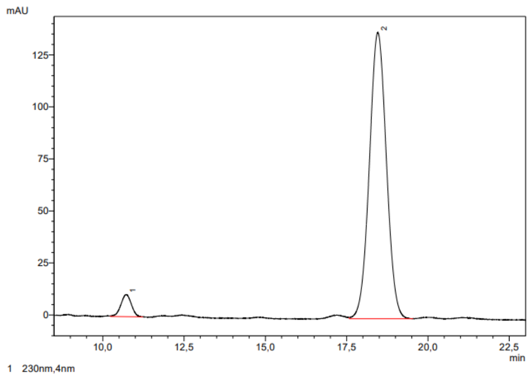

PDA Ch1 230nm

Peak #	Ret. Time	Area %	Name
1	10,72	4,56	1. Enantiomer
2	18,45	95,44	2. Enantiomer 90.9 % ee
Total		100,00	

Methyl (5*R*,6*R*,*E*)-5-((*tert*-butoxycarbonyl)amino)-6-(furan-2-yl)-6-hydroxyhex-2-enoate (31). The title compound was prepared according to the general procedure **A** and oxidative workup **I**, using furfural (16.6 µL, 0.20 mmol) and diene **13a** (90.9 mg, 0.40 mmol); the reaction time was 23 h. The crude material (rr = 13:1) was purified by flash chromatography (SiO_2, toluene/*tert*-butyl methyl ether, 4:1 → 2:1) and the product was obtained as an off-white solid (55.1 mg, 85% yield, dr > 20:1, 93% ee). mp = 98-100 °C; $[\alpha]_D^{20}$ (99% ee, sample after recrystallization) = +12.6 (c = 0.50, $CHCl_3$); ^1H NMR (400 MHz, $CDCl_3$): δ = 7.39 (dd, *J* = 1.8, 0.9 Hz, 1H), 6.93 – 6.85 (m, 1H), 6.35 (dd, *J* = 3.3, 1.8 Hz, 1H), 6.31 (dt, *J* = 3.3, 0.8 Hz, 1H), 5.84 (dt, *J* = 15.6, 1.4 Hz, 1H), 4.81 (br, 2H), 4.11 (s, 1H), 3.71 (s, 3H), 2.43 – 2.30 (m, 2H), 1.42 (s, 9H); ^{13}C NMR (101 MHz, $CDCl_3$): δ = 166.7, 156.4, 153.6, 144.9, 142.5, 123.7, 110.5, 107.9, 80.3, 70.5, 54.2, 51.6, 33.4, 28.4 (3C); IR (ATR): ṽ = 3356, 1720, 1682, 1252, 1164, 1007, 976, 736 cm^{-1}; HRMS (ESI$^+$): *m/z*: calcd. for $C_{16}H_{23}NO_6Na$ $[M+Na]^+$: 348.14176, found: 348.14190.

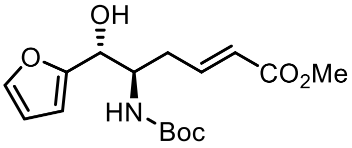

26.0 mg of the product were recrystallized from *tert*-butyl methyl ether/*iso*-hexane to give the enantioenriched product in 99% ee (20.8 mg).

The ee was determined by HPLC analysis: Chiralpak 150 mm IC-3, 3 µm, 4.6 mm ∅, *n*-heptane/ethanol = 90:10, v = 1.0 mL/min, λ = 220 nm, t(minor) = 6.19 min, t(major) = 22.26 min (racemate: top left, enantioenriched sample: top right, recrystallized enantioenriched sample: bottom left).

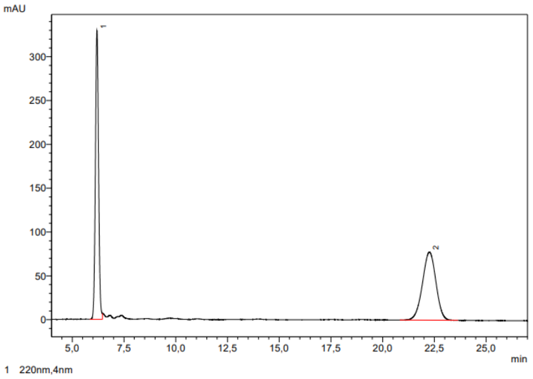

PDA Ch1 220nm

Peak #	Ret. Time	Area %	Name
1	6,19	49,89	1. Enantiomer
2	22,26	50,11	2. Enantiomer
Total		100,00	

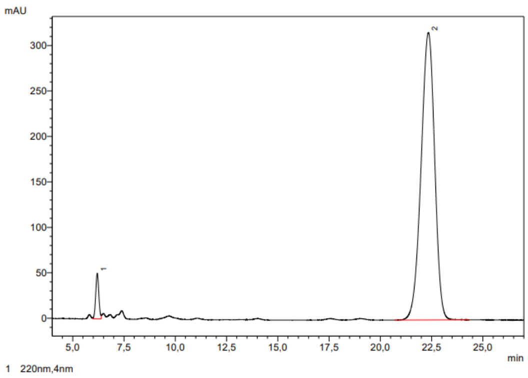

PDA Ch1 220nm

Peak #	Ret. Time	Area %	Name
1	6,20	3,52	1. Enantiomer
2	22,34	96,48	2. Enantiomer 93.0 % ee
Total		100,00	

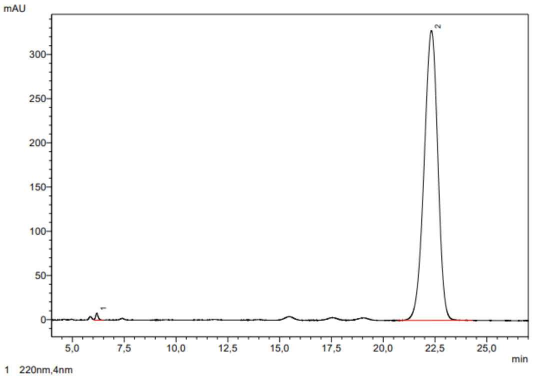

PDA Ch1 220nm

Peak #	Ret. Time	Area %	Name
1	6,18	0,46	1. Enantiomer
2	22,33	99,54	2. Enantiomer 99.1 % ee
Total		100,00	

Methyl (5*R*,6*R*,*E*)-5-((*tert*-butoxycarbonyl)amino)-6-hydroxy-6-(thiophen-2-yl)hex-2-enoate (32).

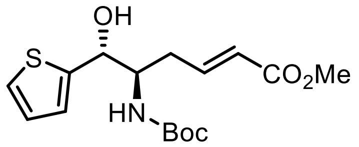

The title compound was prepared according to the general procedure **A** and oxidative workup **I**, using thiophene-2-carbaldehyde (18.7 µL, 0.20 mmol) and diene **13a** (136.4 mg, 0.60 mmol); the reaction time was 48 h. The crude material (rr = 10:1) was purified by flash chromatography (SiO_2, toluene/*tert*-butyl methyl ether, 4:1) and the product was obtained as a pale yellow solid (52.1 mg, 76% yield, dr > 20:1, 73% ee). mp = 106-108 °C; $[\alpha]_D^{20}$ (99% ee, sample after recrystallization) = +11.2 (c = 0.50, $CHCl_3$); ^{1}H NMR (400 MHz, CD_2Cl_2): δ = 7.28 (dd, *J* = 4.9, 1.3 Hz, 1H), 7.01 – 6.97 (m, 2H), 6.87 (ddd, *J* = 15.6, 7.9, 6.8 Hz, 1H), 5.83 (dt, *J* = 15.6, 1.5 Hz, 1H), 5.08 (s, 1H), 4.85 (d, *J* = 8.8 Hz, 1H), 4.00 (s, 1H), 3.83 (s, 1H), 3.68 (s, 3H), 2.45 – 2.39 (m, 1H), 2.34 – 2.26 (m, 1H), 1.41 (s, 9H); ^{13}C NMR (101 MHz, CD_2Cl_2): δ = 166.8, 156.8, 145.5, 145.0, 127.2, 125.1, 124.7, 123.7, 80.4, 73.4, 56.0, 51.7, 33.0, 28.4 (3C); IR (ATR): ṽ = 3358, 1691, 1650, 1526, 1322, 1227, 1166, 1048, 979, 639 cm^{-1}; HRMS (ESI$^+$): *m/z*: calcd. for $C_{16}H_{23}NO_5SNa$ [M+Na]$^+$: 364.11892, found: 364.11882.

16.0 mg of the product were recrystallized from *tert*-butyl methyl ether/*iso*-hexane to give the enantioenriched product in 99% ee (7.9 mg).

The ee was determined by HPLC analysis: Chiralpak 150 mm IG-3, 3 µm, 4.6 mm ∅, *n*-heptane/ethanol = 90:10, v = 1.0 mL/min, λ = 230 nm, t(minor) = 12.22 min, t(major) = 21.18 min (racemate: top left, enantioenriched sample: top right, recrystallized enantioenriched sample: bottom left).

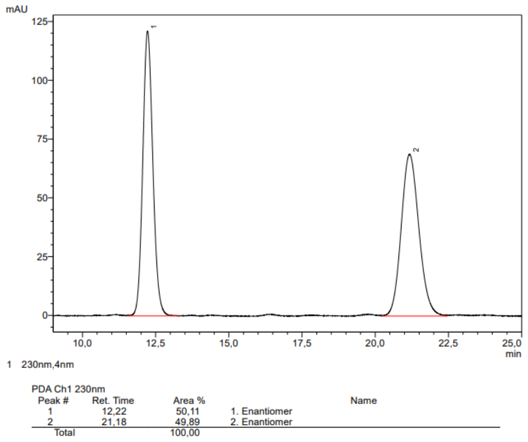

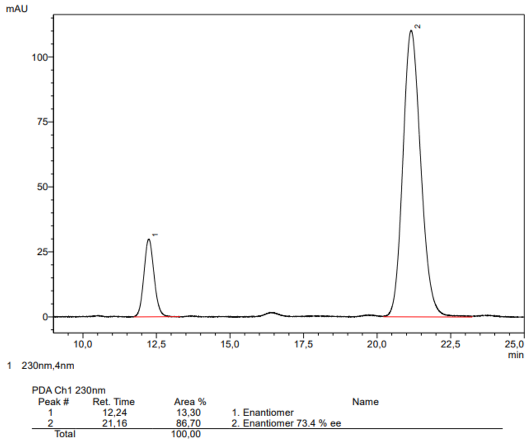

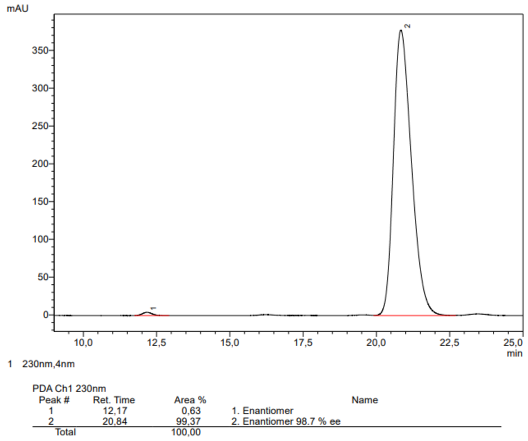

Methyl (5*R*,6*S*,*E*)-5-((*tert*-butoxycarbonyl)amino)-6-(4-cyanophenyl)-6-hydroxyhex-2-enoate (33).

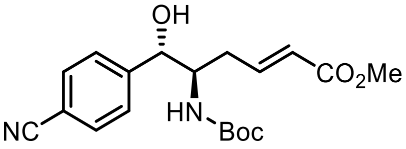

The title compound was prepared according to the general procedure **A** and oxidative workup **I**, using 4-cyanobenzaldehyde (26.2 mg, 0.20 mmol) and diene **13a** (90.9 mg, 0.40 mmol); the reaction time was 72 h. The crude material (rr = 8:1) was purified by flash chromatography (SiO_2, toluene/*tert*-butyl methyl ether, 3:1) and the product was obtained as an off-white solid (39.3 mg, 55% yield, dr > 20:1, 68% ee). mp = 110-113 °C; $[\alpha]_D^{20}$ (88% ee, sample after recrystallization) = +2.6 (c = 0.50, $CHCl_3$); ^{1}H NMR (400 MHz, CD_2Cl_2): δ = 7.68 – 7.64 (m, 2H), 7.52 – 7.49 (m, 2H), 6.84 – 6.77 (m, 1H), 5.80 (dt, *J* = 15.6, 1.5 Hz, 1H), 4.95 (s, 1H), 4.79 (d, *J* = 8.7 Hz, 1H), 3.93 (s, 1H), 3.67 (s, 3H), 3.50 (s, 1H), 2.35 – 2.22 (m, 2H), 1.40 (s, 9H); ^{13}C NMR (101 MHz, CD_2Cl_2): δ = 166.7, 156.5, 146.8, 145.1, 132.6 (2C), 127.5 (2C), 123.9, 119.1, 111.9, 80.5, 76.0, 56.0, 51.7, 32.0, 28.4 (3C); IR (ATR): ṽ = 3367, 2926, 2229, 1703, 1518, 1367, 1276, 1167, 1044 cm^{-1}; HRMS (ESI$^+$): *m/z*: calcd. for $C_{19}H_{24}N_2O_5Na$ $[M+Na]^+$: 383.15774, found: 383.15758.

35.0 mg of the product were recrystallized from *tert*-butyl methyl ether/*iso*-hexane to give the enantioenriched product in 88% ee (25.0 mg).

The ee was determined by HPLC analysis: Chiralpak 150 mm IB-N-3, 3 µm, 4.6 mm ∅, *n*-heptane/*iso*-propanol = 90:10, v = 1.0 mL/min, λ = 230 nm, t(major) = 7.74 min, t(minor) = 10.94 min (racemate: top left, enantioenriched sample: top right, recrystallized enantioenriched sample: bottom left).

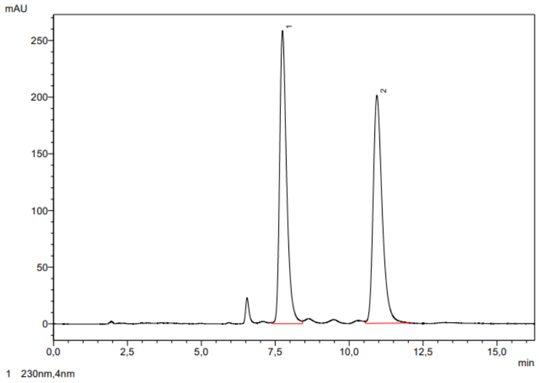

PDA Ch1 230nm

Peak #	Ret. Time	Area %	Name
1	7,74	50,02	1. Enantiomer
2	10,94	49,98	2. Enantiomer
Total		100,00	

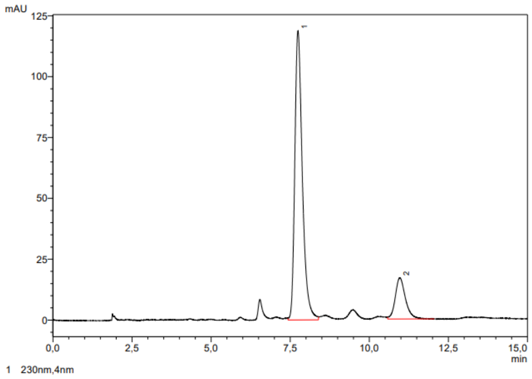

PDA Ch1 230nm

Peak #	Ret. Time	Area %	Name
1	7,75	83,84	1. Enantiomer 67.6 % ee
2	10,97	16,16	2. Enantiomer
Total		100,00	

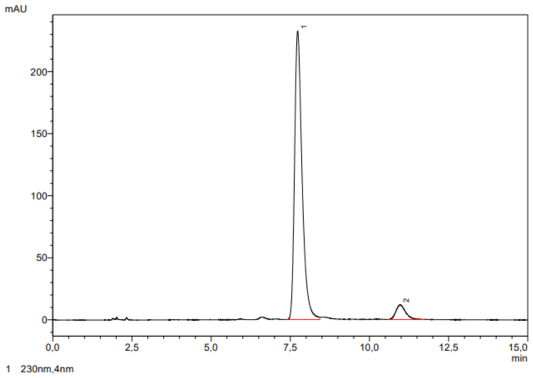

PDA Ch1 230nm

Peak #	Ret. Time	Area %	Name
1	7,73	93,85	1. Enantiomer 87.7 % ee
2	10,96	6,15	2. Enantiomer
Total		100,00	

Methyl (5*R*,6*S*,*E*)-5-((*tert*-butoxycarbonyl)amino)-6-hydroxy-8-phenyloct-2-enoate (34). The title compound was prepared according to the general procedure **B** and oxidative workup **I**, using hydrocinnamaldehyde (26.3 µL, 0.20 mmol) and diene **13a** (90.9 mg, 0.40 mmol). The crude material (rr = 4:1) was purified by flash chromatography (SiO_2, toluene/*tert*-butyl methyl ether, 4:1) and the product was obtained as an off-white solid (52.4 mg, 72% yield, dr > 20:1, 95% ee). mp = 122-123 °C; $[\alpha]_D^{20}$ (95% ee, sample before recrystallization) = –2.4 (c = 0.50, $CHCl_3$); ^1H NMR (400 MHz, CD_2Cl_2): δ = 7.30 – 7.26 (m, 2H), 7.22 – 7.16 (m, 3H), 6.95 – 6.88 (m, 1H), 5.86 (dt, *J* = 15.6, 1.5 Hz, 1H), 4.80 (d, *J* = 8.7 Hz, 1H), 3.74 – 3.63 (br, 2H), 3.69 (s, 3H), 2.88 – 2.81 (m, 1H), 2.69 – 2.61 (m, 1H), 2.55 (s, 1H), 2.49 – 2.43 (m, 1H), 2.38 – 2.30 (m, 1H), 1.82 – 1.70 (m, 2H), 1.40 (s, 9H); ^{13}C NMR (101 MHz, CD_2Cl_2): δ = 166.8, 156.4, 145.9, 142.3, 128.8 (4C), 126.3, 123.6, 80.0, 73.9, 54.8, 51.7, 35.8, 33.0, 32.6, 28.4 (3C); IR (ATR): ṽ = 3342, 1724, 1681, 1523, 1264, 1166, 1012, 752, 699 cm^{-1}; HRMS (ESI$^+$): *m/z*: calcd. for $C_{20}H_{29}NO_5Na$ $[M+Na]^+$: 386.19379, found: 386.19391.

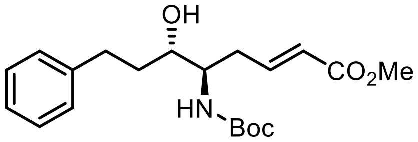

20.0 mg of the product were recrystallized from *tert*-butyl methyl ether/*iso*-hexane to give the enantioenriched product in >99% ee (15.2 mg).

The ee was determined by HPLC analysis: Chiralpak 150 mm IB-N-3, 3 µm, 4.6 mm ∅, CH_3CN/water = 35:65, v = 1.0 mL/min, λ = 210 nm, t(major) = 31.66 min, t(minor) = 39.17 min (racemate: top left, enantioenriched sample: top right, recrystallized enantioenriched sample: bottom left).

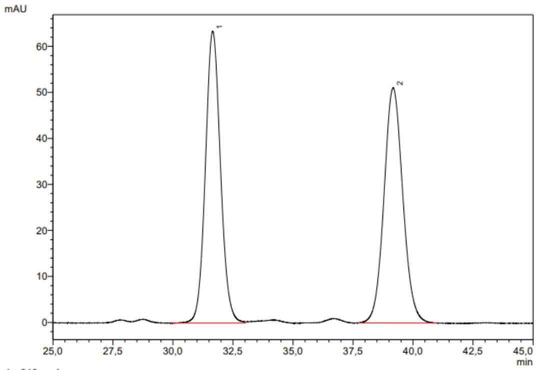

1 210nm,4nm

PDA Ch1 210nm

Peak #	Ret. Time	Area %	Name
1	31,66	49,75	1. Enantiomer
2	39,17	50,25	2. Enantiomer
Total		100,00	

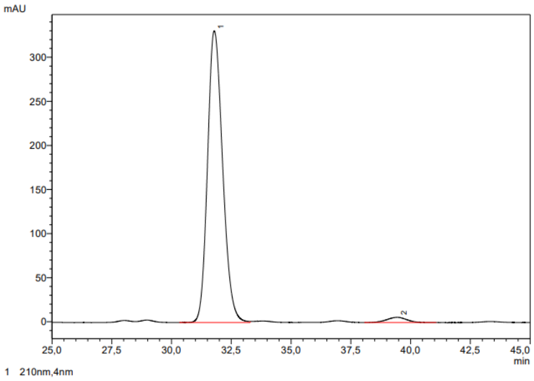

1 210nm,4nm

PDA Ch1 210nm

Peak #	Ret. Time	Area %	Name
1	31,79	97,67	1. Enantiomer 95.3 % ee
2	39,45	2,33	2. Enantiomer
Total		100,00	

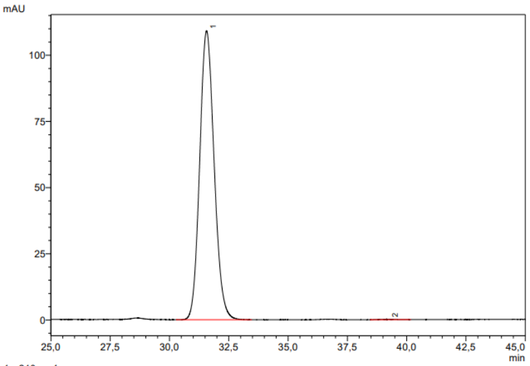

1 210nm,4nm

PDA Ch1 210nm

Peak #	Ret. Time	Area %	Name
1	31,56	99,89	1. Enantiomer 99.8 % ee
2	39,24	0,11	2. Enantiomer
Total		100,00	

Methyl (5*R*,6*S*,*E*)-5-((*tert*-butoxycarbonyl)amino)-6-hydroxytridec-2-enoate (36). The title compound was prepared according to the general procedure **B** and oxidative workup **I**, using octanal (31.2 µL, 0.20 mmol) and diene **13a** (90.9 mg, 0.40 mmol). The crude material (rr = 3:1) was purified by flash chromatography (SiO_2, toluene/*tert*-butyl methyl ether, 4:1) and the product was obtained as an off-white solid (28.6 mg, 40% yield, dr > 20:1, 94% ee). mp = 112-114 °C; $[\alpha]_D^{20}$ (99% ee, sample after recrystallization) = –4.4 (c = 0.50, $CHCl_3$); ^1H NMR (400 MHz, CD_2Cl_2): δ = 6.97 – 6.90 (m, 1H), 5.87 (dt, *J* = 15.6, 1.5 Hz, 1H), 4.80 (d, *J* = 8.8 Hz, 1H), 3.69 (s, 3H), 3.68 – 3.60 (m, 2H), 2.49 – 2.43 (m, 1H), 2.37 – 2.29 (m, 1H), 2.21 (br, 1H), 1.48 – 1.43 (m, 2H), 1.40 (s, 9H), 1.34 – 1.23 (m, 10H), 0.90 – 0.87 (m, 3H); ^{13}C NMR (101 MHz, CD_2Cl_2): δ = 166.8, 156.2, 146.2, 123.5, 79.8, 74.5, 54.5, 51.7, 34.1, 32.8, 32.2, 30.0, 29.6, 28.4 (3C), 26.3, 23.1, 14.3; IR (ATR): ṽ = 3349, 2923, 2854, 1725, 1680, 1526, 1169 cm^{-1}; HRMS (ESI$^+$): *m/z*: calcd. for $C_{19}H_{35}NO_5Na$ $[M+Na]^+$: 380.24074, found: 380.24087.

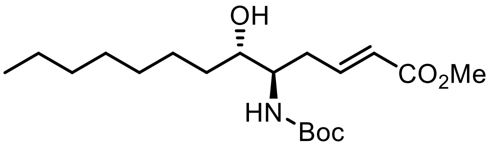

26.3 mg of the product were recrystallized from *tert*-butyl methyl ether/*iso*-hexane to give the enantioenriched product in 99% ee (15.6 mg).

The ee was determined by HPLC analysis: Chiralpak 150 mm IG-3, 3 µm, 4.6 mm ∅, *n*-heptane/ethanol = 95:5, v = 1.0 mL/min, λ = 210 nm, t(major) = 15.12 min, t(minor) = 20.06 min (racemate: top left, enantioenriched sample: top right, recrystallized enantioenriched sample: bottom left).

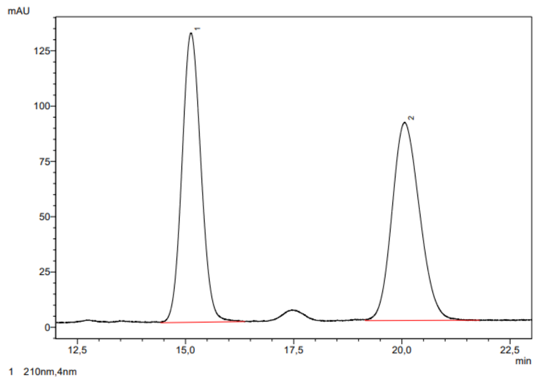

PDA Ch1 210nm

Peak #	Ret. Time	Area %	Name
1	15,12	50,09	1. Enantiomer
2	20,06	49,91	2. Enantiomer
Total		100,00	

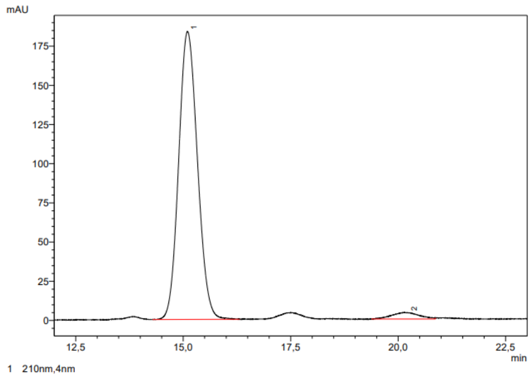

PDA Ch1 210nm

Peak #	Ret. Time	Area %	Name
1	15,10	96,73	1. Enantiomer 93.5 % ee
2	20,22	3,27	2. Enantiomer
Total		100,00	

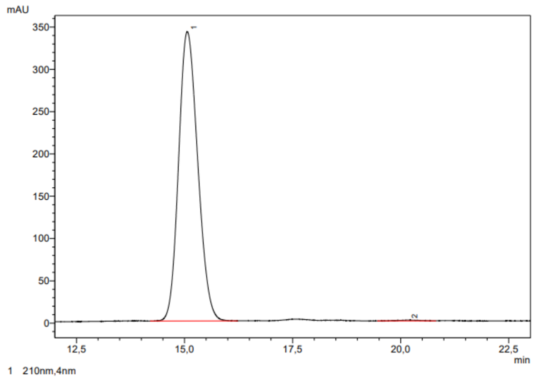

PDA Ch1 210nm

Peak #	Ret. Time	Area %	Name
1	15,06	99,62	1. Enantiomer 99.2 % ee
2	20,17	0,38	2. Enantiomer
Total		100,00	

Methyl (5*R*,6*S*,*E*)-5-((*tert*-butoxycarbonyl)amino)-6-hydroxyoct-2-enoate (37). The title compound was prepared according to the general procedure **B** and oxidative workup **I**, using propionaldehyde (14.4 µL, 0.20 mmol) and diene **13a** (90.9 mg, 0.40 mmol). The crude material (rr = 2:1) was purified by flash chromatography (SiO_2, toluene/*tert*-butyl methyl ether, 2:1) and the product was obtained as a colorless solid (30.5 mg, 53% yield, dr > 20:1, 97% ee). mp = 110-113 °C; $[\alpha]_D^{20}$ (97% ee) = –4.4 (c = 0.50, $CHCl_3$); ^1H NMR (400 MHz, CD_2Cl_2): δ = 6.97 – 6.90 (m, 1H), 5.87 (dt, *J* = 15.6, 1.5 Hz, 1H), 4.82 (d, *J* = 9.0 Hz, 1H), 3.71 – 3.64 (m, 1H), 3.69 (s, 3H), 3.58 – 3.52 (m, 1H), 2.50 – 2.43 (m, 1H), 2.38 – 2.29 (m, 2H), 1.57 – 1.43 (m, 2H), 1.40 (s, 9H), 0.97 (t, *J* = 7.4 Hz, 3H); ^{13}C NMR (101 MHz, CD_2Cl_2): δ = 166.9, 156.2, 146.3, 123.5, 79.8, 75.9, 54.3, 51.7, 32.8, 28.4 (3C), 27.1, 10.5; IR (ATR): ṽ = 3351, 2959, 1717, 1673, 1655, 1525, 1302, 1165, 1028, 971, 646 cm^{-1}; HRMS (ESI$^+$): *m/z*: calcd. for $C_{14}H_{25}NO_5Na$ $[M+Na]^+$: 310.16249 , found: 310.16243.

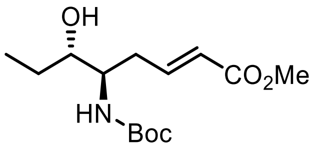

The ee was determined by HPLC analysis: Chiralpak 150 mm IG-3, 3 µm, 4.6 mm ∅, *n*-heptane/ethanol = 90:10, v = 1.0 mL/min, λ = 210 nm, t(minor) = 8.43 min, t(major) = 12.74 min (racemate: left, enantioenriched sample: right).

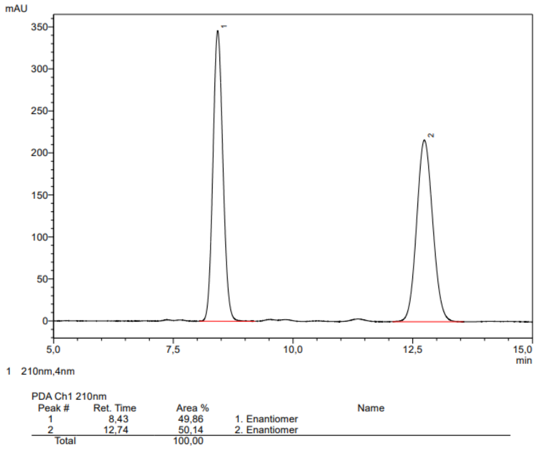

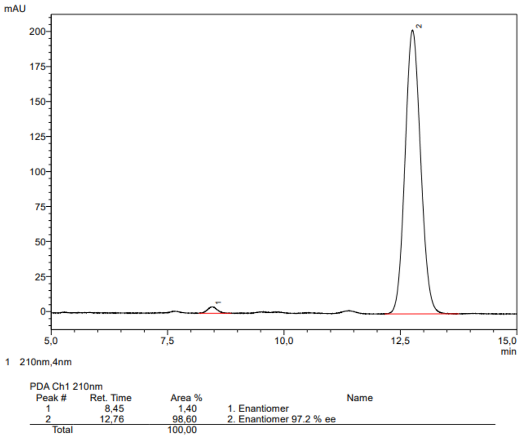

Methyl (5*R*,6*S*,*E*)-5-((*tert*-butoxycarbonyl)amino)-6-hydroxy-7-methyloct-2-enoate (38). The title compound was prepared according to the general procedure **B** and oxidative workup **I**, using *iso*-butyraldehyde (18.3 µL, 0.20 mmol) and diene **13a** (90.9 mg, 0.40 mmol). The crude material (rr = 3:1) was purified by flash chromatography (SiO_2, toluene/*tert*-butyl methyl ether, 3:1) and the product was obtained as an off-white solid (36.5 mg, 61% yield, dr > 20:1, 94% ee). $[\alpha]_D^{20}$ (94% ee) = –4.0 (c = 0.50, $CHCl_3$); ^1H NMR (400 MHz, CD_2Cl_2): δ = 7.00 – 6.92 (m, 1H), 5.87 (dt, *J* = 15.6, 1.5 Hz, 1H), 4.89 (d, *J* = 8.2 Hz, 1H), 3.85 – 3.73 (m, 1H), 3.68 (s, 3H), 3.31 – 3.26 (m, 1H), 2.51 – 2.45 (m, 1H), 2.39 – 2.30 (m, 1H), 2.18 (d, *J* = 5.4 Hz, 1H), 1.74 – 1.66 (m, 1H), 1.40 (s, 9H), 0.97 (d, *J* = 6.6 Hz, 3H), 0.93 (d, *J* = 6.7 Hz, 3H); ^{13}C NMR (101 MHz, CD_2Cl_2): δ = 166.9, 155.8, 146.6, 123.3, 79.6 (2C), 51.9, 51.6, 32.5, 31.1, 28.4 (3C), 19.2, 18.5; IR (ATR): ṽ = 3367, 2959, 1716, 1674, 1522, 1301, 1163, 987, 627 cm^{-1}; HRMS (ESI^+): *m/z*: calcd. for $C_{15}H_{27}NO_5Na$ $[M+Na]^+$: 324.17814, found: 324.17831.

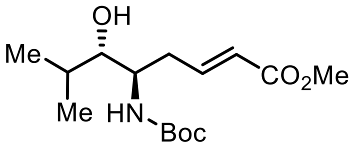

The ee was determined by HPLC analysis: Chiralpak 150 mm IC-3, 3 µm, 4.6 mm ∅, *n*-heptane/*iso*-propanol = 90:10, v = 1.0 mL/min, λ = 210 nm, t(minor) = 7.77 min, t(major) = 14.49 min (racemate: left, enantioenriched sample: right).

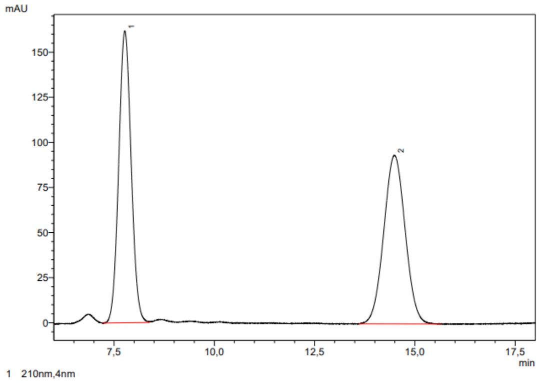

PDA Ch1 210nm

Peak #	Ret. Time	Area %	Name
1	7,77	50,17	1. Enantiomer
2	14,49	49,83	2. Enantiomer
Total		100,00	

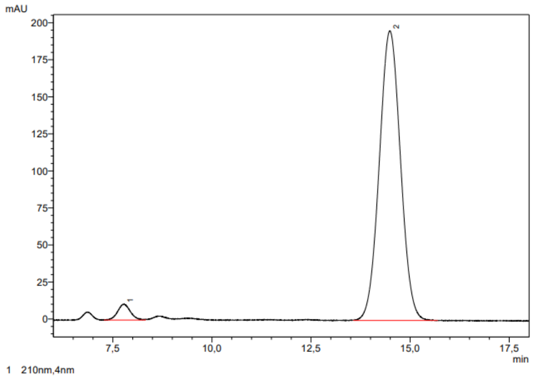

PDA Ch1 210nm

Peak #	Ret. Time	Area %	Name
1	7,77	3,23	1. Enantiomer
2	14,49	96,77	2. Enantiomer 93.5 % ee
Total		100,00	

Methyl (5*R*,6*S*,*E*)-5-((*tert*-butoxycarbonyl)amino)-6-cyclopropyl-6-hydroxyhex-2-enoate (39). The title compound was prepared according to the general procedure **B** and oxidative workup **I**, using cyclopropanecarboxaldehyde (14.9 µL, 0.20 mmol) and diene **13a** (90.9 mg, 0.40 mmol). The crude material (rr = 6:1) was purified by flash chromatography (SiO_2, toluene/*tert*-butyl methyl ether, 2.5:1) and the product was obtained as an off-white solid (41.8 mg, 70% yield, dr > 20:1, 94% ee). mp = 108-110 °C, $[\alpha]_D^{20}$ (94% ee) = +5.6 (c = 0.50, $CHCl_3$); ^1H NMR (400 MHz, CD_2Cl_2): δ = 7.00 – 6.92 (m, 1H), 5.88 (dt, *J* = 15.6, 1.5 Hz, 1H), 4.84 (br, 1H), 3.86 – 3.76 (m, 1H), 3.69 (s, 3H), 2.99 – 2.95 (m, 1H), 2.63 – 2.56 (m, 1H), 2.48 – 2.40 (m, 1H), 2.34 – 2.24 (m, 1H), 1.40 (s, 9H), 0.93 – 0.85 (m, 1H), 0.59 – 0.49 (m, 2H), 0.36 – 0.24 (m, 2H); ^{13}C NMR (101 MHz, CD_2Cl_2): δ = 166.9, 156.2, 146.3, 123.4, 79.7, 78.6, 54.8, 51.7, 33.2, 28.4 (3C), 14.6, 2.8, 2.6; IR (ATR): ṽ = 3357, 1724, 1677, 1525, 1311, 1165, 1009, 651 cm^{-1}; HRMS (ESI^+): *m/z*: calcd. for $C_{15}H_{25}NO_5Na$ $[M+Na]^+$: 322.16249, found: 322.16256.

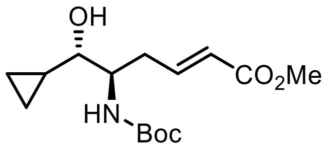

The ee was determined by HPLC analysis: Chiralpak 150 mm IG-3, 3 µm, 4.6 mm ∅, *n*-heptane/ethanol = 90:10, v = 1.0 mL/min, λ = 210 nm, t(minor) = 9.99 min, t(major) = 16.75 min (racemate: left, enantioenriched sample: right).

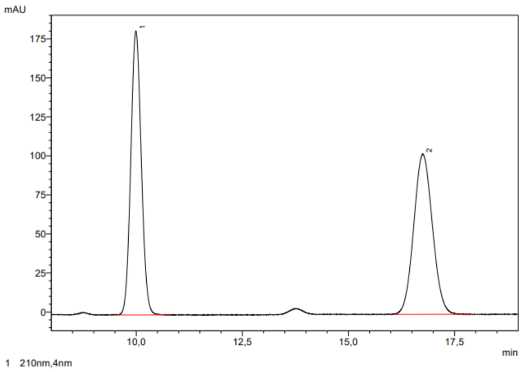

PDA Ch1 210nm

Peak #	Ret. Time	Area %	Name
1	9,99	49,89	1. Enantiomer
2	16,75	50,11	2. Enantiomer
Total		100,00	

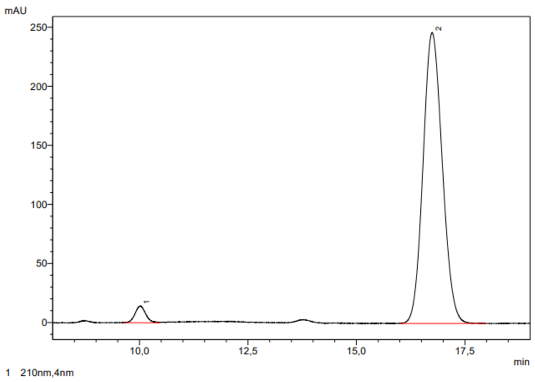

PDA Ch1 210nm

Peak #	Ret. Time	Area %	Name
1	10,02	3,10	1. Enantiomer
2	16,75	96,90	2. Enantiomer 93.8 % ee
Total		100,00	

Methyl (5*R*,6*S*,*E*)-5-((*tert*-butoxycarbonyl)amino)-6-cyclobutyl-6-hydroxyhex-2-enoate (40). The title compound was prepared according to the general procedure **B** and oxidative workup **I**, using cyclobutanecarbaldehyde (18.0 µL, 0.20 mmol) and diene **13a** (90.9 mg, 0.40 mmol). The crude material (rr = 5:1) was purified by flash chromatography (SiO_2, toluene/*tert*-butyl methyl ether, 2.5:1) and the product was obtained as an off-white solid (42.2 mg, 67% yield, dr > 20:1, 90% ee). mp = 139-140 °C, $[\alpha]_D^{20}$ (90% ee) = –8.4 (c = 0.50, $CHCl_3$); ^1H NMR (400 MHz, CD_2Cl_2): δ = 6.95 – 6.88 (m, 1H), 5.85 (dt, *J* = 15.6, 1.5 Hz, 1H), 4.80 (d, *J* = 8.9 Hz, 1H), 3.68 (s, 3H), 3.66 – 3.53 (m, 2H), 2.47 – 2.27 (m, 3H), 2.23 (s, 1H), 2.08 – 1.97 (m, 1H), 1.97 – 1.77 (m, 5H), 1.40 (s, 9H); ^{13}C NMR (101 MHz, CD_2Cl_2): δ = 166.9, 156.1, 146.4, 123.4, 79.7, 78.4, 52.7, 51.7, 38.7, 32.8, 28.4 (3C), 25.4, 24.9, 18.6; IR (ATR): ṽ = 3356, 2936, 1719, 1675, 1523, 1275, 1163, 985, 646 cm^{-1}; HRMS (ESI+): *m/z*: calcd. for $C_{16}H_{27}NO_5Na$ $[M+Na]^+$: 336.17814, found: 336.17816.

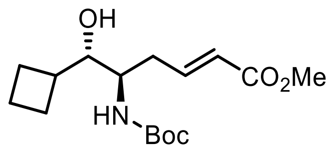

The ee was determined by 2D-HPLC analysis: First dimension = 50 mm RX-SIL, 4.6 mm ∅, *n*-heptane/*iso*-propanol = 99:1, v = 1.0 mL/min, λ = 220 nm; second Dimension = 150 mm Chiralpak IB-N-3, 4.6 mm ∅, *n*-heptane/*iso*-propanol = 98:2, v = 1.0 ml/min, λ = 220 nm.

First dimension racemic sample:

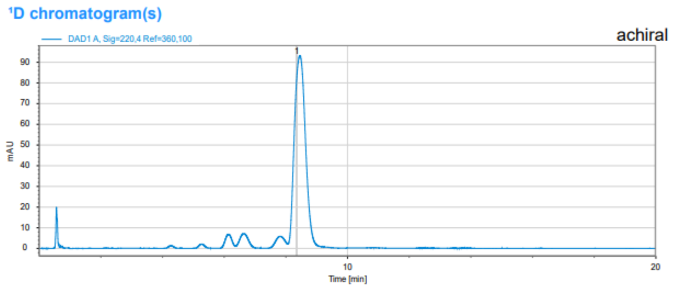

Sampling table (¹D)

Cut group	Cut #	¹D Cut start [min]	¹D Ret. time [min]	¹D Duration [min]	Trigger	²D Run start [min]
	1	8.34	***	0.04	Peak	8.40

Second dimension racemic sample:

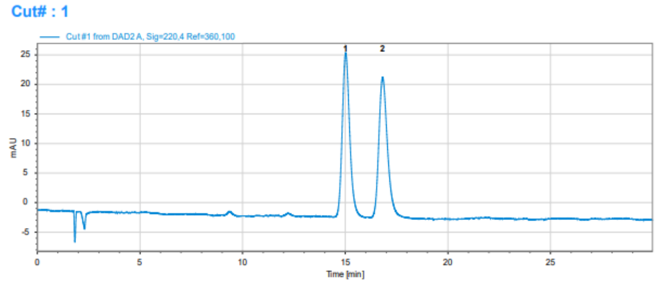

Signal: DAD2 A, Sig=220,4 Ref=360,100

Compound	Cut	Ret.Time	Area	Width	Height	Symmetry
1	1	15.026	700.164<br>**700.164**	0.409	27.532	0.818
2	1	16.828	695.305<br>**695.305**	0.470	23.730	0.758

Component table

Signal: DAD2 A, Sig=220,4 Ref=360,100

chiral

Component	¹D Sampling range [min]	Ret.Time ²D [min]	Area	Area%	
1	8.34 - 8.38	15.026	700.164	50.174	1. Enantiomer
2	8.34 - 8.38	16.828	695.305	49.826	2. Enantiomer

First dimension enantioenriched sample:

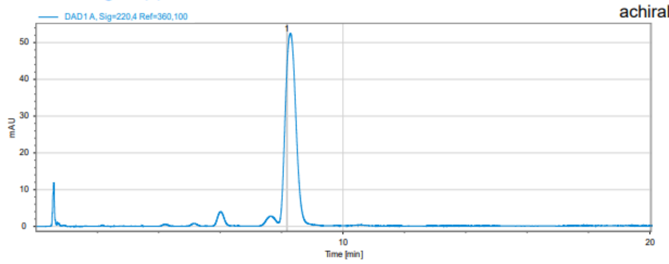

Sampling table (¹D)

Cut group	Cut #	¹D Cut start [min]	¹D Ret. time [min]	¹D Duration [min]	Trigger	²D Run start [min]
	1	8.16	***	0.04	Peak	8.22

Second dimension enantioenriched sample:

Cut# : 1

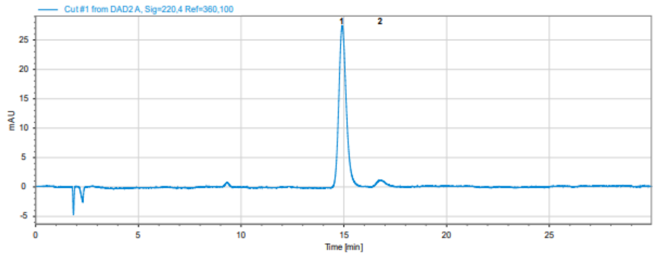

Signal: DAD2 A, Sig=220,4 Ref=360,100

Compound	Cut	Ret.Time	Area	Width	Height	Symmetry
1	1	14.920	693.516 **693.516**	0.358	27.573	0.788
2	1	16.793	35.371 **35.371**	0.369	1.147	0.717

Component table

chiral

Signal: DAD2 A, Sig=220,4 Ref=360,100

Component	¹D Sampling range [min]	Ret.Time ²D [min]	Area	Area%	
1	8.16 - 8.20	14.920	693.516	95.147	1. Enantiomer
2	8.16 - 8.20	16.793	35.371	4.853	2. Enantiomer

= 90.3 % ee

Methyl (5*R*,6*S*,*E*)-5-((*tert*-butoxycarbonyl)amino)-6-cyclohexyl-6-hydroxyhex-2-enoate (41). The title compound was prepared according to the general procedure **B** and oxidative workup **I**, using cyclohexanecarbaldehyde (24.2 µL, 0.20 mmol) and diene **13a** (90.9 mg, 0.40 mmol). The crude material (rr = 5:1) was purified by flash chromatography (SiO_2, toluene/*tert*-butyl methyl ether, 4:1) and the product was obtained as an off-white solid (44.3 mg, 65% yield, dr > 20:1, 88% ee). mp = 96-97 °C; $[\alpha]_D^{20}$ (98% ee, sample after recrystallization) = –11.0 (c = 0.50, $CHCl_3$); ^1H NMR (400 MHz, CD_2Cl_2): δ = 6.99 – 6.92 (m, 1H), 5.87 (dt, *J* = 15.6, 1.5 Hz, 1H), 4.91 (d, *J* = 9.4 Hz, 1H), 3.85 – 3.75 (m, 1H), 3.68 (s, 3H), 3.35 – 3.31 (m, 1H), 2.49 – 2.42 (m, 1H), 2.37 – 2.29 (m, 1H), 2.13 (d, *J* = 5.3 Hz, 1H), 1.96 – 1.92 (m, 1H), 1.79 – 1.73 (m, 2H), 1.66 – 1.55 (m, 2H), 1.40 (s, 9H), 1.28 – 0,92 (m, 6H); ^{13}C NMR (101 MHz, CD_2Cl_2): δ = 166.9, 155.8, 146.7, 123.3, 79.5, 78.7, 51.6, 51.4, 40.8, 32.3, 29.5, 29.2, 28.4 (3C), 26.7, 26.4, 26.3; IR (ATR): ṽ = 3360, 2921, 1725, 1680, 1524, 1252, 1164, 1005, 974, 651 cm^{-1}; HRMS (ESI^+): *m/z*: calcd. for $C_{18}H_{31}NO_5Na$ $[M+Na]^+$: 364.20944, found: 364.20946.

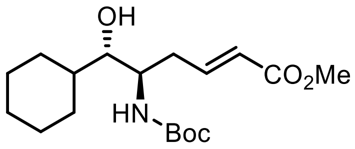

36.3 mg of the product were recrystallized from *tert*-butyl methyl ether/*iso*-hexane to give the enantioenriched product in 98% ee (24.4 mg).

The ee was determined by HPLC analysis: Chiralpak 150 mm IG-3, 3 µm, 4.6 mm ∅, CH_3CN/water = 40:60, v = 1.0 mL/min, λ = 210 nm, t(major) = 18.01 min, t(minor) = 20.67 min (racemate: top left, enantioenriched sample: top rigt, recrystallized enantioenriched sample: bottom left).

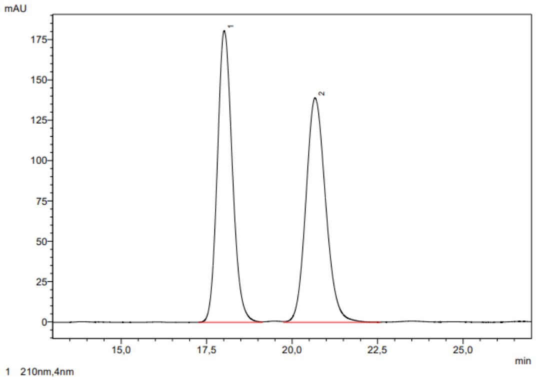

PDA Ch1 210nm

Peak #	Ret. Time	Area %	Name
1	18,01	49,61	1. Enantiomer
2	20,67	50,39	2. Enantiomer
Total		100,00	

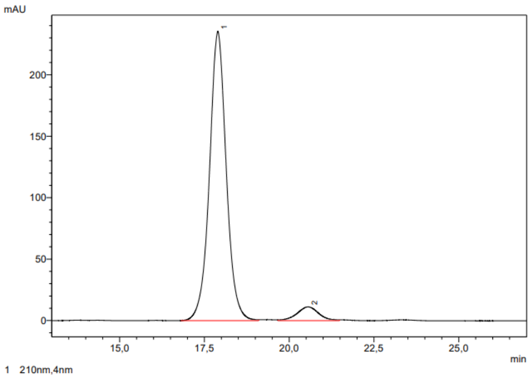

PDA Ch1 210nm

Peak #	Ret. Time	Area %	Name
1	17,90	94,16	1. Enantiomer 88.3 % ee
2	20,55	5,84	2. Enantiomer
Total		100,00	

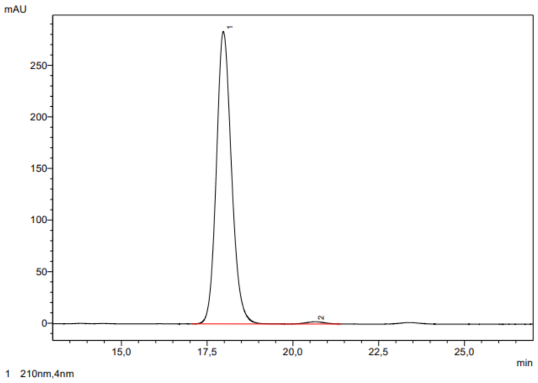

PDA Ch1 210nm

Peak #	Ret. Time	Area %	Name
1	17,97	99,06	1. Enantiomer 98.1 % ee
2	20,63	0,94	2. Enantiomer
Total		100,00	

Methyl (5*R*,6*S*,*E*)-5-((*tert*-butoxycarbonyl)amino)-6-hydroxy-6-(tetrahydro-2*H*-pyran-4-yl)hex-2-enoate (42). The title compound was prepared according to the general procedure **B** and oxidative workup **I**, using tetrahydropyran-4-carbaldehyde (21.7 µL, 0.20 mmol) and diene **13a** (90.9 mg, 0.40 mmol). The crude material (rr = 4:1) was purified by flash chromatography (SiO_2, toluene/*tert*-butyl methyl ether, 1:2) and the product was obtained as a colorless, sticky oil (44.1 mg, 64% yield, dr > 20:1, 87% ee). $[\alpha]_D^{20}$ (87% ee) = –5.4 (c = 0.53, $CHCl_3$); ^1H NMR (400 MHz, CD_2Cl_2): δ = 6.99 – 6.91 (m, 1H), 5.88 (dt, *J* = 15.6, 1.5 Hz, 1H), 4.89 (d, *J* = 9.3 Hz, 1H), 3.99 – 3.91 (m, 2H), 3.83 – 3.73 (m, 1H), 3.69 (s, 3H), 3.40 – 3.30 (m, 3H), 2.50 – 2.43 (m, 1H), 2.42 – 2.29 (m, 2H), 1.83 – 1.77 (m, 1H), 1.65 – 1.59 (m, 1H, overlaps with H_2O signal), 1.46 – 1.42 (m, 2H), 1.40 (s, 9H), 1.36 – 1.32 (m, 1H); ^{13}C NMR (101 MHz, CD_2Cl_2): δ = 166.9, 155.9, 146.2, 123.5, 79.8, 78.1, 68.1, 67.7, 51.7, 51.3, 38.2, 32.3, 29.5, 29.2, 28.4 (3C); IR (ATR): ṽ = 3368, 2926, 1705, 1700, 1517, 1366, 1165, 1091, 710 cm^{-1}; HRMS (ESI$^+$): *m/z*: calcd. for $C_{17}H_{29}NO_6Na$ $[M+Na]^+$: 366.18871, found: 366.18879.

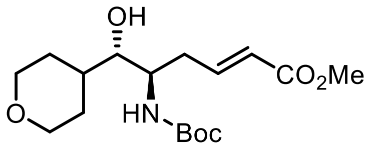

The ee was determined by HPLC analysis: Chiralpak 150 mm IB-N-3, 3 µm, 4.6 mm ∅, *n*-heptane/ethanol = 95:5, v = 1.0 mL/min, λ = 210 nm, t(major) = 22.99 min, t(minor) = 24.82 min (racemate: left, enantioenriched sample: right).

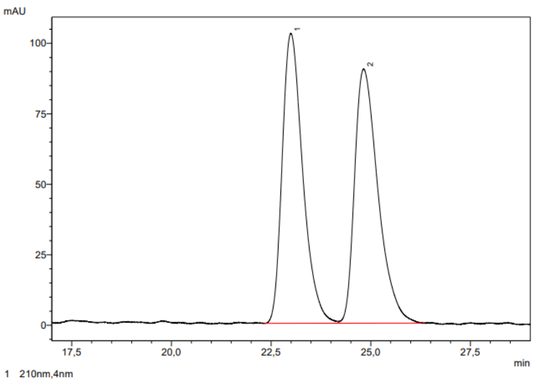

PDA Ch1 210nm

Peak #	Ret. Time	Area %	Name
1	22,99	50,06	1. Enantiomer
2	24,82	49,94	2. Enantiomer
Total		100,00	

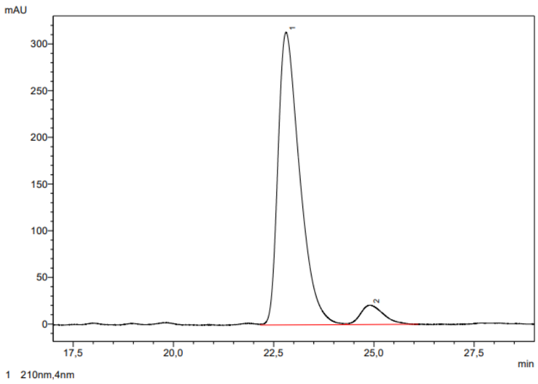

PDA Ch1 210nm

Peak #	Ret. Time	Area %	Name
1	22,81	93,41	1. Enantiomer 86.8 % ee
2	24,90	6,59	2. Enantiomer
Total		100,00	

Methyl (5*R*,6*R*,*E*)-5-((*tert*-butoxycarbonyl)amino)-7-((*tert*-butyldiphenylsilyl)oxy)-6-hydroxyhept-2-enoate (43a). The title compound was prepared according to the general procedure **B** and oxidative workup **I**, using 2-((*tert*-butyldiphenylsilyl)oxy)acetaldehyde (59.7 mg, 0.20 mmol)[1] and diene **13a** (90.9 mg, 0.40 mmol). The crude material (rr = 1.5:1) was purified by flash chromatography (SiO_2, toluene/*tert*-butyl methyl ether, 8:1 → 6:1) and the product was obtained as a colorless sticky oil (56.2 mg, 53% yield, dr > 20:1, 97% ee). $[\alpha]_D^{20}$ (97% ee) = +4.2 (c = 0.50, $CHCl_3$); ^1H NMR (400 MHz, CD_2Cl_2): δ = 7.68 – 7.64 (m, 4H), 7.48 – 7.39 (m, 6H), 6.94 – 6.87 (m, 1H), 5.84 (dt, *J* = 15.6, 1.5 Hz, 1H), 4.89 (d, *J* = 9.4 Hz, 1H), 3.86 – 3.76 (m, 1H), 3.72 – 3.66 (m, 3H), 3.68 (s, 3H), 2.82 (s, 1H), 2.51 – 2.35 (m, 2H), 1.37 (s, 9H), 1.07 (s, 9H); ^{13}C NMR (101 MHz, CD_2Cl_2): δ = 166.8, 156.0, 145.7, 136.0 (2C), 135.9 (2C), 133.3, 133.2, 130.3 (2C), 128.3 (4C), 123.7, 79.7, 73.5, 65.7, 52.4, 51.7, 34.2, 28.4 (3C), 27.0 (3C), 19.4; IR (ATR): $\tilde{\nu}$ = 3367, 2930, 1709, 1503, 1166, 1111, 701 cm^{-1}; HRMS (ESI^+): *m/z*: calcd. for $C_{29}H_{41}NO_6SiNa$ $[M+Na]^+$: 550.25954 , found: 550.25932.

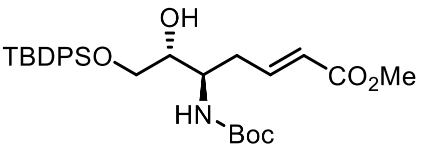

The ee was determined by HPLC analysis: Chiralpak 150 mm IB-N-3, 3 µm, 4.6 mm ∅, *n*-heptane/*iso*-propanol = 98:2, v = 1.0 mL/min, λ = 220 nm, t(minor) = 14.18 min, t(major) = 16.26 min (racemate: left, enantioenriched sample: right).

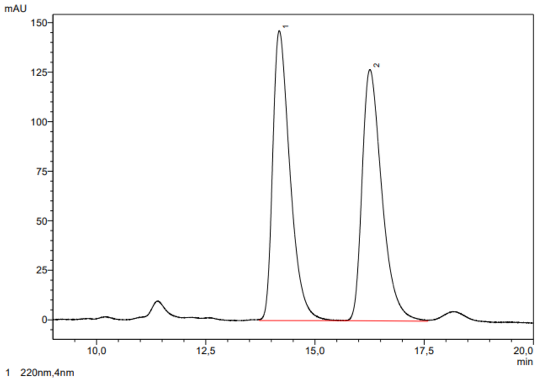

PDA Ch1 220nm

Peak #	Ret. Time	Area %	Name
1	14,18	50,14	1. Enantiomer
2	16,26	49,86	2. Enantiomer
Total		100,00	

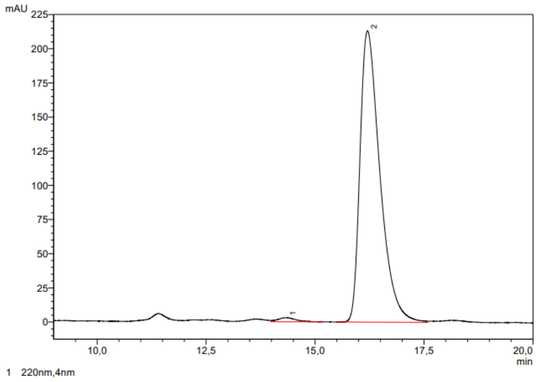

PDA Ch1 220nm

Peak #	Ret. Time	Area %	Name
1	14,34	1,28	1. Enantiomer
2	16,20	98,72	2. Enantiomer 97.4 % ee
Total		100,00	

Benzyl (5*R*,6*R*,*E*)-5-((*tert*-butoxycarbonyl)amino)-7-((*tert*-butyldiphenylsilyl)oxy)-6-hydroxyhept-2-enoate (43b). The title compound was prepared according to the general procedure **B** and oxidative workup **I**, using 2-((*tert*-butyldiphenylsilyl)oxy)acetaldehyde (20.9 mg, 70.0 µmol)[1] and the benzyl ester derivative **S3** (42.5 mg, 140 µmol). The crude material (rr = 2:1) was purified by flash chromatography (SiO_2, toluene/*tert*-butyl methyl ether, 8:1 → 7:1) and the product was obtained as a pale yellow sticky oil (24.6 mg, 58% yield, dr > 20:1, 97% ee). $[\alpha]_D^{20}$ (97% ee) = –10.4 (c = 0.25, $CHCl_3$); ^1H NMR (400 MHz, CD_2Cl_2): δ = 7.68 – 7.65 (m, 4H), 7.47 – 7.30 (m, 11H), 6.95 (dt, *J* = 15.2, 7.4 Hz, 1H), 5.89 (dt, *J* = 15.6, 1.5 Hz, 1H), 5.15 (s, 2H), 4.89 (d, *J* = 9.2 Hz, 1H), 3.85 – 3.75 (m, 1H), 3.73 – 3.65 (m, 3H), 2.81 (br, 1H), 2.52 – 2.37 (m, 2H), 1.35 (s, 9H), 1.07 (s, 9H); ^{13}C NMR (101 MHz, CD_2Cl_2): δ = 166.1, 156.0, 146.1, 136.8, 135.97 (2C), 135.95 (2C), 133.3, 133.2, 130.3 (2C), 128.9 (2C), 128.5 (2C), 128.4, 128.3 (4C), 123.7, 79.7, 73.5, 66.4, 65.7, 52.4, 34.3, 28.4 (3C), 27.0 (3C), 19.4; IR (ATR): ṽ = 3400, 2930, 1711, 1499, 1365, 1250, 1165, 1111, 1009, 739, 699 cm^{-1}; HRMS (ESI^+): *m/z*: calcd. for $C_{35}H_{45}NO_6SiNa$ $[M+Na]^+$: 626.29084, found: 626.29075.

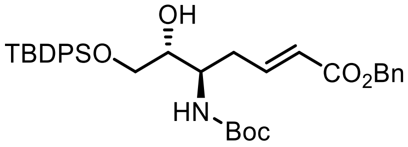

The ee was determined by HPLC analysis: Chiralpak 150 mm IB-N-3, 3 µm, 4.6 mm ∅, *n*-heptane/ethanol = 98:2, v = 1.0 mL/min, λ = 210 nm, t(minor) = 11.30 min, t(major) = 12.55 min (racemate: left, enantioenriched sample: right).

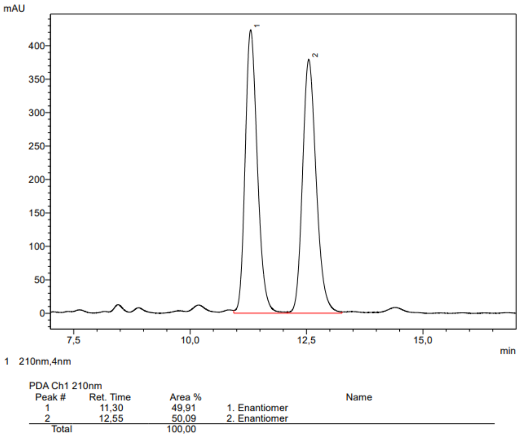

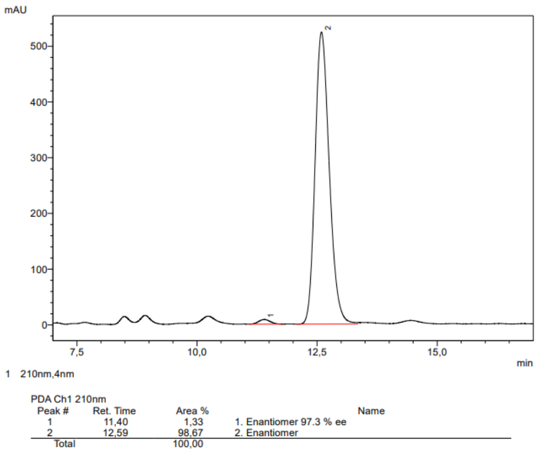

Methyl (5*R*,6*R*,*E*)-5-((*tert*-butoxycarbonyl)amino)-6-hydroxy-7-(4-methoxyphenoxy)hept-2-enoate (44). The title compound was prepared according to the general procedure **B** and oxidative workup **I**, using 2-(4-methoxyphenoxy)-acetaldehyde (33.2 mg, 0.20 mmol)[2] and diene **13a** (90.9 mg, 0.40 mmol). The crude material (rr = 2:1) was purified by flash chromatography (SiO_2, toluene/*tert*-butyl methyl ether, 4:1 → 3:1) and the product was obtained as an off-white solid (40.2 mg, 51% yield, dr > 20:1, 96% ee). mp = 110-112 °C; $[\alpha]_D^{20}$ (96% ee) = –15.8 (c = 0.50, $CHCl_3$); ^1H NMR (400 MHz, CD_2Cl_2): δ = 6.99 – 6.91 (m, 1H), 6.87 – 6.81 (m, 4H), 5.90 (dt, *J* = 15.6, 1.5 Hz, 1H), 4.89 (d, *J* = 9.1 Hz, 1H), 4.01 – 3.84 (m, 4H), 3.75 (s, 3H), 3.69 (s, 3H), 3.06 (s, 1H), 2.62 – 2.55 (m, 1H), 2.52 – 2.44 (m, 1H), 1.41 (s, 9H); ^{13}C NMR (101 MHz, CD_2Cl_2): δ = 166.8, 156.2, 154.7, 152.9, 145.5, 123.9, 115.9 (2C), 115.0 (2C), 80.1, 72.4, 70.7, 56.0, 52.4, 51.7, 33.8, 28.4 (3C); IR (ATR): ṽ = 3370, 2928, 1710, 1509, 1231, 1169, 1041, 826 cm^{-1}; HRMS (ESI^+): *m/z*: calcd. for $C_{20}H_{29}NO_7Na$ $[M+Na]^+$: 418.18362 , found: 418.18376.

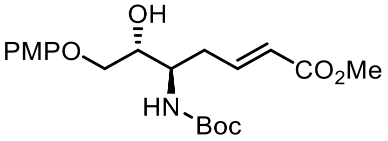

The ee was determined by HPLC analysis: Chiralpak 150 mm IG-3, 3 µm, 4.6 mm ∅, *n*-heptane/*iso*-propanol = 85:15, v = 1.0 mL/min, λ = 225 nm, t(major) = 15.45 min, t(minor) = 20.95 min (racemate: left, enantioenriched sample: right).

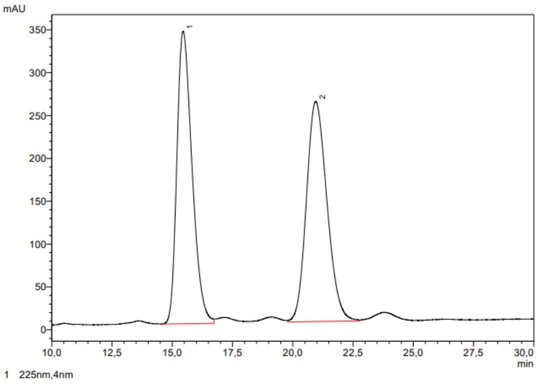

PDA Ch1 225nm

Peak #	Ret. Time	Area %	Name
1	15,45	49,52	1. Enantiomer
2	20,95	50,48	2. Enantiomer
Total		100,00	

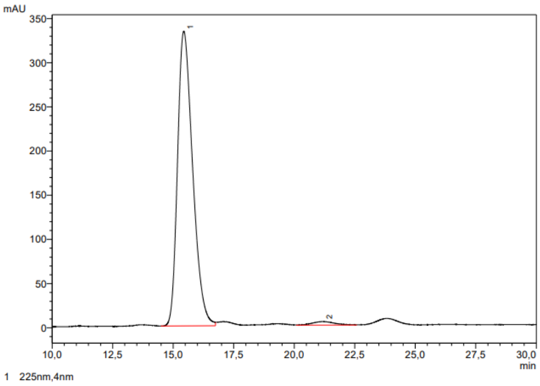

PDA Ch1 225nm

Peak #	Ret. Time	Area %	Name
1	15,44	98,19	1. Enantiomer 96.4 % ee
2	21,18	1,81	2. Enantiomer
Total		100,00	

Methyl (5*R*,6*S*,8*R*,*E*)-5-((*tert*-butoxycarbonyl)amino)-6-hydroxy-8,12-dimethyltrideca-2,11-dienoate

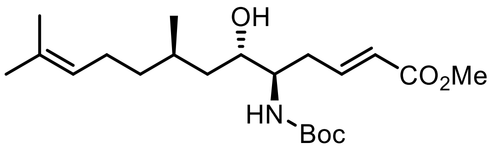

(45). The title compound was prepared according to the general procedure **B** and oxidative workup **I**, using (*R*)-citronellal (36.3 µL, 0.20 mmol) and diene **13a** (90.9 mg, 0.40 mmol). The crude material (rr = 2:1, dr = 11:1) was purified by flash chromatography (SiO_2, toluene/*tert*-butyl methyl ether, 4:1) to give the product as a colorless solid (41.0 mg, 53% yield, dr = 11:1). The product was carefully washed with cold pentane (2 x 1 mL) to obtain the product in an improved diastereomeric ratio (32.2 mg, 42% yield, dr = 20:1). mp = 101 – 103 °C; $[\alpha]_D^{20}$ = –13.4 (c = 0.50, $CHCl_3$); ^1H NMR (400 MHz, CD_2Cl_2): δ = 6.97 – 6.89 (m, 1H), 5.87 (dt, *J* = 15.6, 1.4 Hz, 1H), 5.13 – 5.08 (m, 1H), 4.78 (d, *J* = 8.9 Hz, 1H), 3.77 – 3.71 (m, 1H), 3.69 (s, 3H), 3.66 – 3.62 (br, 1H), 2.49 – 2.43 (m, 1H), 2.37 – 2.29 (m, 1H), 2.24 (s, 1H), 2.07 – 1.92 (m, 2H), 1.68 (d, *J* = 1.4 Hz, 3H), 1.60 (s, 3H), 1.48 – 1.44 (m, 1H), 1.41 (s, 9H), 1.36 – 1.12 (m, 4H), 0.90 (d, *J* = 6.5 Hz, 3H); ^{13}C NMR (101 MHz, CD_2Cl_2): δ = 166.8, 156.3, 146.1, 131.6, 125.0, 123.5, 79.8, 72.1, 55.2, 51.7, 41.1, 38.3, 33.1, 29.3, 28.4 (3C), 25.8 (2C), 19.1, 17.8; IR (ATR): ṽ = 3370, 2920, 1691, 1509, 1437, 1366, 1249, 1166, 1042 cm^{-1}; HRMS (ESI$^+$): *m/z*: calcd. for $C_{21}H_{37}NO_5Na$ $[M+Na]^+$: 406.25639, found: 406.25645.

Methyl (5*S*,6*S*,*E*)-5-((*tert*-butoxycarbonyl)amino)-6-hydroxy-6-phenylhex-2-enoate (50). A flame-dried Schlenk flask under argon was charged with tris(*trans*-1,2-bis(4-*tert*-butylphenyl)ethene)nickel(0) (Ni(^{t}Bu-stb)$_3$, **16**) (18.7 mg, 0.02 mmol) and phosphoramidite **L1** (12.8 mg, 0.02 mmol). The Schlenk flask was evacuated and refilled with argon (3 cycles). A solution of diene ***(E,Z)*-13a** (90.9 mg, 0.40 mmol) in THF (0.35 mL) and triethylborane (1 M in THF, 0.30 mL, 0.30 mmol) were successively added and the resulting mixture was stirred for 5 min. Benzaldehyde (20.2 µL, 0.20 mmol) was added under an argon counterflow, the flask was sealed and the mixture stirred at room temperature for 48 h. The reaction was quenched at 0 °C by addition of pH 7 phosphate buffer solution (1.5 mL) and aq. H_2O_2 (30% *w/w* in water, 0.3 mL). Ethyl acetate (2.0 mL) was added and the resulting mixture was vigorously stirred for 1 h. The mixture was diluted with ethyl acetate (10 mL) and water (5 mL) and the aqueous phase was extracted with ethyl acetate (2 x 10 mL). The combined organic layers were washed with sat. aq. $Na_2S_2O_3$ solution (15 mL) and brine (15 mL), dried over $MgSO_4$, filtered, and the solvent was removed under reduced pressure. The crude material (rr > 20:1) was purified by flash chromatography (SiO_2, toluene/*tert*-butyl methyl ether, 3:1) and the product was obtained as a colorless solid (62.6 mg, 93% yield, dr > 20:1, 40% ee). mp = 85-87 °C; $[\alpha]_D^{20}$ (98% ee, sample after recrystallization) = + 7.1 (c = 0.56, $CHCl_3$); ^{1}H NMR (400 MHz, CD_2Cl_2): δ = 7.29 – 7.17 (m, 5H), 6.83 (dt, *J* = 15.1, 7.4 Hz, 1H), 5.78 (dt, *J* = 15.6, 1.5 Hz, 1H), 4.76 (d, *J* = 7.2 Hz, 1H), 4.62 (t, *J* = 4.1 Hz, 1H), 3.79 – 3.72 (m, 1H), 3.60 (s, 3H), 3.03 (s, 1H), 2.47 – 2.40 (m, 1H), 2.32 – 2.24 (m, 1H), 1.25 (s, 9H); ^{13}C NMR (101 MHz, CD_2Cl_2): δ = 166.9, 156.4, 145.6, 142.1, 128.7 (2C), 128.1, 126.7 (2C), 123.7, 79.9, 75.7, 56.0, 51.7, 35.3, 28.4 (3C); IR (ATR): ṽ = 3402, 2976, 1712, 1673, 1513, 1356, 1221, 1166, 1107, 1043, 696 cm^{-1}; HRMS (ESI$^+$): *m/z*: calcd. for $C_{18}H_{25}NO_5Na$ $[M+Na]^+$: 358.16249, found: 358.16250.

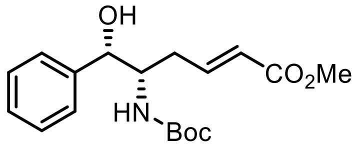

55.0 mg of the product were recrystallized from *tert*-butyl methyl ether/*iso*-hexane to yield the enantioenriched product from the mother liquor in 98% ee (25.2 mg).

The ee was determined by HPLC analysis: Chiralpak 150 mm IG-3, 3 µm, 4.6 mm ∅, *n*-heptane/*iso*-propanol = 90:10, v = 1.0 mL/min, λ = 210 nm, t(minor) = 16.11 min, t(major) = 19.80 min (racemate: top left, enantioenriched sample: top right, recrystallized enantioenriched sample: bottom left).

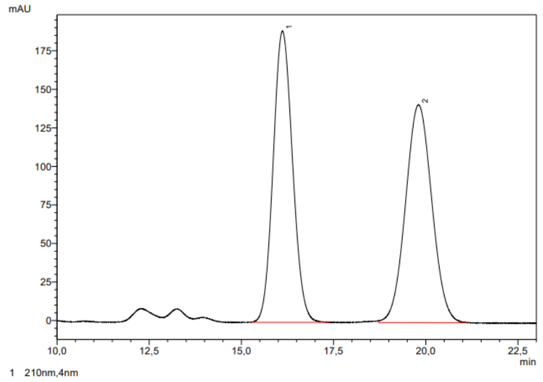

PDA Ch1 210nm

Peak #	Ret. Time	Area %	Name
1	16,11	49,62	1. Enantiomer
2	19,80	50,38	2. Enantiomer
Total		100,00	

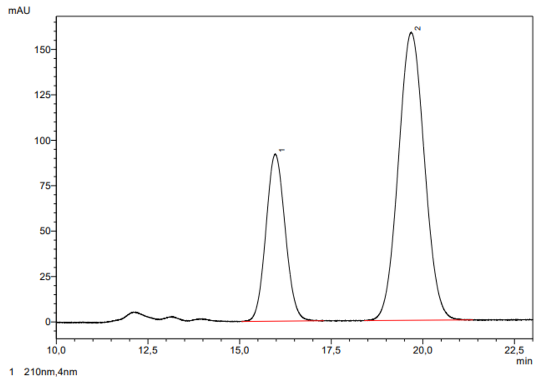

PDA Ch1 210nm

Peak #	Ret. Time	Area %	Name
1	15,97	29,98	1. Enantiomer
2	19,68	70,02	2. Enantiomer 40.0 % ee
Total		100,00	

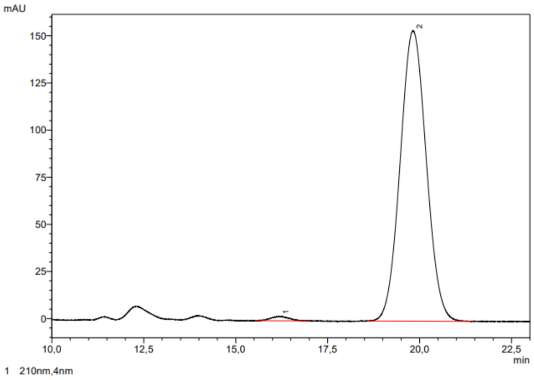

PDA Ch1 210nm

Peak #	Ret. Time	Area %	Name
1	16,18	1,05	1. Enantiomer
2	19,82	98,95	2. Enantiomer 97.9 % ee
Total		100,00	

Mosher Ester Analysis. Methyl (5*S*,6*S*,*E*)-5-((*tert*-butoxycarbonyl)amino)-6-phenyl-6-(((*S*)-3,3,3-trifluoro-2-methoxy-2-phenylpropanoyl)oxy)hex-2-enoate (S7). To a solution of aminoalcohol **49** (8.0 mg, 23.9 µmol, 98% ee) in dichloromethane (0.5 mL) were added DMAP (0.6 mg, 4.77 µmol), triethylamine (10.0 µL, 71.6 µmol) and (*R*)-Mosher's acid chloride (5.00 µL, 26.7 µmol) and the resulting mixture was stirred at room temperature overnight. Dichloromethane (2 mL) and sat. aq. NH_4Cl solution (2 mL) were added and the resulting mixture was vigorously stirred for 5 min. The organic layer was separated and the aqueous layer was extracted with dichloromethane (2 x 5 mL). The combined organic phases were dried over $MgSO_4$, filtered and the solvent was removed under reduced pressure. The residue was purified by flash chromatography (SiO_2, pentane/ethyl acetate, 7:1 → 4:1) to give the title compound as a colorless oil (7.5 mg, 57%). $[\alpha]_D^{20}$ = + 7.7 (c = 0.70, $CHCl_3$); ^1H NMR (600 MHz, $CDCl_3$): δ = 7.41 – 7.33 (m, 5H), 7.33 – 7.29 (m, 3H), 7.21 – 7.18 (m, 2H), 6.83 (dt, *J* = 15.6, 7.7 Hz, 1H), 5.92 (d, *J* = 6.6 Hz, 1H), 5.79 (d, *J* = 15.5 Hz, 1H), 4.53 (d, *J* = 9.4 Hz, 1H), 4.18 (br, 1H), 3.72 (s, 3H), 3.50 (s, 3H), 2.39 – 2.31 (m, 1H), 2.20 – 2.12 (m, 1H), 1.38 (s, 9H); ^{13}C NMR (151 MHz, $CDCl_3$): δ = 166.5, 165.6, 155.1, 143.6, 135.7, 131.8, 129.8, 129.1, 128.8 (2C), 128.6 (2C), 127.6 (2C), 127.3 (2C), 124.4, 123.4 (q, $^1J_{CF}$ = 288.7 Hz), 84.9 (q, $^2J_{CF}$ = 27.9 Hz), 80.2, 78.6, 55.7, 53.4, 51.7, 34.4, 28.4 (3C); ^{19}F NMR (565 MHz, $CDCl_3$): δ = -71.5; IR (ATR): ṽ = 3363, 2979, 1749, 1707, 1518, 1249, 1162, 1016, 721, 699 cm^{-1}; HRMS (ESI$^+$): *m/z*: calcd. for $C_{28}H_{32}F_3NO_7Na$ $[M+Na]^+$: 574.20231, found: 574.20279.

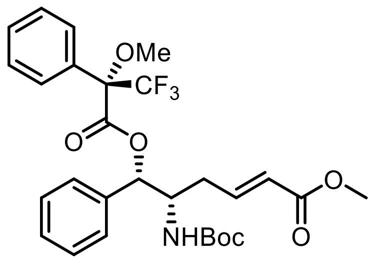

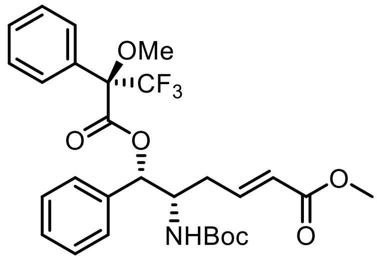

The diasteromeric Mosher ester, methyl (5*S*,6*S*,*E*)-5-((*tert*-butoxycarbonyl)amino)-6-phenyl-6-(((*R*)-3,3,3-trifluoro-2-methoxy-2-phenylpropanoyl)oxy)hex-2-enoate (**S8**), was prepared analogously using (*S*)-Mosher's acid chloride as a colorless oil (10.4 mg, 61%). $[\alpha]_D^{20}$ = + 32.2 (c = 0.90, $CHCl_3$); ^1H NMR (600 MHz, $CDCl_3$): δ = 7.44 – 7.38 (m, 3H), 7.37 – 7.33 (m, 5H), 7.31 – 7.27 (m, 2H), 6.81 (dt, *J* = 15.1, 7.3 Hz, 1H), 6.02 (d, *J* = 6.0 Hz, 1H), 5.76 (dt, *J* = 15.6, 1.5 Hz, 1H), 4.47 (d, *J* = 9.4 Hz, 1H), 4.12 (br, 1H), 3.71 (s, 3H), 3.47 (s, 3H), 2.35 – 2.25 (m, 1H), 2.18 – 2.10 (m, 1H), 1.35 (s, 9H); ^{13}C NMR (151 MHz, $CDCl_3$): δ = 166.5, 165.8, 155.0, 143.7, 135.8, 132.0, 129.9, 129.2, 128.9 (2C), 128.6 (2C), 127.6 (2C), 127.4 (2C), 124.2, 123.6 (q, $^1J_{CF}$ = 288.9 Hz), 84.7 (q, $^2J_{CF}$ = 27.8 Hz), 80.1, 78.2, 55.6, 53.6, 51.7, 34.4, 28.3 (3C); ^{19}F NMR (565 MHz, $CDCl_3$): δ = -71.1; IR (ATR): ṽ = 3363, 2953, 1749, 1715, 1499, 1250, 1167, 1015, 730, 699 cm^{-1}; HRMS (ESI$^+$): *m/z*: calcd. for $C_{28}H_{32}F_3NO_7Na$ $[M+Na]^+$: 574.20231, found: 574.20255.

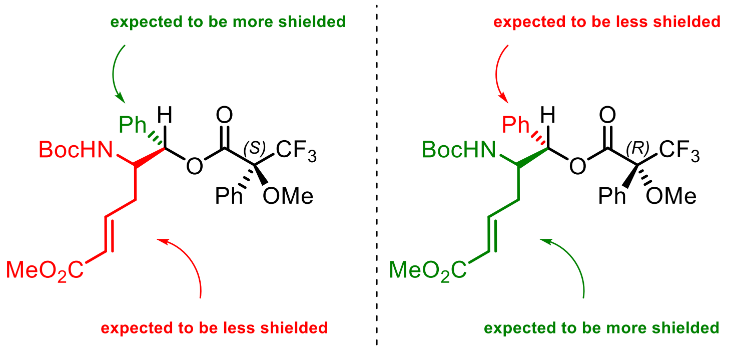

negative $\Delta\delta_{SR}$ expected for resonances of the phenyl group

positive $\Delta\delta_{SR}$ expected for resonances of the chain bearing the amino group and acrylate handle

Observed $\Delta\delta_{SR}$ values (Hz):

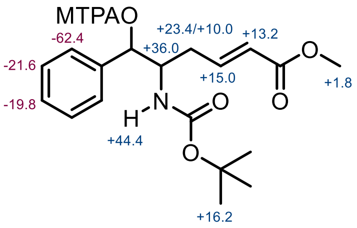

Stereocenter bearing the alcohol group assigned as (*S*). Due to the *syn*-configuration, the absolute configuration on the stereocenter bearing the amino group could be determined as (*S*) as well.

Scheme S4. Mosher ester analysis of compound **49**.[11]

Downstream Functionalization

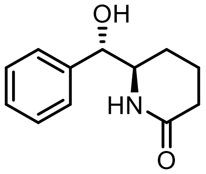

(*R*)-6-((*S*)-Hydroxy(phenyl)methyl)piperidin-2-one (46). A Schlenk flask under argon was charged with the recrystallized aminoalcohol derivative **15a** (67.5 mg, 0.20 mmol, 99% ee), Pd/C (15 mg, 14.1 µmol) and ethyl acetate (2 mL). The mixture was purged with H_2 from a balloon for 1 min and was then stirred under H_2-atmosphere for 30 min at room temperature. The mixture was filtered through a plug of Celite and the filtrate was evaporated under reduced pressure. The hydrogenated product was obtained as an off-white solid (quant.) and was directly used in the next step without further purification.

HCl in dioxane (4 M, 1 mL) was added to a solution of the crude product in 1,4-dioxane (2 mL). The mixture was stirred at 50 °C for 16 h. The solvent was removed under reduced pressure and the hydrochloride salt was obtained as a colorless solid (quant.), which was directly used in the next step without further purification.

The hydrochloride salt was dissolved in ethanol (3 mL). NEt_3 (112 µL, 0.81 mmol) was added and the resulting mixture was stirred at 65 °C for 17 h. The solvent was removed under reduced pressure and the residue was purified by flash chromatography (SiO_2, ethyl acetate/methanol, 100:6) to give the title compound as a colorless solid (36.7 mg, 89% yield, >99% ee). mp = 111-113 °C; $[\alpha]_D^{20}$ (>99% ee) = +65.8 (c = 0.50, $CHCl_3$); ^1H NMR (400 MHz, CD_2Cl_2): δ = 7.40 – 7.30 (m, 5H), 5.98 (s, 1H), 4.59 (d, *J* = 5.5 Hz, 1H), 3.65 – 3.60 (m, 1H), 3.15 (s, 1H), 2.28 – 2.21 (m, 1H), 2.14 – 2.05 (m, 1H), 1.86 – 1.76 (m, 2H), 1.66 – 1.55 (m, 1H), 1.51 – 1.41 (m, 1H); ^{13}C NMR (101 MHz, CD_2Cl_2): δ = 173.0, 140.6, 128.9 (2C), 128.6, 127.3 (2C), 76.4, 58.6, 31.9, 24.1, 19.8; IR (ATR): ṽ = 3392, 1645, 1451, 1410, 1329, 1305, 1192, 1065, 704 cm^{-1}; HRMS (ESI^+): *m/z*: calcd. for $C_{12}H_{15}NO_2Na$ $[M+Na]^+$: 228.09950, found: 228.09927.

The ee was determined by HPLC analysis: Chiralpak 150 mm IC-3, 3 µm, 4.6 mm ∅, *n*-heptane/*iso*-propanol = 60:40, v = 1.0 mL/min, λ = 210 nm, t(minor) = 9.72 min, t(major) = 12.67 min (racemate: left, enantioenriched sample: right).

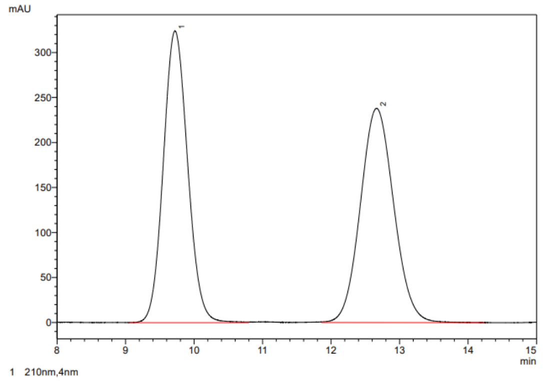

PDA Ch1 210nm

Peak #	Ret. Time	Area %	Name
1	9,72	49,80	1. Enantiomer
2	12,67	50,20	2. Enantiomer
Total		100,00	

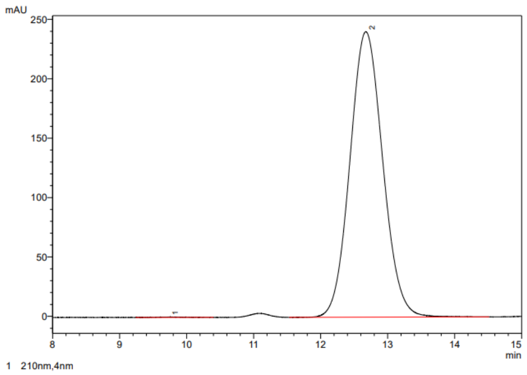

PDA Ch1 210nm

Peak #	Ret. Time	Area %	Name
1	9,74	0,13	1. Enantiomer
2	12,67	99,87	2. Enantiomer 99.7 % ee
Total		100,00	

(*S*)-Phenyl((*R*)-piperidin-2-yl)methanol (47). A flame dried pressure flask was charged with a solution of lithium aluminium hydride (1.0 M in THF, 0.90 mL, 0.90 mmol). A solution of lactam **46** (57.7 mg, 0.28 mmol, >99% ee) in THF (2 mL) was added dropwise at 0°C. After complete addition, the mixture was stirred at 65 °C (bath temperature) for 90 min. The mixture was cooled to 0 °C and the reaction carefully quenched by dropwise addition of sat. aq. Rochelle salt solution (3 mL). The mixture was diluted with dichloromethane (5 mL) and was vigorously stirred for 30 min. The organic phase was separated and the aqueous layer extracted with dichloromethane (4 x 10 mL). The combined organic phases were washed with brine (10 mL), dried over $MgSO_4$, filtered, and the solvent was removed under reduced pressure to give the title compound as an off-white solid that needed no further purification (53.4 mg, quant., >99% ee). mp = 124-126 °C; $[\alpha]_D^{20}$ (>99% ee) = +42.8 (c = 0.50, $CHCl_3$); ^{1}H NMR (400 MHz, CD_2Cl_2): δ = 7.36 – 7.23 (m, 5H), 4.69 (d, *J* = 4.4 Hz, 1H), 3.21 (br, 2H), 3.11 – 3.06 (m, 1H), 2.82 – 2.77 (m, 1H), 2.64 (td, *J* = 12.0, 2.9 Hz, 1H), 1.79 – 1.69 (m, 1H), 1.57 – 1.53 (m, 1H), 1.45 – 1.17 (m, 4H); ^{13}C NMR (101 MHz, CD_2Cl_2): δ = 141.9, 128.5 (2C), 127.6, 126.8 (2C), 76.1, 62.5, 47.0, 26.2, 25.9, 24.5; IR (ATR): ṽ = 3287, 2926, 1448, 1432, 1262, 1108, 997, 899, 817, 701 cm^{-1}; HRMS (ESI$^+$): *m/z*: calcd. for $C_{12}H_{18}NO$ $[M+H]^+$: 192.13829, found: 192.13845.

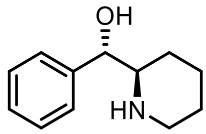

The ee was determined by HPLC analysis: Chiralpak 150 mm IG-3, 3 µm, 4.6 mm ∅, *n*-heptane + 0.05% DEA/*iso*-propanol = 95:5, v = 1.0 mL/min, λ = 210 nm, t(minor) = 7.62 min, t(major) = 8.46 min (racemate: left, enantioenriched sample: right).

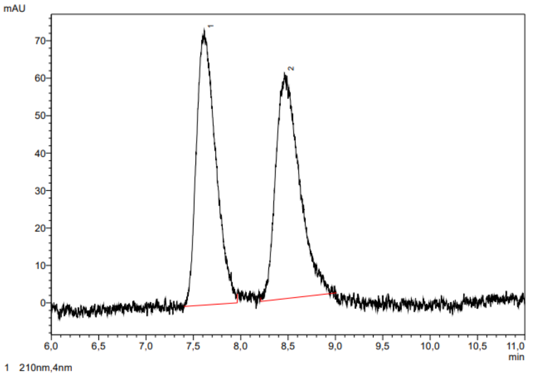

PDA Ch1 210nm

Peak #	Ret. Time	Area %	Name
1	7,62	49,71	1. Enantiomer
2	8,46	50,29	2. Enantiomer
Total		100,00	

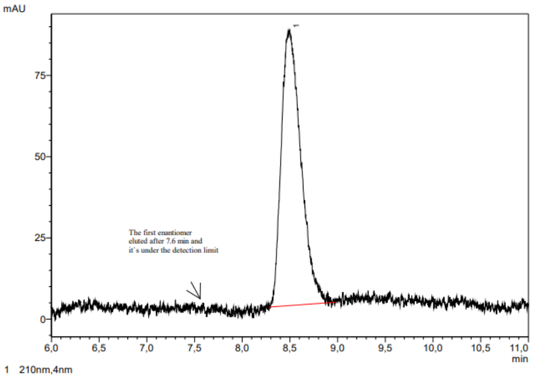

PDA Ch1 210nm

Peak #	Ret. Time	Area %	Name
1	8,50	100,00	2. Enantiomer
Total		100,00	

Methyl (*E*)-4-((4*R*,5*R*)-2-oxo-5-phenyloxazolidin-4-yl)but-2-enoate (48). A flame dried Schlenk flask

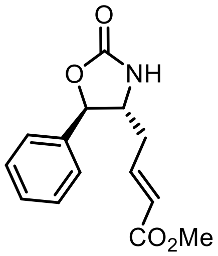

under argon was charged with the recrystallized aminoalcohol derivative **15a** (41.0 mg, 0.12 mmol, 99% ee) and diethyl ether (1.0 mL). The mixture was cooled to 0 °C before $SOCl_2$ (89.0 µL, 1.23 mmol) was added dropwise. The mixture was allowed to warm to room temperature and stirred for 2 h. The reaction was carefully quenched at 0 °C by dropwise addition of sat. aq. $NaHCO_3$ (2.0 mL). Ethyl acetate (2 mL) was added, the organic phase was separated and the aqueous phase was extracted with ethyl acetate (3 x 5 mL). The combined organic phases were washed with brine (5 mL), dried over $MgSO_4$, filtered and the solvent was removed under reduced pressure. The residue was purified by flash chromatography (SiO_2, pentane/ethyl acetate, 3:1 → 1:2) to give the title compound as a colorless solid (27.9 mg, 87% yield, >99% ee). mp = 129-131 °C; $[\alpha]_D^{20}$ (>99% ee) = +52.8 (c = 0.50, $CHCl_3$); ^1H NMR (400 MHz, $CDCl_3$): δ = 7.43 – 7.32 (m, 5H), 6.86 (ddd, *J* = 15.7, 7.8, 6.9 Hz, 1H), 6.00 (br, 1H), 5.99 (dt, *J* = 15.6, 1.4 Hz, 1H), 5.15 (d, *J* = 6.2 Hz, 1H), 3.87 (dddd, *J* = 7.2, 6.2, 5.2, 1.1 Hz, 1H), 3.74 (s, 3H), 2.69 – 2.53 (m, 2H); ^{13}C NMR (101 MHz, $CDCl_3$): δ = 166.2, 158.6, 141.9, 137.7, 129.4, 129.2 (2C), 126.0 (2C), 125.5, 82.8, 59.5, 51.9, 37.5; IR (ATR): ṽ =3305, 2923, 1752, 1720, 1436, 1278, 1224, 1167, 1010, 699 cm^{-1}; HRMS (ESI$^+$): *m/z*: calcd. for $C_{14}H_{15}NO_4Na$ $[M+Na]^+$: 284.08933, found: 284.08920.

The ee was determined by HPLC analysis: Chiralpak 150 mm IG-3, 3 µm, 4.6 mm ∅, CH_3CN/water = 50:50, v = 1.0 mL/min, λ = 220 nm, t(major) = 4.89 min, t(minor) = 5.63 min (racemate: left, enantioenriched sample: right).

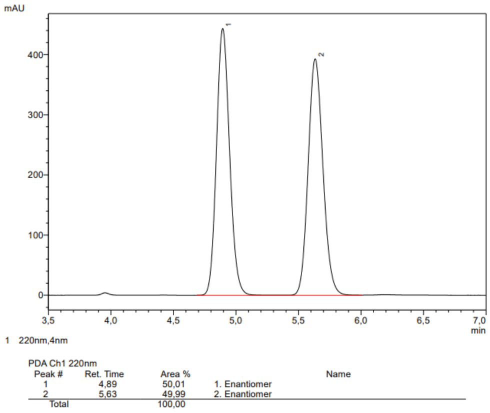

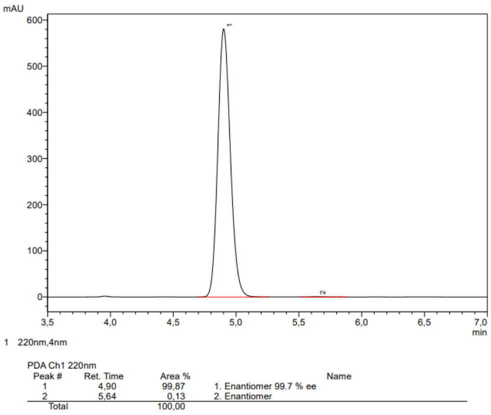

Note: Single crystals suitable for X-ray diffraction analysis were obtained by recrystallization from cyclohexane/ethyl acetate, which were used to confirm the cyclization proceeds with inversion of the alcohol center in **15a** to give the *syn*-configured product **48**, see Figure S5

Methyl 2-((2*S*,4*R*,5*S*)-4-((*tert*-butoxycarbonyl)amino)-5-phenyltetrahydrofuran-2-yl)acetate and Diastereomer (49).

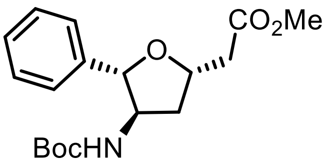

(major isomer)

DBU (2.5 µL, 16.7 µmol, 0.22 eq.) was added to a solution of the recrystallized aminoalcohol **15a** (25.5mg, 76.0 µmol, 99% ee) in THF (0.75 mL) and the resulting mixture was stirred at room temperature for 16 h. The solvent was evaporated and the residue purified by flash chromatography (SiO_2, *iso*-hexane/ethyl acetate, 2:1) to give the title compound as a colorless solid (24.8 mg, 73.9 µmol, 97% yield, 2:1 dr). *Note: The compound shows partially broadened signals in NMR at room temperature and was therefore analyzed at 263K. The dr was determined as 2:1, with one rotamer present for each diastereomer. The ratio of rotamer pairs to each other was ~ 4:1.* mp = 100-102 °C; ^{1}H NMR (600 MHz, 263 K, $CDCl_3$): δ = 7.42 – 7.39 (m, 2H), 7.36 – 7.32 (m, 2H), 7.29 – 7.24 (m, 1H), 5.00 (d, *J* = 7.4 Hz, 1H), 4.83 (d, *J* = 3.7 Hz, 1H), 4.66 – 4.58 (m, 1H), 4.14 – 4.10 (m, 1H), 3.72 (3H), 2.84 (dd, *J* = 15.8, 7.4 Hz, 1H), 2.67 (dd, *J* = 15.8, 5.8 Hz, 1H), 2.03 (ddd, *J* = 13.1, 5.9, 3.0 Hz, 1H), 1.93 (ddd, *J* = 13.1, 9.5, 7.2 Hz, 1H), 1.43 (s, 9H); ^{13}C NMR (151 MHz, 263 K, $CDCl_3$): δ =171.6, 155.2, 140.5, 128.4 (2C), 127.7, 125.7 (2C), 86.6, 79.9, 75.0, 59.0, 52.2, 40.4, 37.3, 28.4 (3C); IR (ATR): ṽ =3354, 2977, 1737, 1708, 1521, 1366, 1249, 1168, 1062, 701 cm^{-1}; HRMS (ESI$^+$): *m/z*: calcd. for $C_{18}H_{26}NO_5$ $[M+H]^+$: 336.18055, found: 336.18032.

The diastereomers were assigned based on NOESY data:

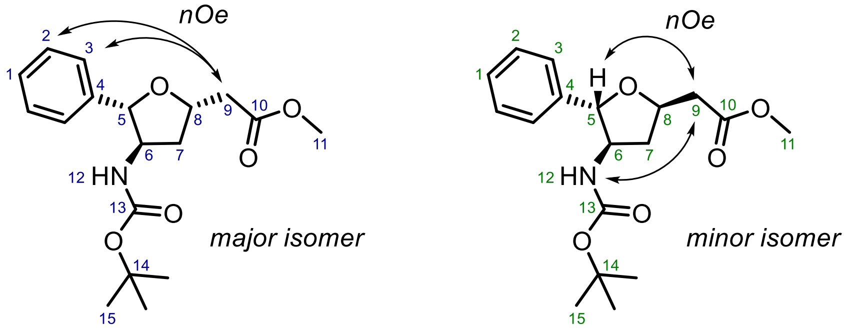

References

1. Bowen, J. I.; Wang, L.; Crump, M. P.; Willis, C. L., Ambruticins: tetrahydropyran ring formation and total synthesis. *Org. Biom. Chem.* **2021,** *19* (28), 6210-6215.
2. Guimond, N.; MacDonald, M. J.; Lemieux, V.; Beauchemin, A. M., Catalysis through Temporary Intramolecularity: Mechanistic Investigations on Aldehyde-Catalyzed Cope-type Hydroamination Lead to the Discovery of a More Efficient Tethering Catalyst. *J. Am. Chem. Soc.* **2012,** *134* (40), 16571-16577.
3. Ding, Z.; Osminski, W. E. G.; Ren, H.; Wulff, W. D., Scalable Syntheses of the Vaulted Biaryl Ligands VAPOL and VANOL via the Cycloaddition/Electrocyclization Cascade. *Prg. Process Res. Dev.* **2011,** *15* (5), 1089-1107.
4. Davies, T. Q.; Murphy, J. J.; Dousset, M.; Fürstner, A., Nickel-Catalyzed Enantioselective Synthesis of Pre-Differentiated Homoallylic syn- or anti-1,2-Diols from Aldehydes and Dienol Ethers. *J. Am. Chem. Soc.* **2021,** *143* (34), 13489-13494.
5. Boisvert, L.; Beaumier, F.; Spino, C., Evidence for a Concerted [4 + 1]- Cycloaddition between Electron-Rich Carbenes and Electron-Deficient Dienes. *Org. Lett.* **2007,** *9* (26), 5361-5363.
6. Pollack, S. R.; Dion, A., Metal-Free Stereoselective Synthesis of (*E*)- and (*Z*)-N-Monosubstituted β-Aminoacrylates via Condensation Reactions of Carbamates. *J. Org. Chem.* **2021,** *86* (17), 11748-11762.
7. Yin, X.; Zheng, L.; Mohammadlou, A.; Cagnon, B. R.; Wulff, W. D., Resolution of Vaulted Biaryl Ligands via Borate Esters of Quinine and Quinidine. *J. Org. Chem.* **2020,** *85* (16), 10432-10450.
8. Kimura, M.; Ezoe, A.; Mori, M.; Iwata, K.; Tamaru, Y., Regio- and Stereoselective Nickel-Catalyzed Homoallylation of Aldehydes with 1,3-Dienes. *J. Am. Chem. Soc.* **2006,** *128* (26), 8559-8568.
9. Guan, Y.; Ding, Z.; Wulff, W. D., Vaulted Biaryls in Catalysis: A Structure–Activity Relationship Guided Tour of the Immanent Domain of the VANOL Ligand. *Chem. Eur. J.* **2013,** *19* (46), 15565-15571.
10. Hashimoto, T.; Nakatsu, H.; Maruoka, K., Catalytic Asymmetric Diels–Alder Reaction of Quinone Imine Ketals: A Site-Divergent Approach. *Angew. Chem. Int. Ed.* **2015,** *54* (15), 4617-4621.
11. Hoye, T. R.; Jeffrey, C. S.; Shao, F., Mosher ester analysis for the determination of absolute configuration of stereogenic (chiral) carbinol carbons. *Nat. Protocols* **2007,** *2* (10), 2451-2458.

NMR Spectra of New Compounds

^{1}H NMR (400 MHz, $CDCl_3$; top) and ^{13}C NMR (101 MHz, $CDCl_3$; bottom) of compound **9**

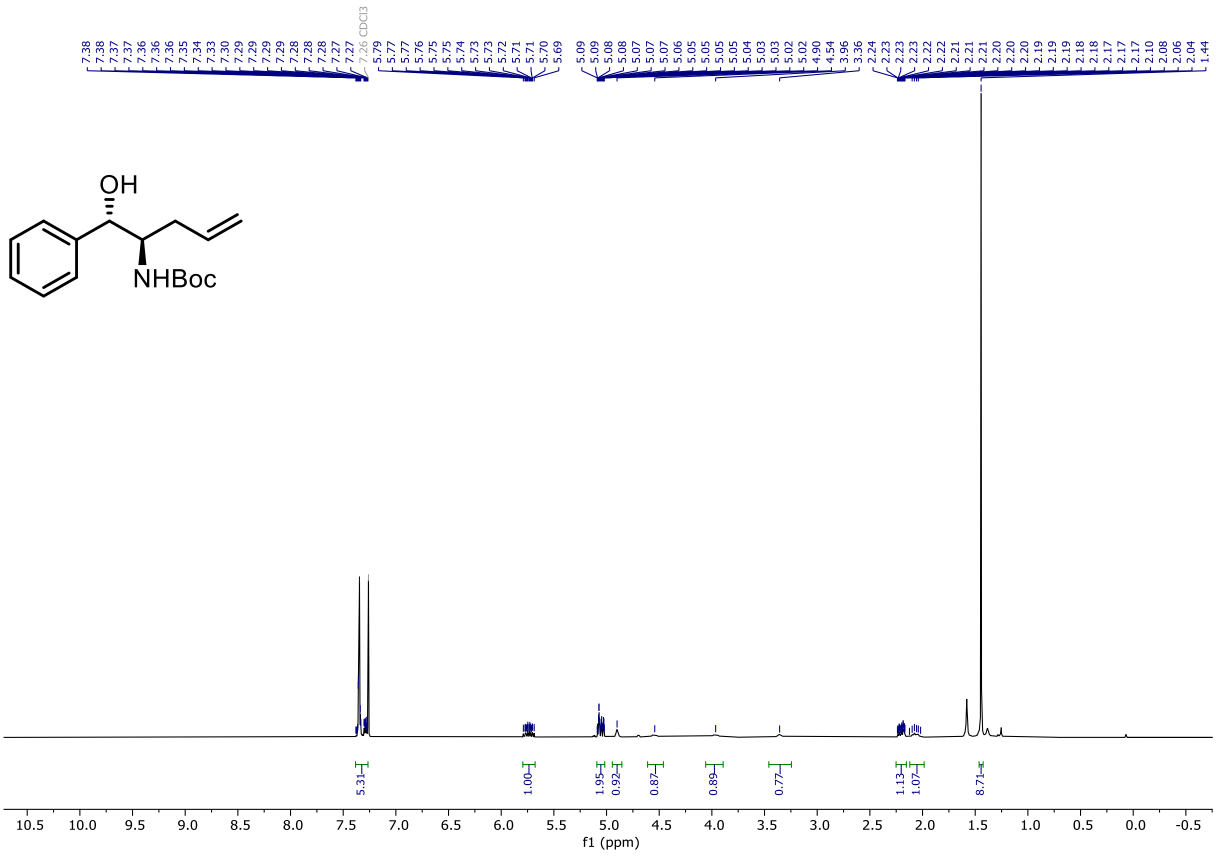

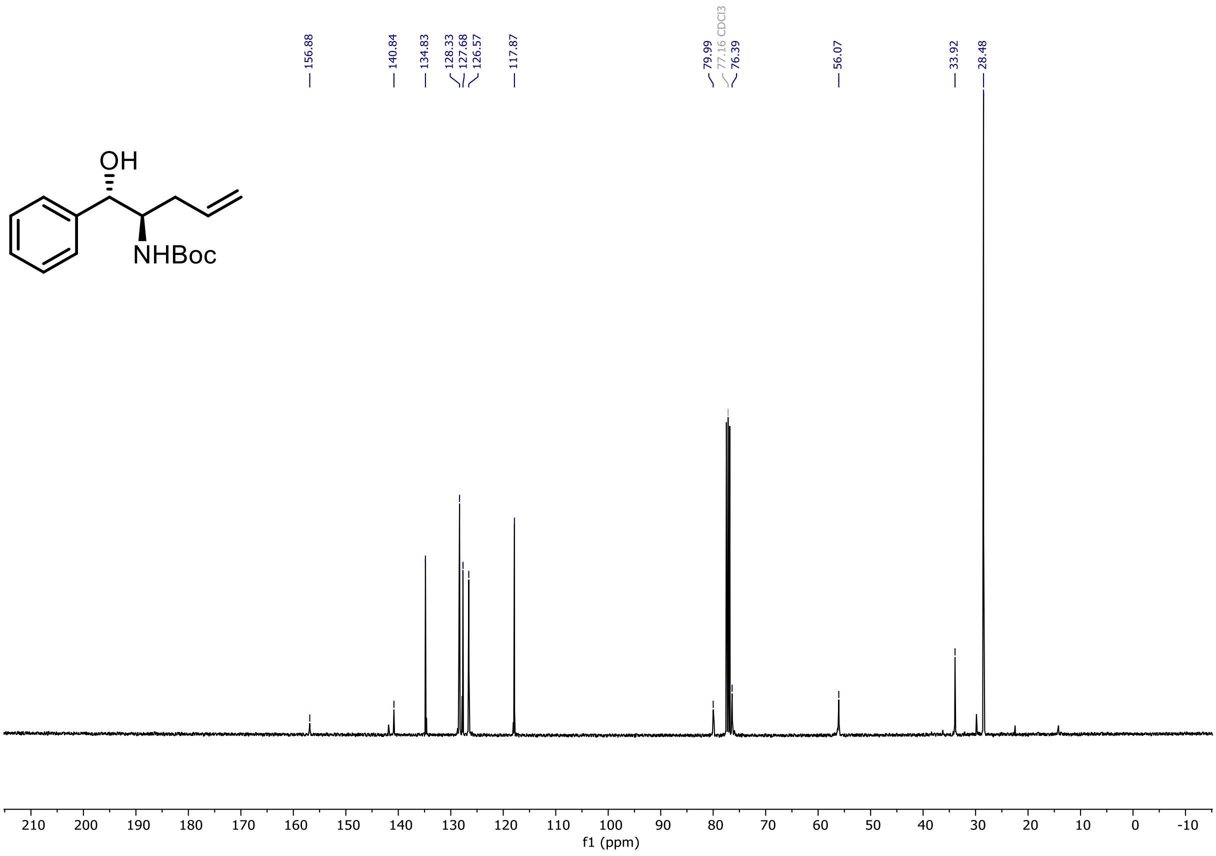

^{1}H NMR (400 MHz, CD_2Cl_2; top) and ^{13}C NMR (101 MHz, CD_2Cl_2; bottom) of compound **12**

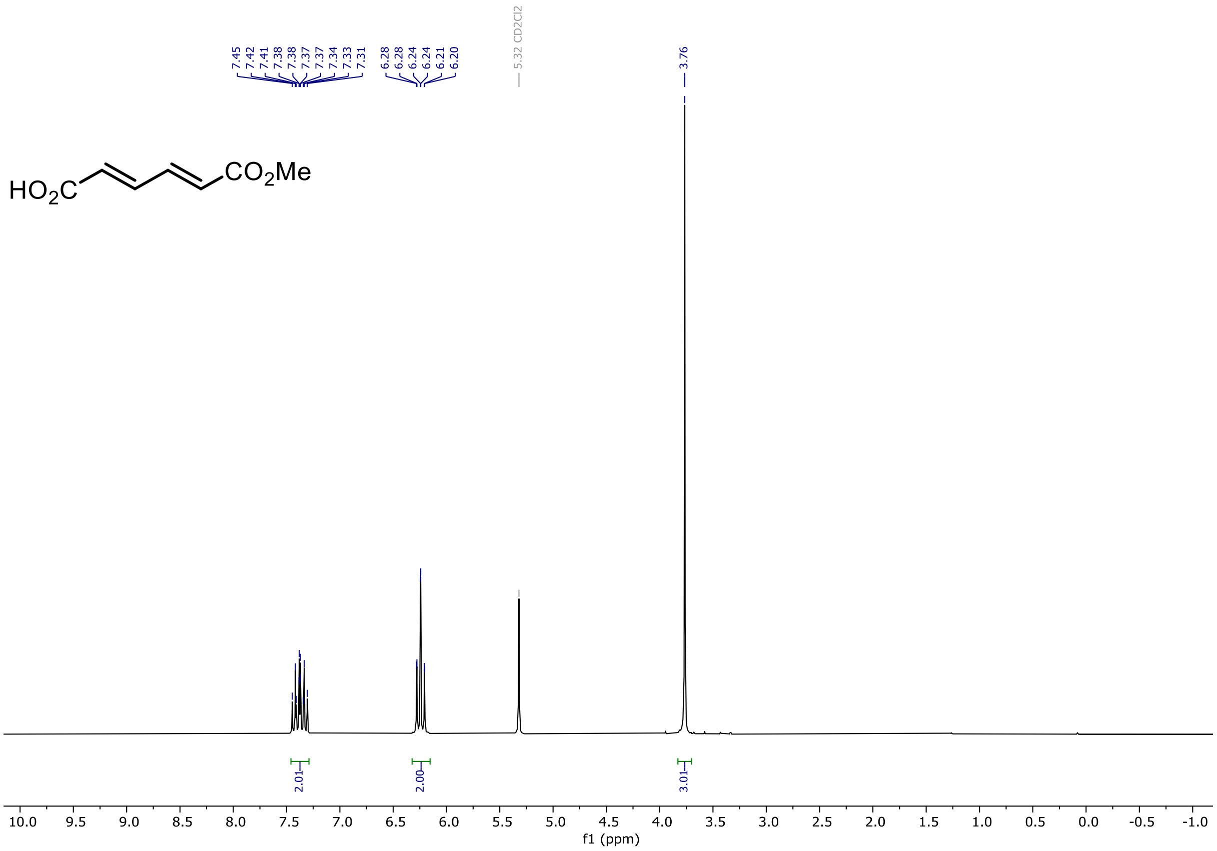

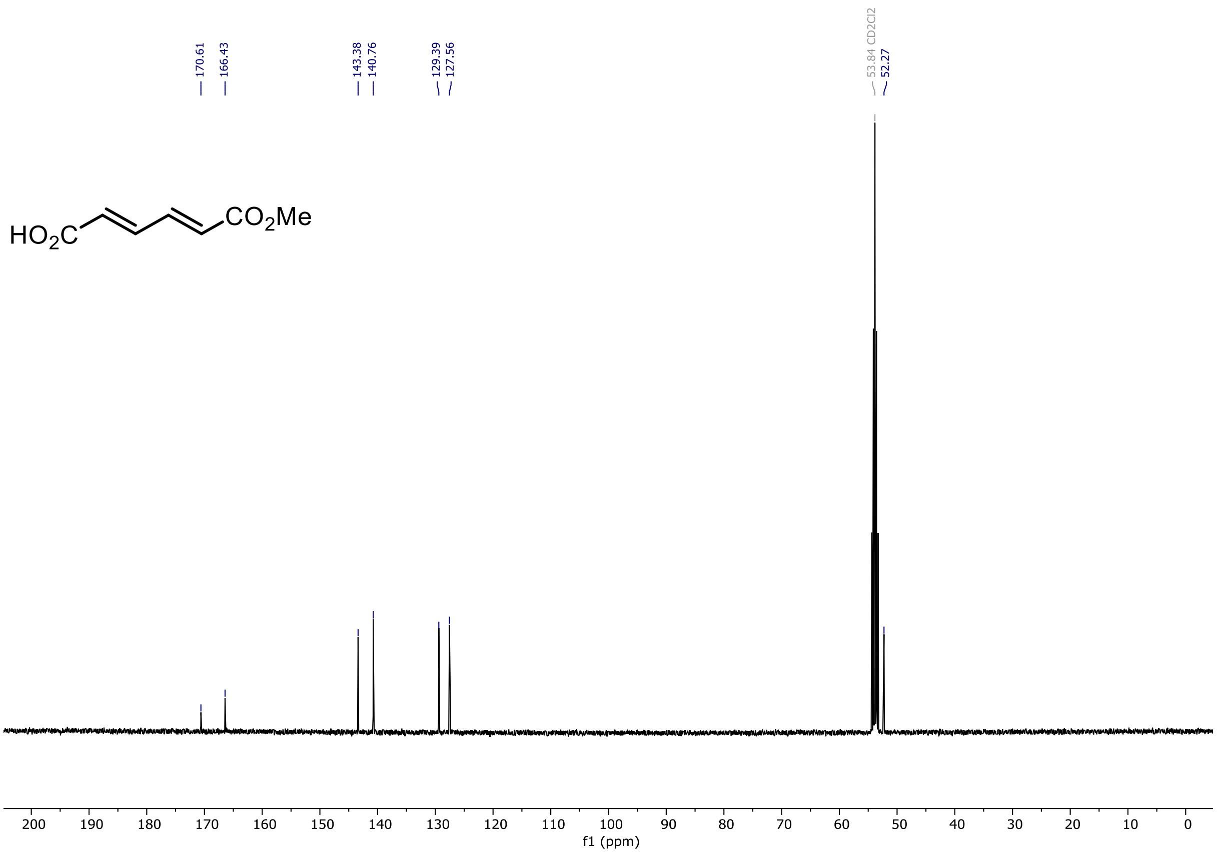

^{1}H NMR (400 MHz, CD_2Cl_2; top) and ^{13}C NMR (101 MHz, CD_2Cl_2; bottom) of compound **13a**

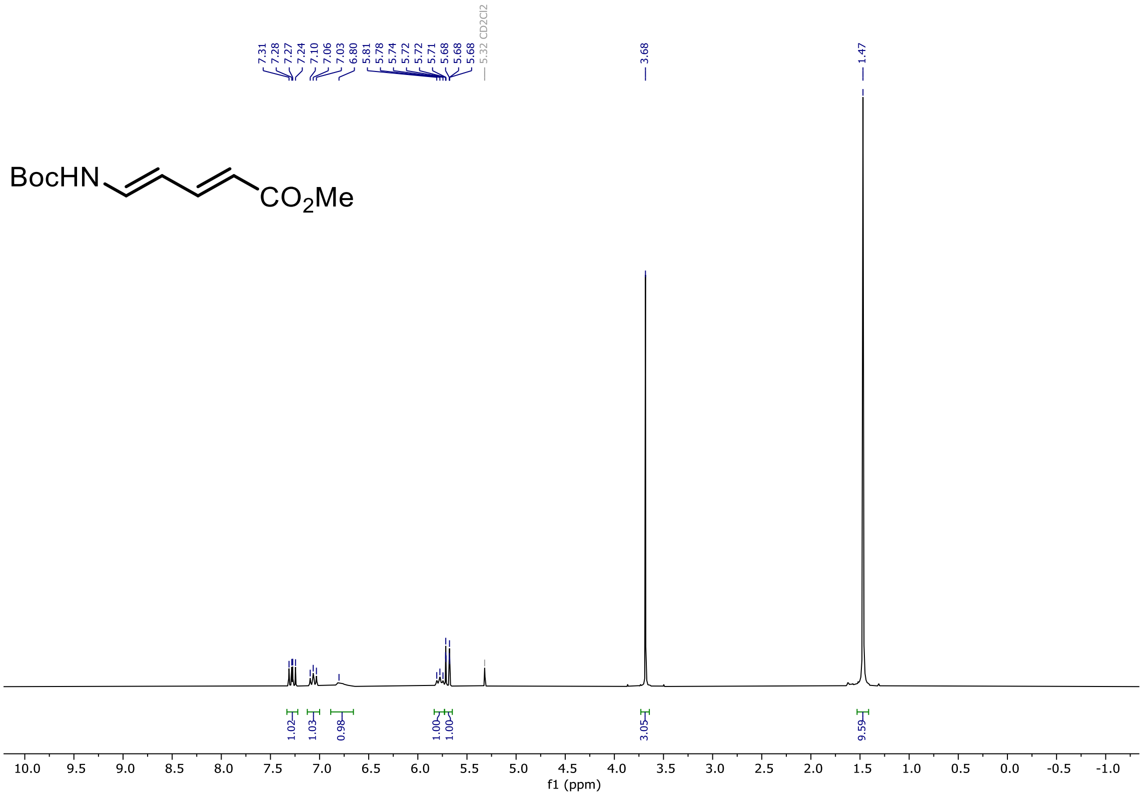
BocHN
CO2Me
7.31
7.28
7.27
7.24
7.10
7.06
7.03
6.80
5.81
5.78
5.74
5.72
5.72
5.71
5.68
5.68
5.68
5.32 CD2Cl2
3.68
1.47
1.02
1.03
0.98
1.00
1.00
3.05
9.59
10.0 9.5 9.0 8.5 8.0 7.5 7.0 6.5 6.0 5.5 5.0 4.5 4.0 3.5 3.0 2.5 2.0 1.5 1.0 0.5 0.0 -0.5 -1.0
f1 (ppm)

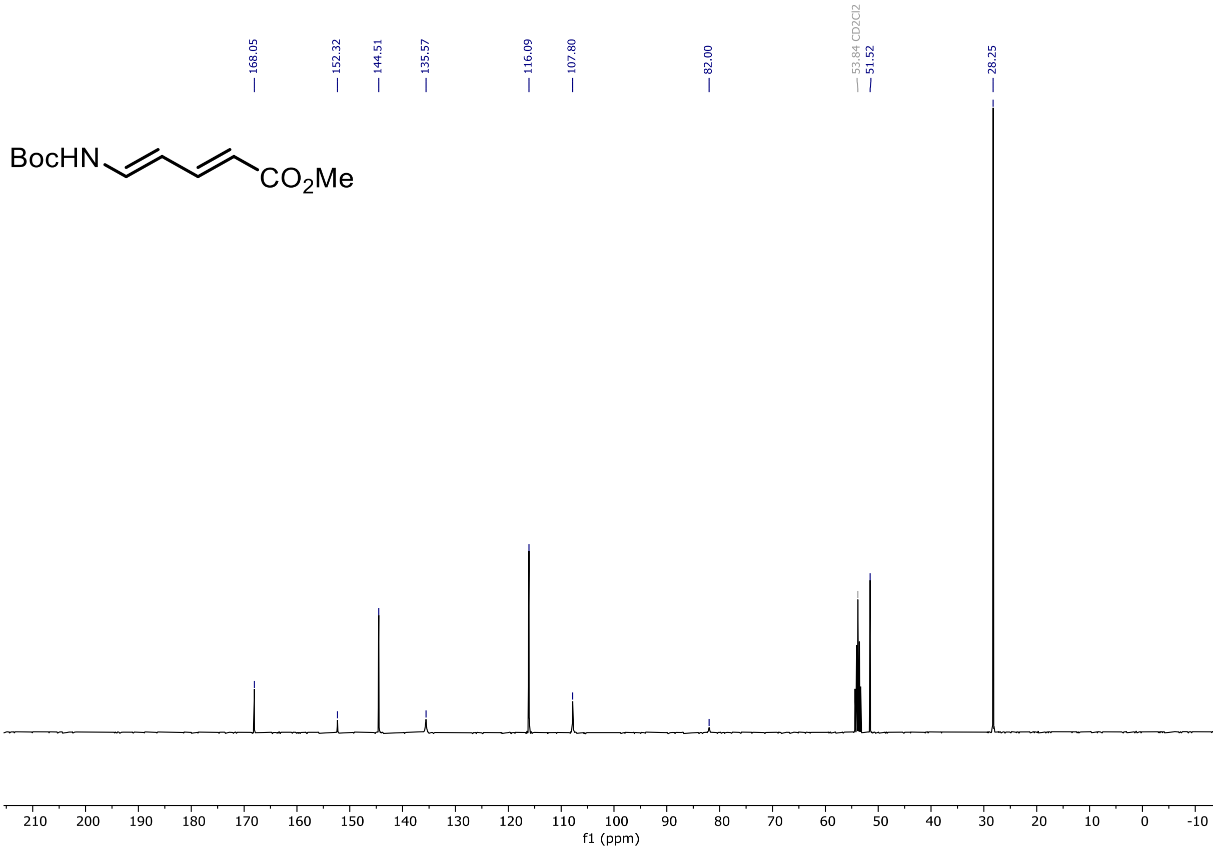
BocHN
CO2Me
168.05
152.32
144.51
135.57
116.09
107.80
82.00
53.84 CD2Cl2
51.52
28.25
210 200 190 180 170 160 150 140 130 120 110 100 90 80 70 60 50 40 30 20 10 0 -10
f1 (ppm)

^{1}H NMR (400 MHz, [D_6]-DMSO; top) and ^{13}C NMR (101 MHz, [D_6]-DMSO; bottom) of compound **13b**

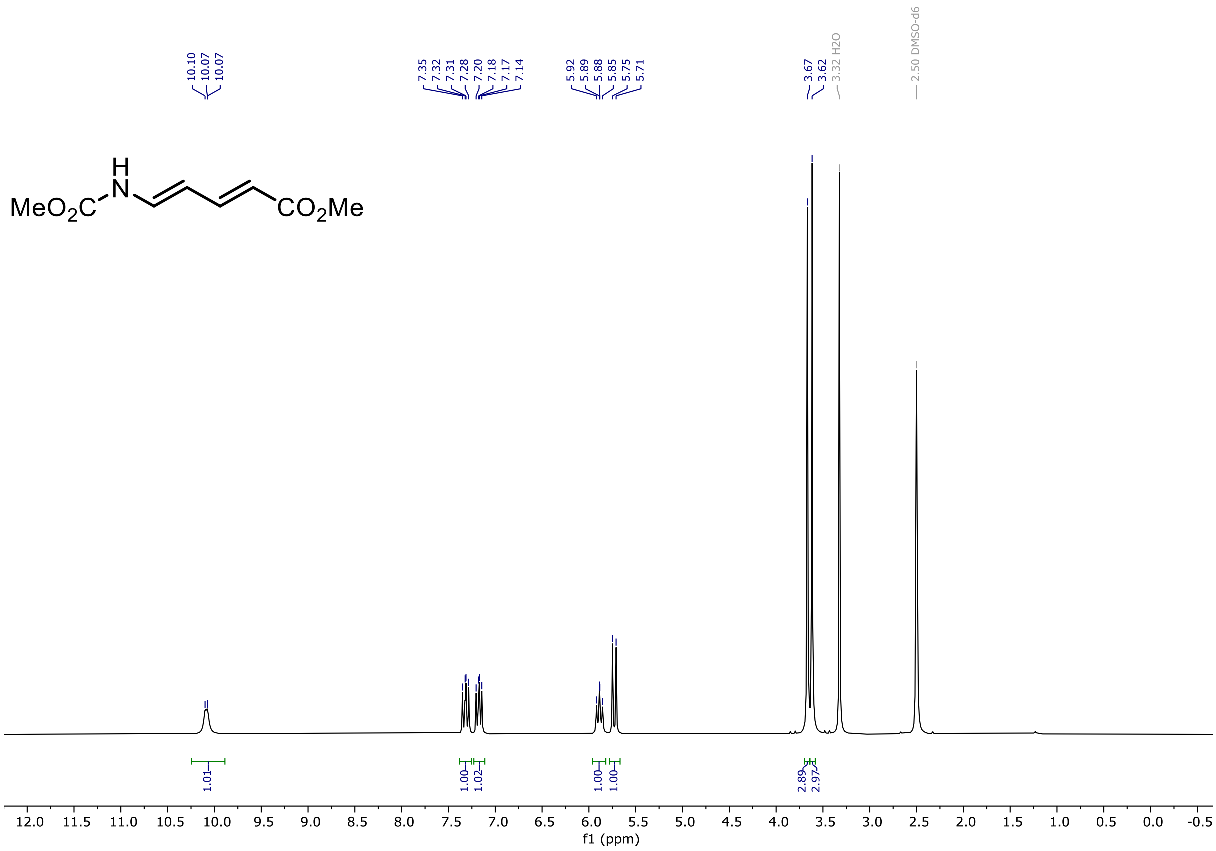

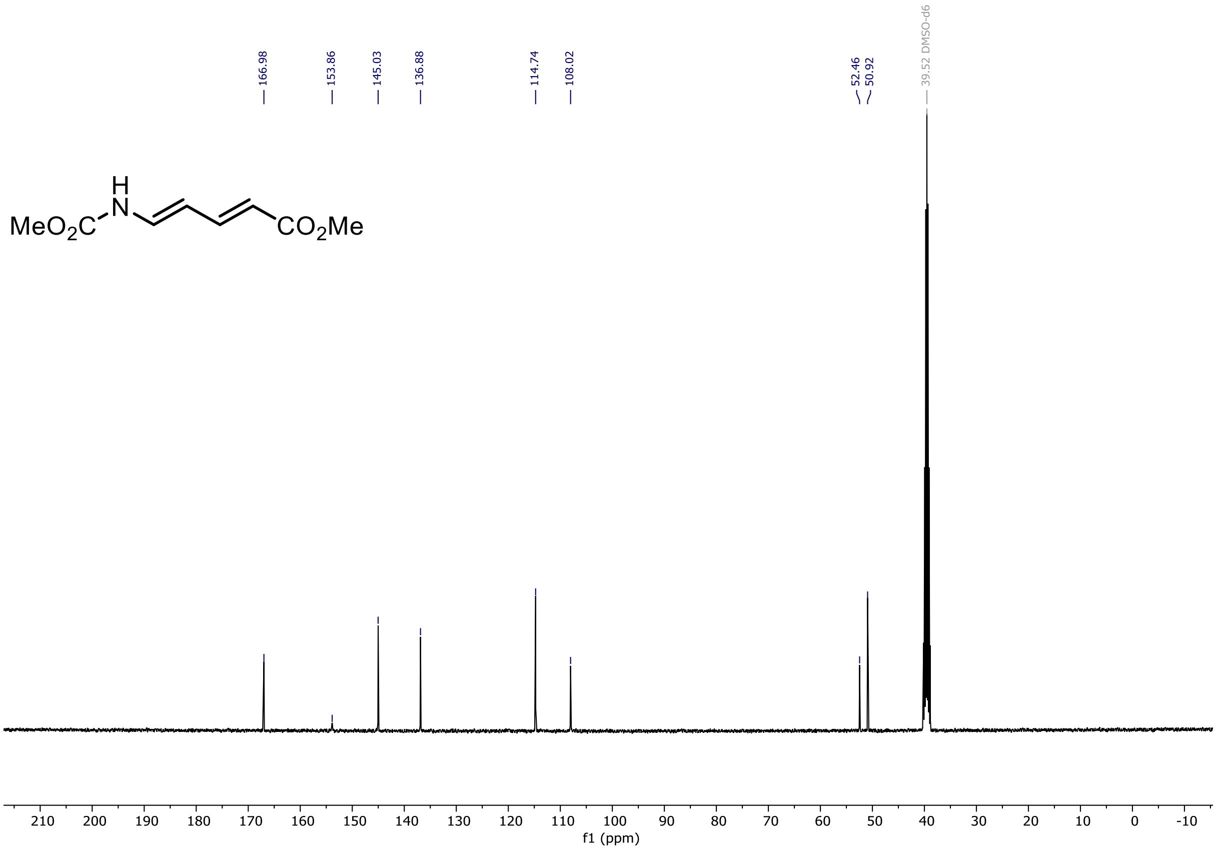

^1H NMR (400 MHz, CD_2Cl_2; top) and ^{13}C NMR (101 MHz, CD_2Cl_2; bottom) of compound **13c**

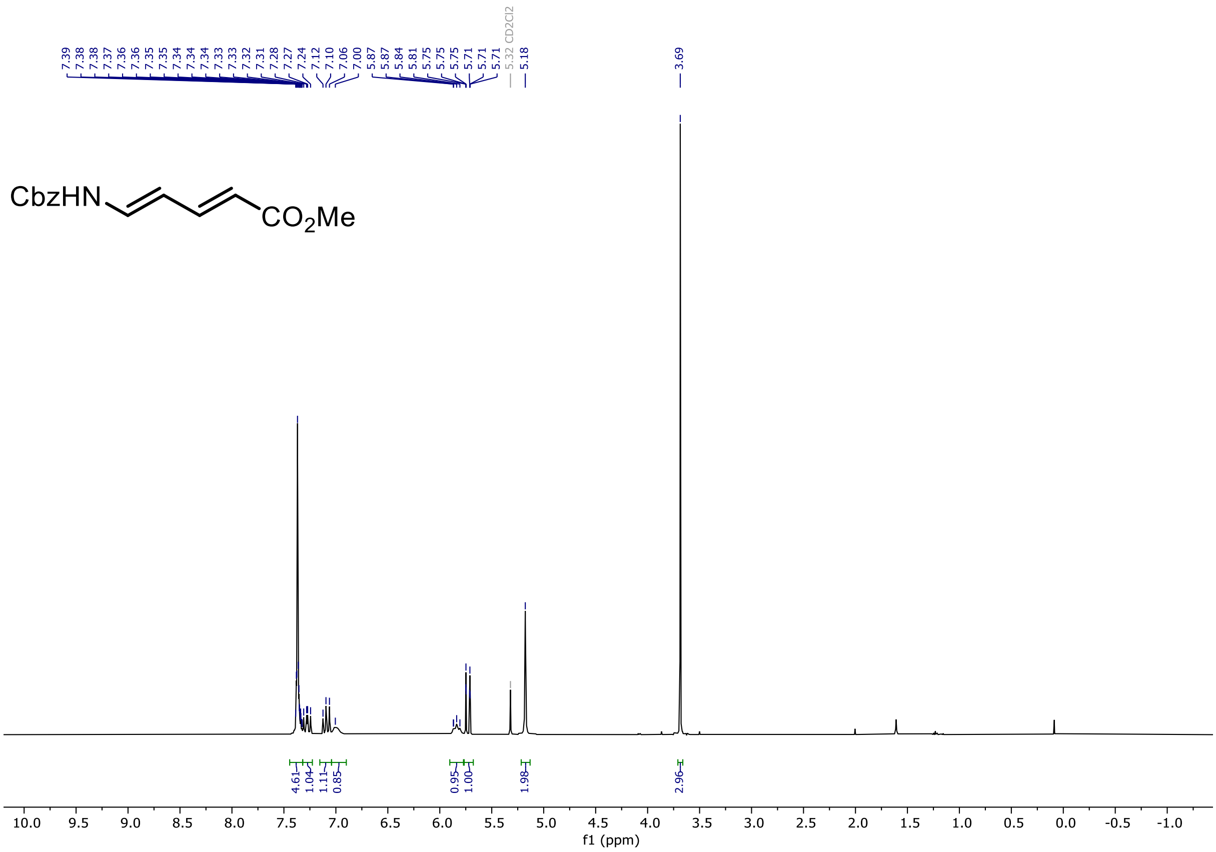

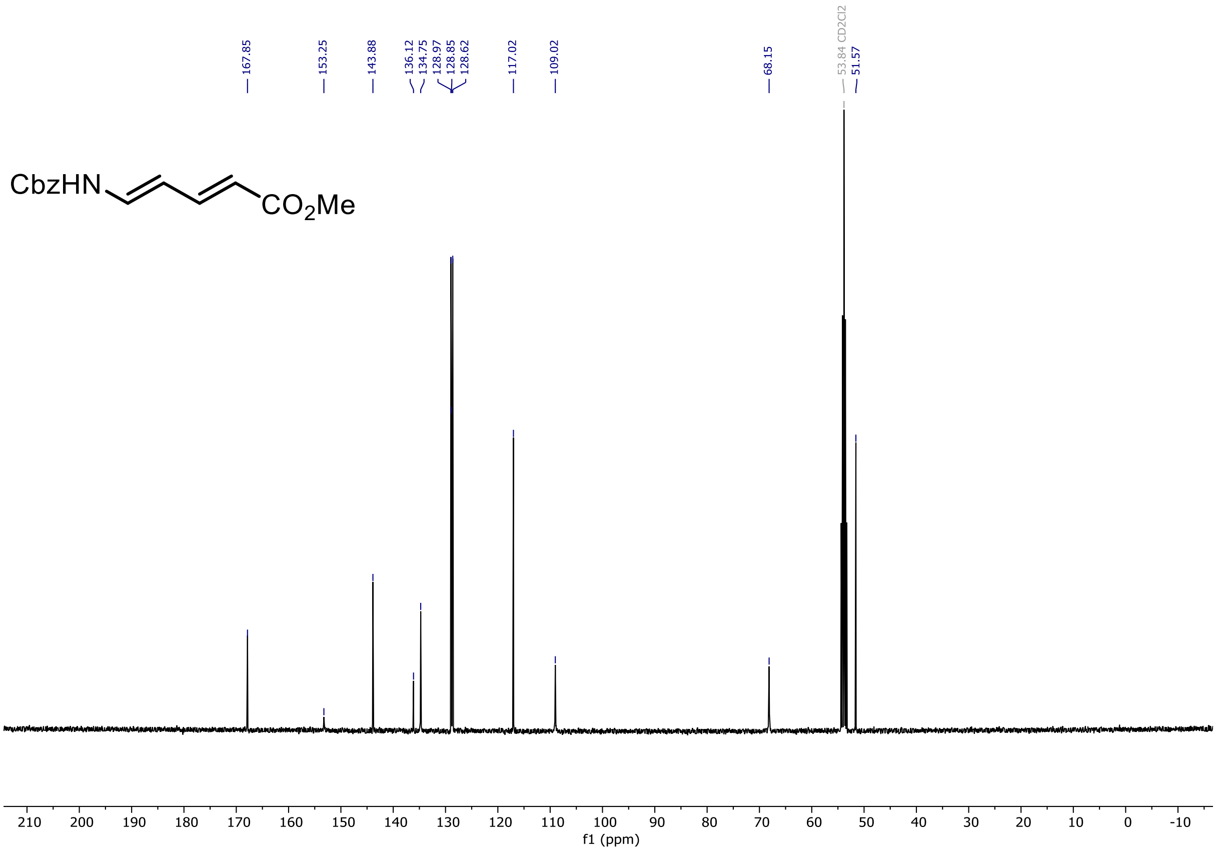

^{1}H NMR (400 MHz, CD_2Cl_2; top) and ^{13}C NMR (101 MHz, CD_2Cl_2; bottom) of compound **13d**

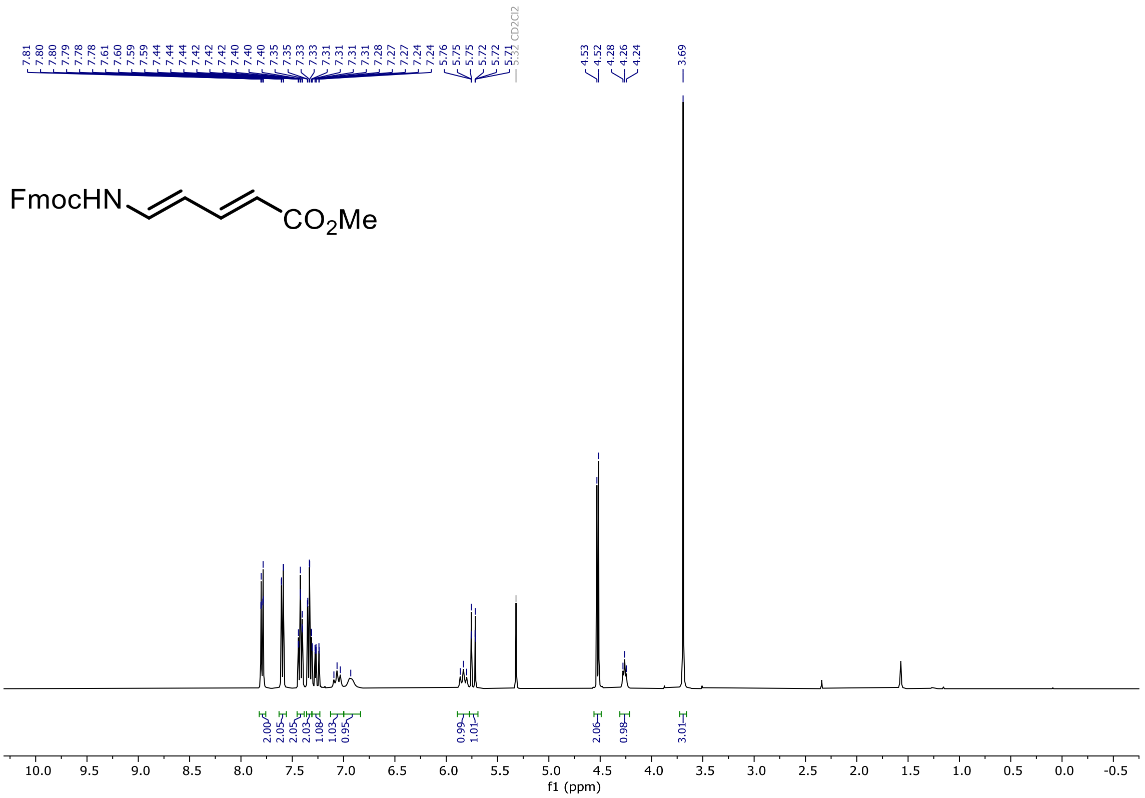

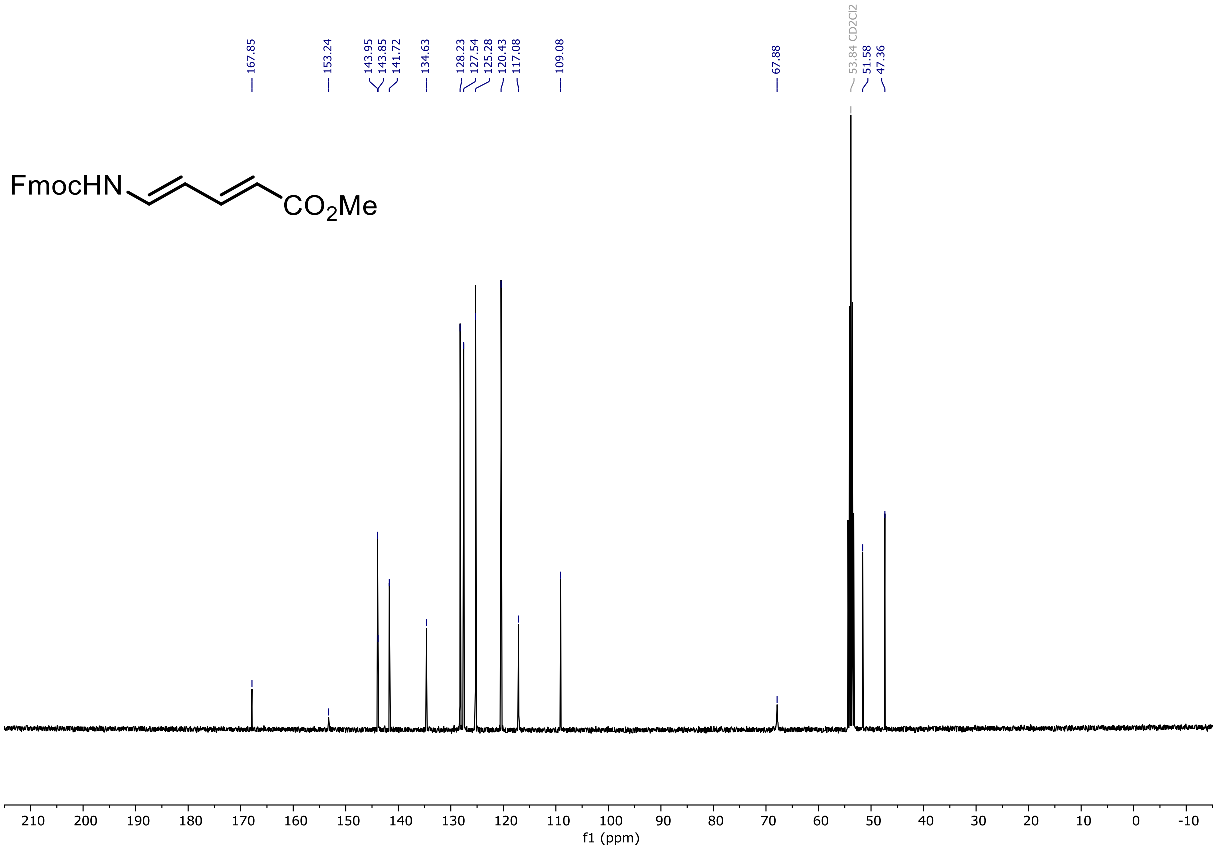

^{1}H NMR (400 MHz, $CDCl_3$; top) and ^{13}C NMR (101 MHz, $CDCl_3$; bottom) of compound **S1**

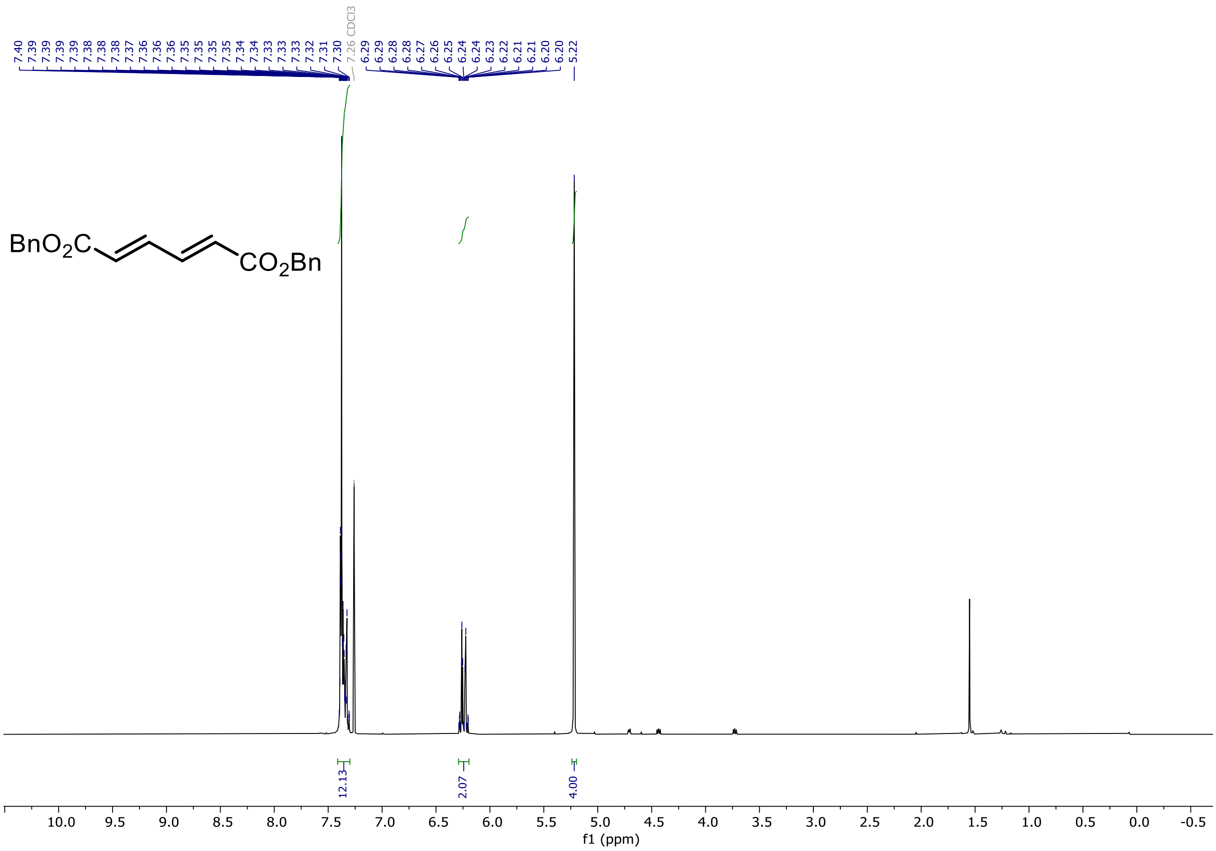

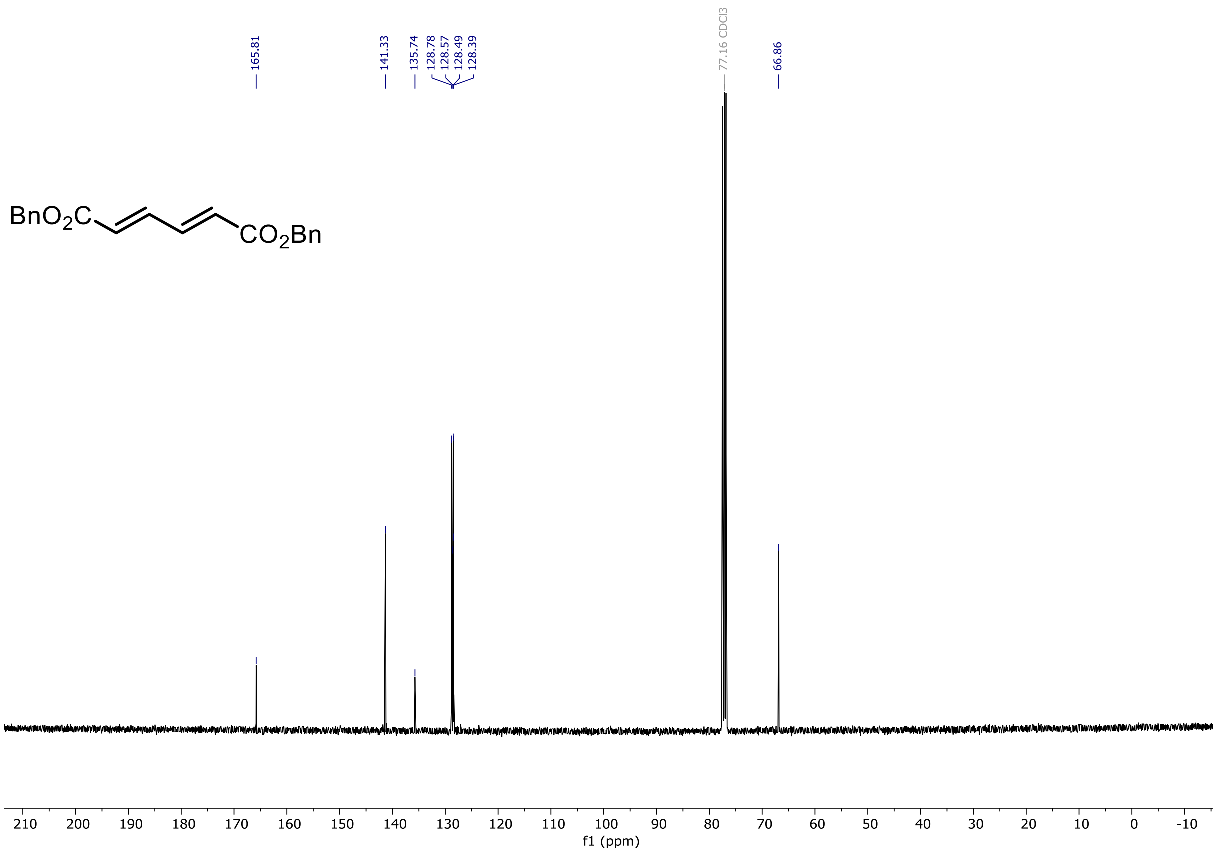

^{1}H NMR (400 MHz, $CDCl_3$; top) and ^{13}C NMR (101 MHz, $CDCl_3$; bottom) of compound **S2**

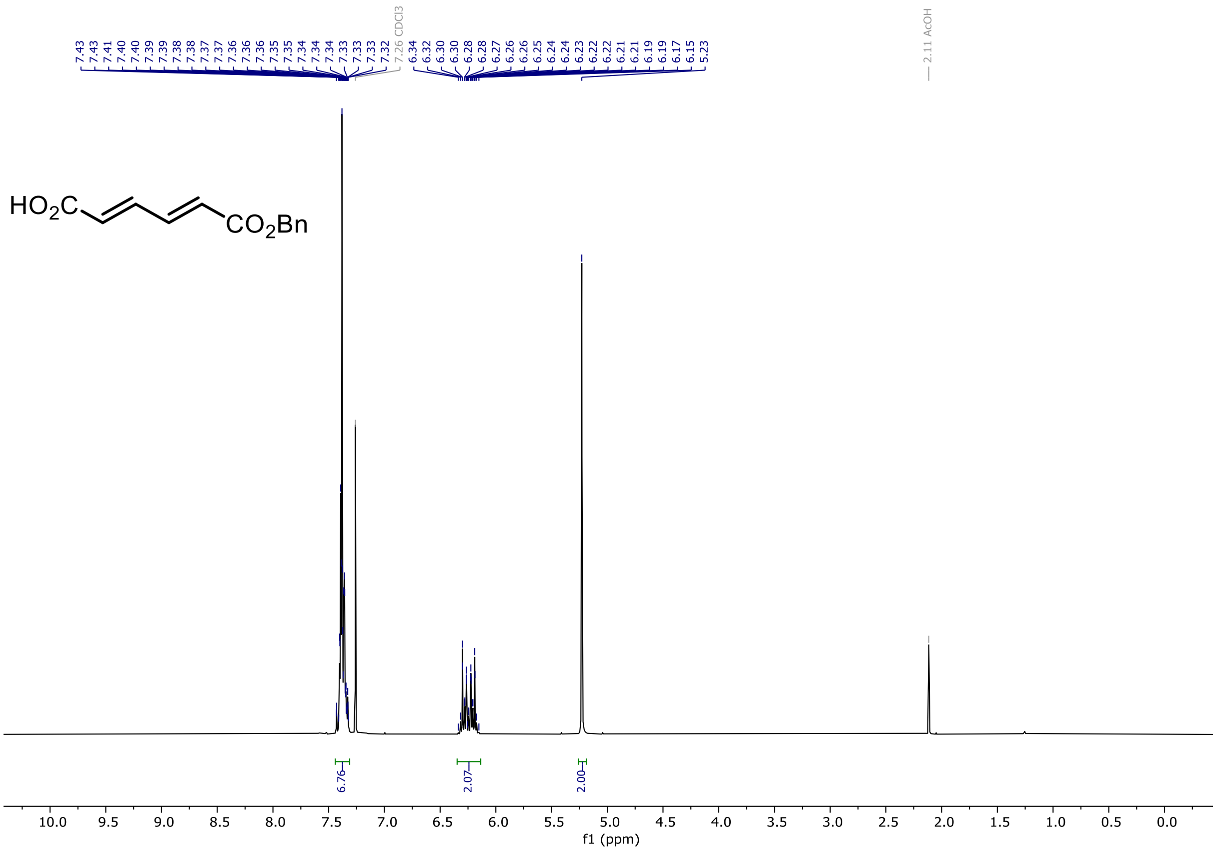

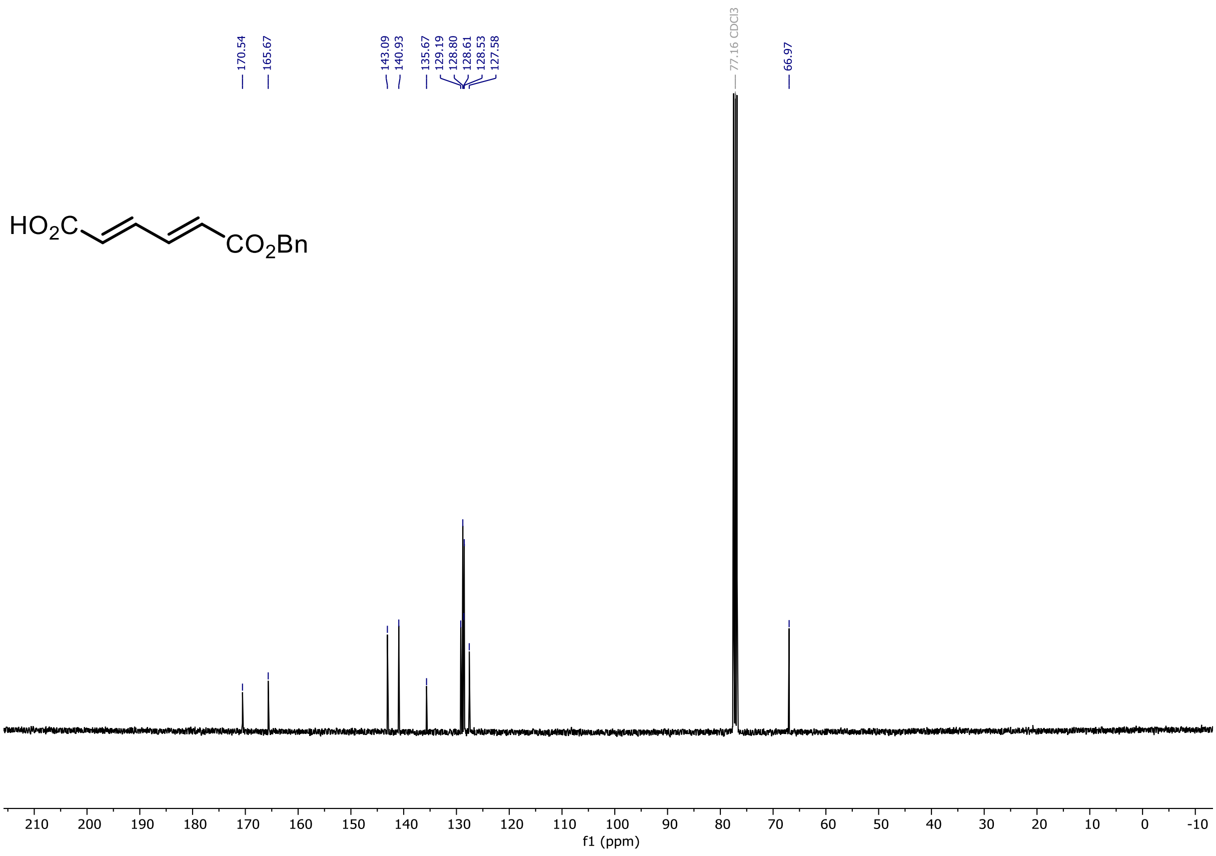

^{1}H NMR (400 MHz, CD_2Cl_2; top) and ^{13}C NMR (101 MHz, CD_2Cl_2; bottom) of compound **S3**

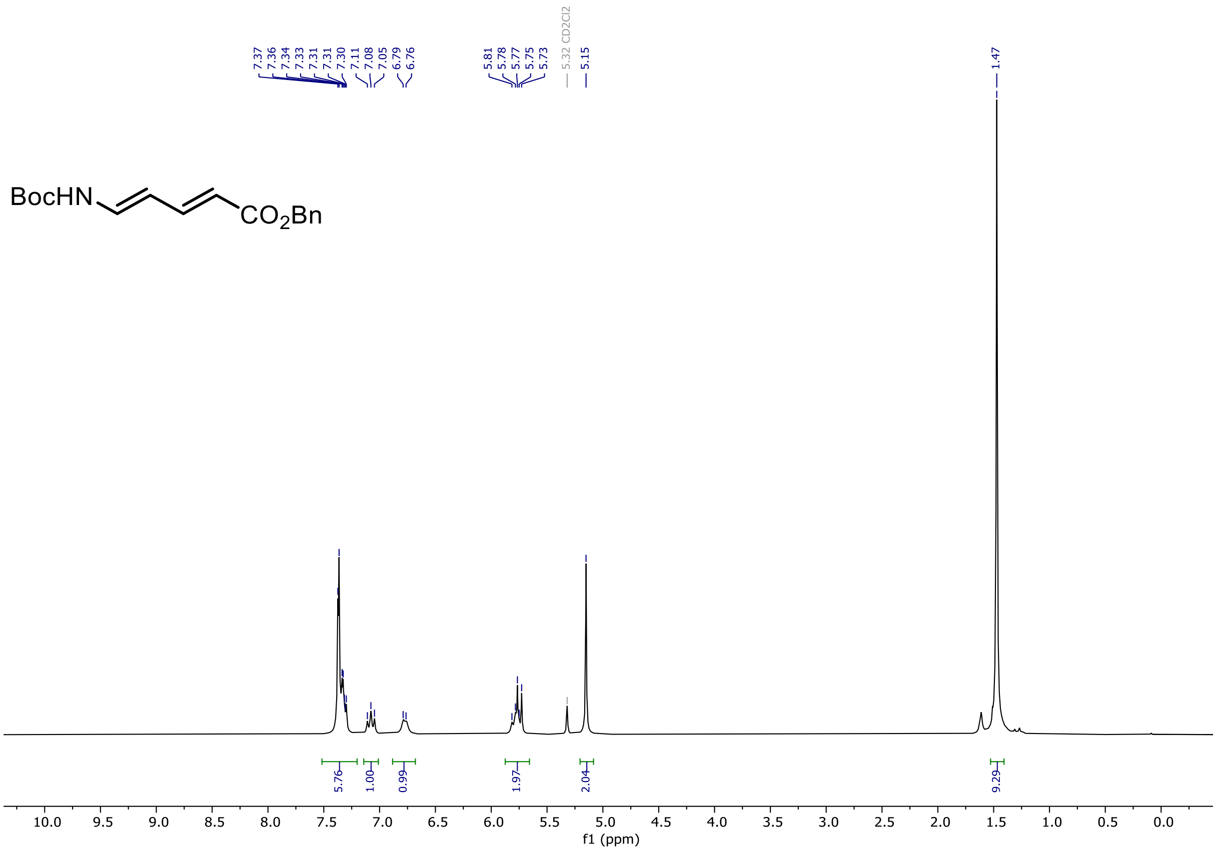

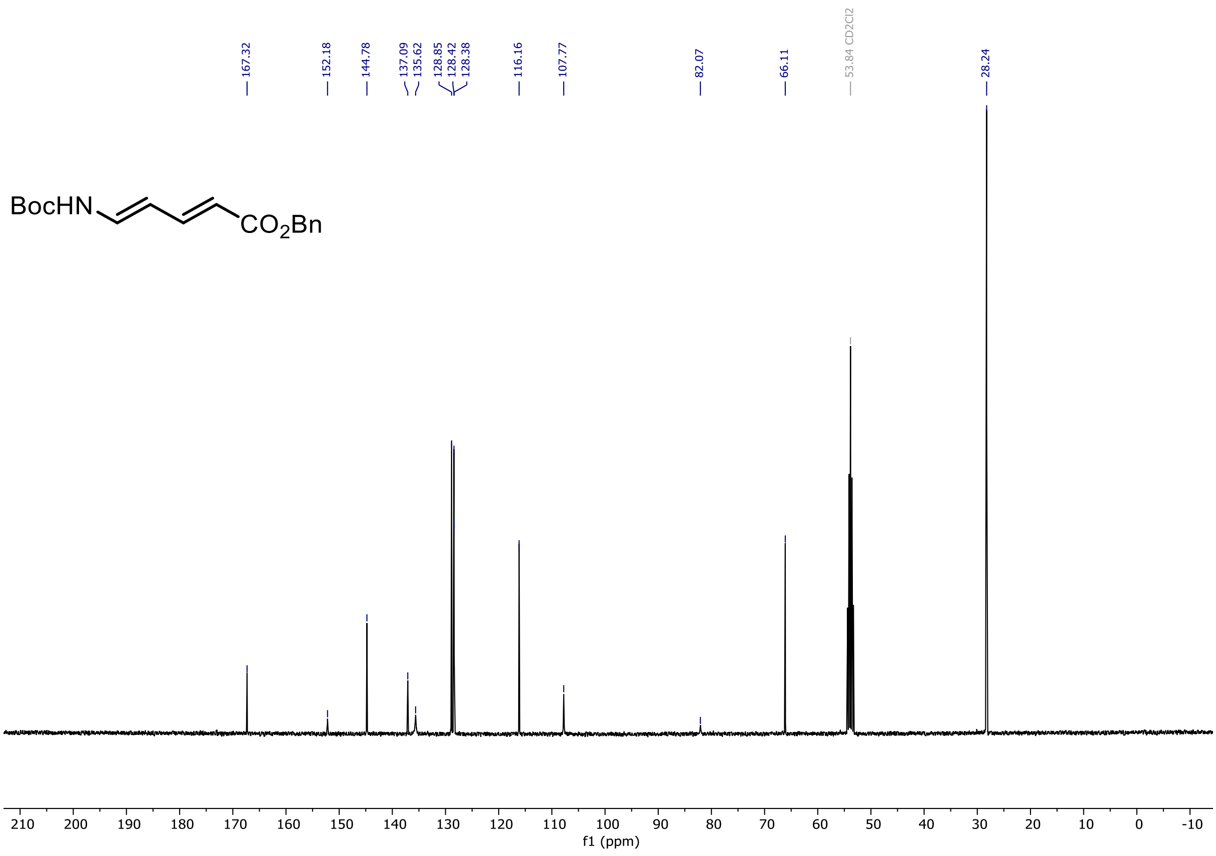

^{1}H NMR (400 MHz, CD_2Cl_2; top) and ^{13}C NMR (101 MHz, CD_2Cl_2; bottom) of compound ***(E,Z)*-13a**

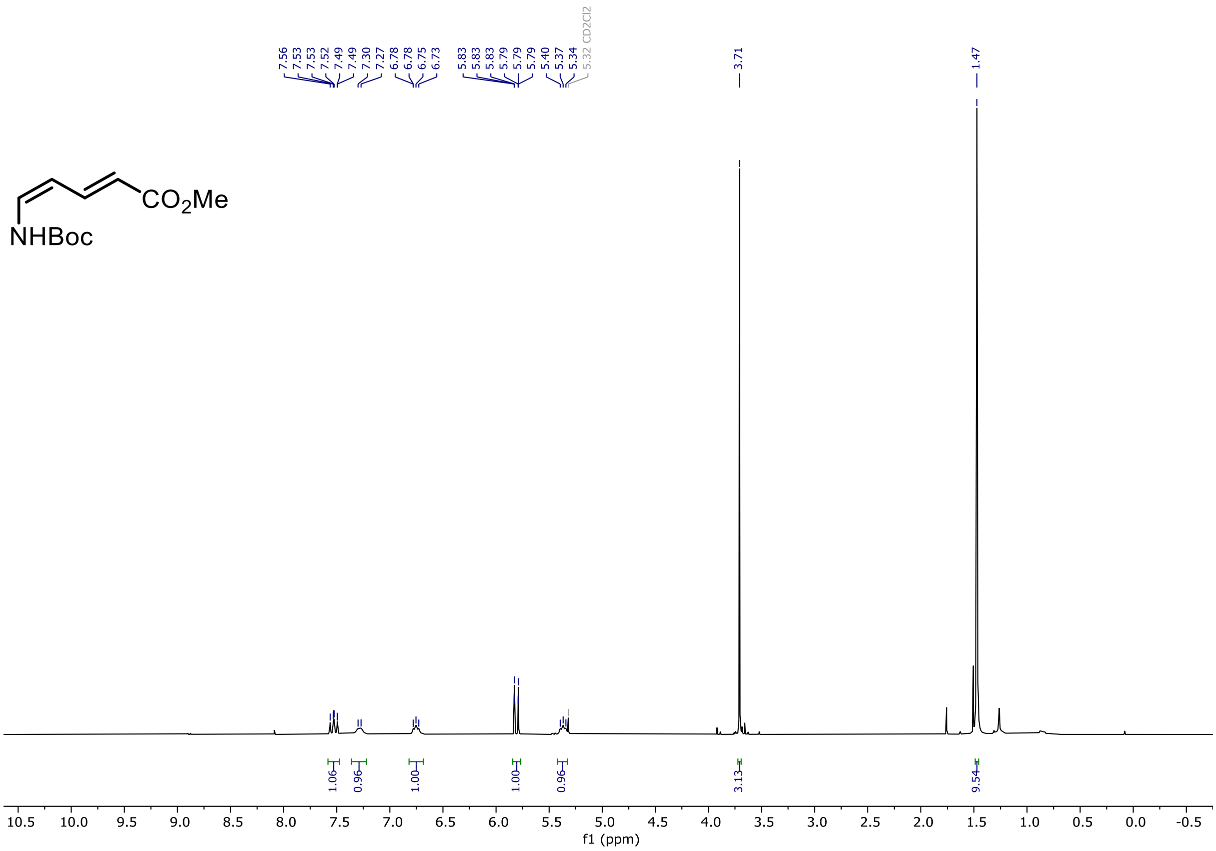

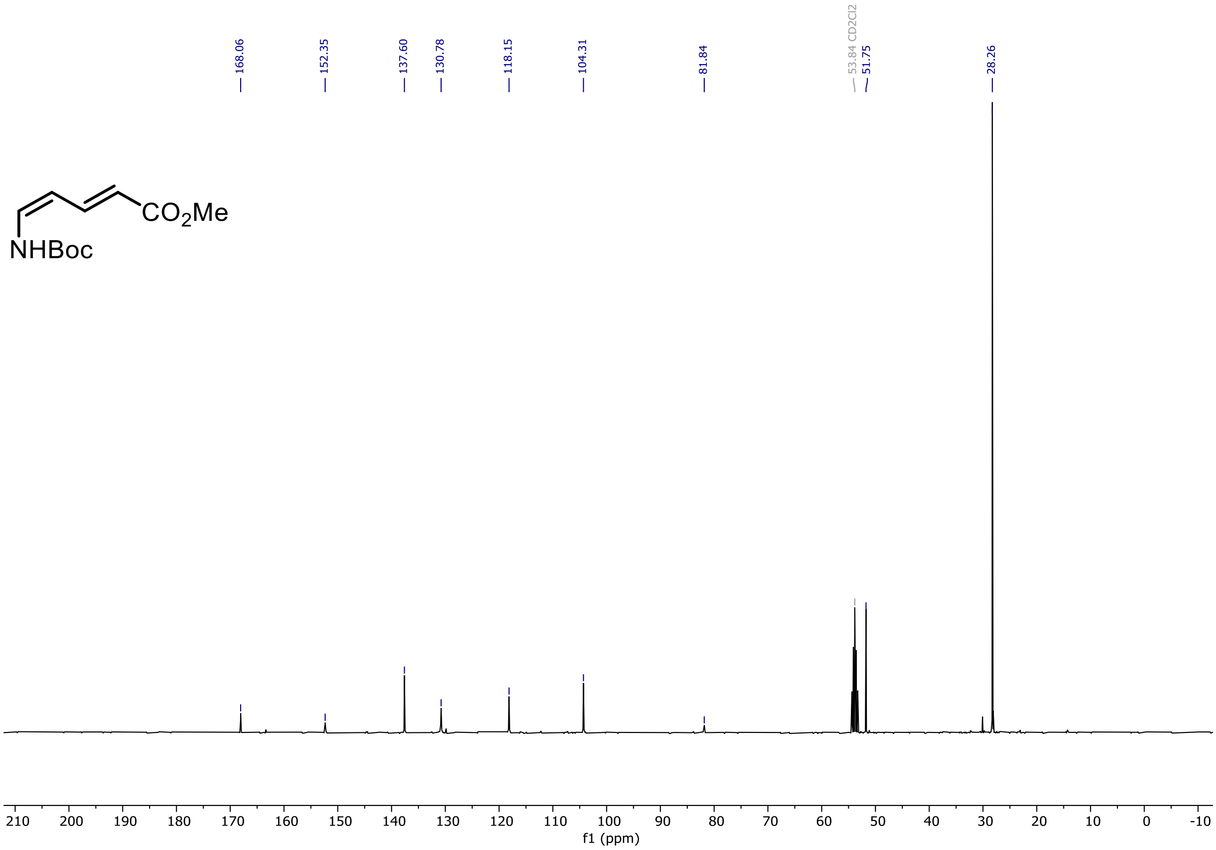

^{1}H NMR (400 MHz, $CDCl_3$; top) and ^{13}C NMR (101 MHz, $CDCl_3$; bottom) of compound **S6**

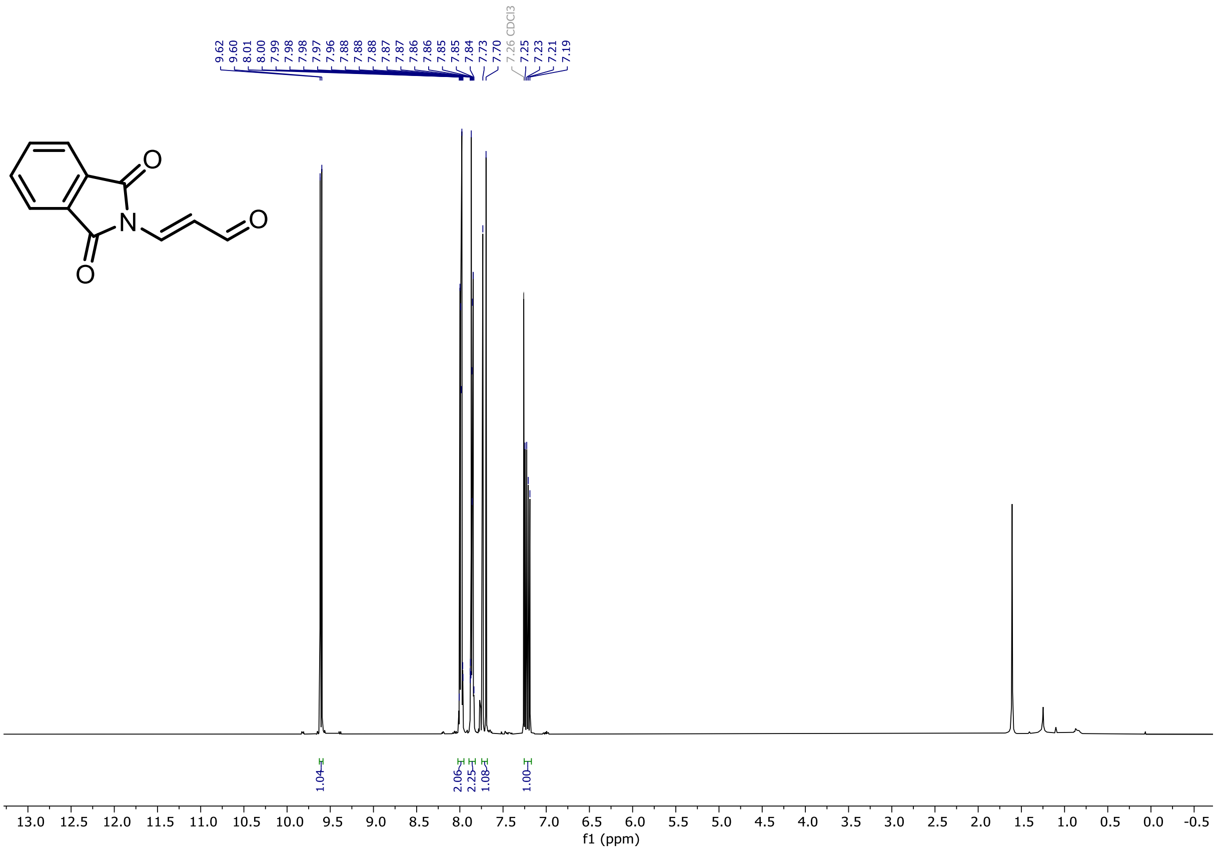

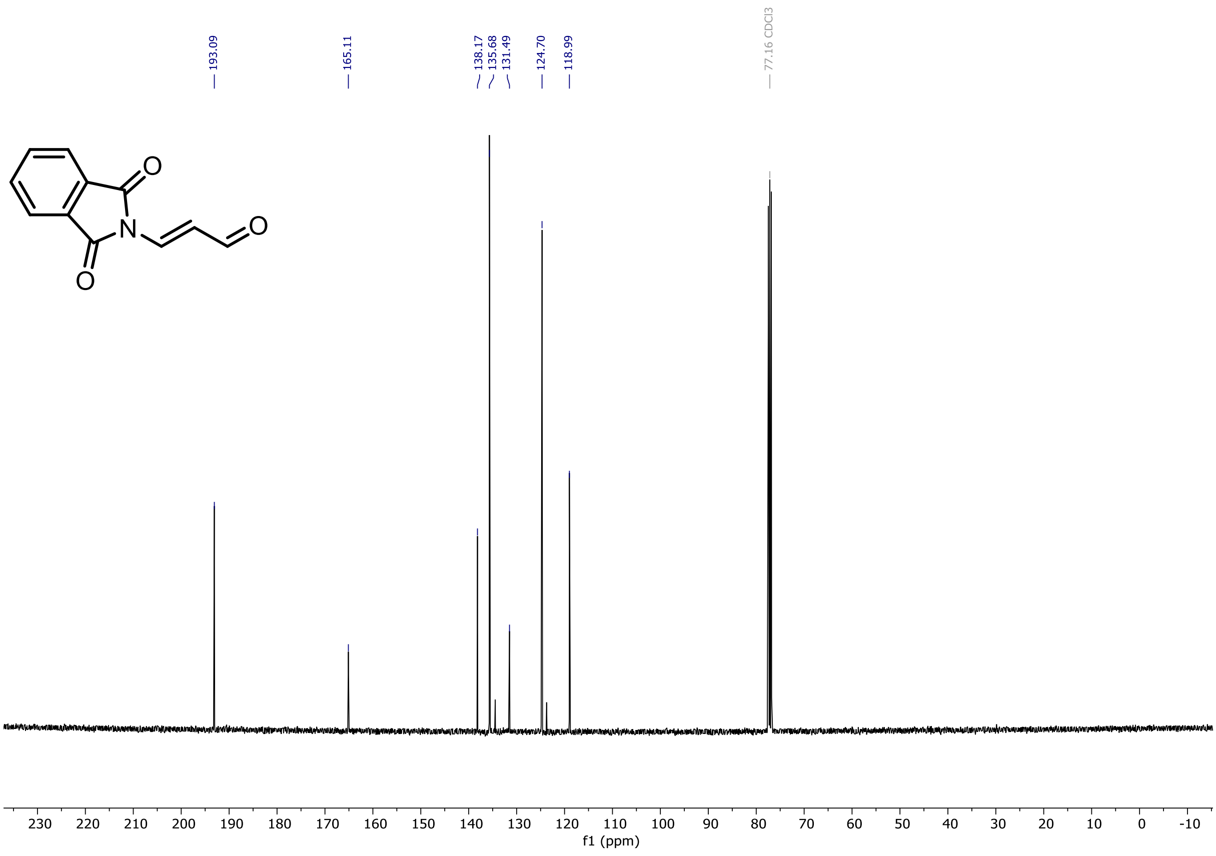

^{1}H NMR (600 MHz, CD_2Cl_2; top) and ^{13}C NMR (151 MHz, CD_2Cl_2; bottom) of compound **17a**

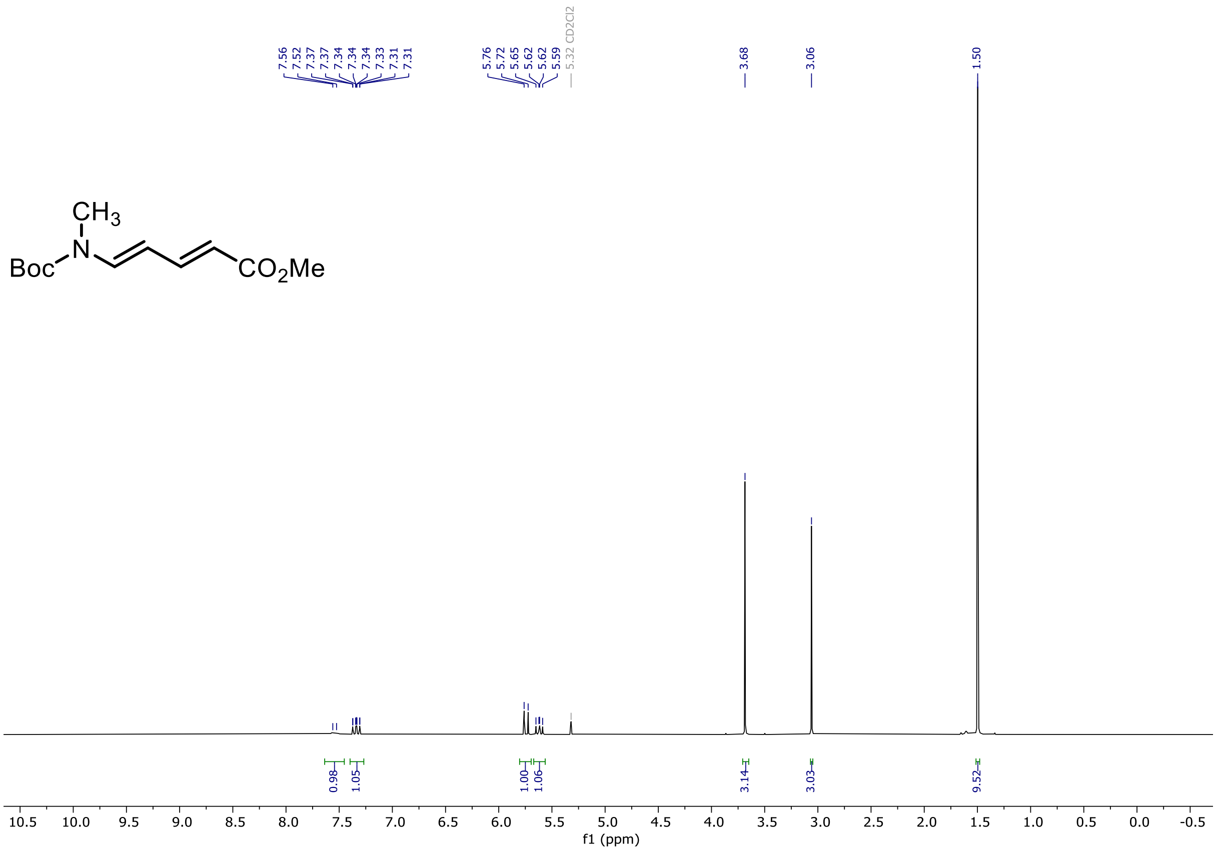

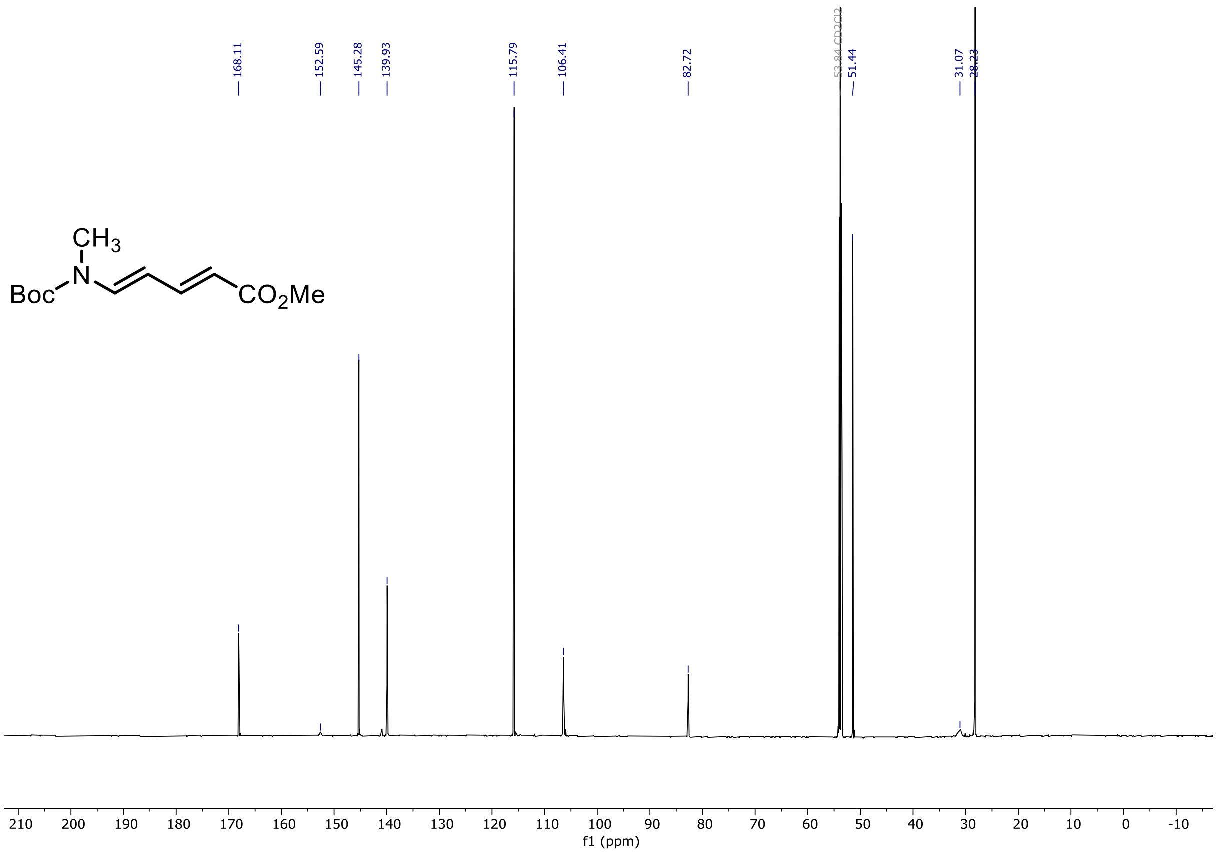

^{1}H NMR (400 MHz, CD_2Cl_2; top) and ^{13}C NMR (101 MHz, CD_2Cl_2; bottom) of compound **17b**

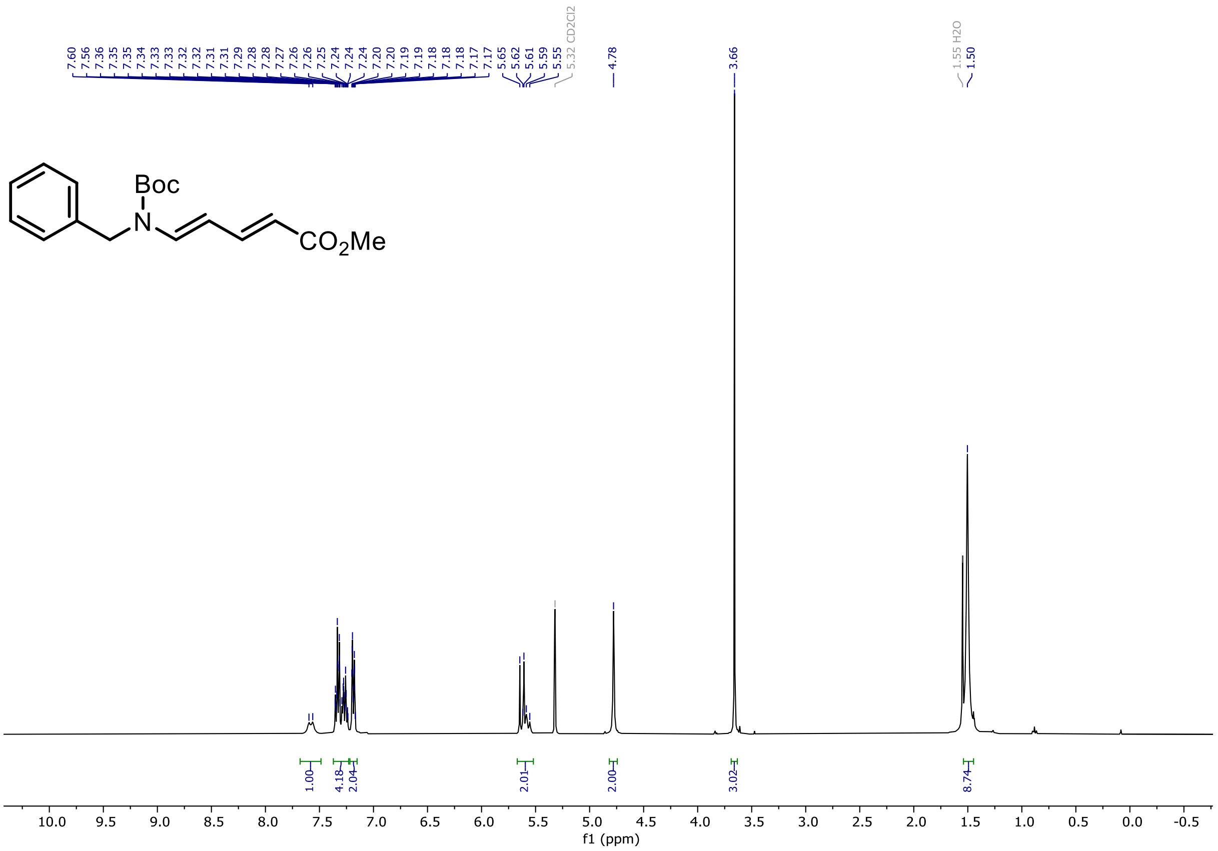

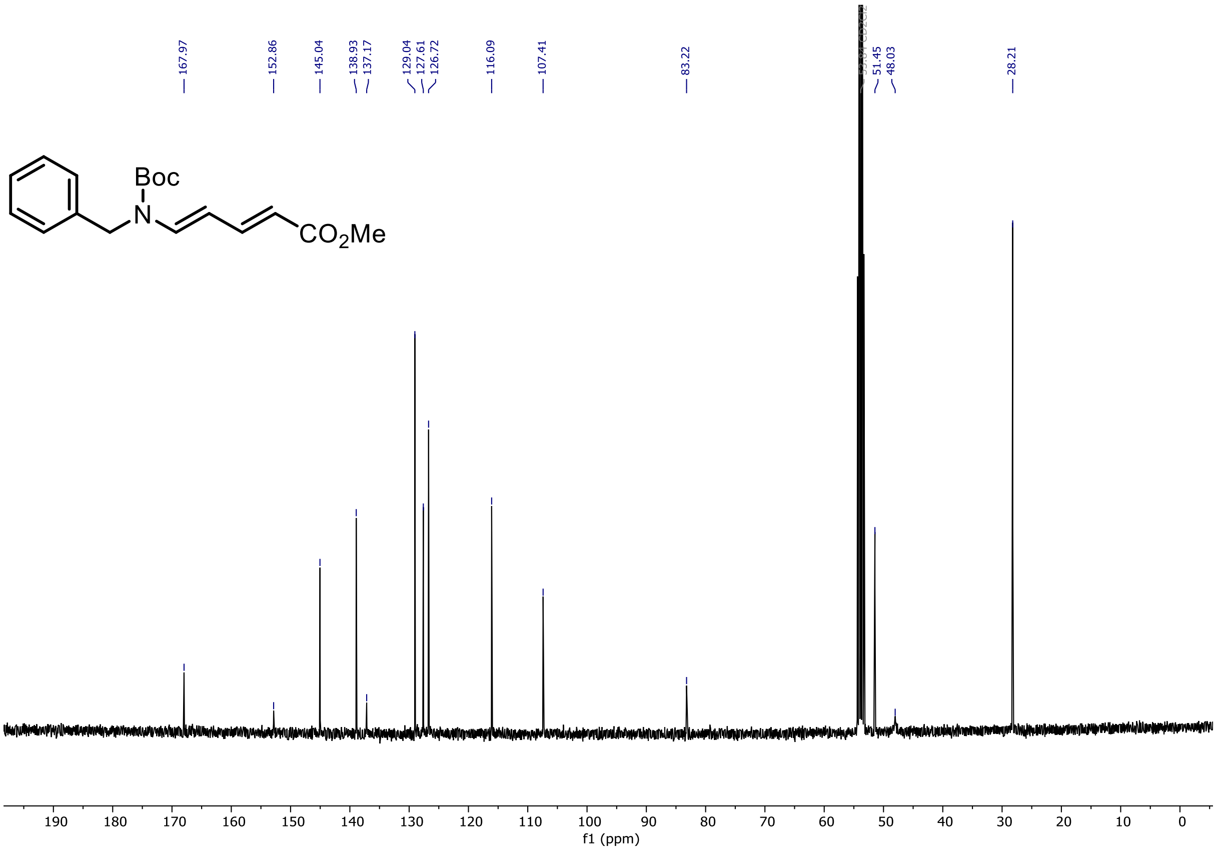

^{1}H NMR (400 MHz, $CDCl_3$; top) and ^{13}C NMR (101 MHz, $CDCl_3$; bottom) of compound **17c**

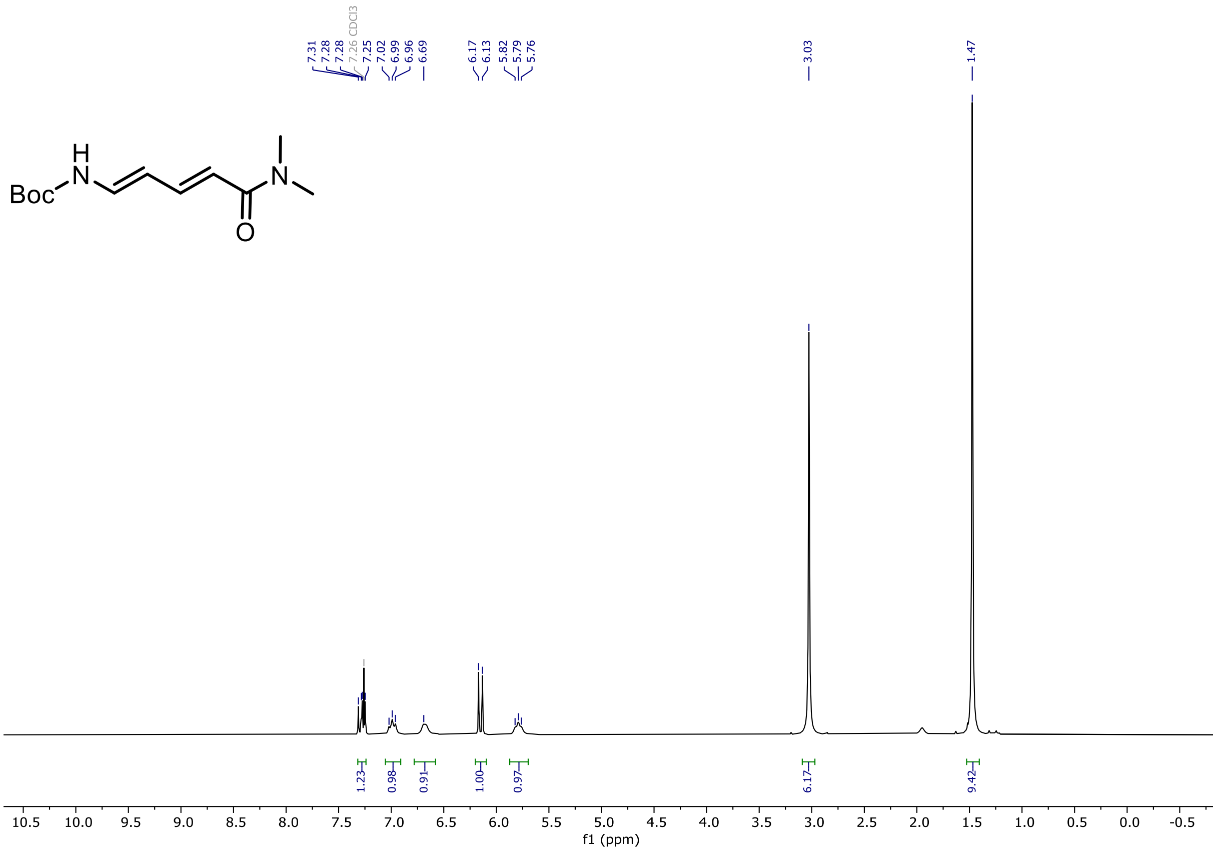

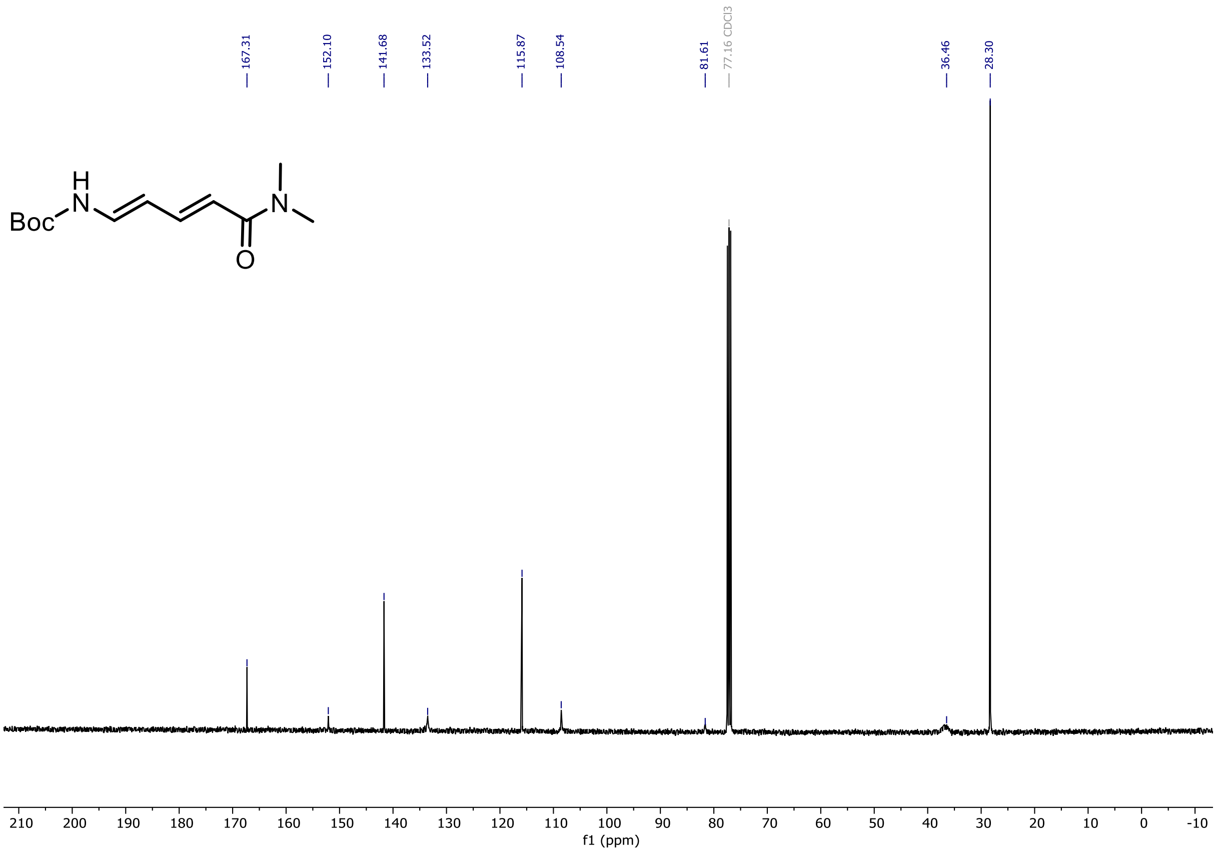

^{1}H NMR (400 MHz, CD_2Cl_2; top) and ^{13}C NMR (101 MHz, CD_2Cl_2; bottom) of compound **17d**

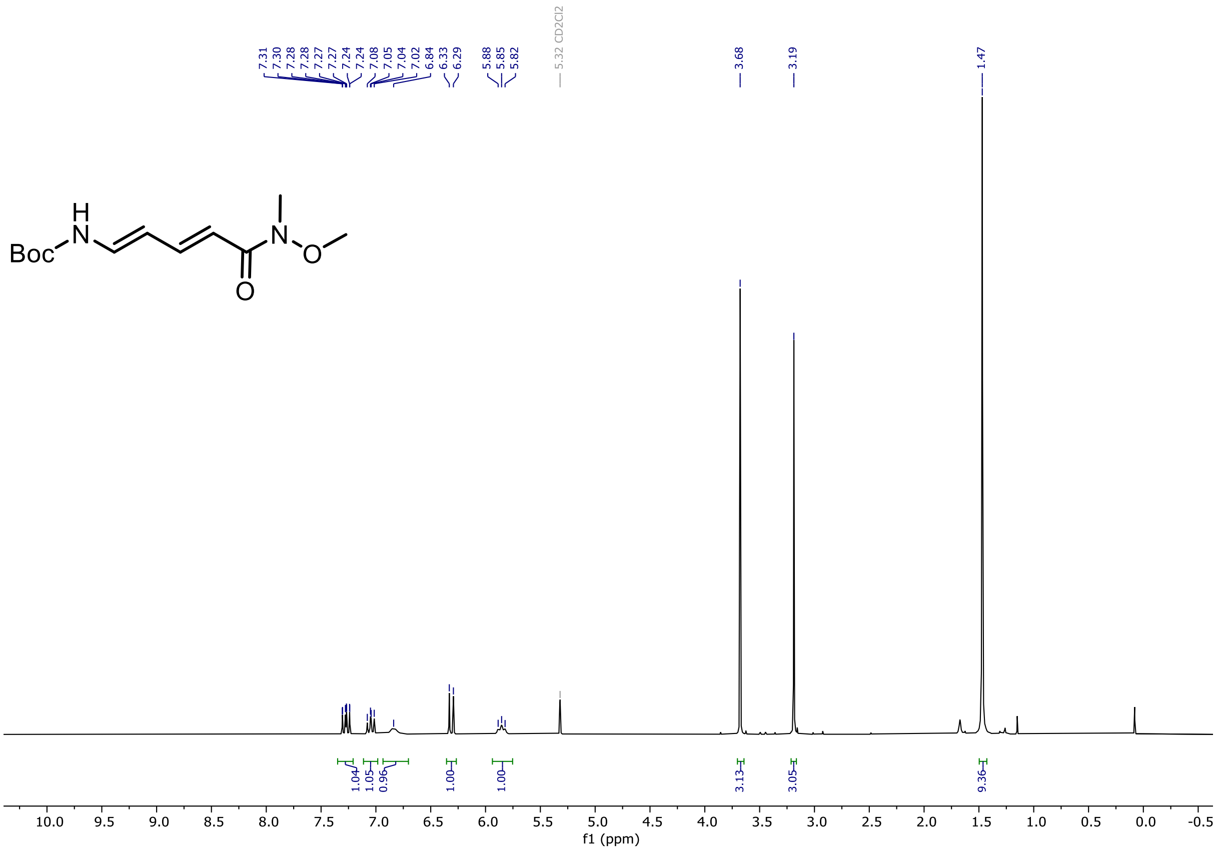

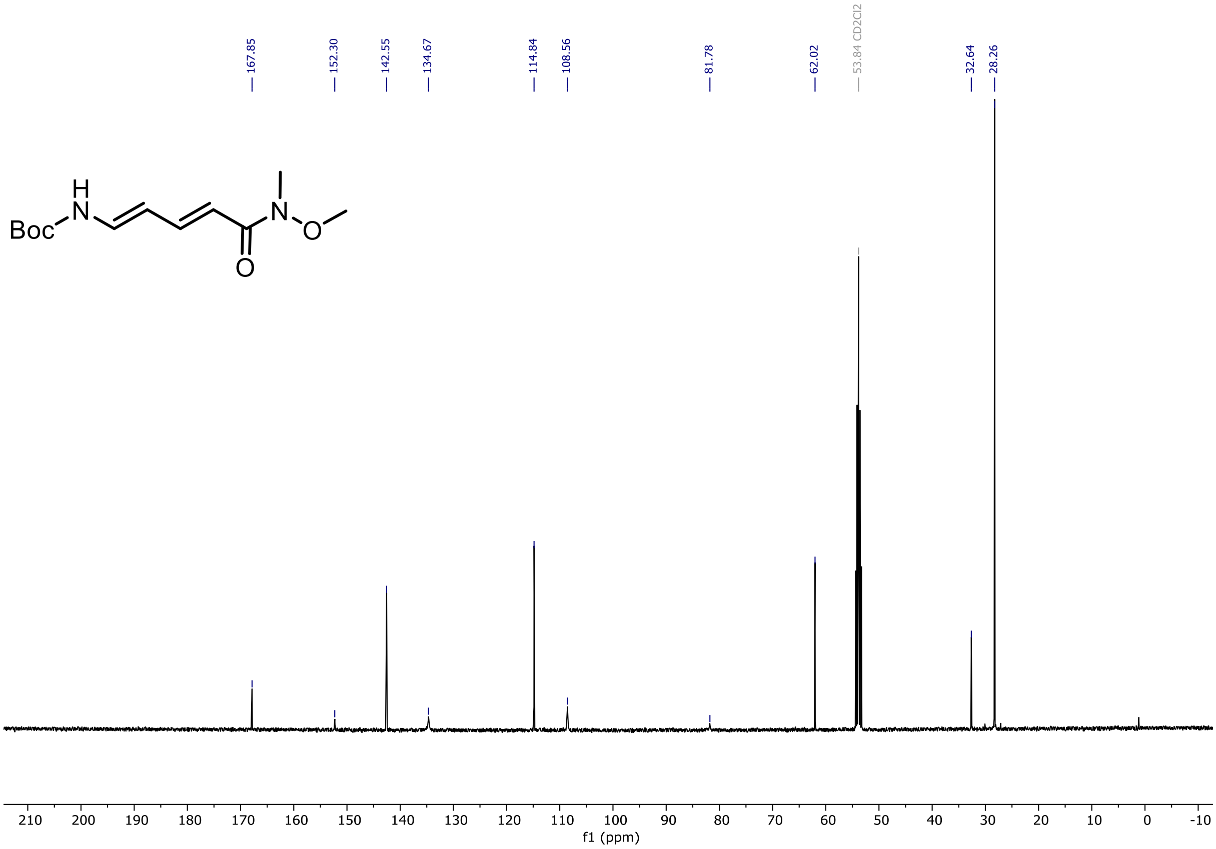

^{1}H NMR (600 MHz, [D_6]-DMSO, 393 K) and ^{13}C NMR (151 MHz, [D_6]-DMSO, 393 K) of compound **18a**

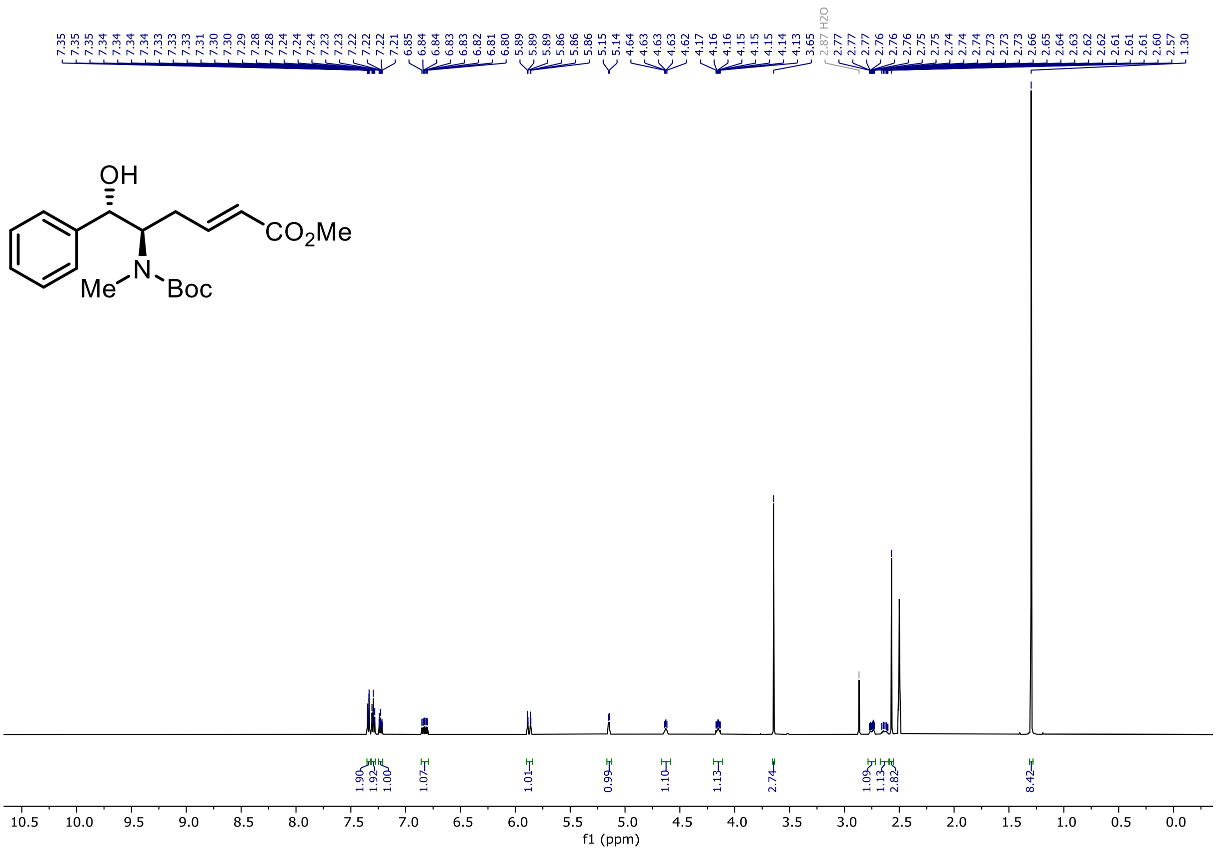

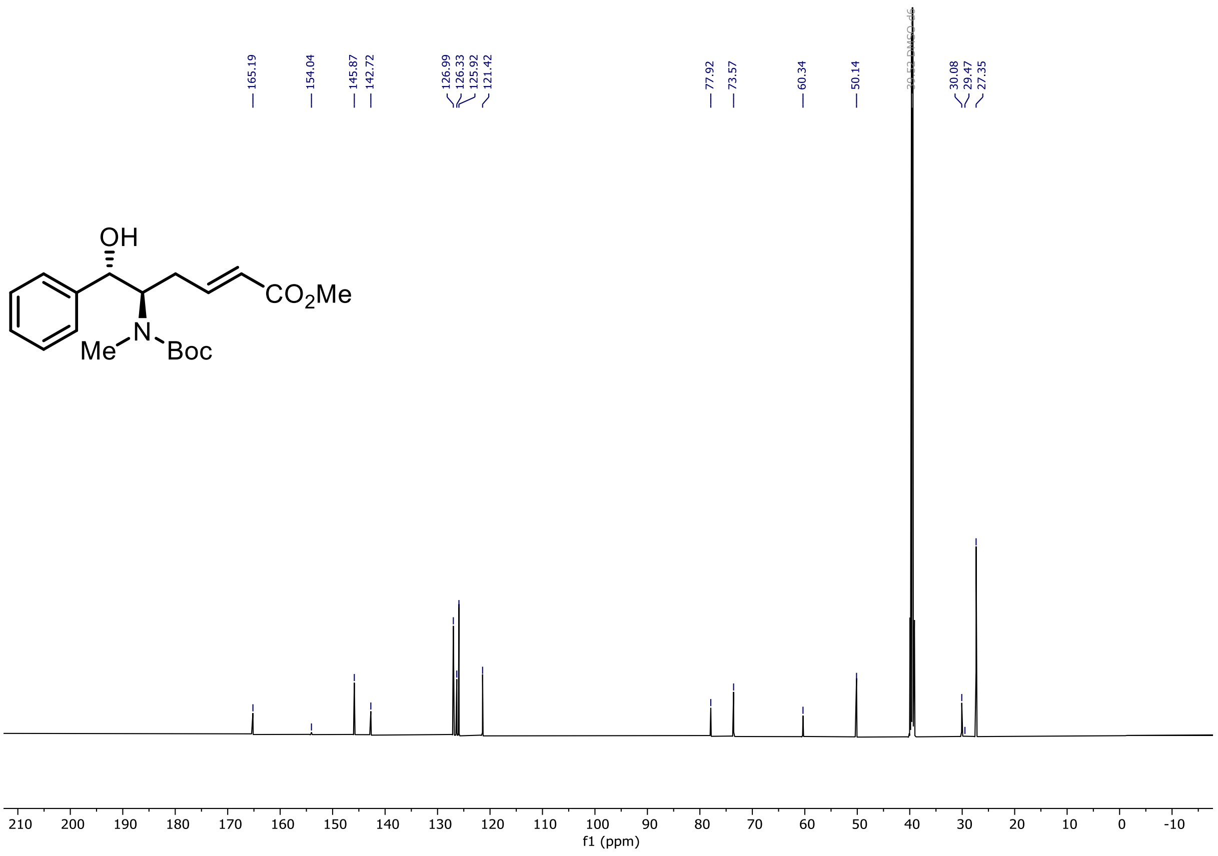

^{1}H NMR (600 MHz, [D$_6$]-DMSO, 393 K) and ^{13}C NMR (151 MHz, [D$_6$]-DMSO, 393 K) of compound **18b**

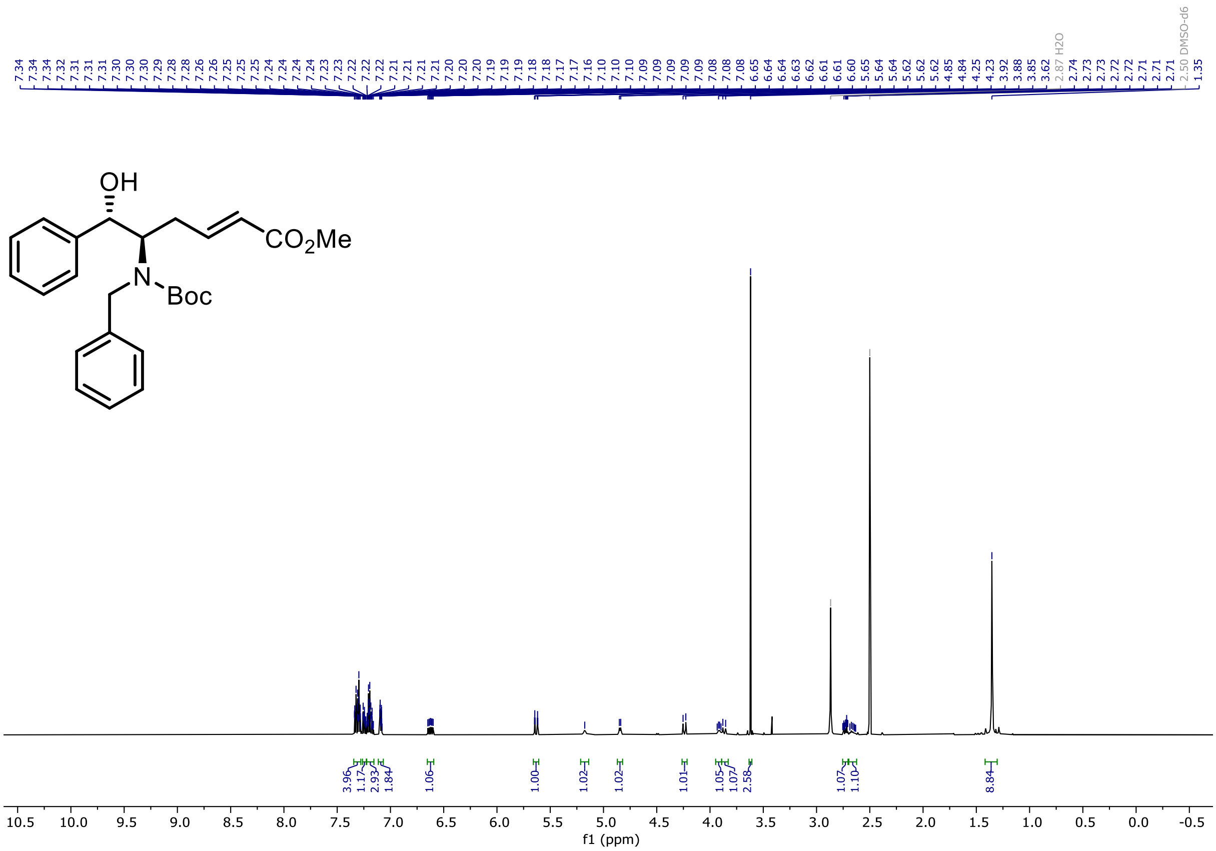

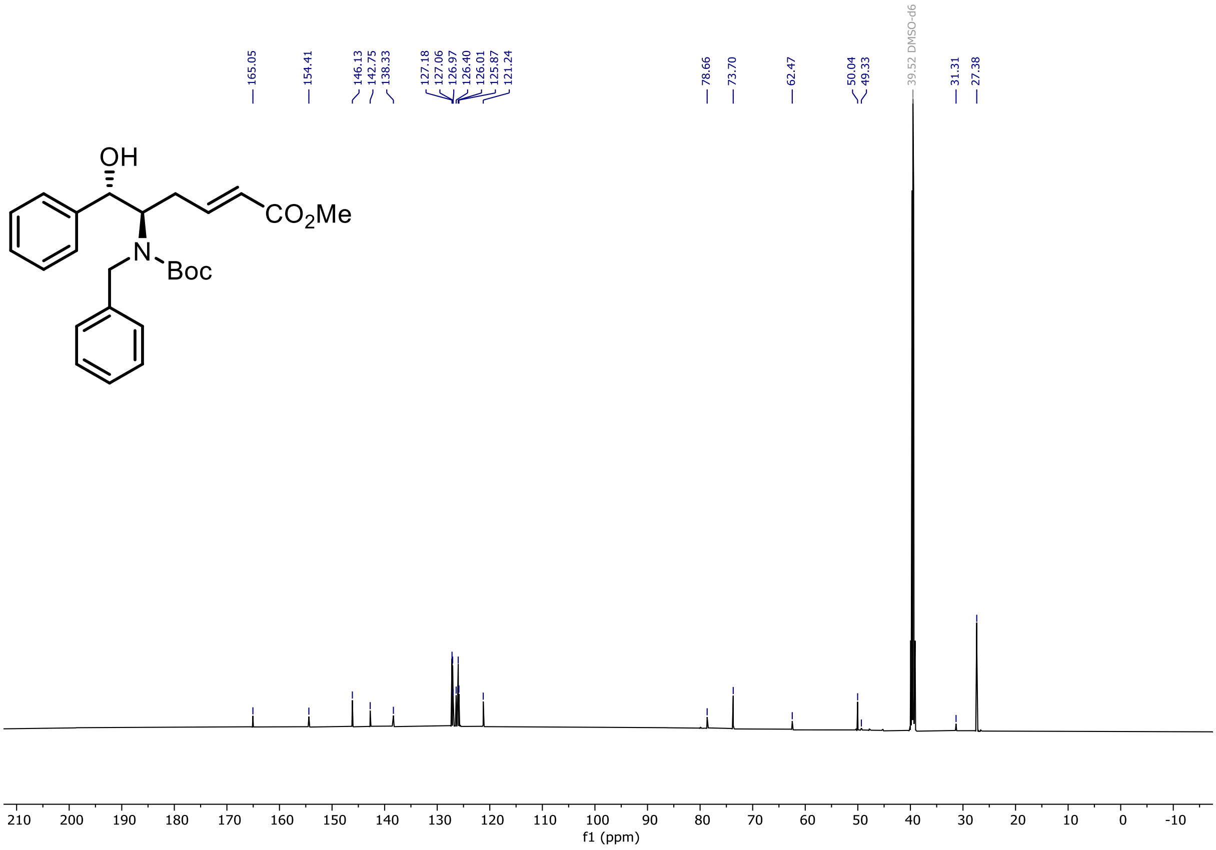

^{1}H NMR (400 MHz, CD_2Cl_2; top) and ^{13}C NMR (101 MHz, CD_2Cl_2; bottom) of compound **18c**

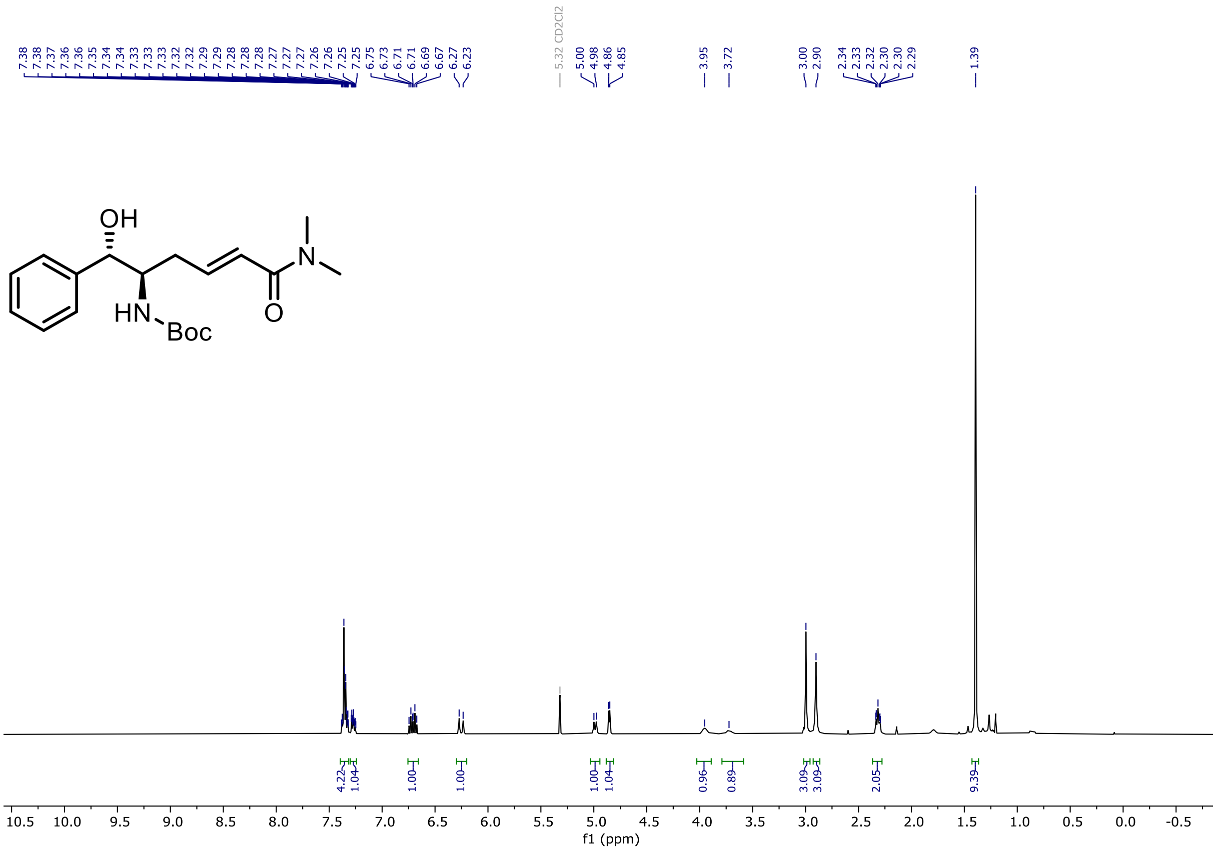

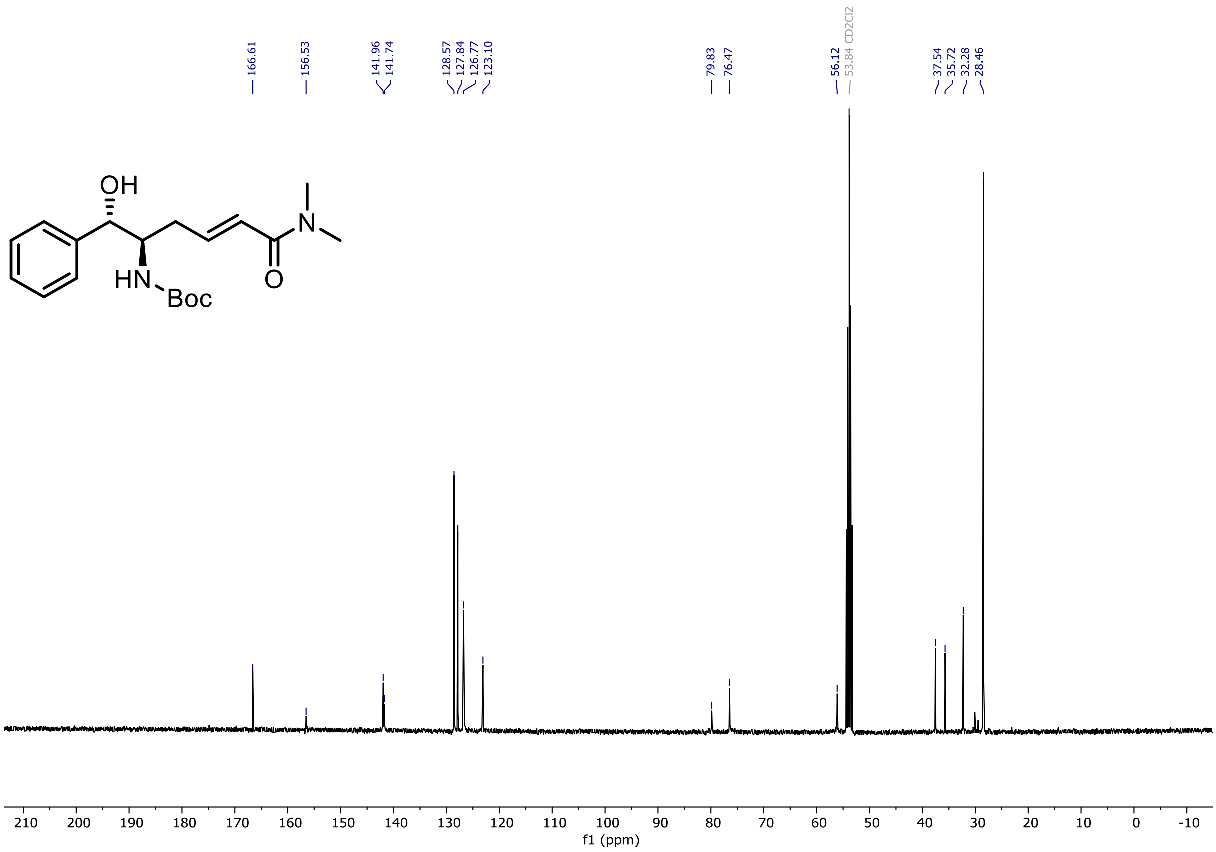

^{1}H NMR (400 MHz, CD_2Cl_2; top) and ^{13}C NMR (101 MHz, CD_2Cl_2; bottom) of compound **18d**

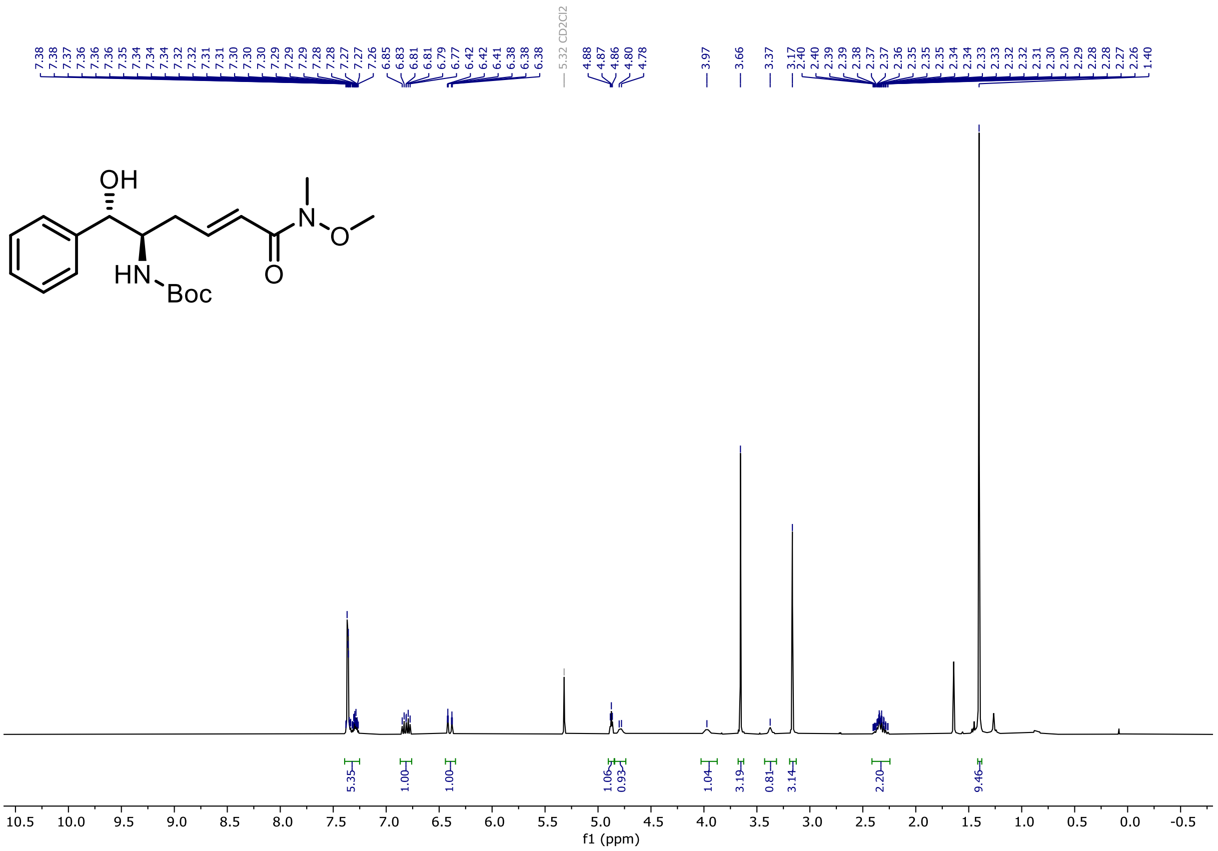

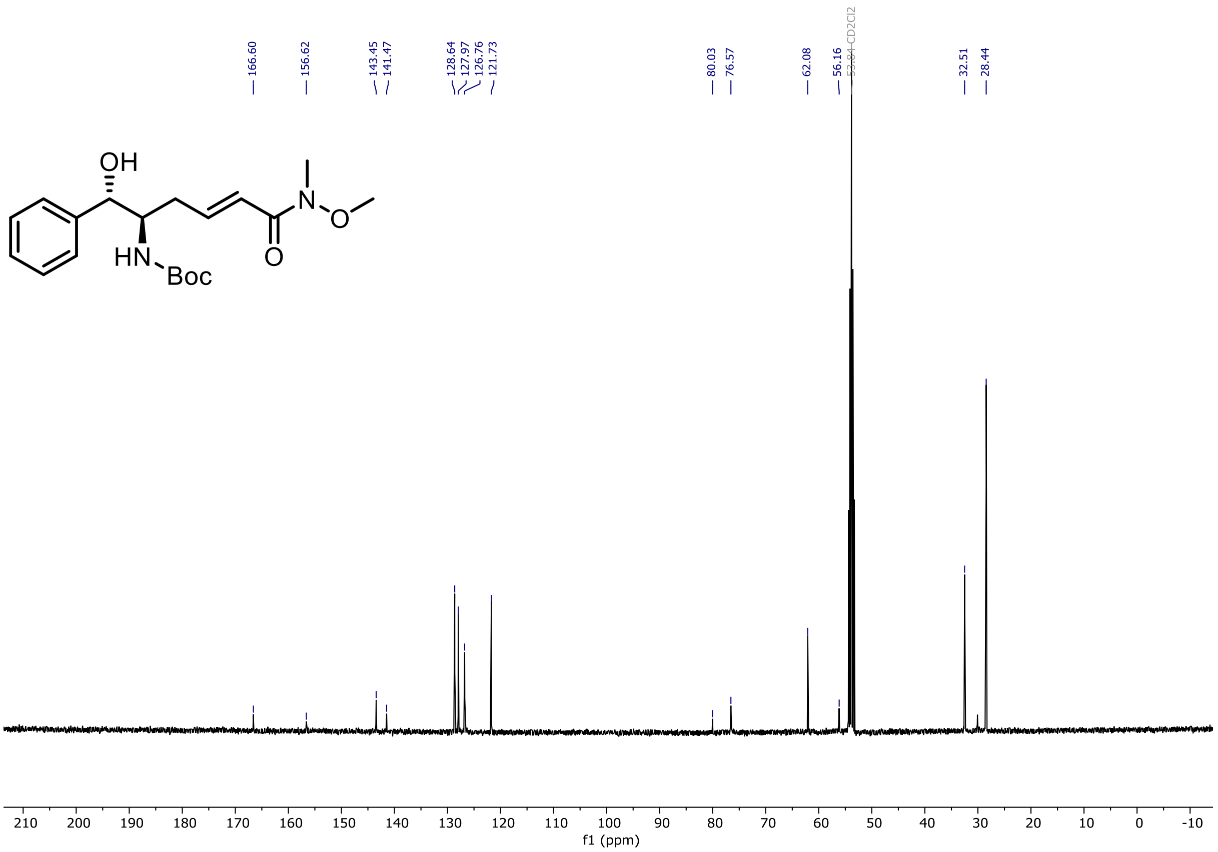

^{1}H NMR (400 MHz, $CDCl_3$; top) and ^{13}C NMR (101 MHz, $CDCl_3$; bottom) of compound **19**

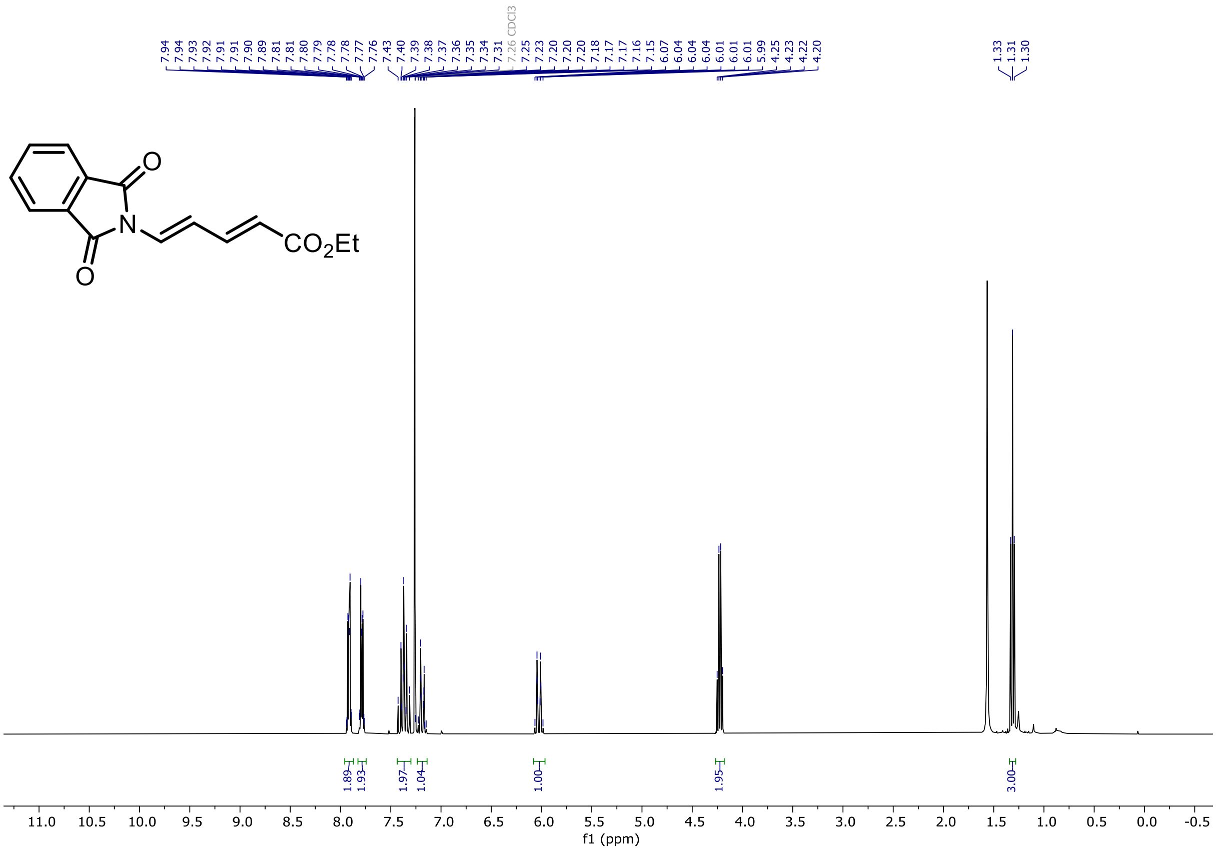

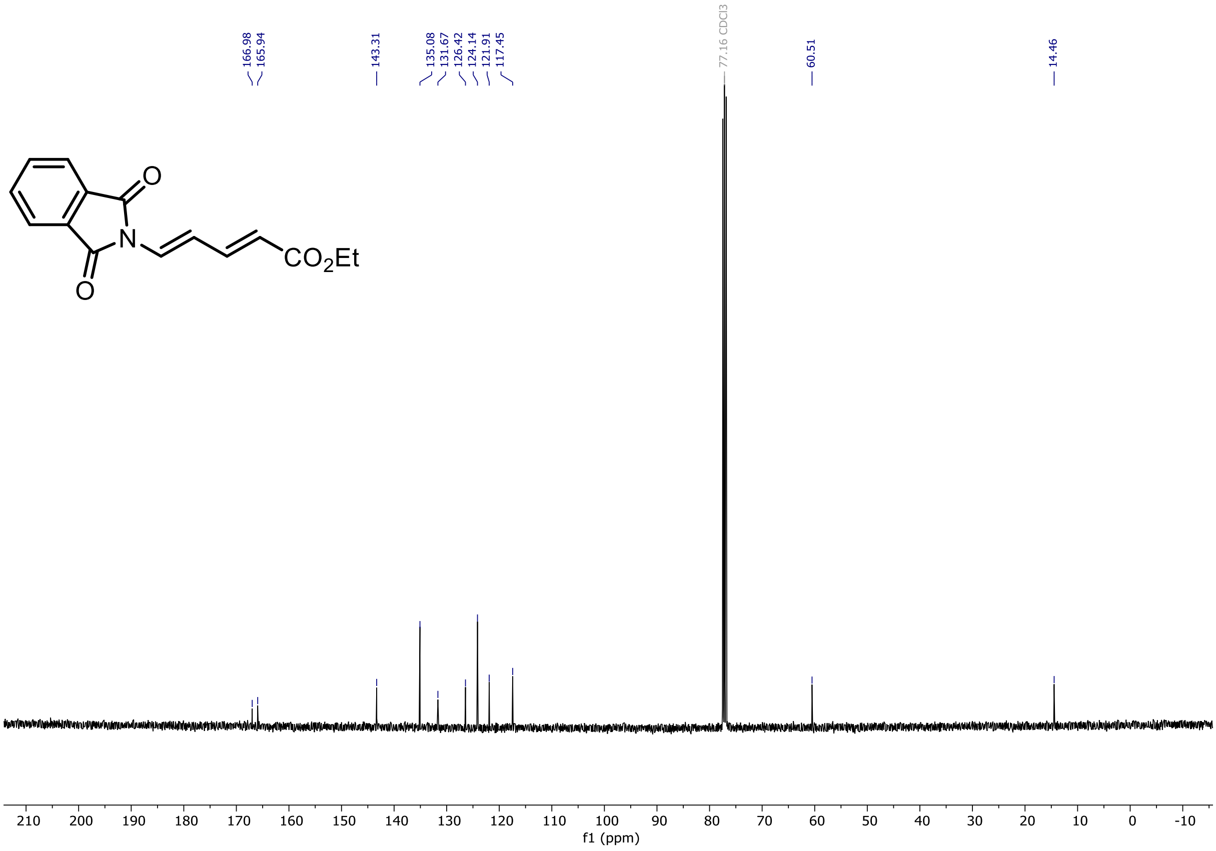

^{1}H NMR (400 MHz, C_6D_6; top) and ^{13}C NMR (101 MHz, C_6D_6; bottom) of compound **L2**

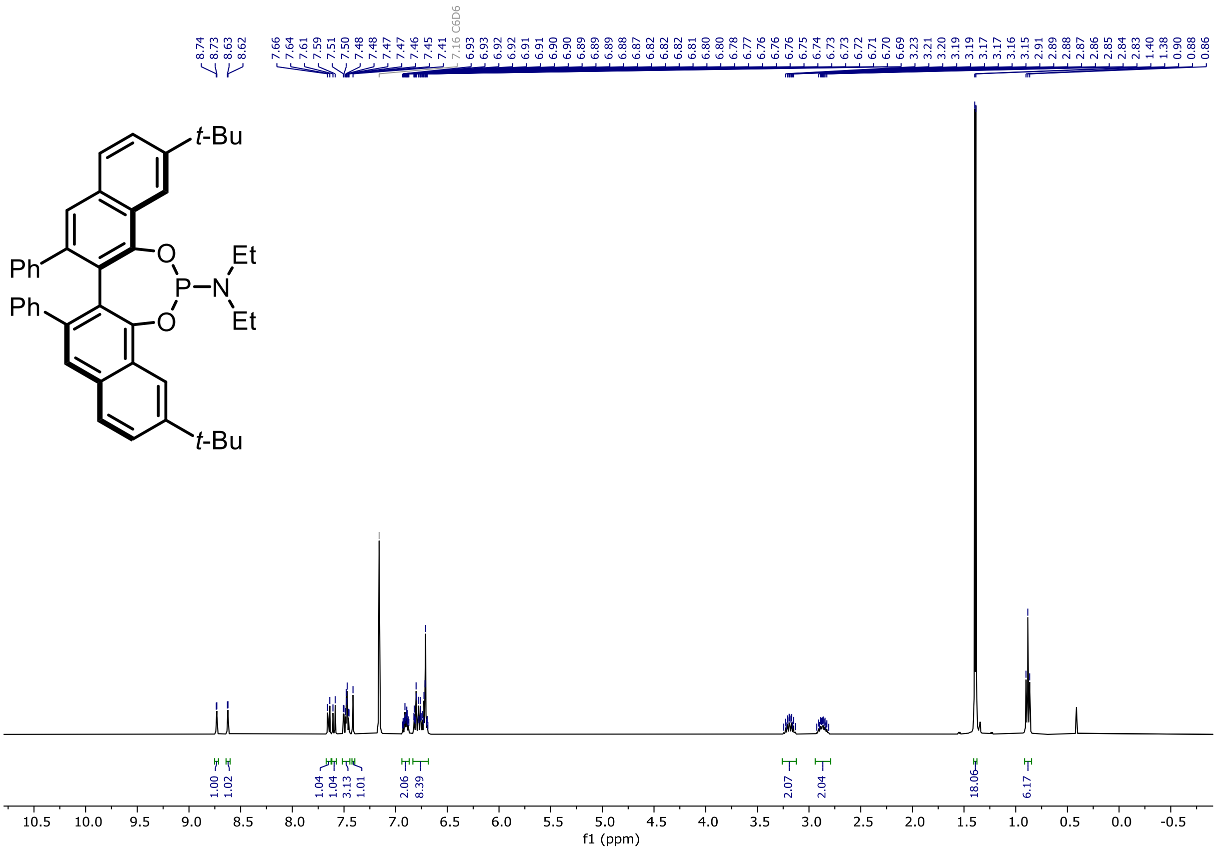

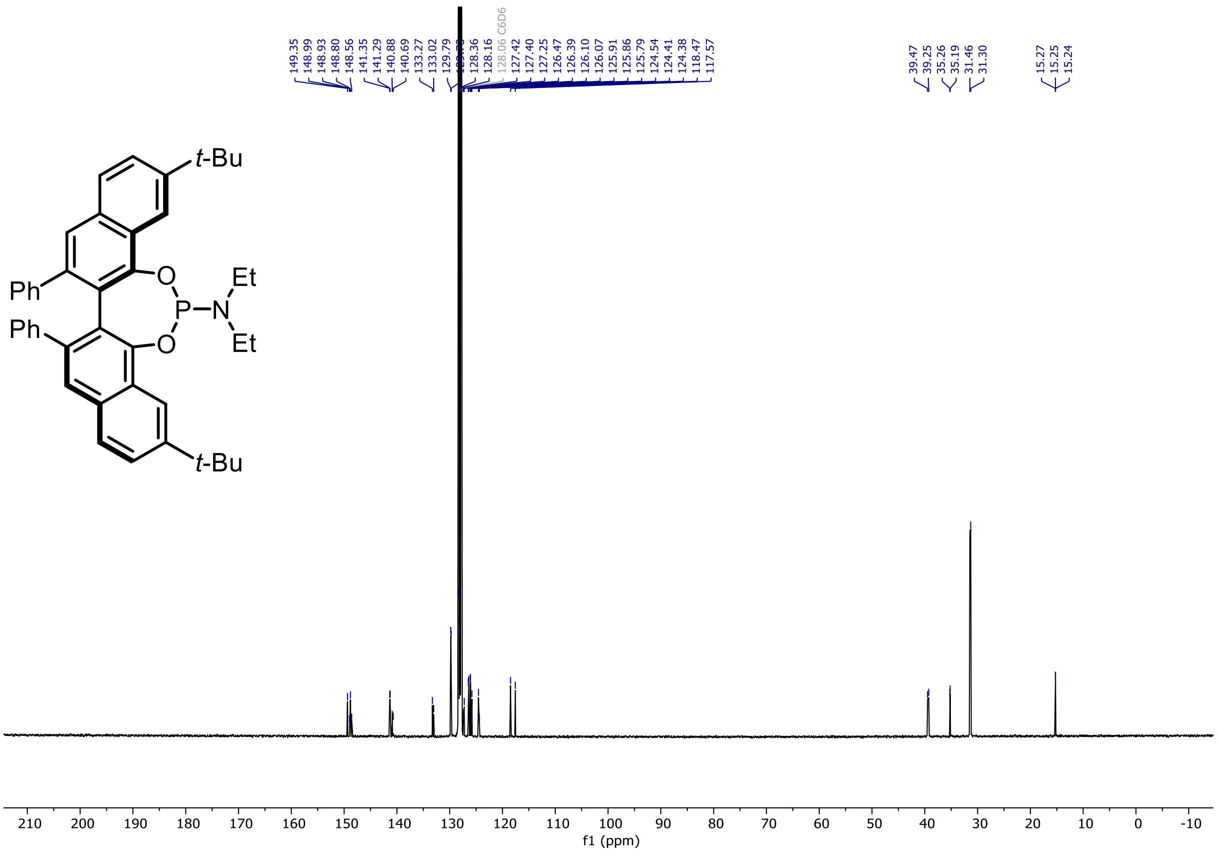

^{31}P NMR (162 MHz, C_6D_6) of compound **L2**

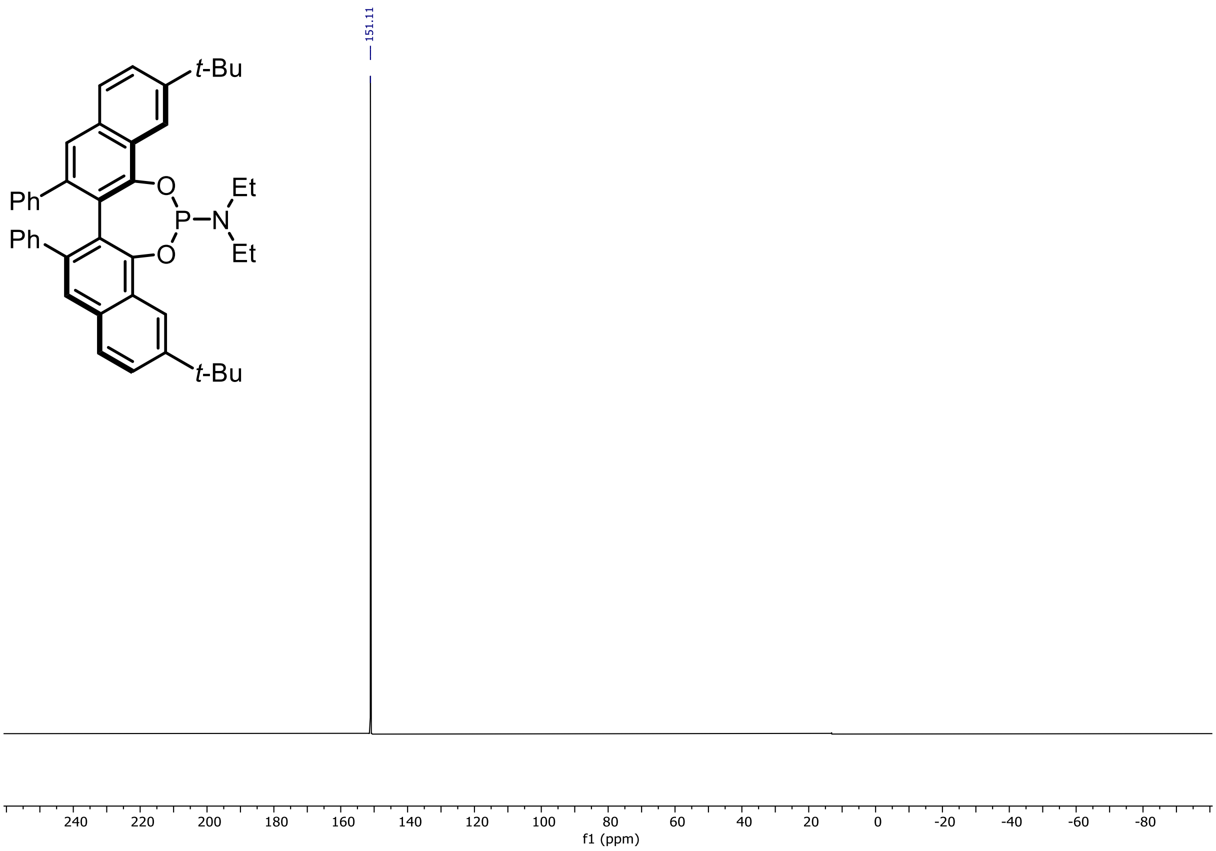

^{1}H NMR (400 MHz, CD_2Cl_2; top) and ^{13}C NMR (101 MHz, CD_2Cl_2; bottom) of compound ***rac*-14**

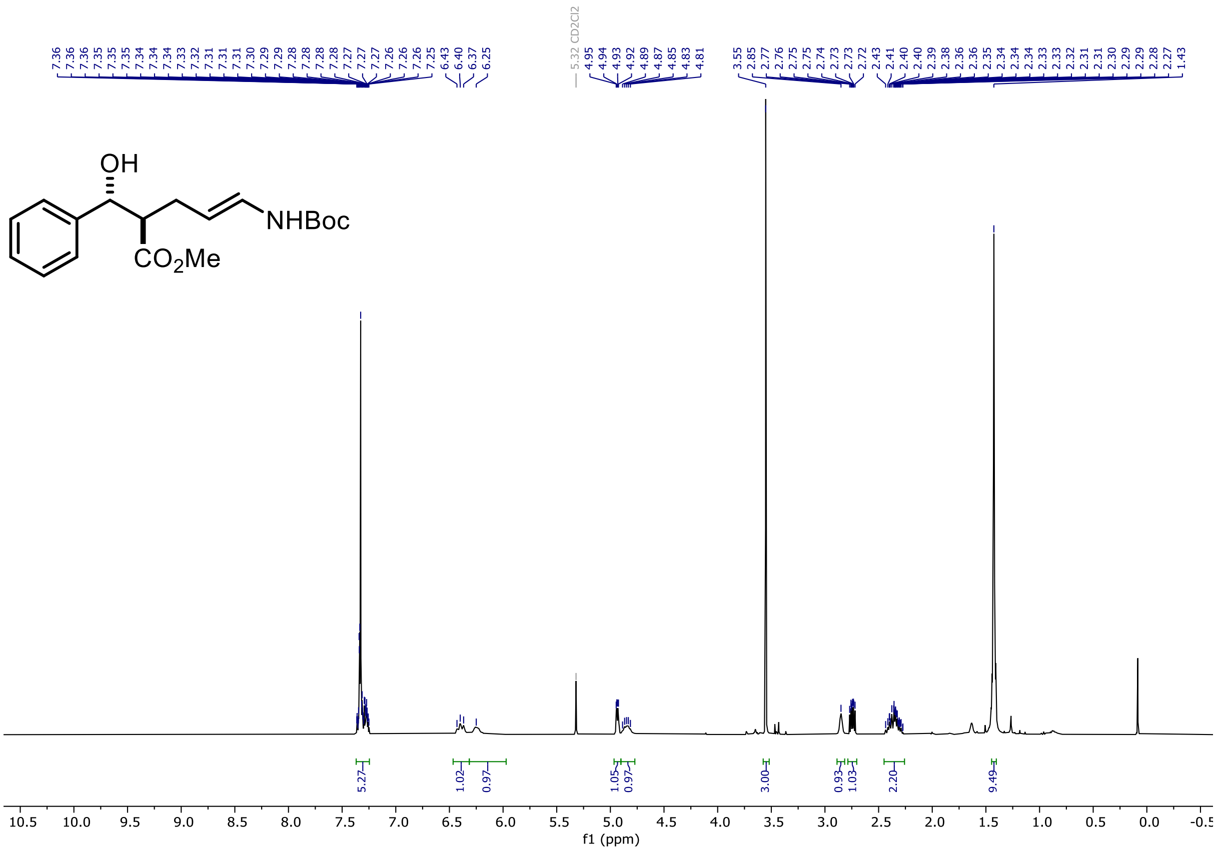

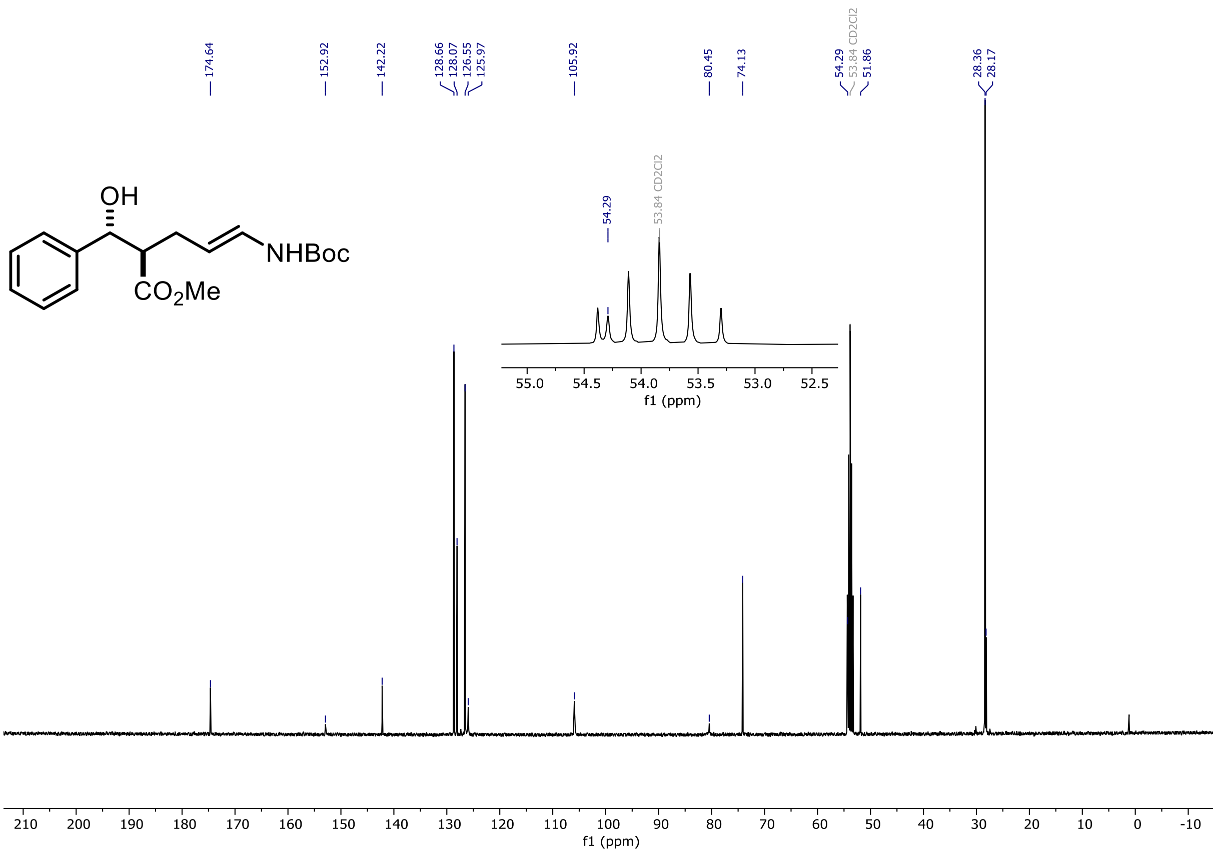

^{1}H NMR (400 MHz, CD_2Cl_2; top) and ^{13}C NMR (101 MHz, CD_2Cl_2; bottom) of compound **15a**

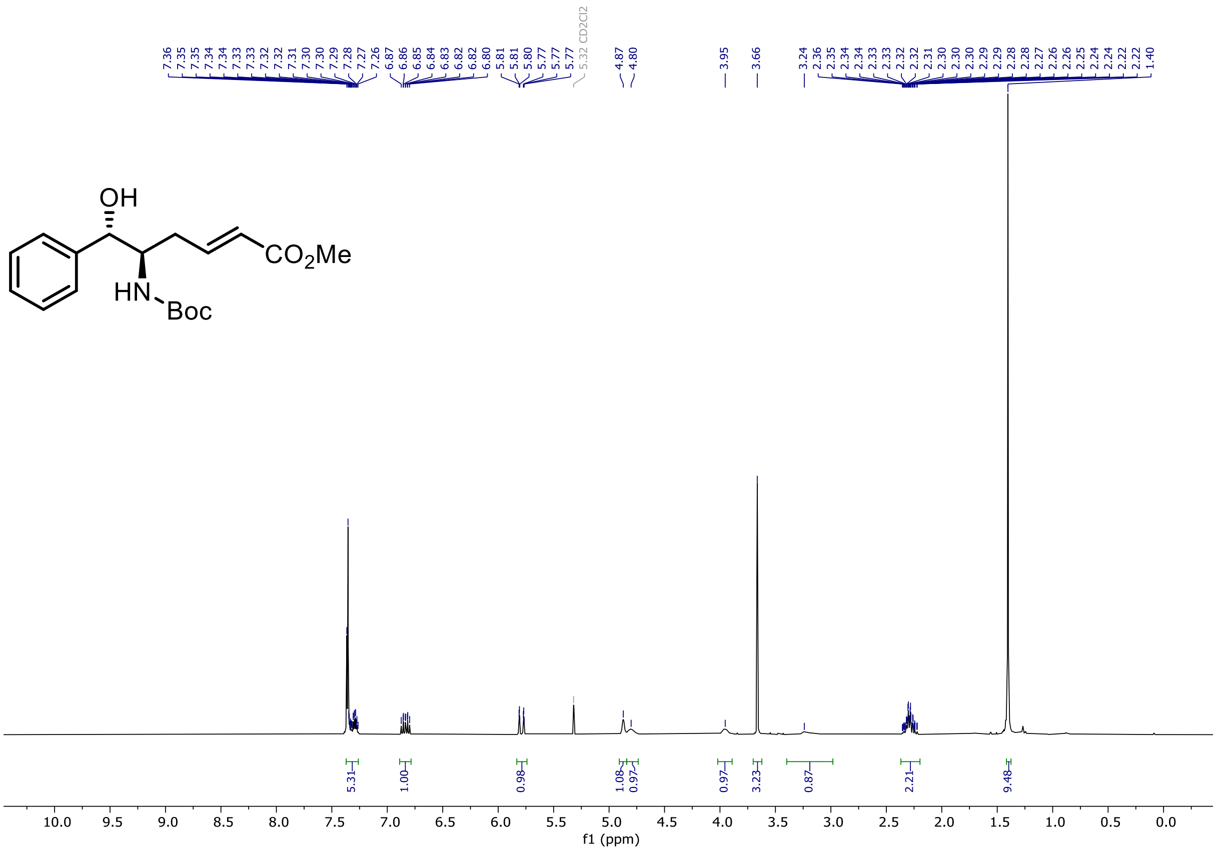

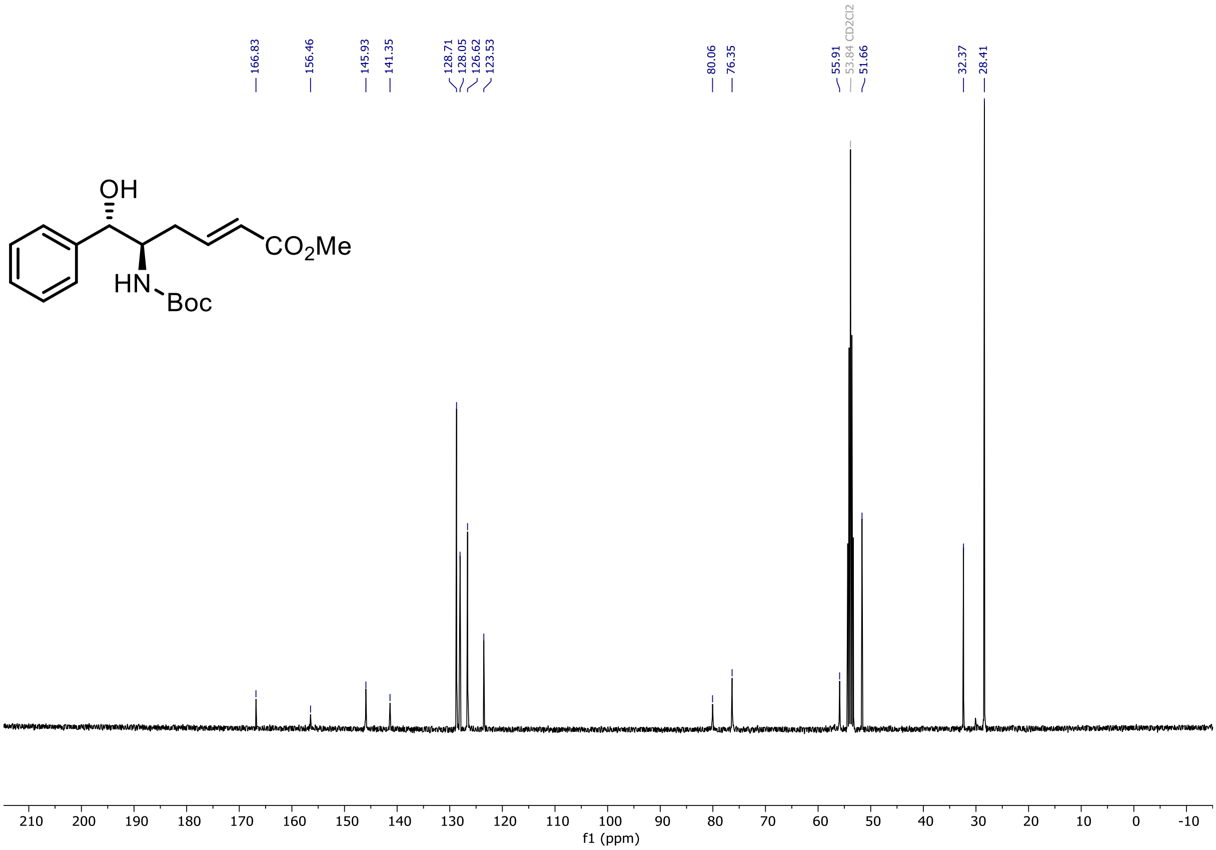

^1H NMR (400 MHz, CD_2Cl_2; top) and ^{13}C NMR (101 MHz, CD_2Cl_2; bottom) of compound **15b**

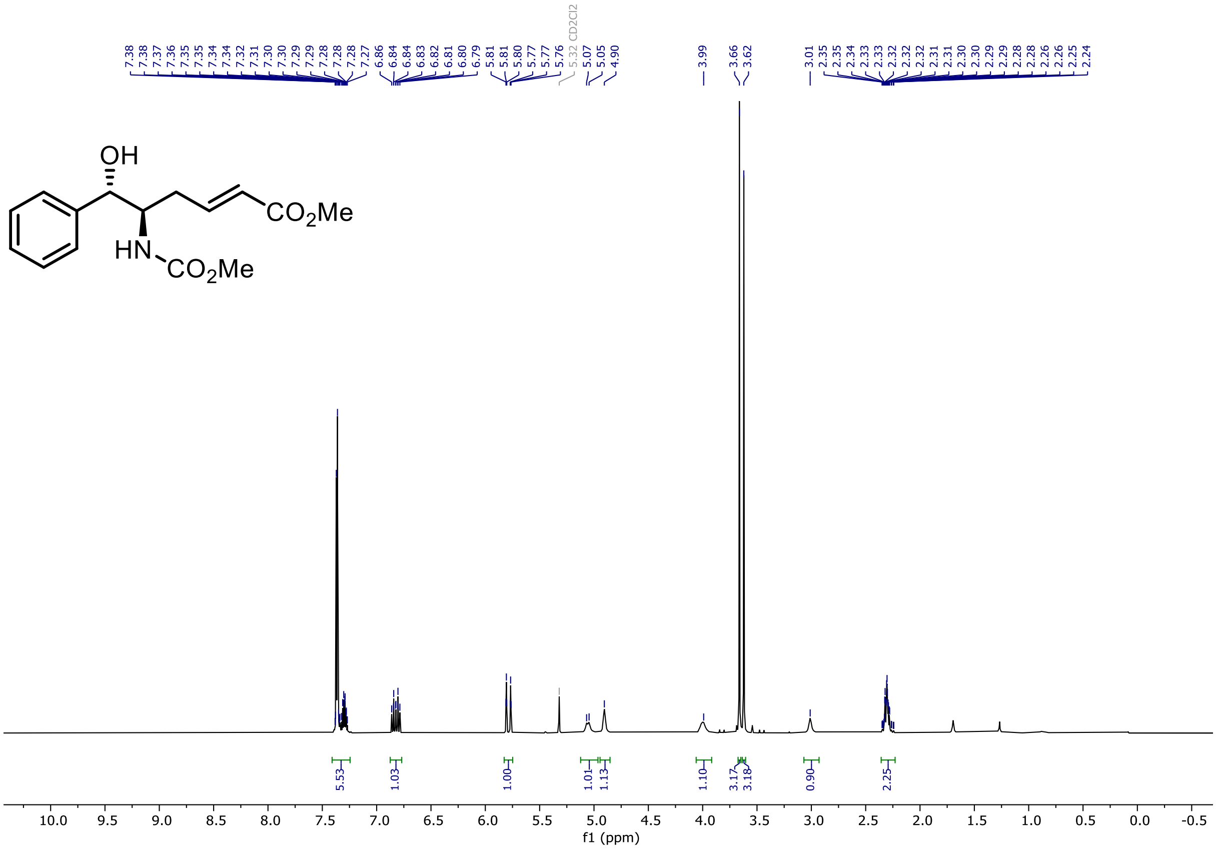

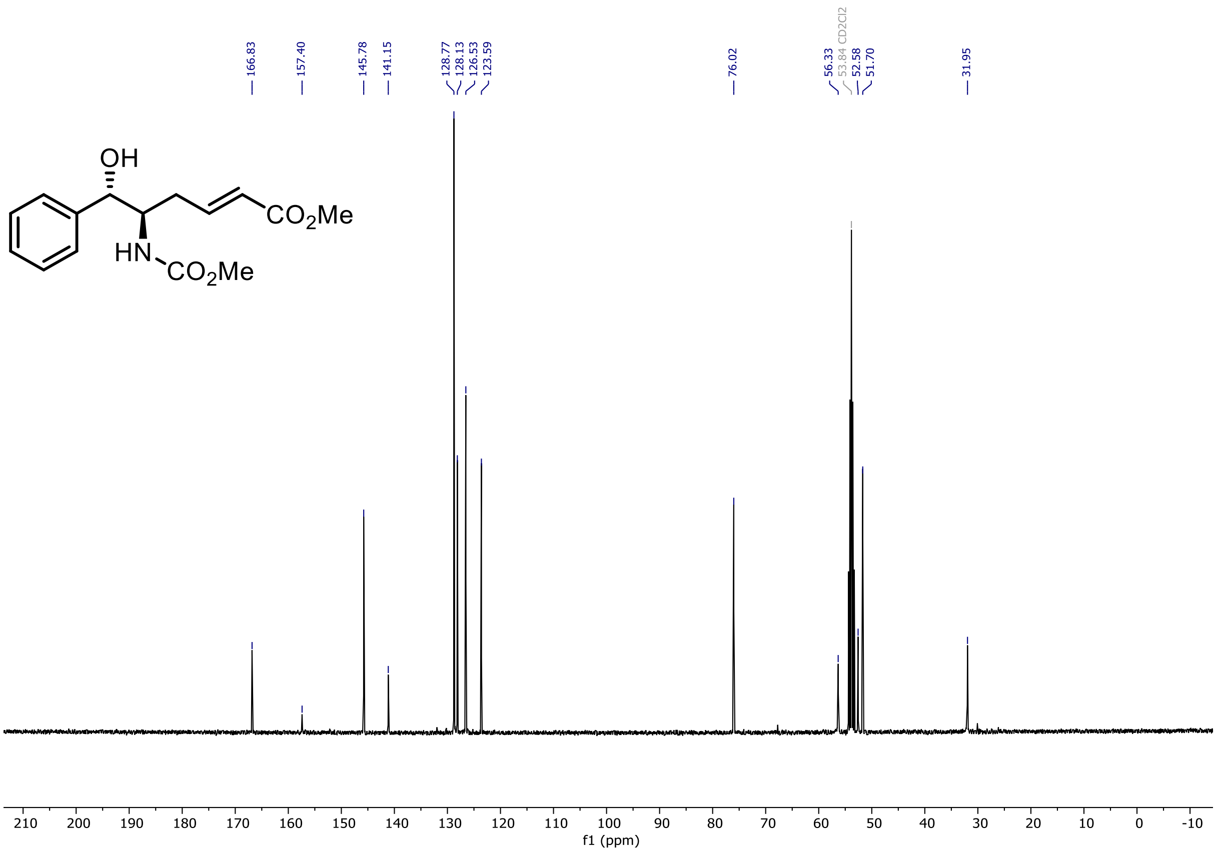

^{1}H NMR (400 MHz, CD_2Cl_2; top) and ^{13}C NMR (101 MHz, CD_2Cl_2; bottom) of compound **15c**

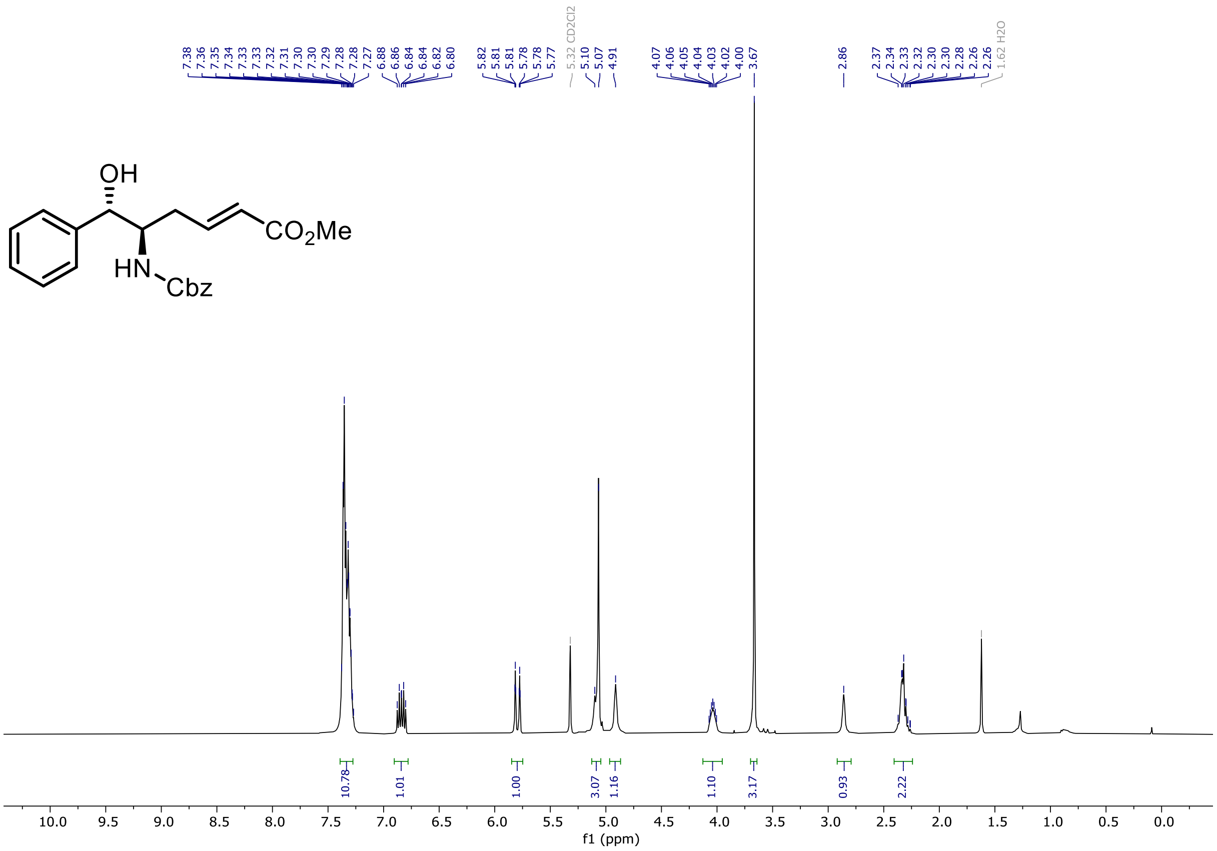

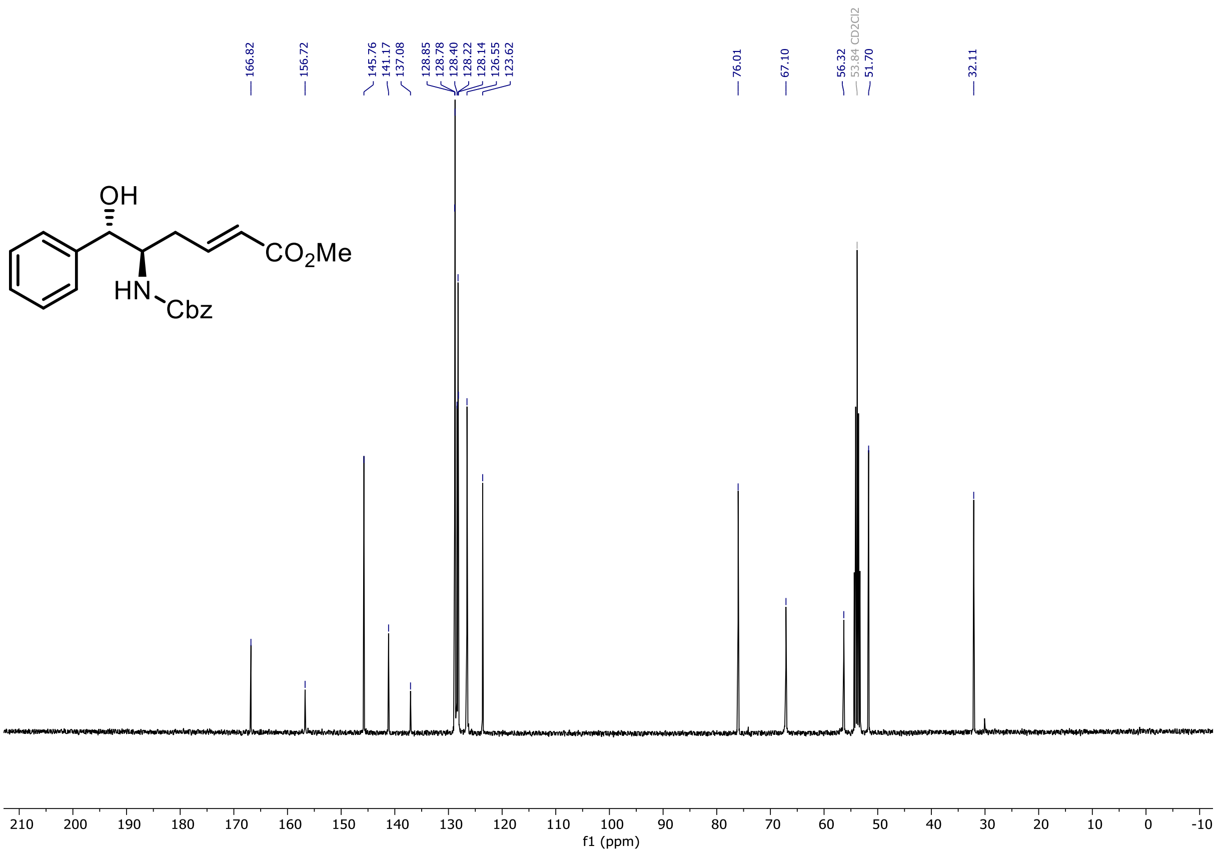

^{1}H NMR (600 MHz, CD_2Cl_2; top) and ^{13}C NMR (151 MHz, CD_2Cl_2; bottom) of compound **15d**

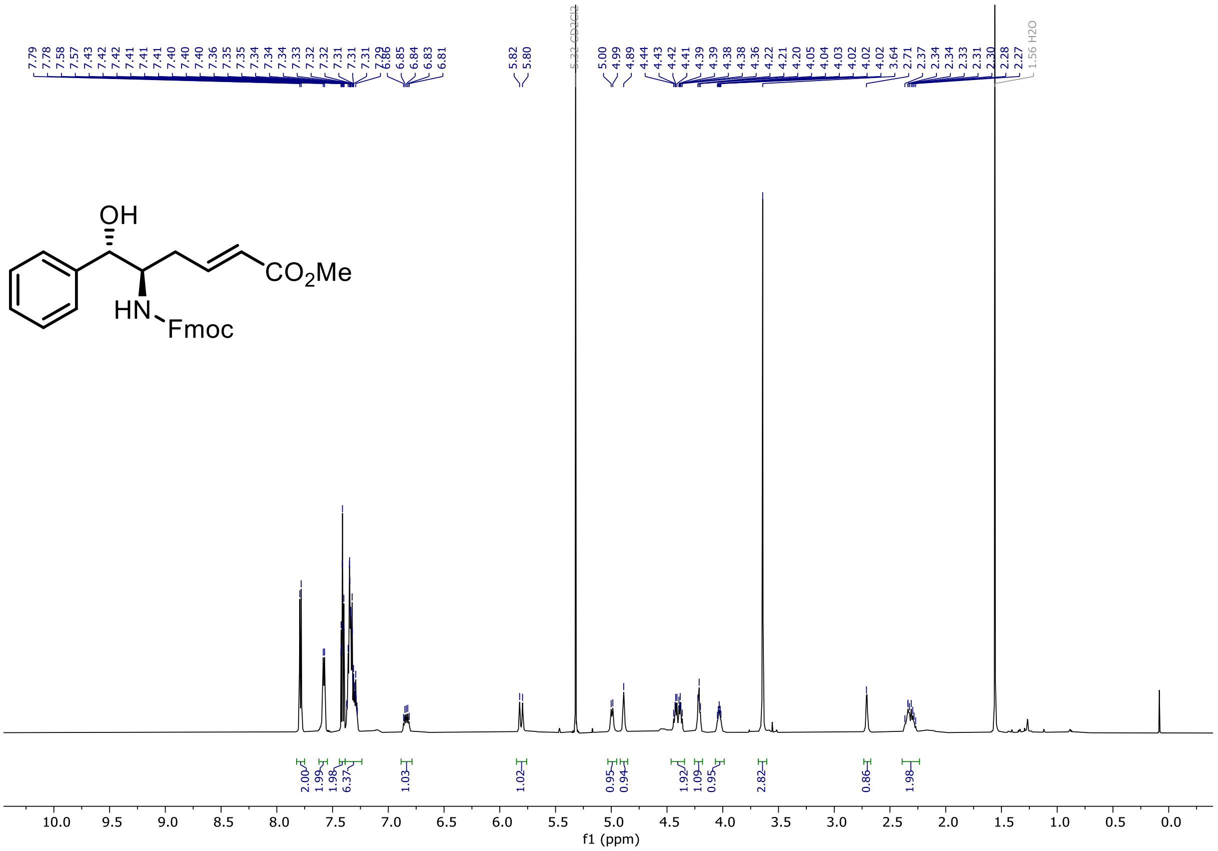

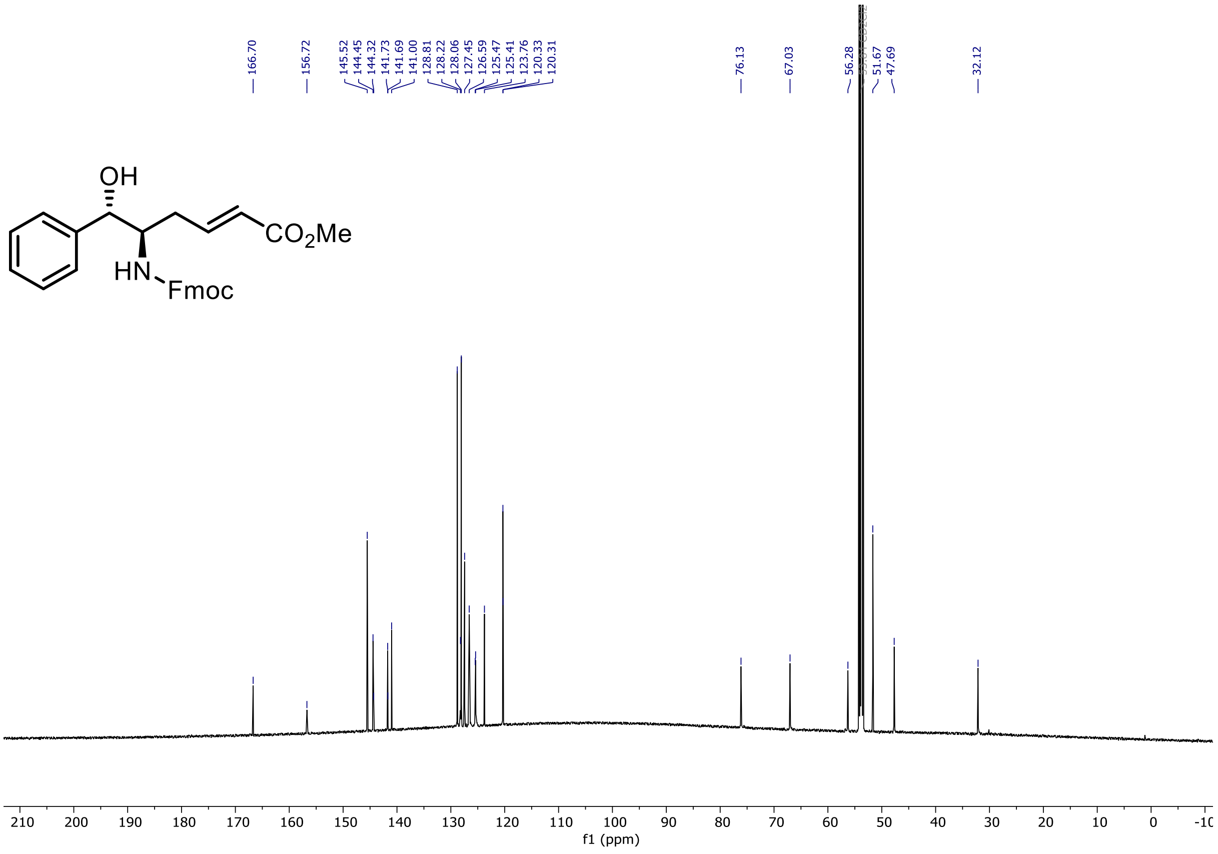

^{1}H NMR (400 MHz, CD_2Cl_2; top) and ^{13}C NMR (101 MHz, CD_2Cl_2; bottom) of compound **21**

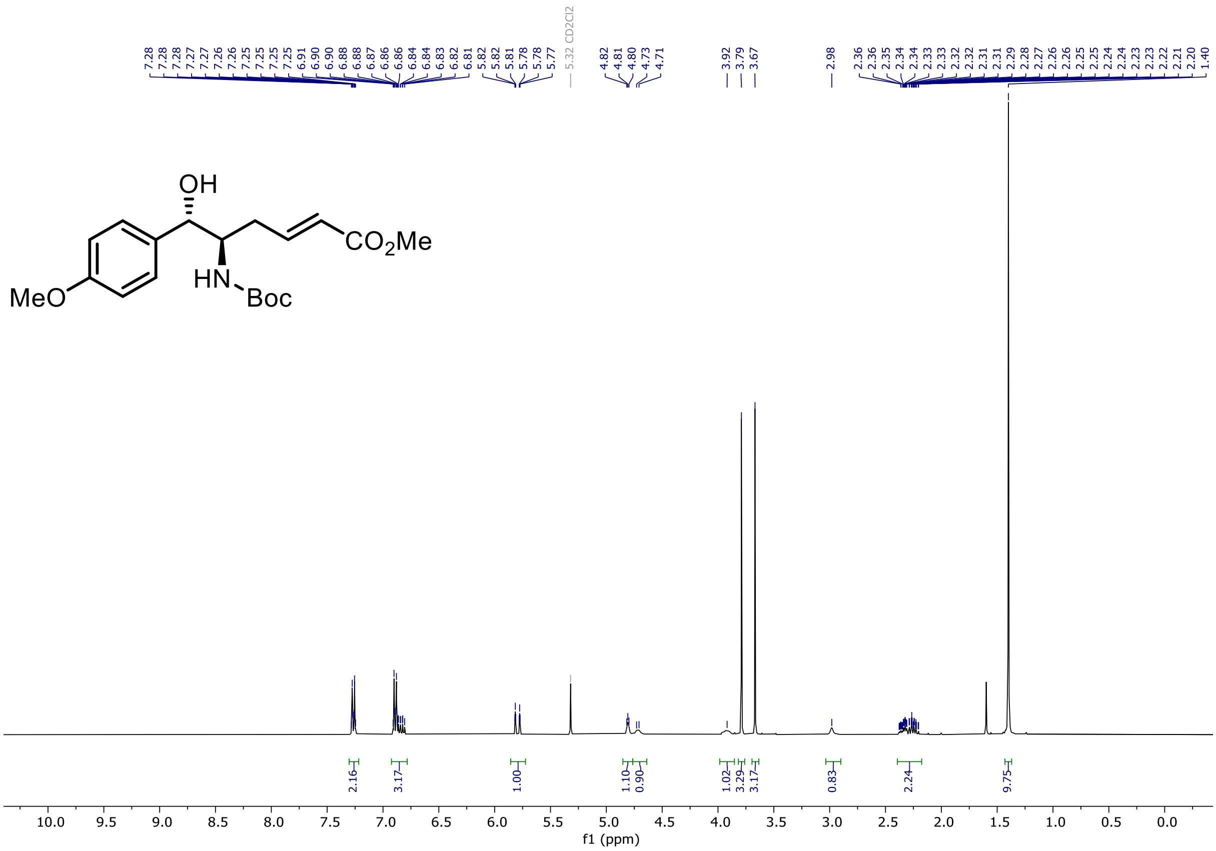

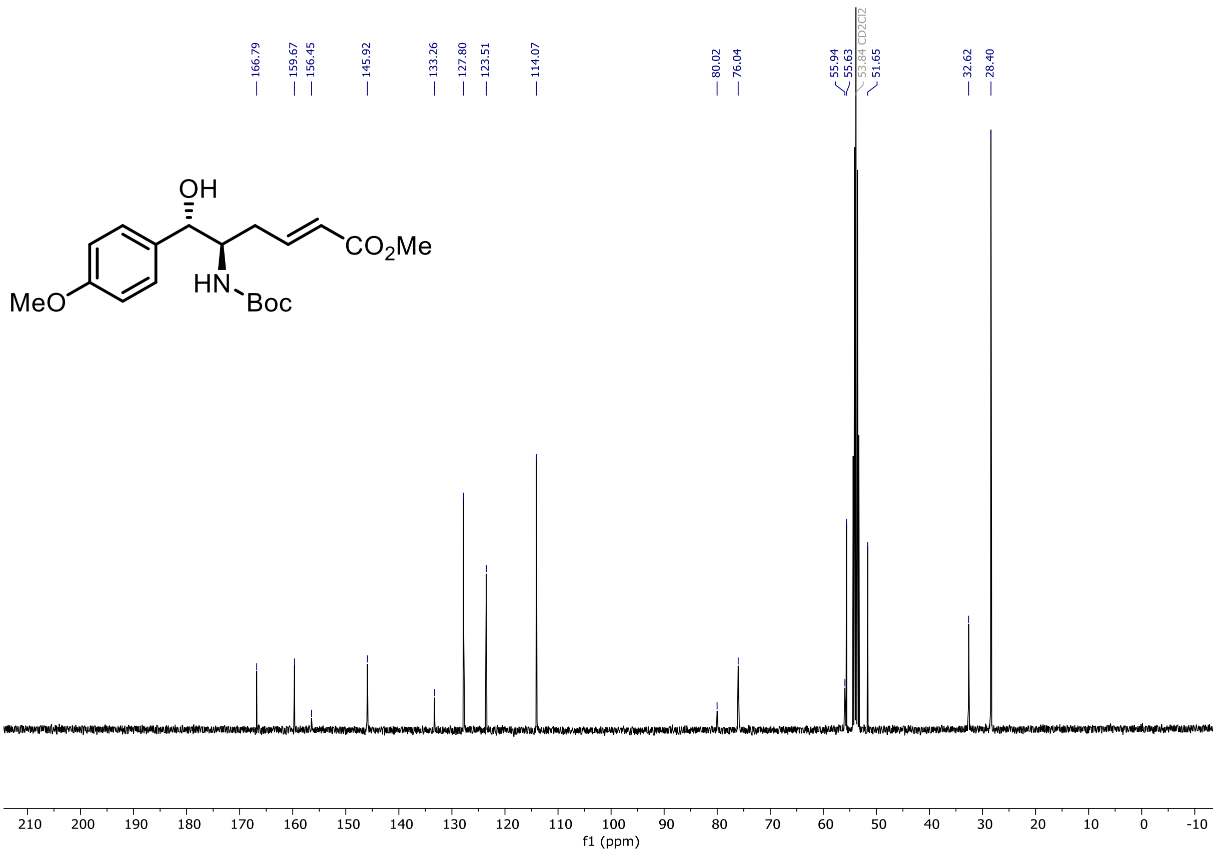

^{1}H NMR (400 MHz, CD_2Cl_2; top) and ^{13}C NMR (101 MHz, CD_2Cl_2; bottom) of compound **22**

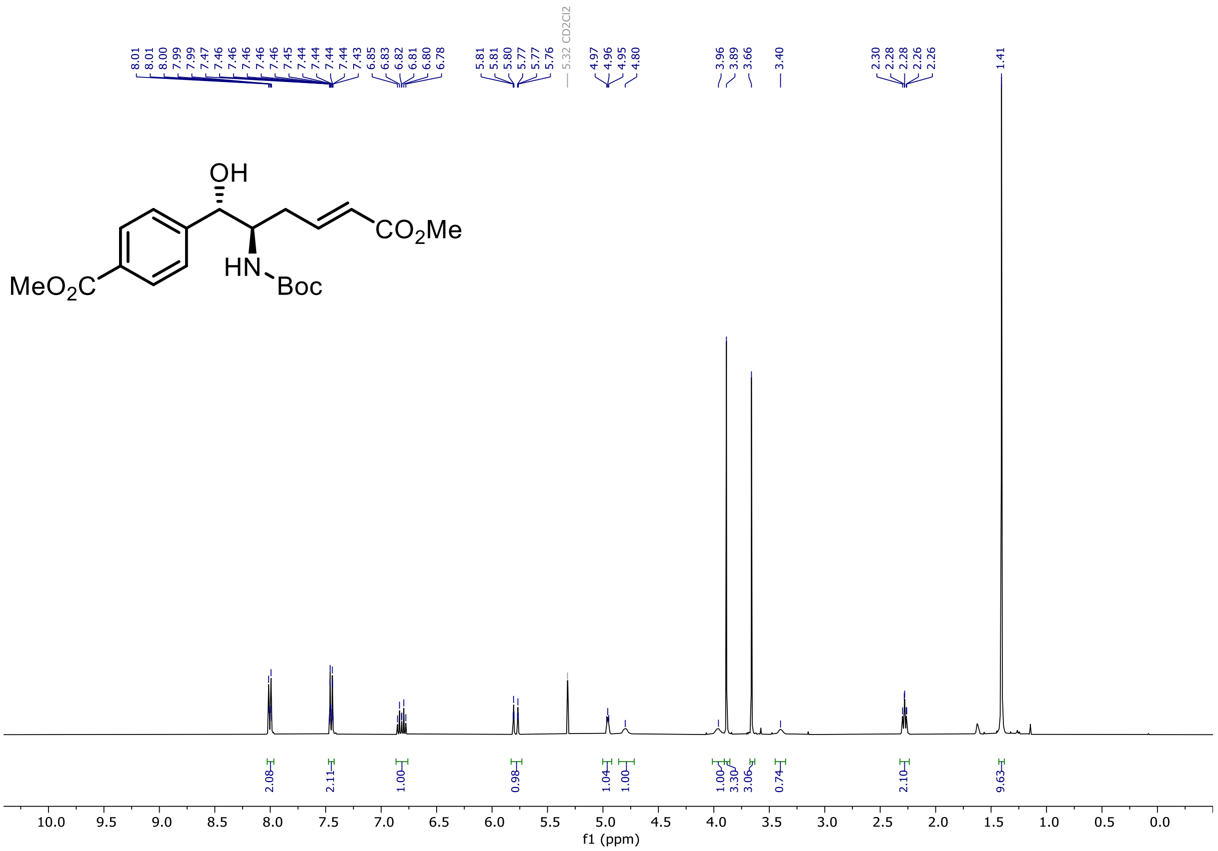

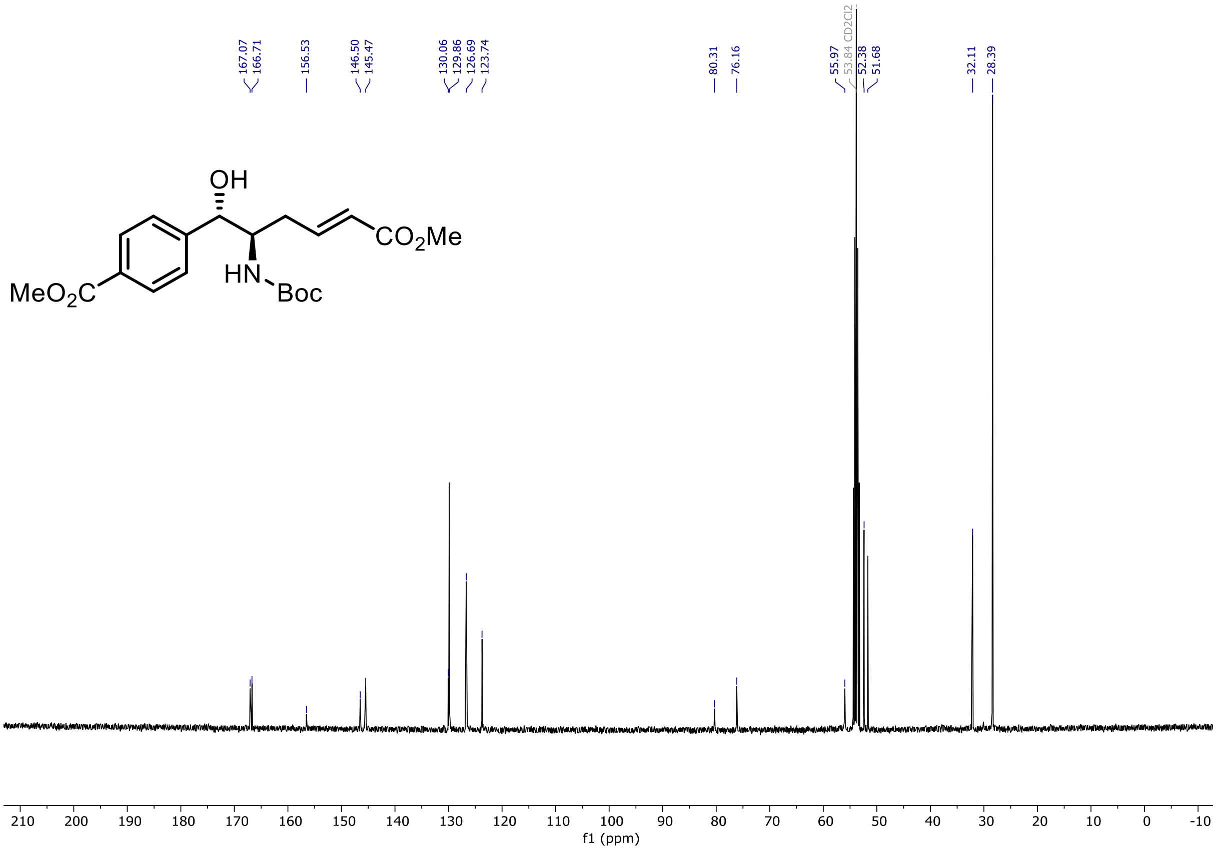

^{1}H NMR (400 MHz, CD_2Cl_2; top) and ^{13}C NMR (101 MHz, CD_2Cl_2; bottom) of compound **23**

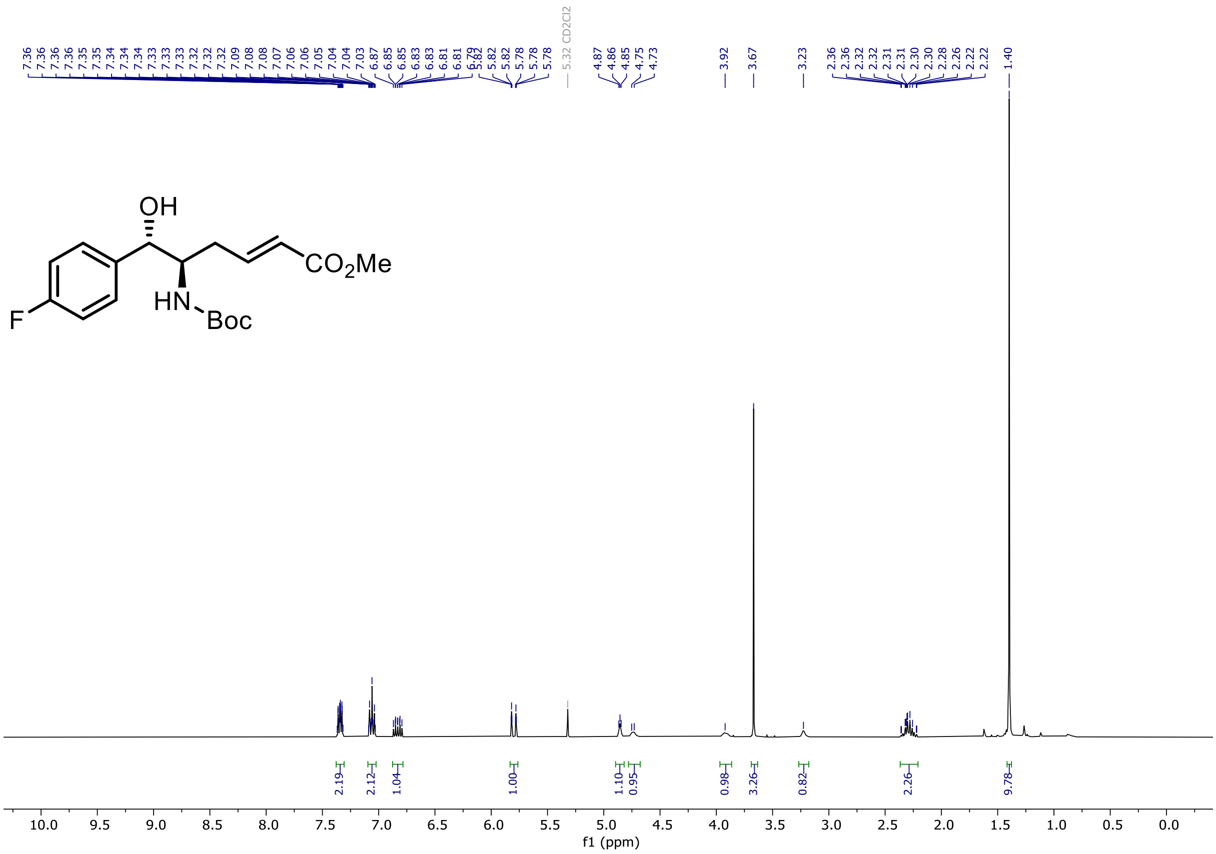

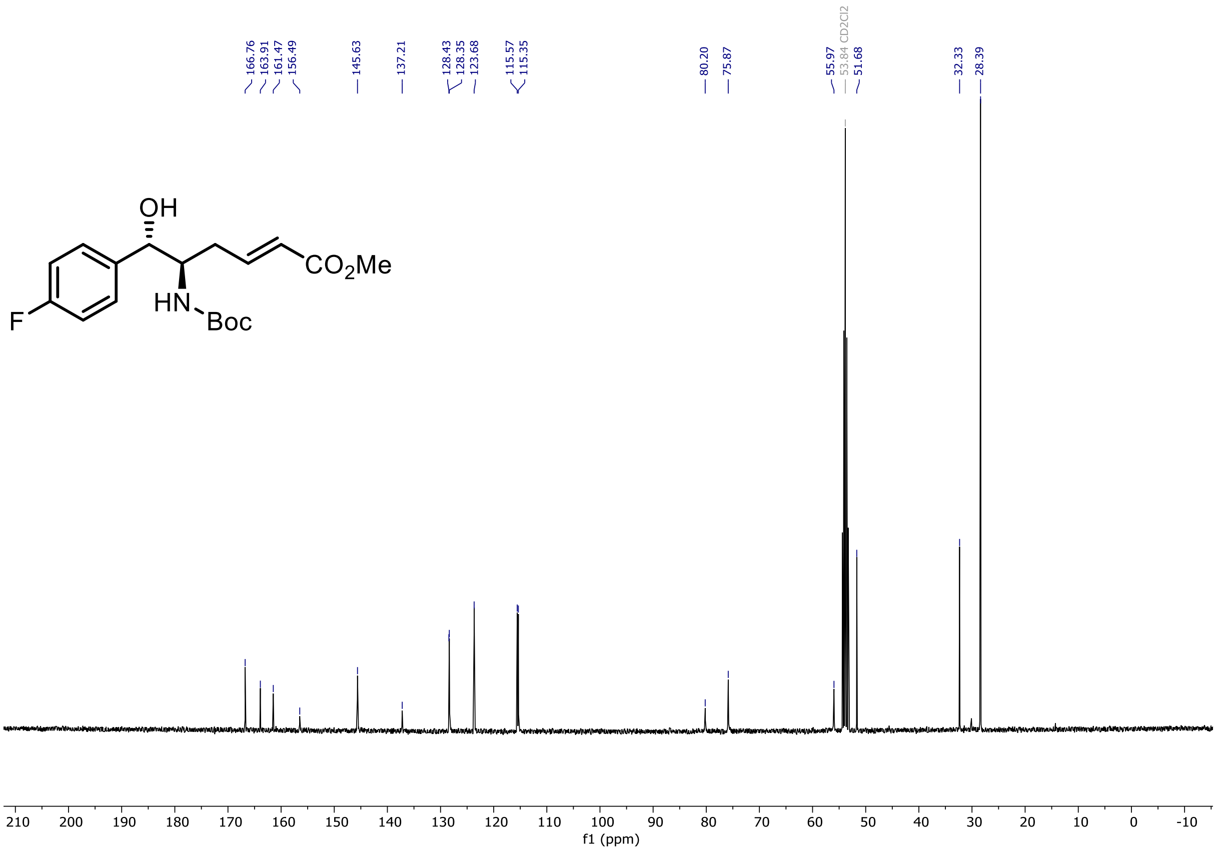

^{19}F NMR (282 MHz, CD_2Cl_2) of compound **23**

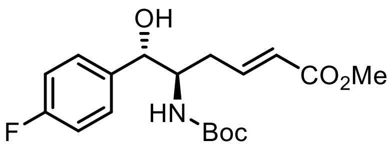

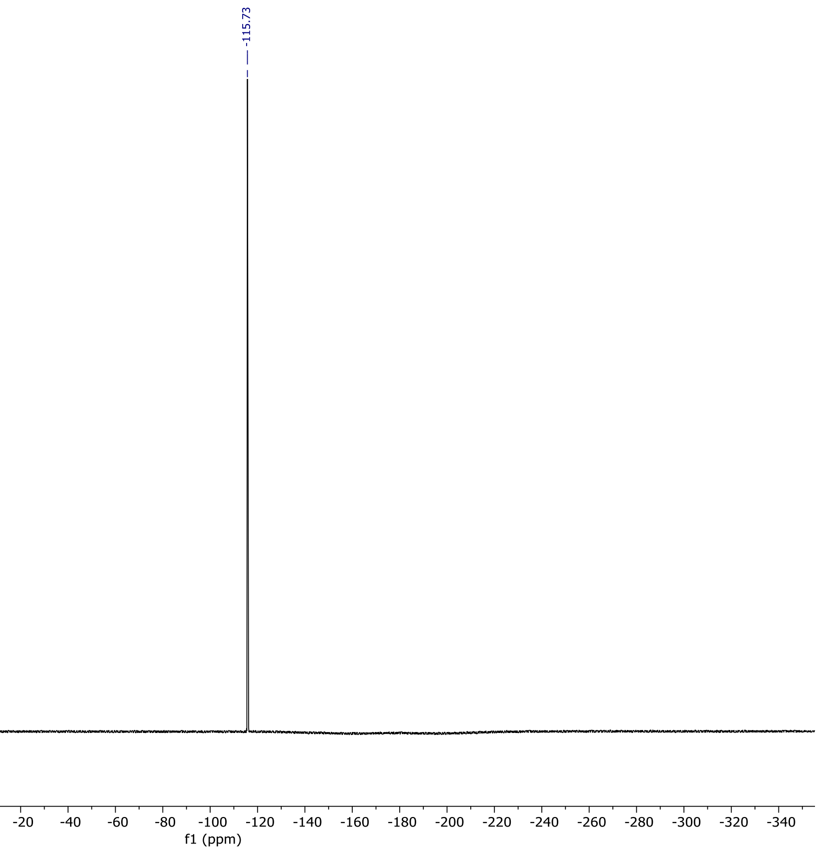

^{1}H NMR (400 MHz, CD_2Cl_2; top) and ^{13}C NMR (101 MHz, CD_2Cl_2; bottom) of compound **24**

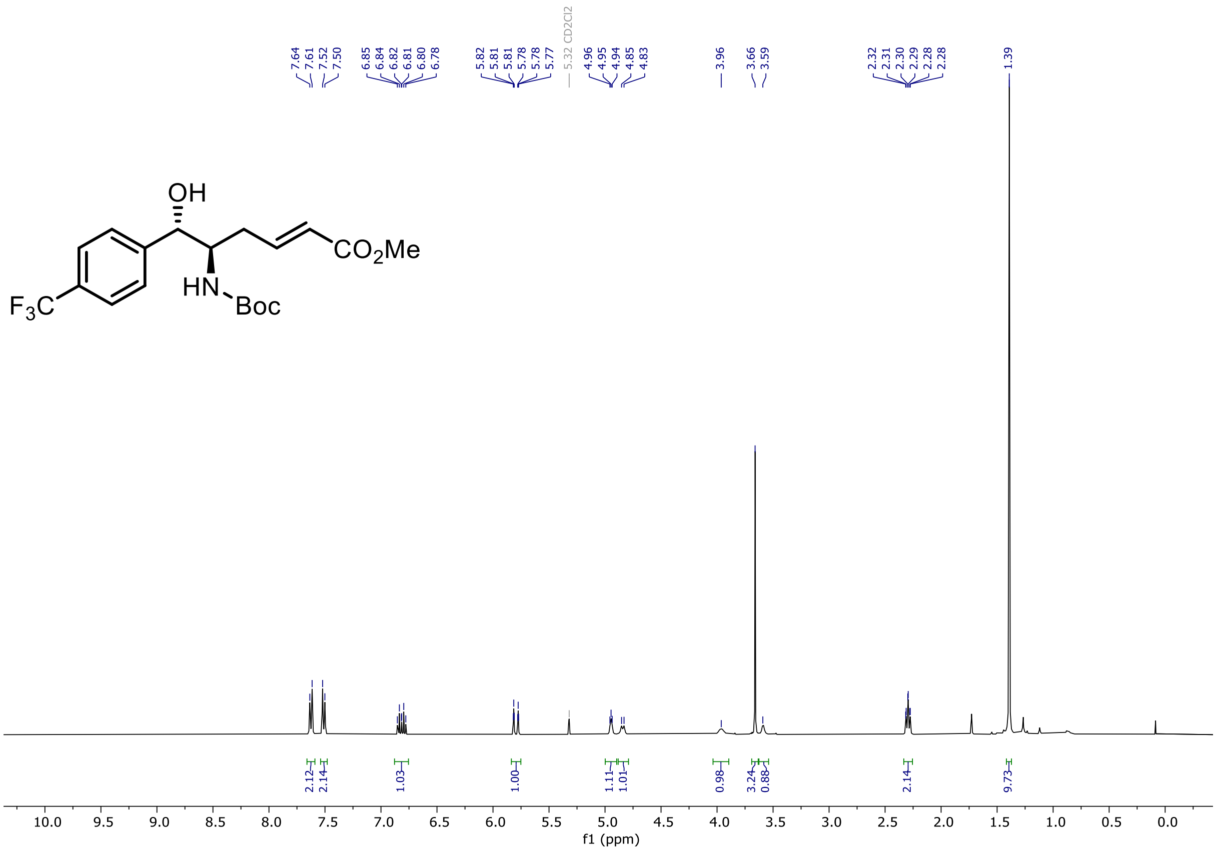

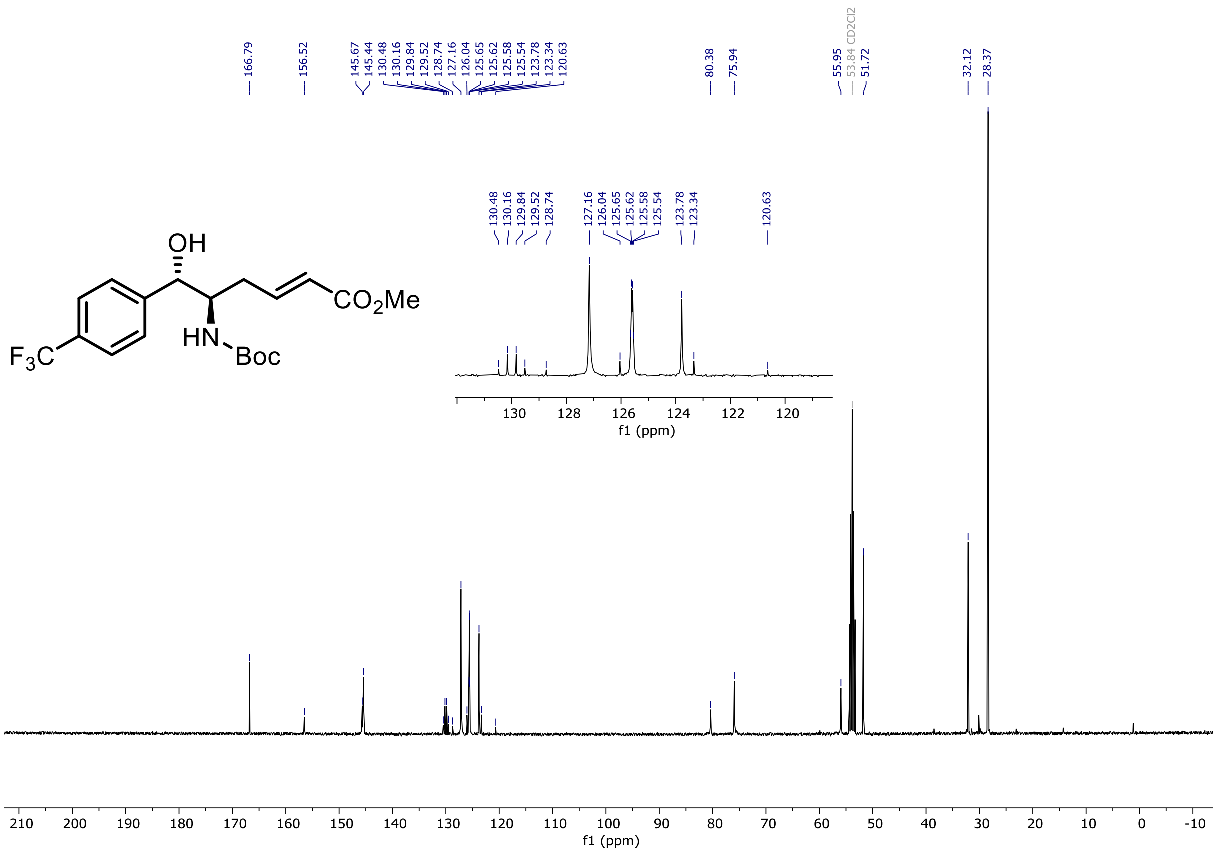

^{19}F NMR (282 MHz, CD_2Cl_2) of compound **24**

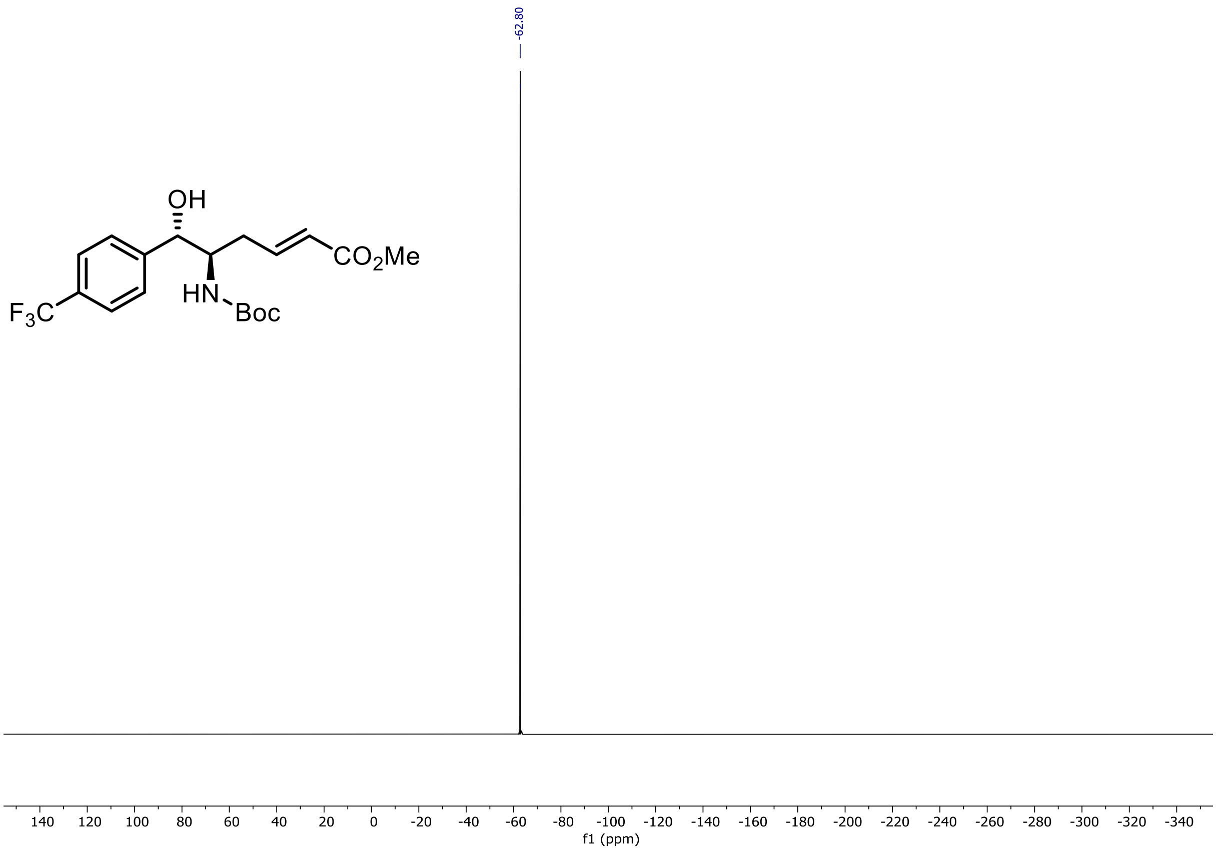

^{1}H NMR (400 MHz, CD_2Cl_2; top) and ^{13}C NMR (101 MHz, CD_2Cl_2; bottom) of compound **25**

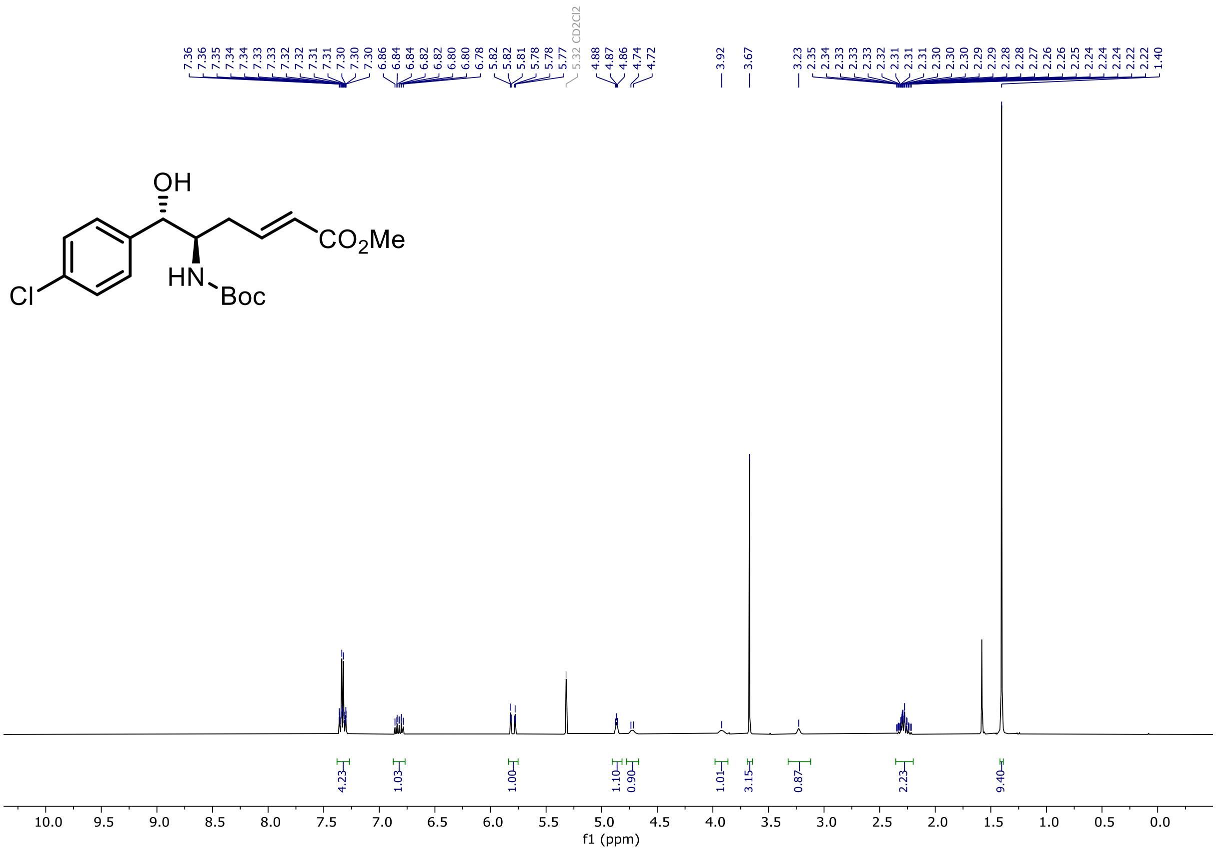

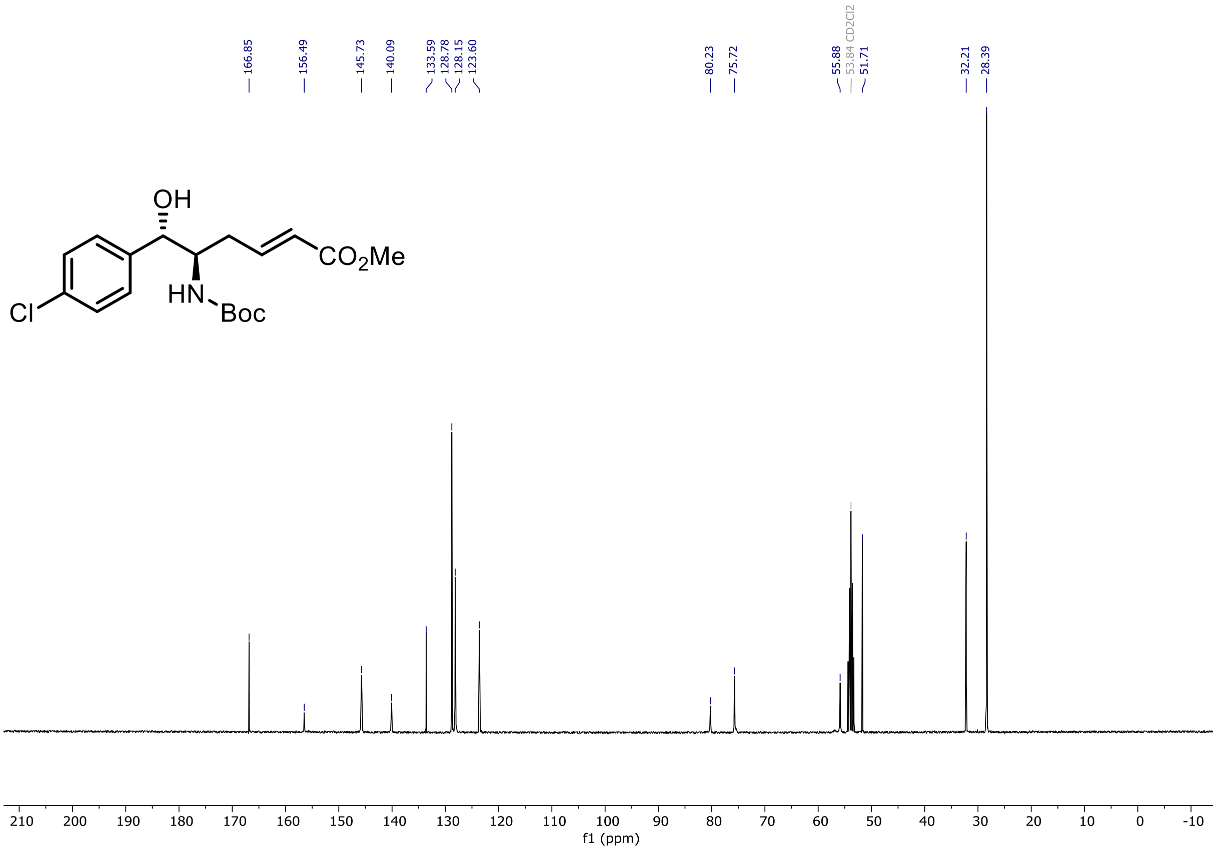

^{1}H NMR (400 MHz, CD_2Cl_2; top) and ^{13}C NMR (101 MHz, CD_2Cl_2; bottom) of compound **26**

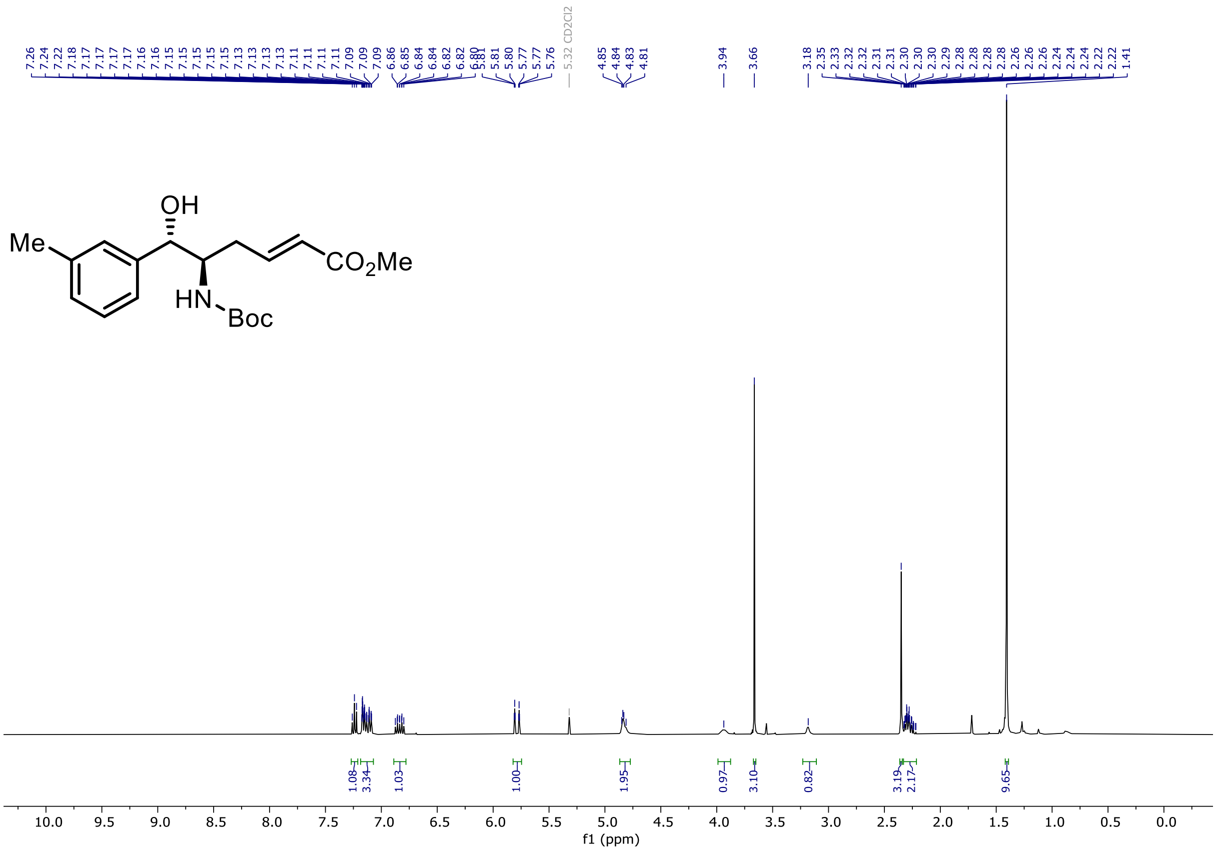

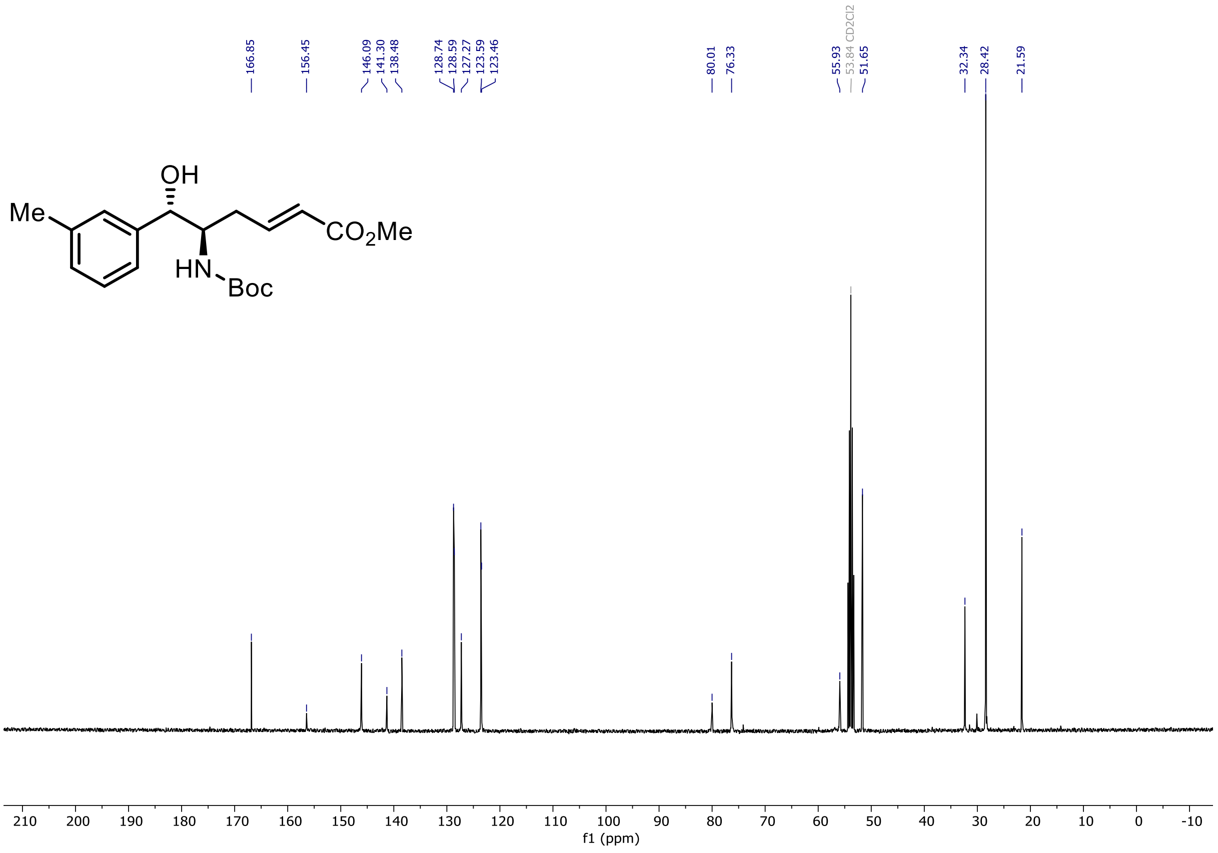

^1H NMR (400 MHz, CD_2Cl_2; top) and ^{13}C NMR (101 MHz, CD_2Cl_2; bottom) of compound **27**

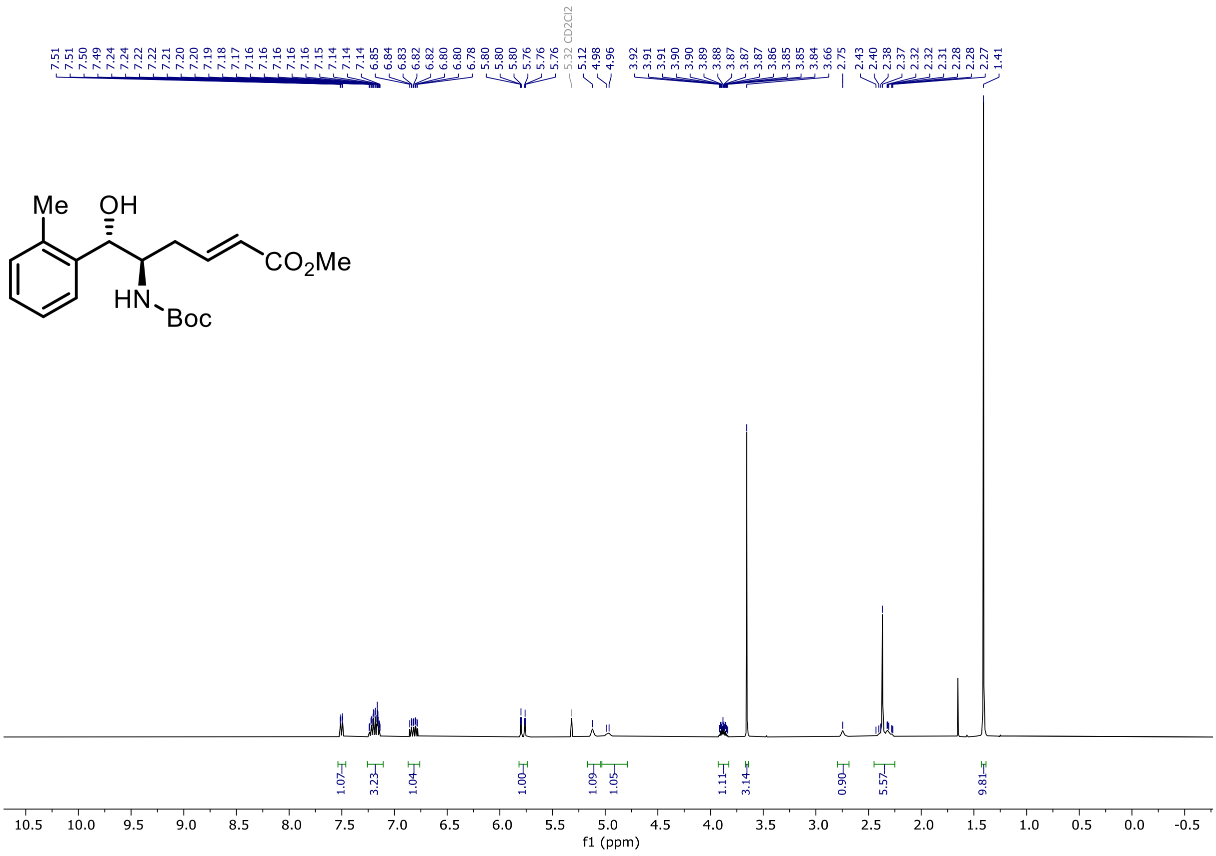

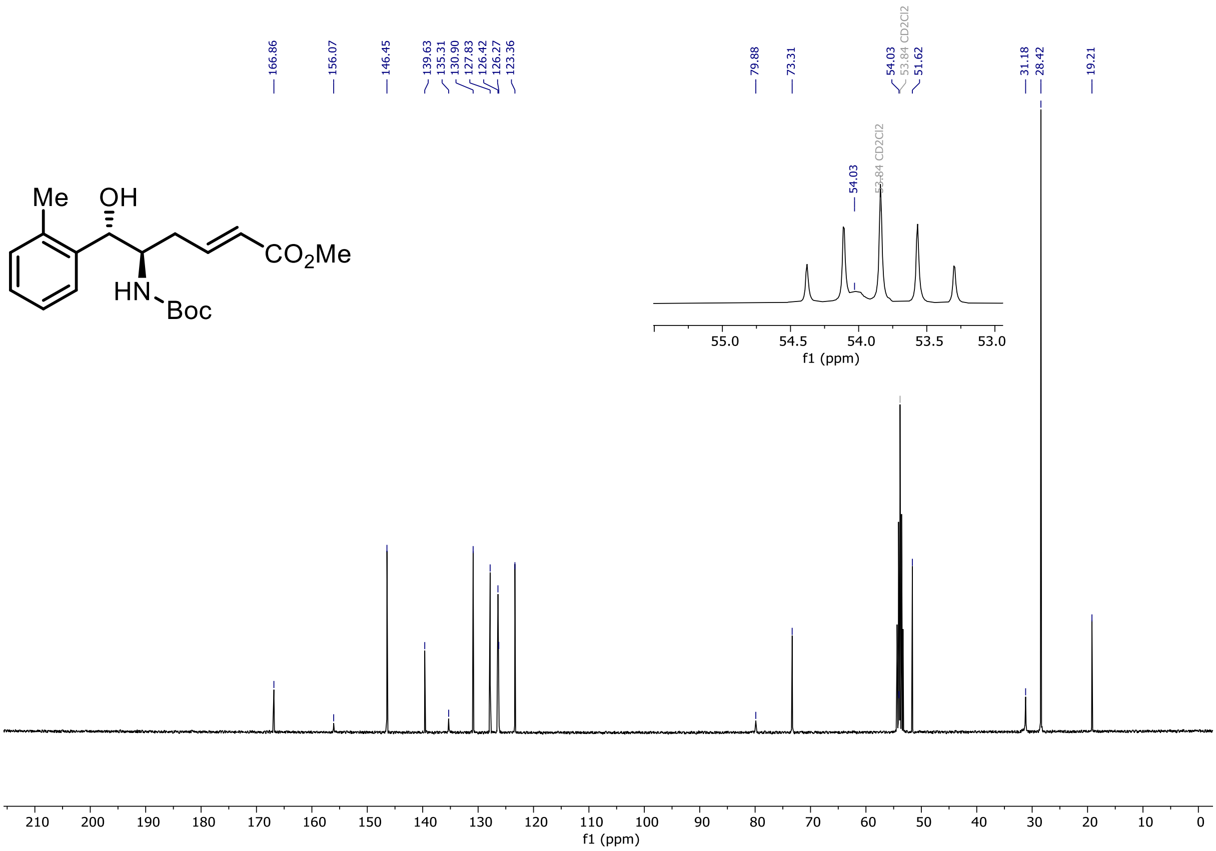

^{1}H NMR (400 MHz, CD_2Cl_2; top) and ^{13}C NMR (101 MHz, CD_2Cl_2; bottom) of compound **28**

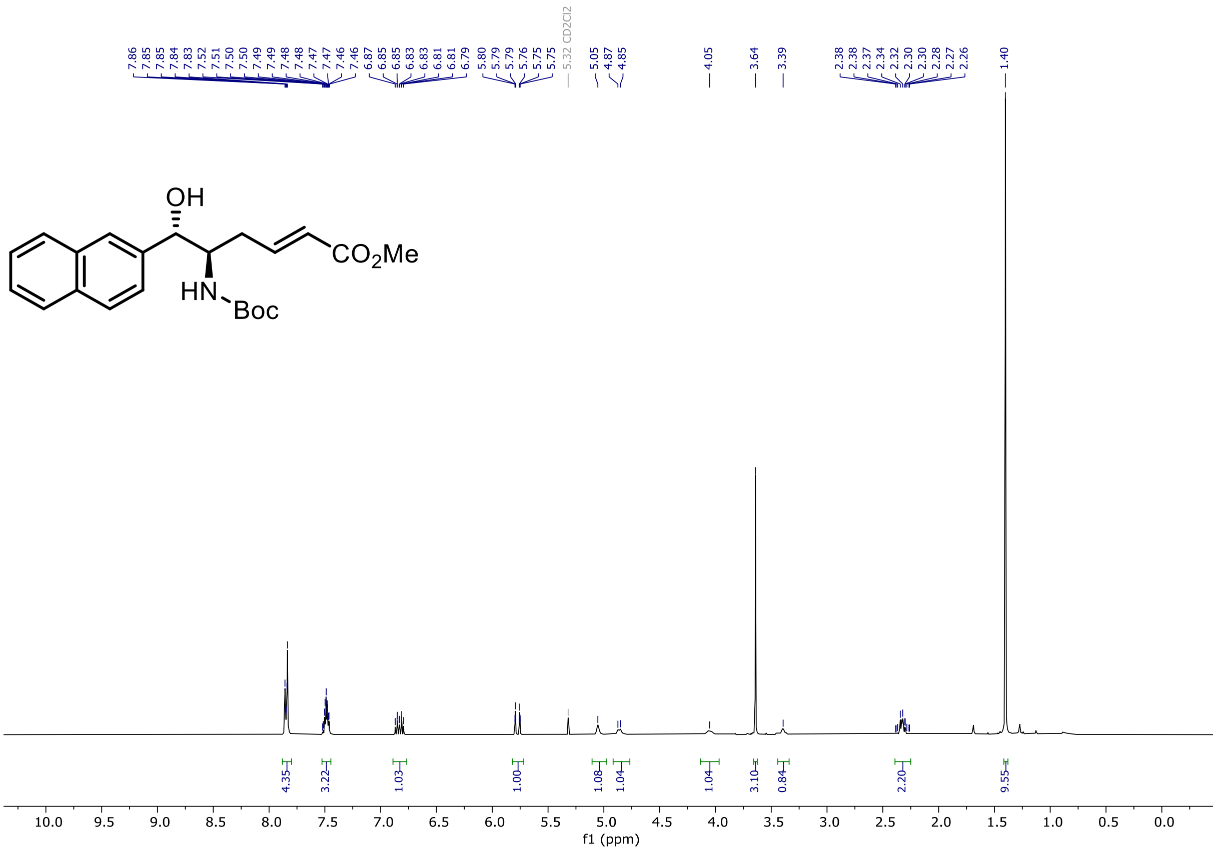

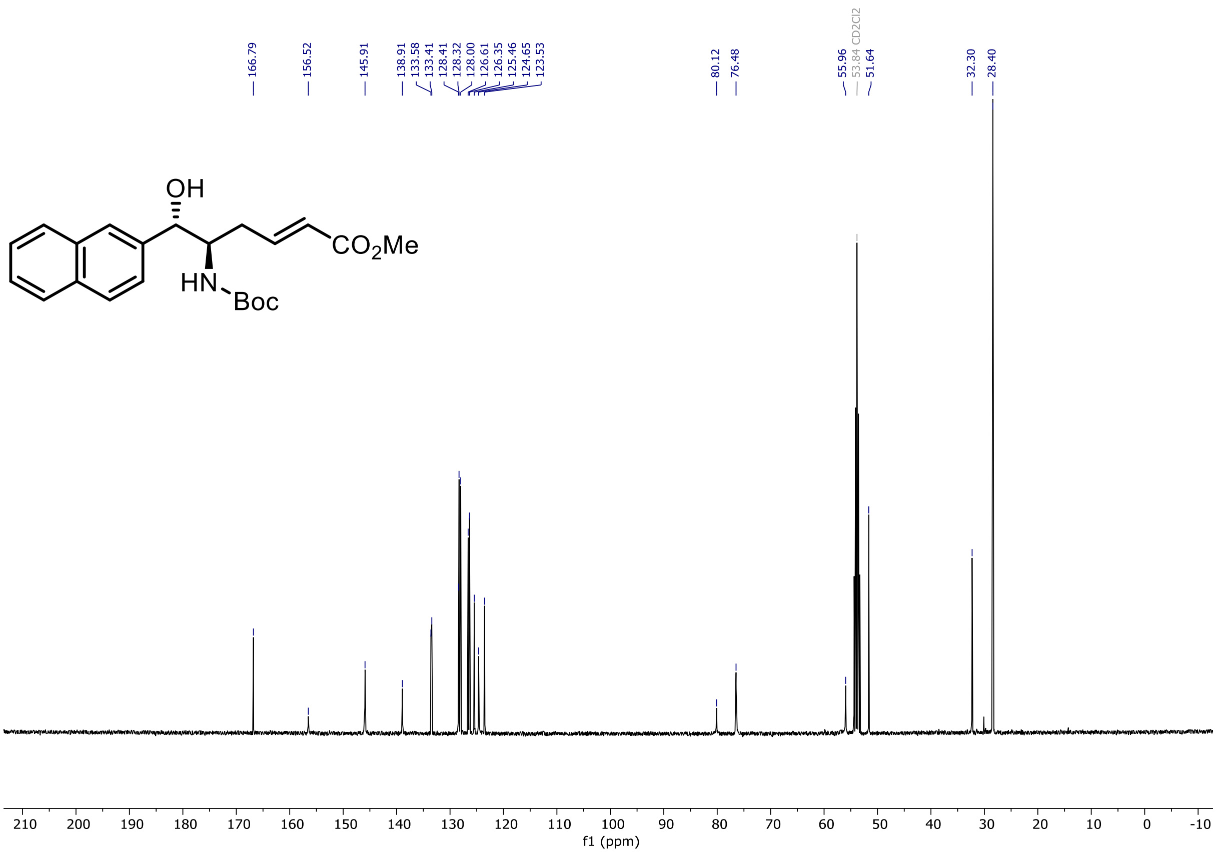

^{1}H NMR (400 MHz, CD_2Cl_2; top) and ^{13}C NMR (101 MHz, CD_2Cl_2; bottom) of compound **29**

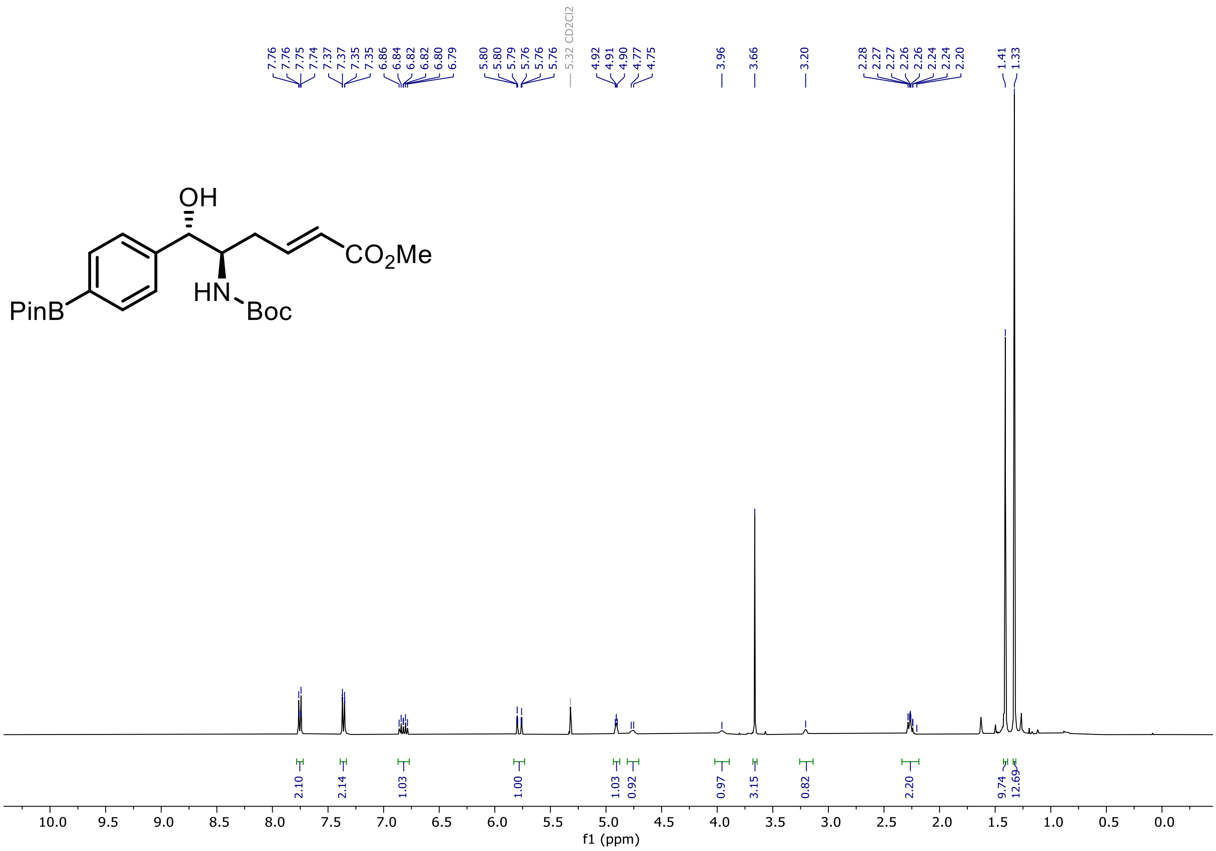

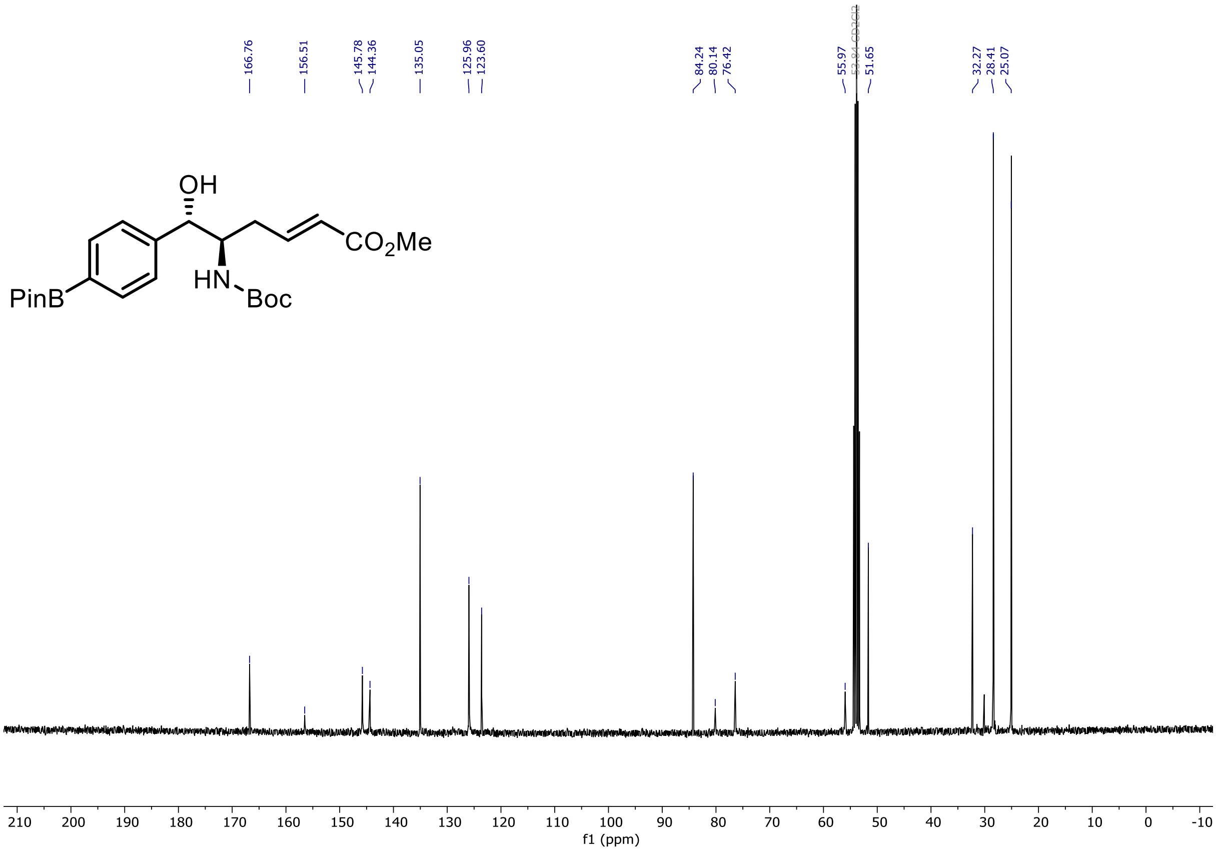

^{1}H NMR (400 MHz, CD_2Cl_2; top) and ^{13}C NMR (151 MHz, CD_2Cl_2; bottom) of compound **30**

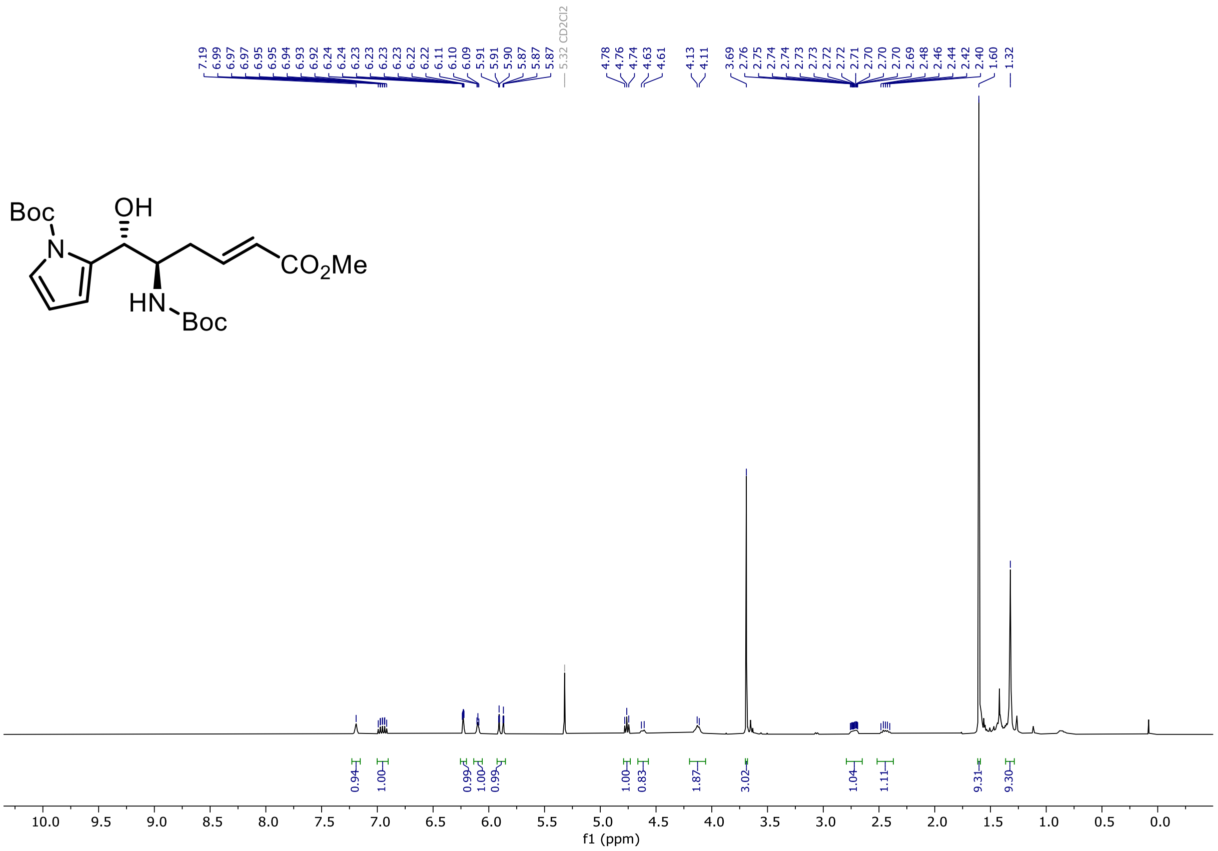

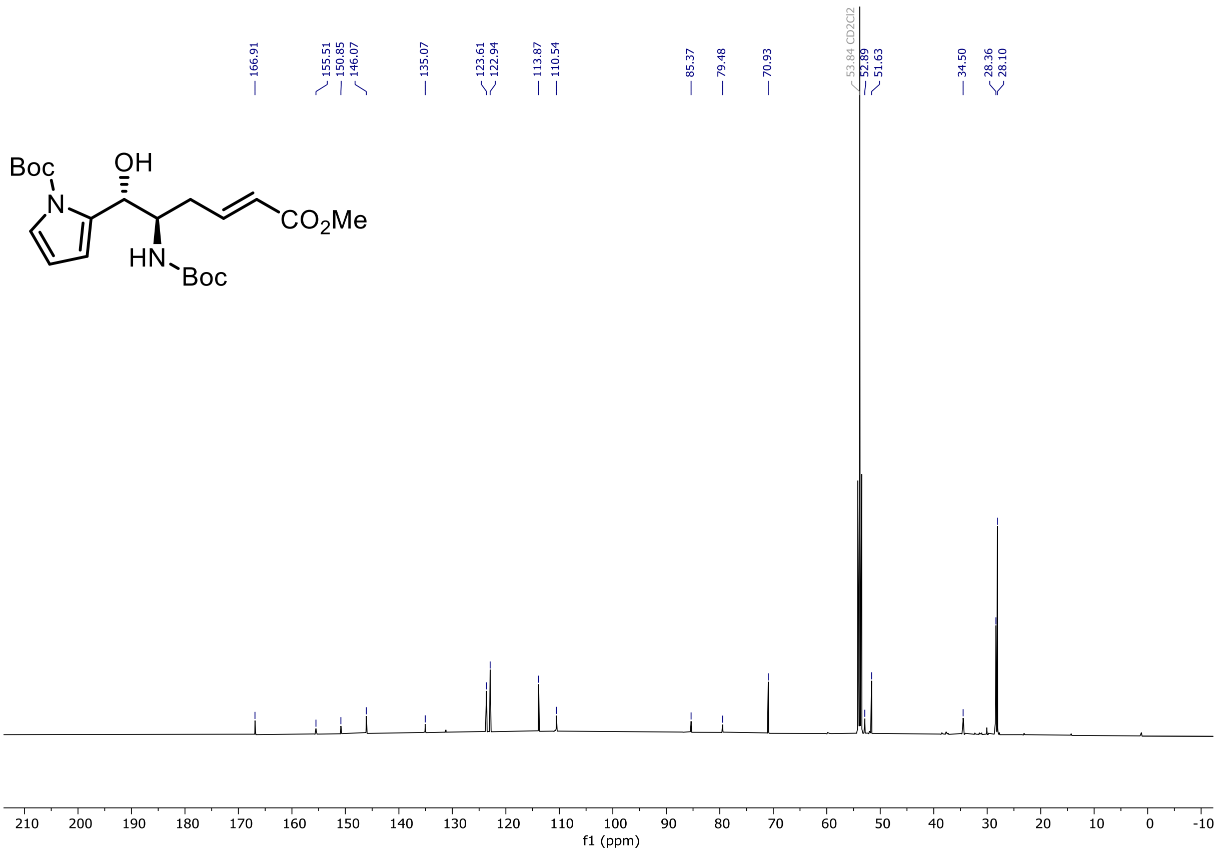

^{1}H NMR (400 MHz, $CDCl_3$; top) and ^{13}C NMR (101 MHz, $CDCl_3$; bottom) of compound **31**

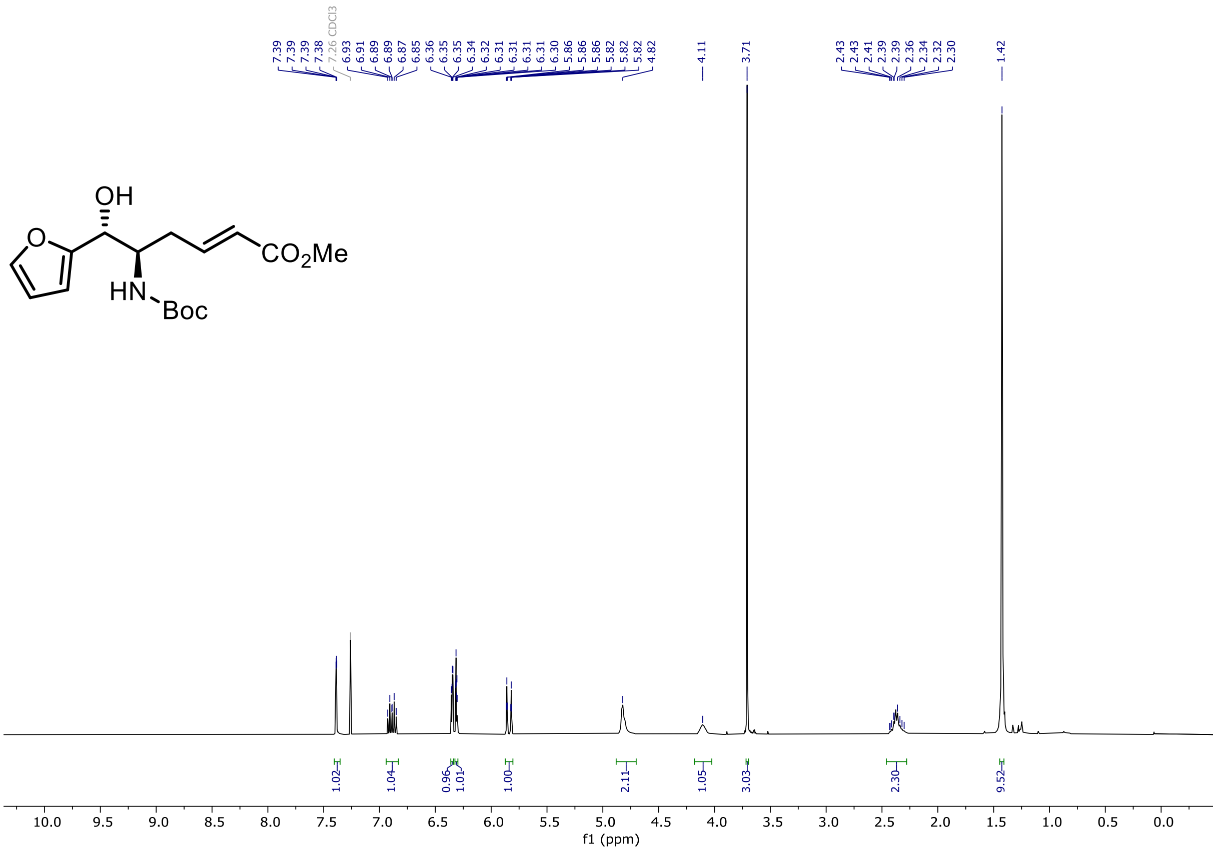

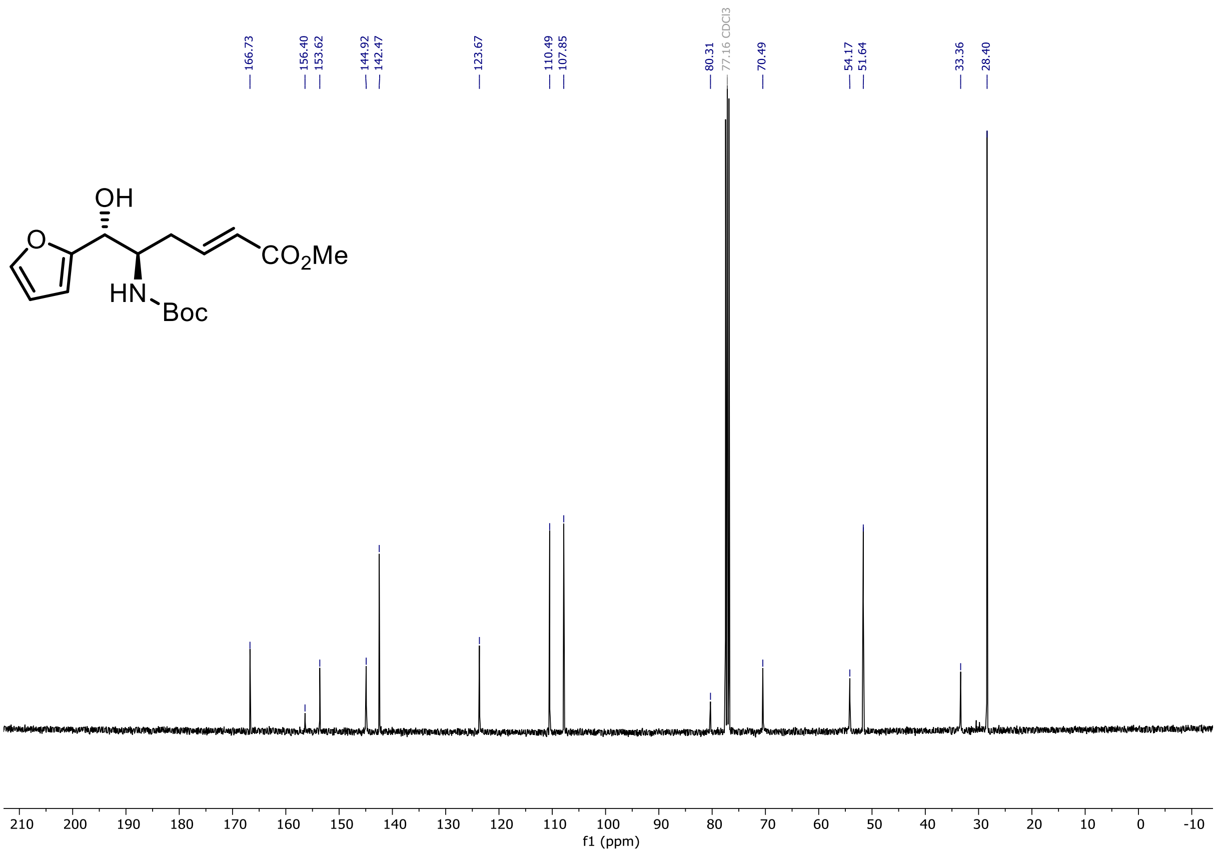

^{1}H NMR (400 MHz, CD_2Cl_2; top) and ^{13}C NMR (101 MHz, CD_2Cl_2; bottom) of compound **32**

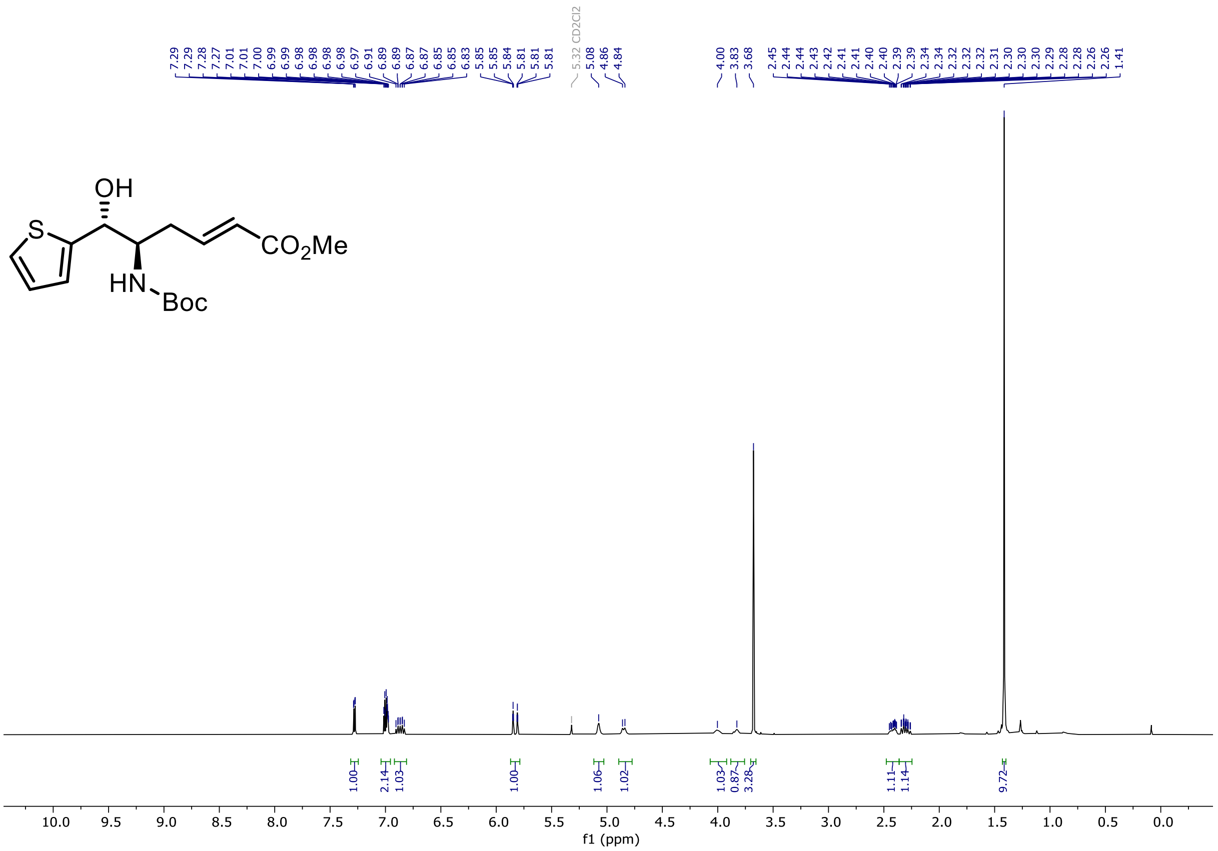

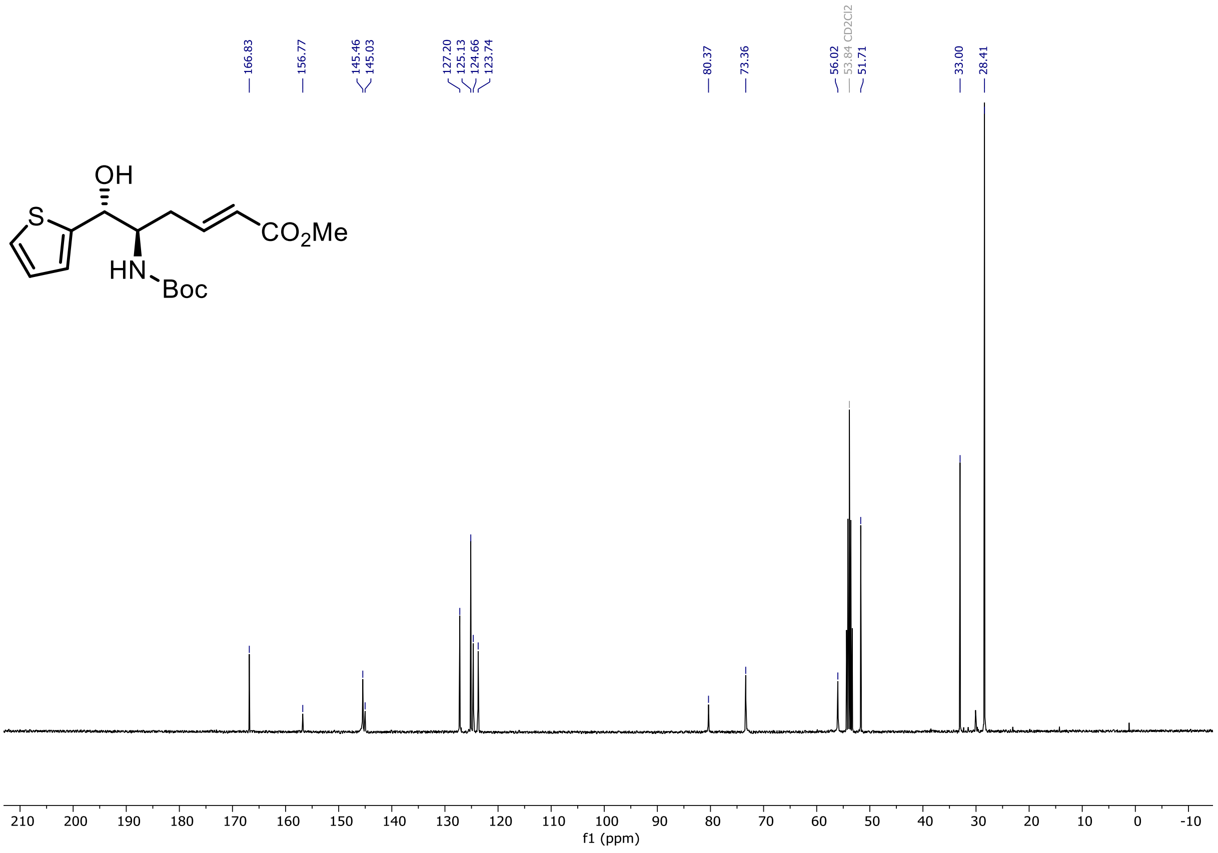

^{1}H NMR (400 MHz, CD_2Cl_2; top) and ^{13}C NMR (101 MHz, CD_2Cl_2; bottom) of compound **33**

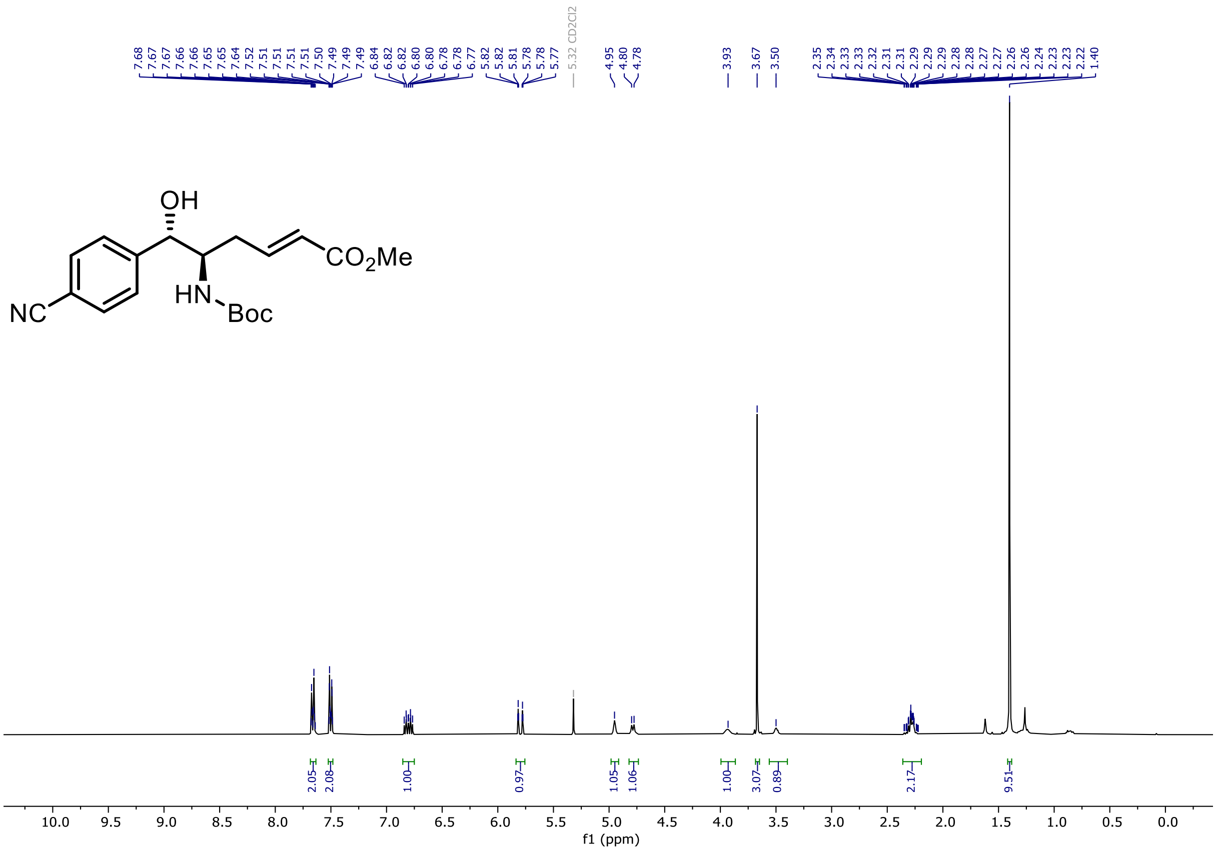

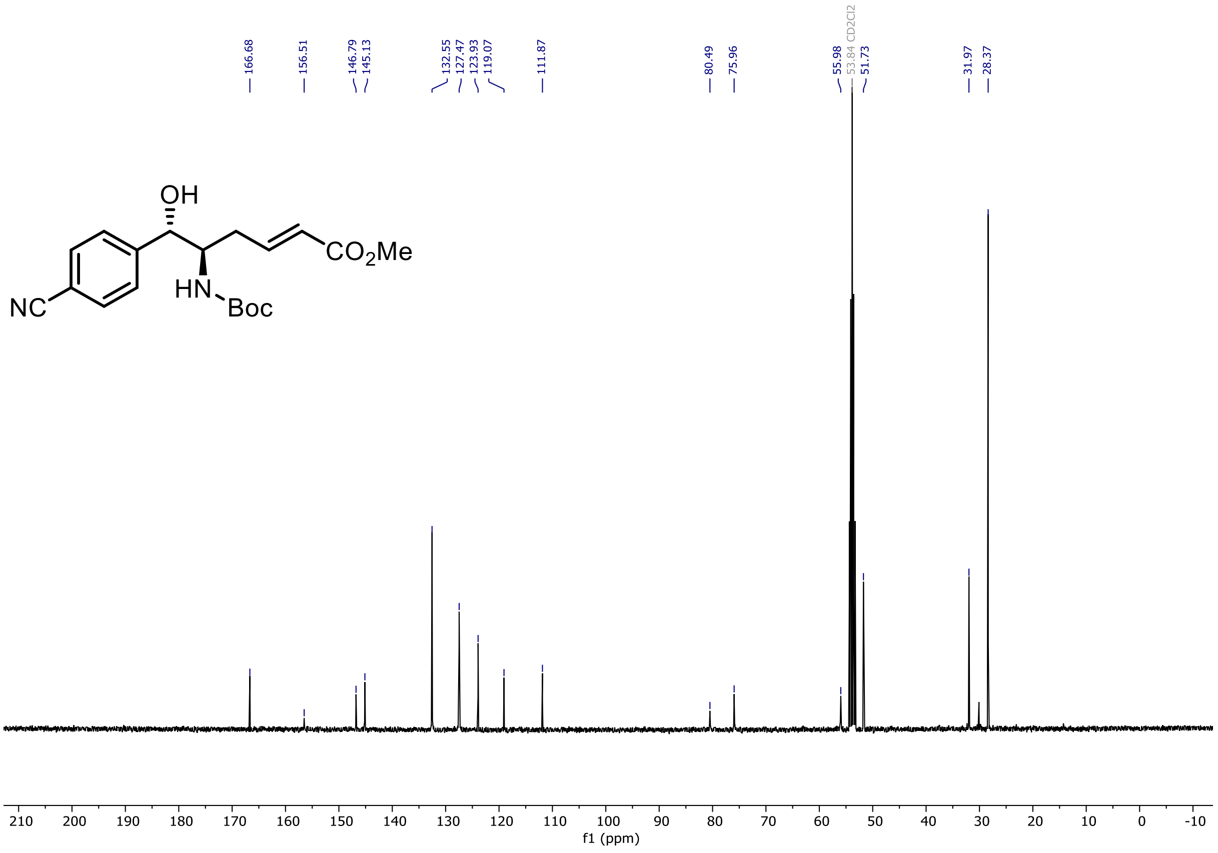

^{1}H NMR (400 MHz, CD_2Cl_2; top) and ^{13}C NMR (101 MHz, CD_2Cl_2; bottom) of compound **34**

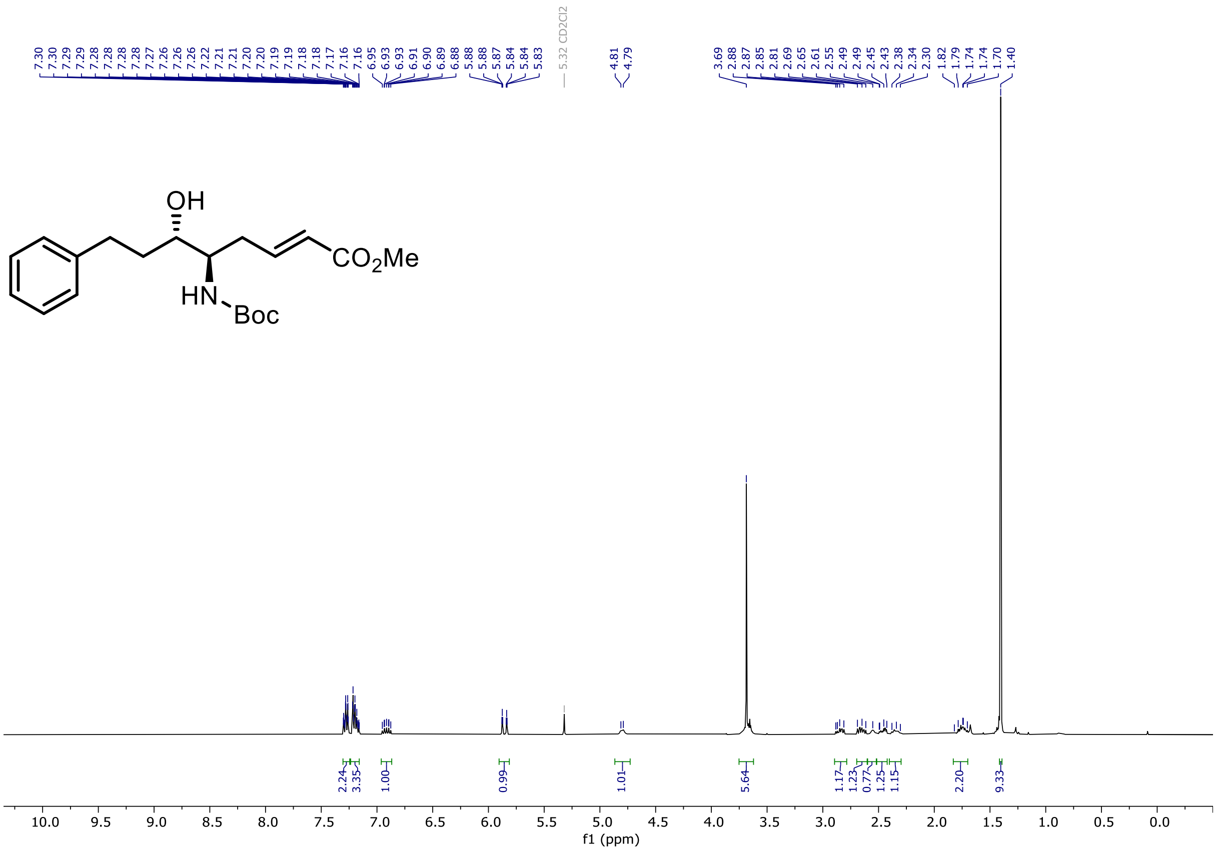

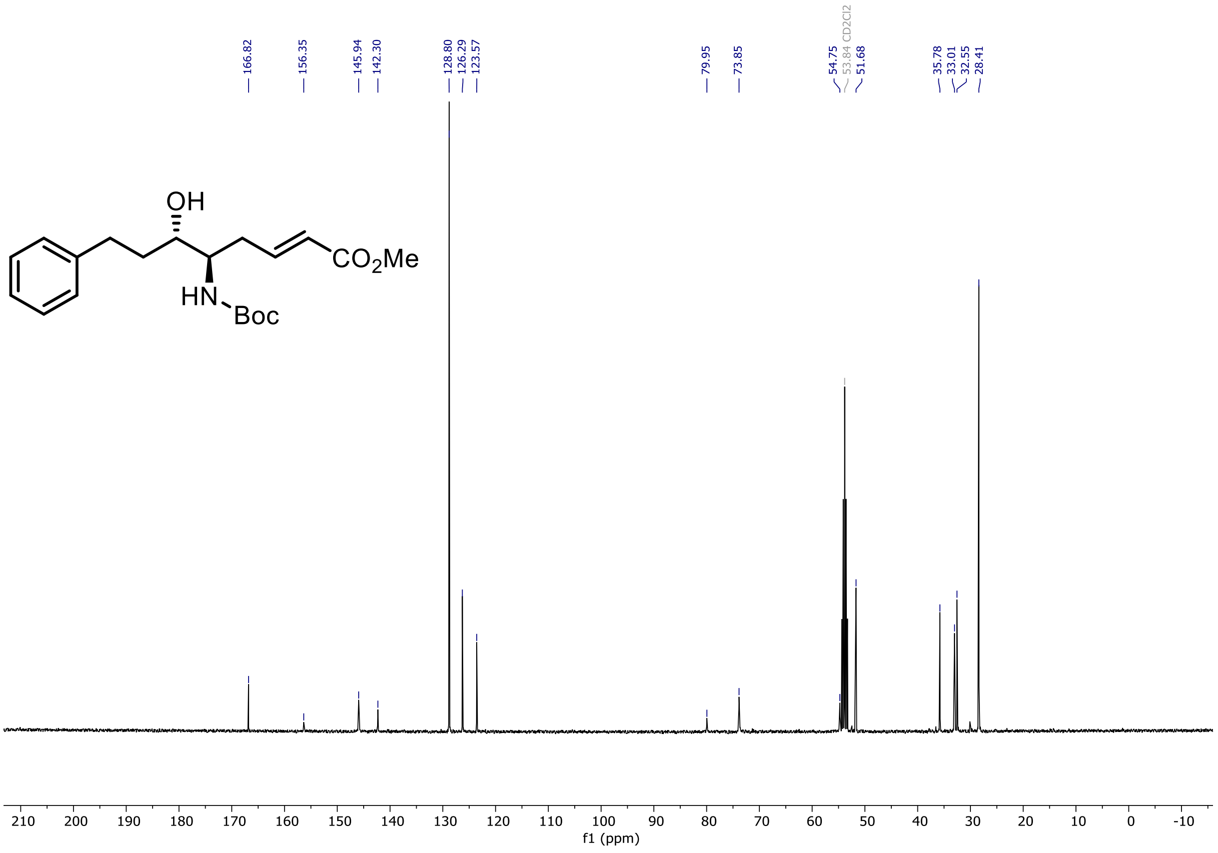

^{1}H NMR (400 MHz, CD_2Cl_2; top) and ^{13}C NMR (101 MHz, CD_2Cl_2; bottom) of compound **36**

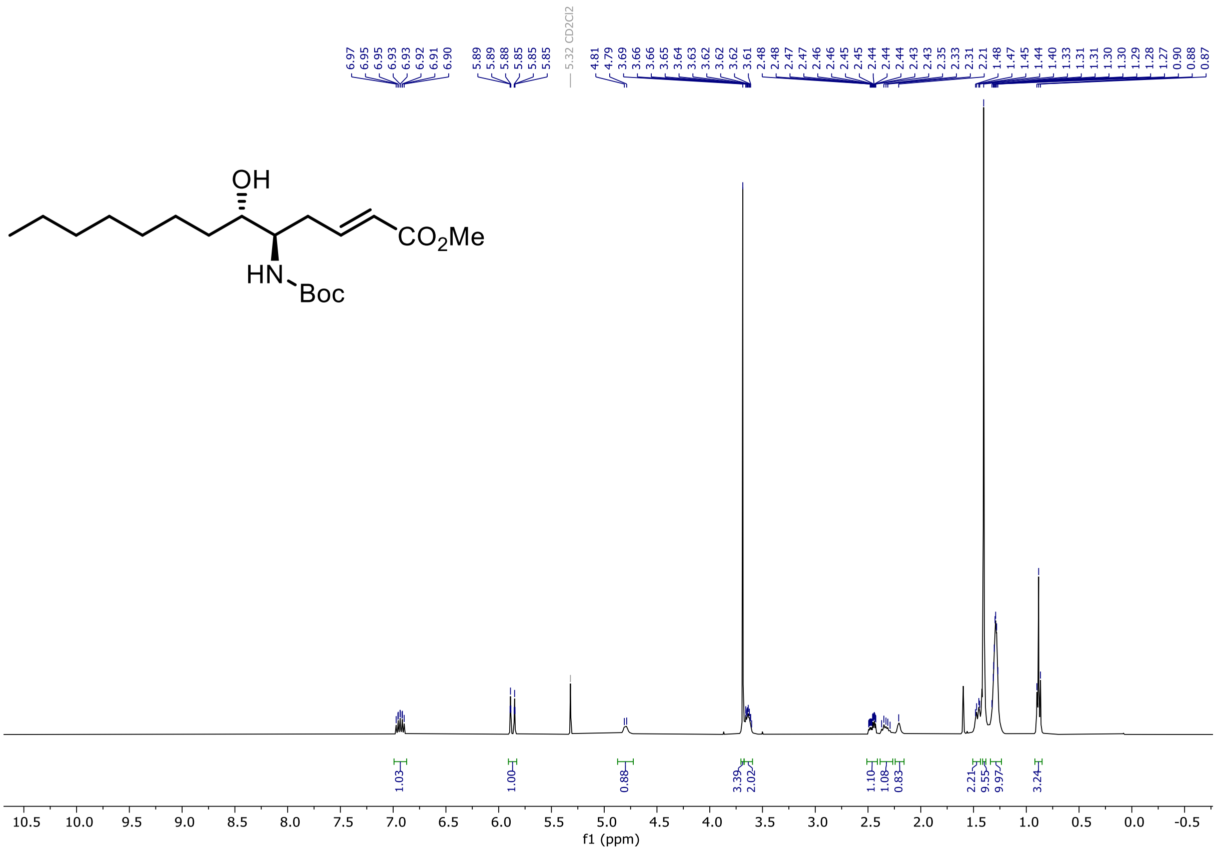

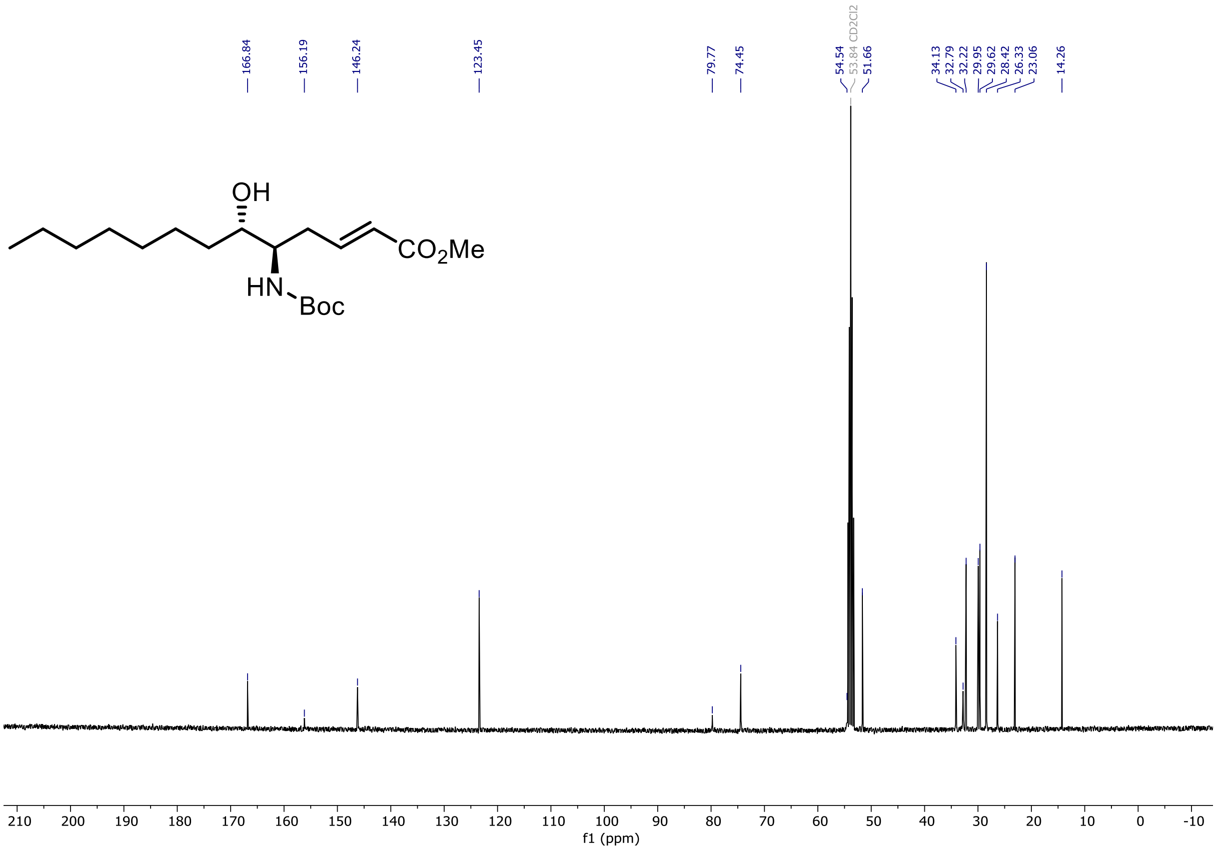

^{1}H NMR (400 MHz, CD_2Cl_2; top) and ^{13}C NMR (101 MHz, CD_2Cl_2; bottom) of compound **37**

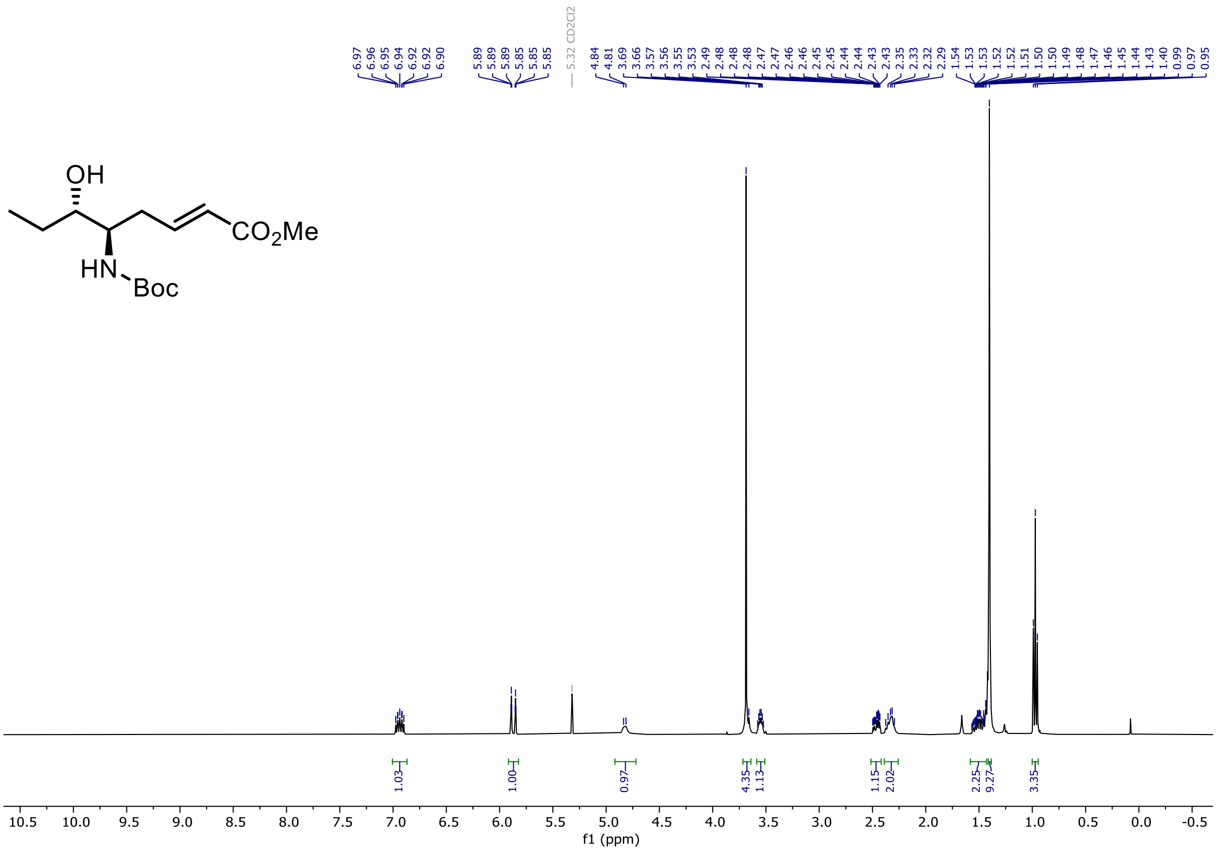

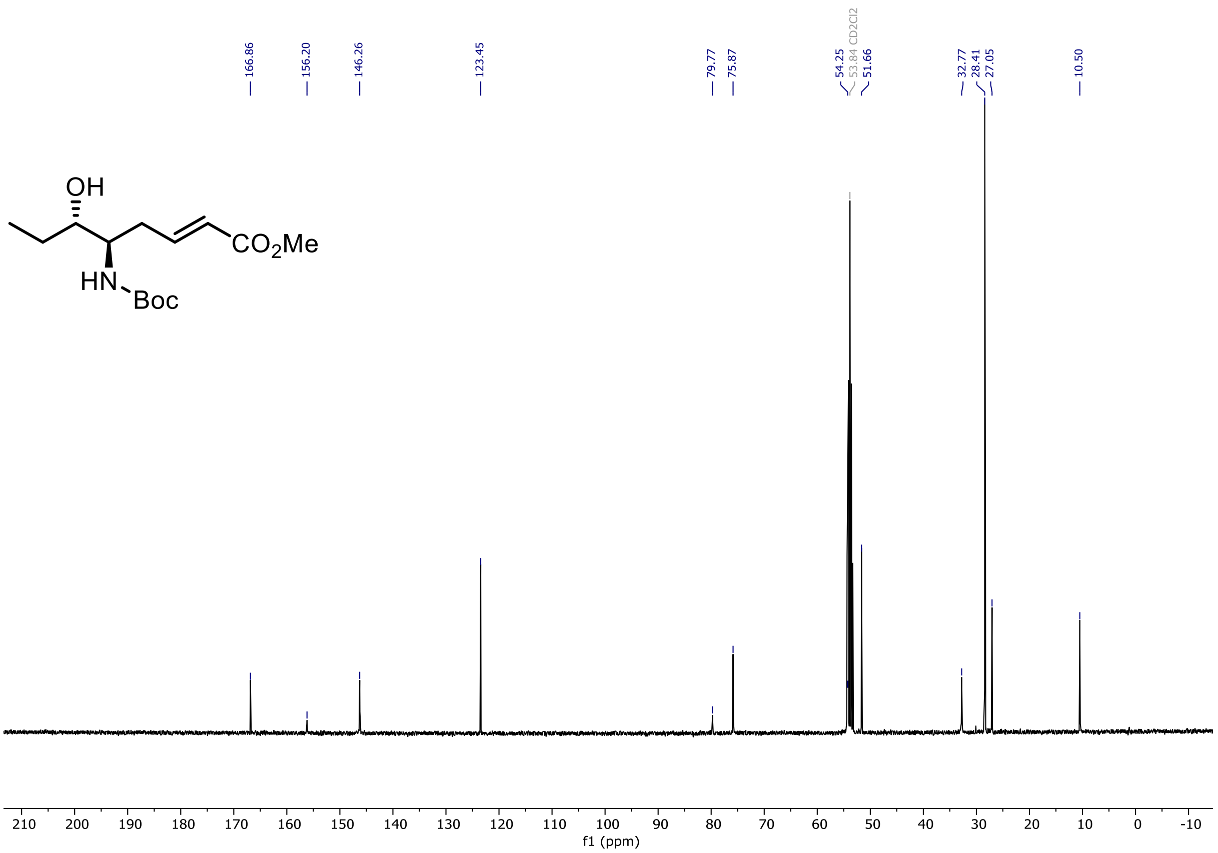

^{1}H NMR (400 MHz, CD_2Cl_2; top) and ^{13}C NMR (101 MHz, CD_2Cl_2; bottom) of compound **38**

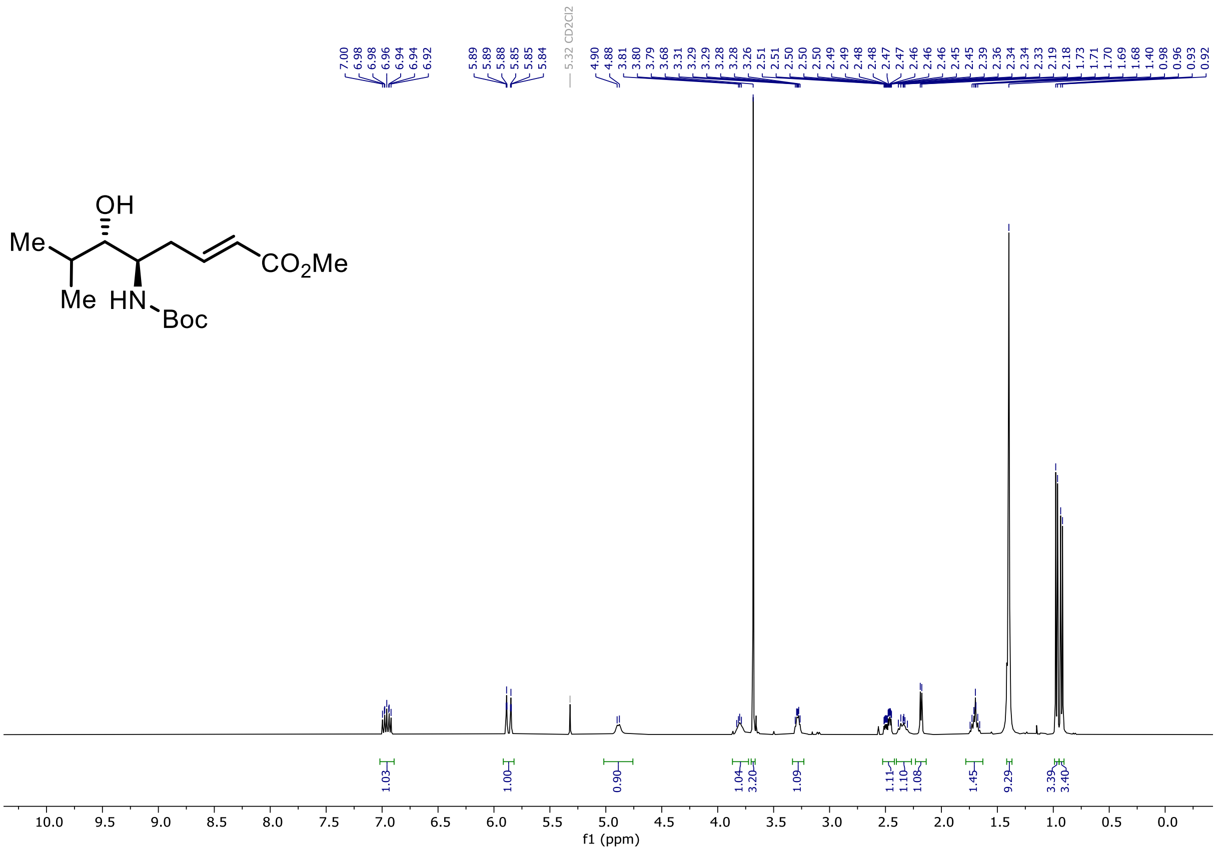

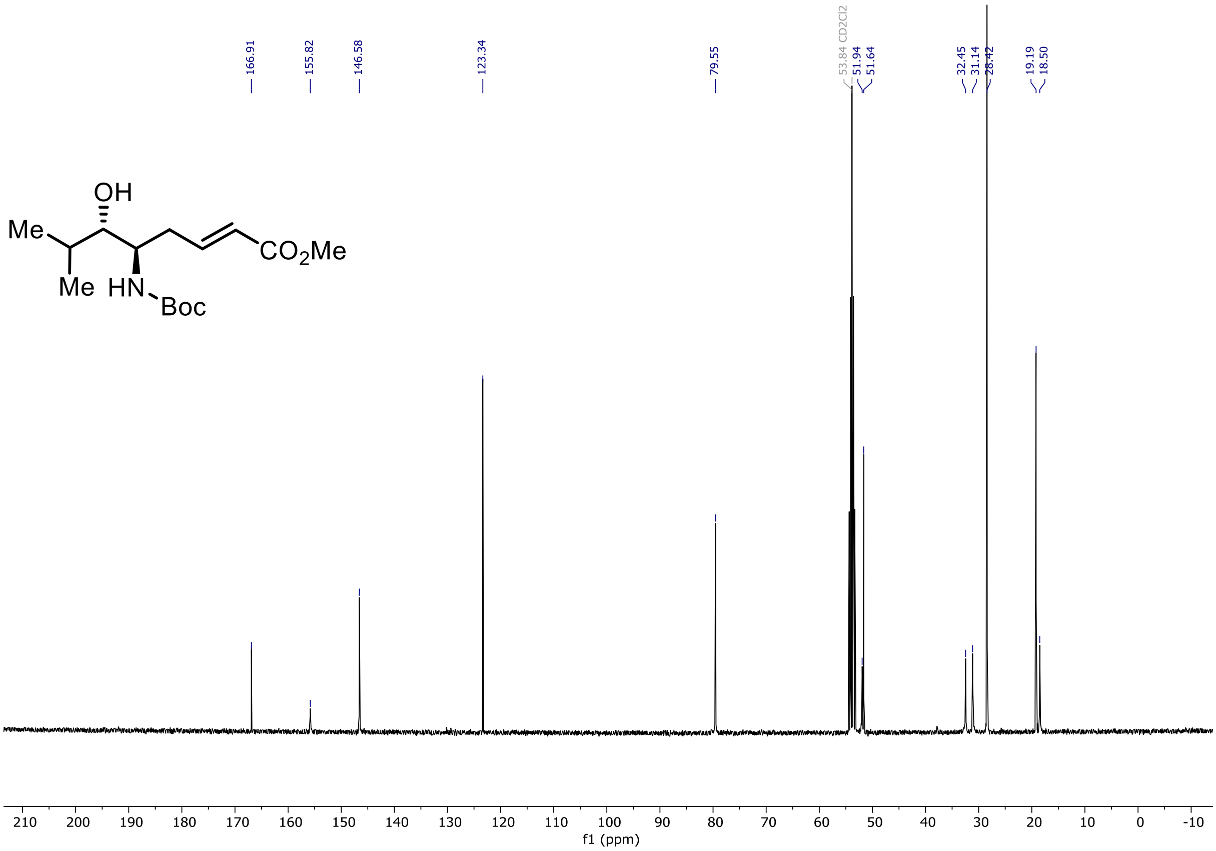

^{1}H NMR (400 MHz, CD_2Cl_2; top) and ^{13}C NMR (101 MHz, CD_2Cl_2; bottom) of compound **39**

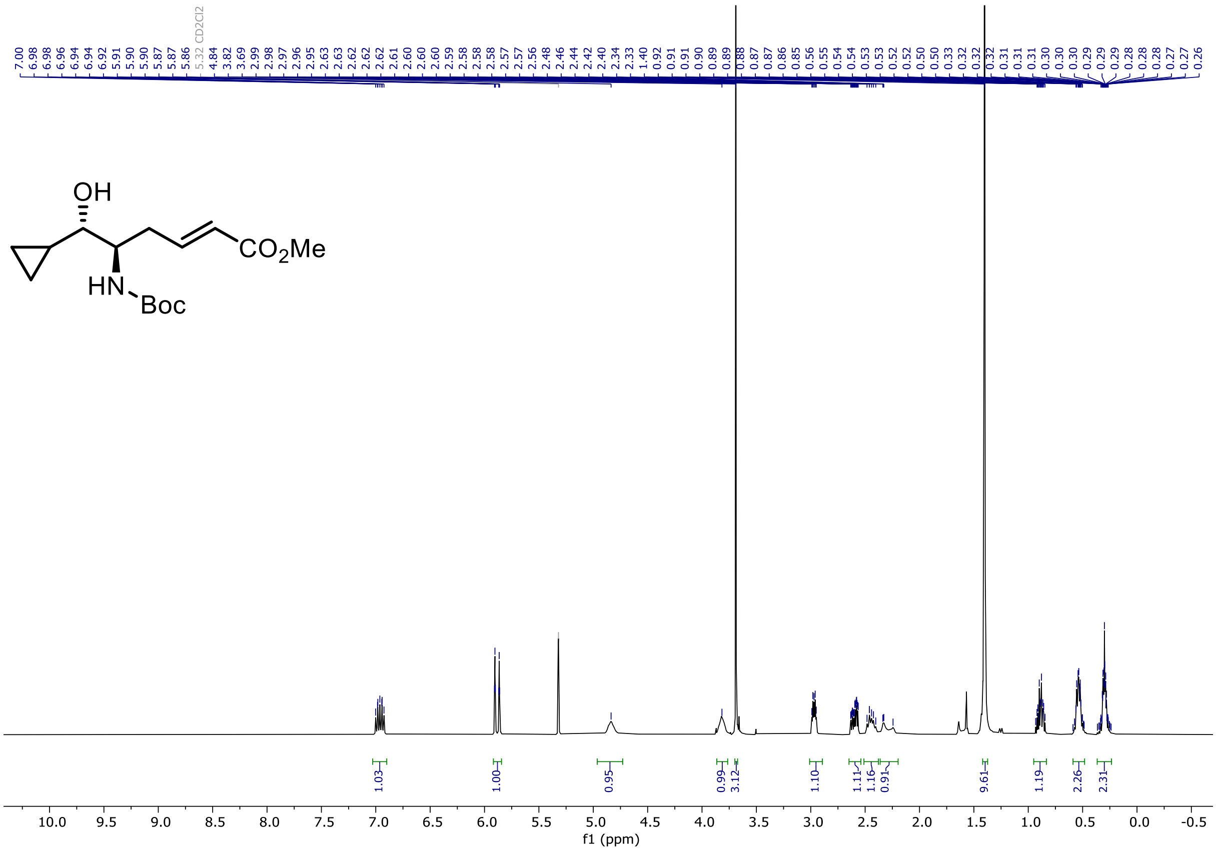

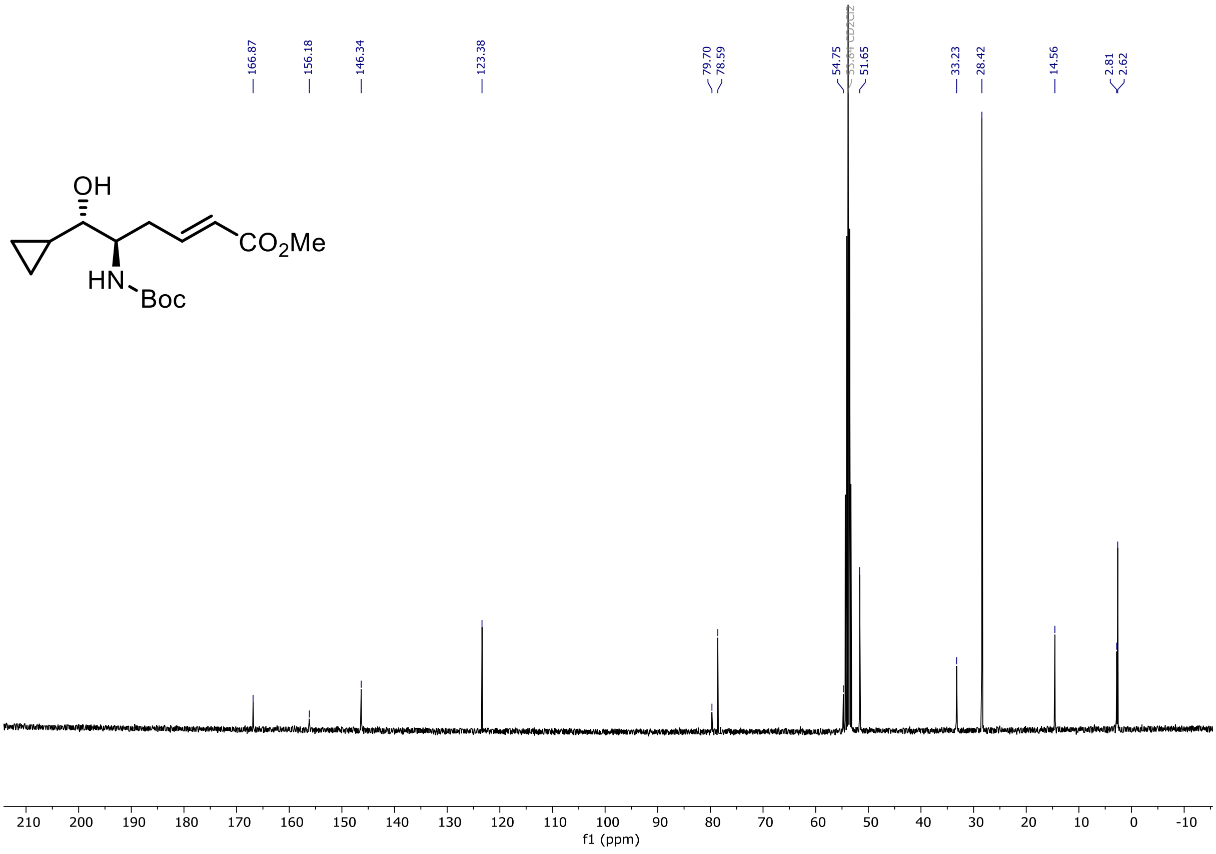

^{1}H NMR (400 MHz, CD_2Cl_2; top) and ^{13}C NMR (101 MHz, CD_2Cl_2; bottom) of compound **40**

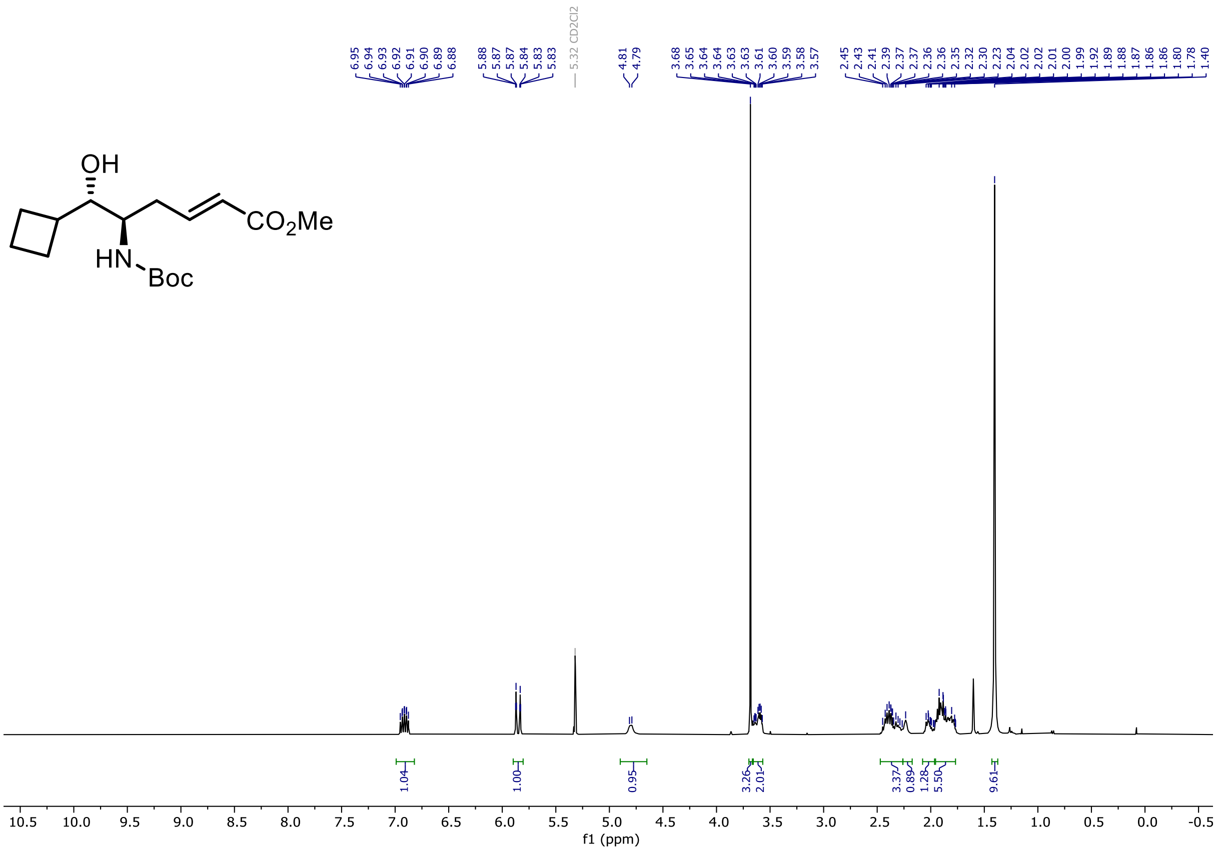

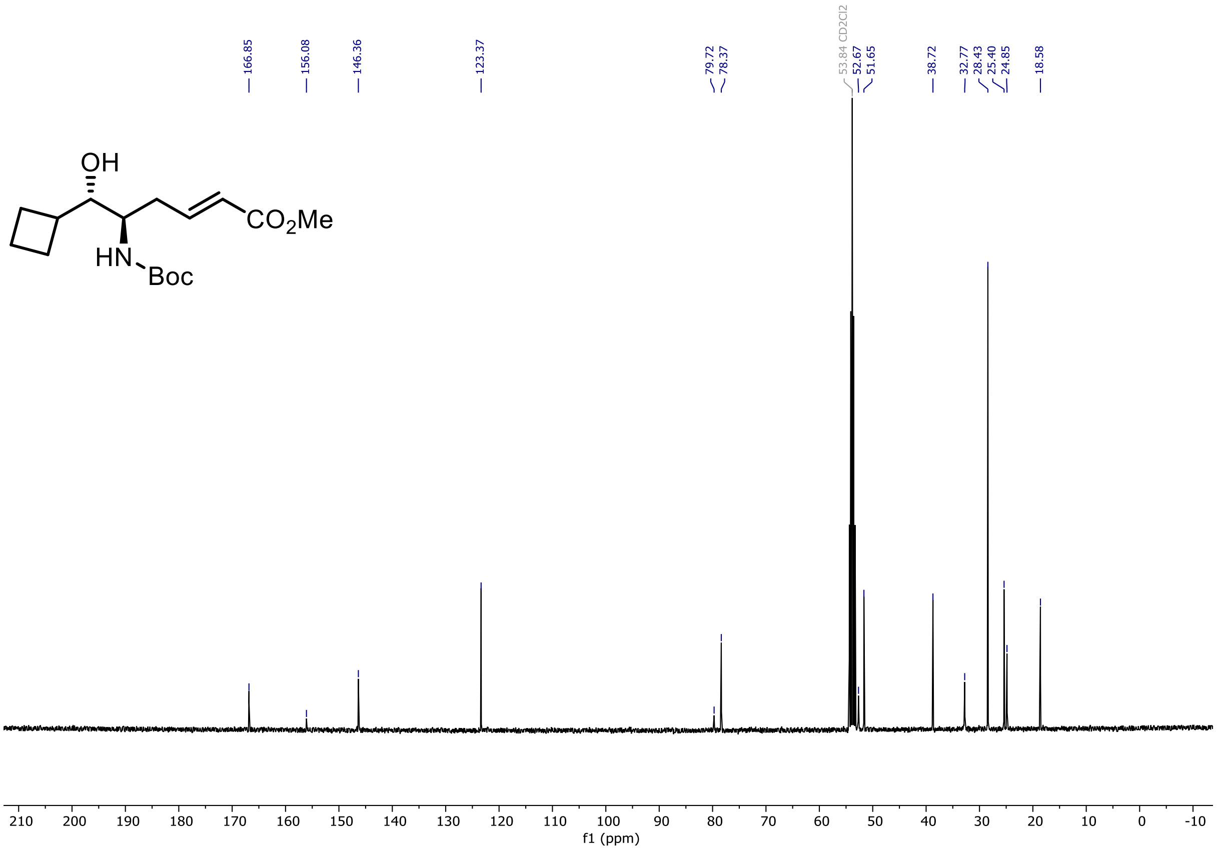

^{1}H NMR (400 MHz, CD_2Cl_2; top) and ^{13}C NMR (101 MHz, CD_2Cl_2; bottom) of compound **41**

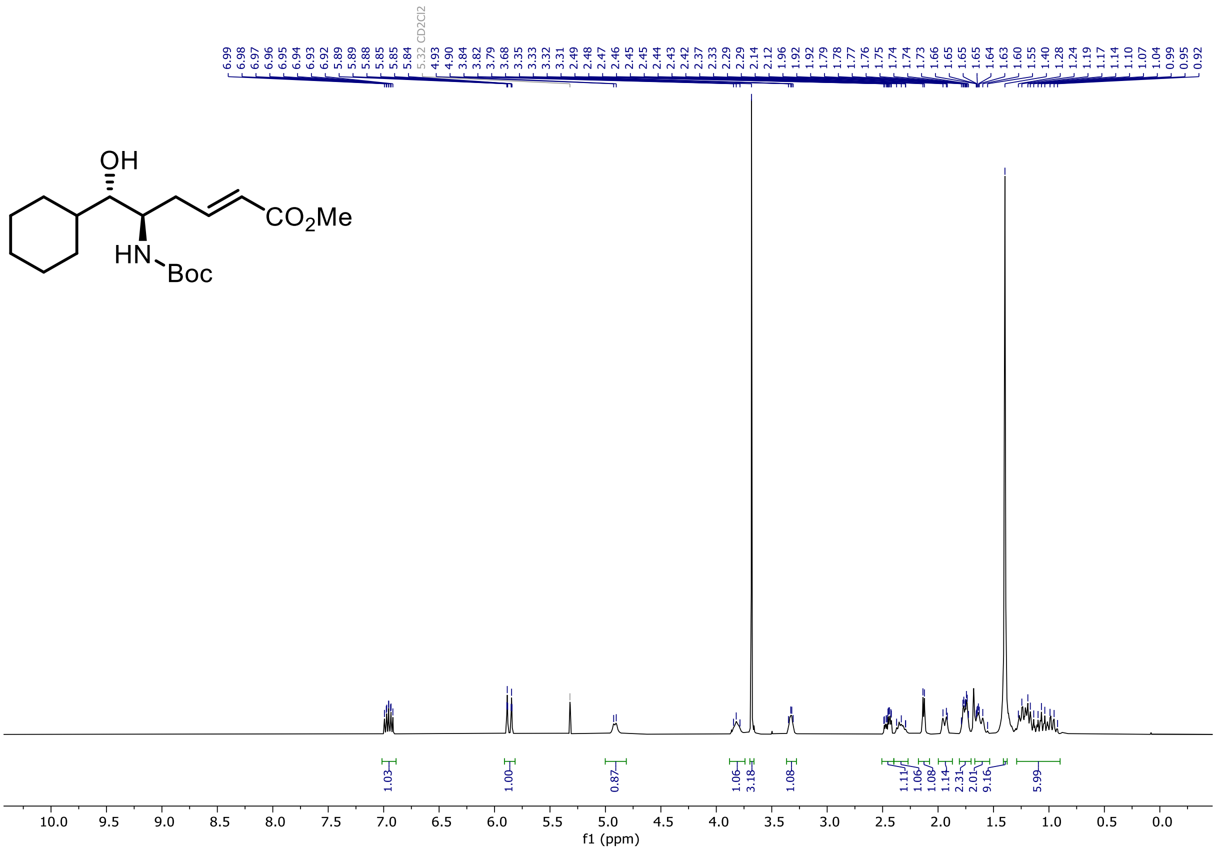

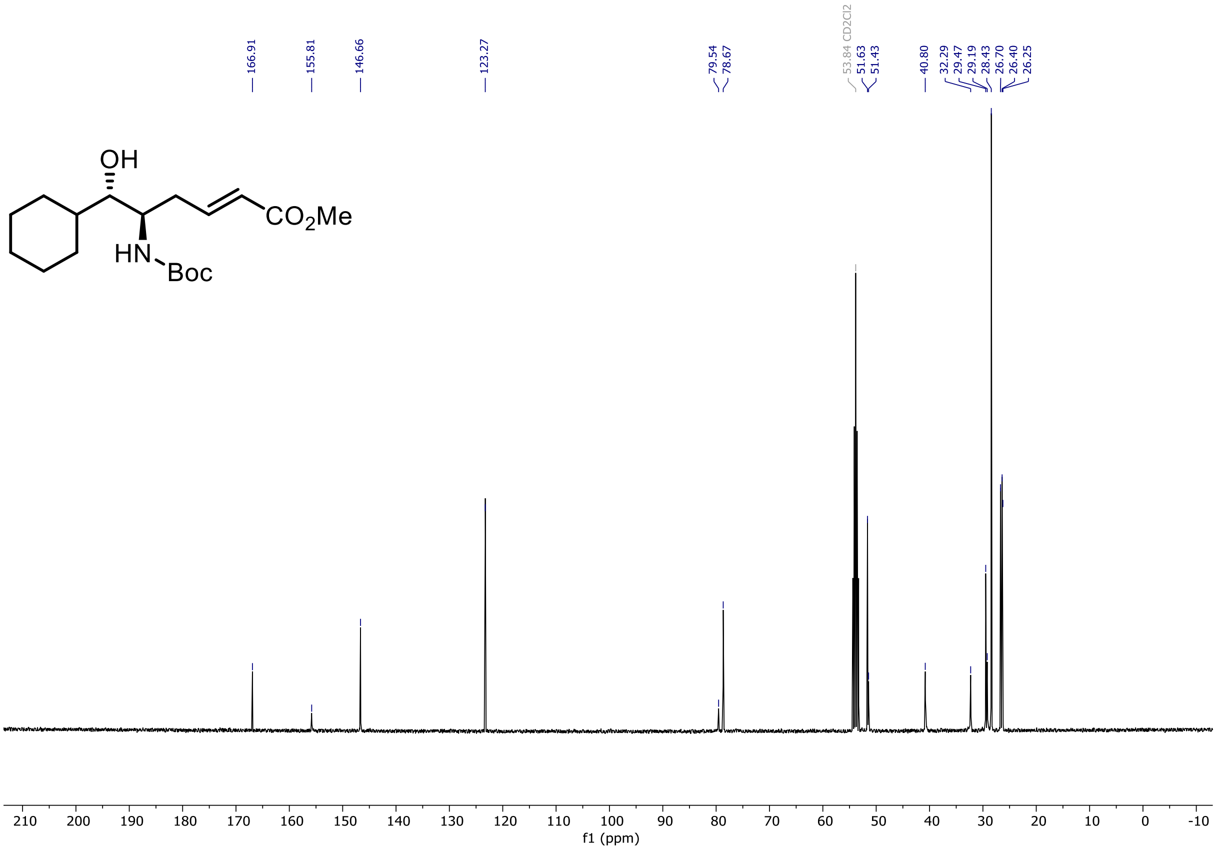

^{1}H NMR (400 MHz, CD_2Cl_2; top) and ^{13}C NMR (101 MHz, CD_2Cl_2; bottom) of compound **42**

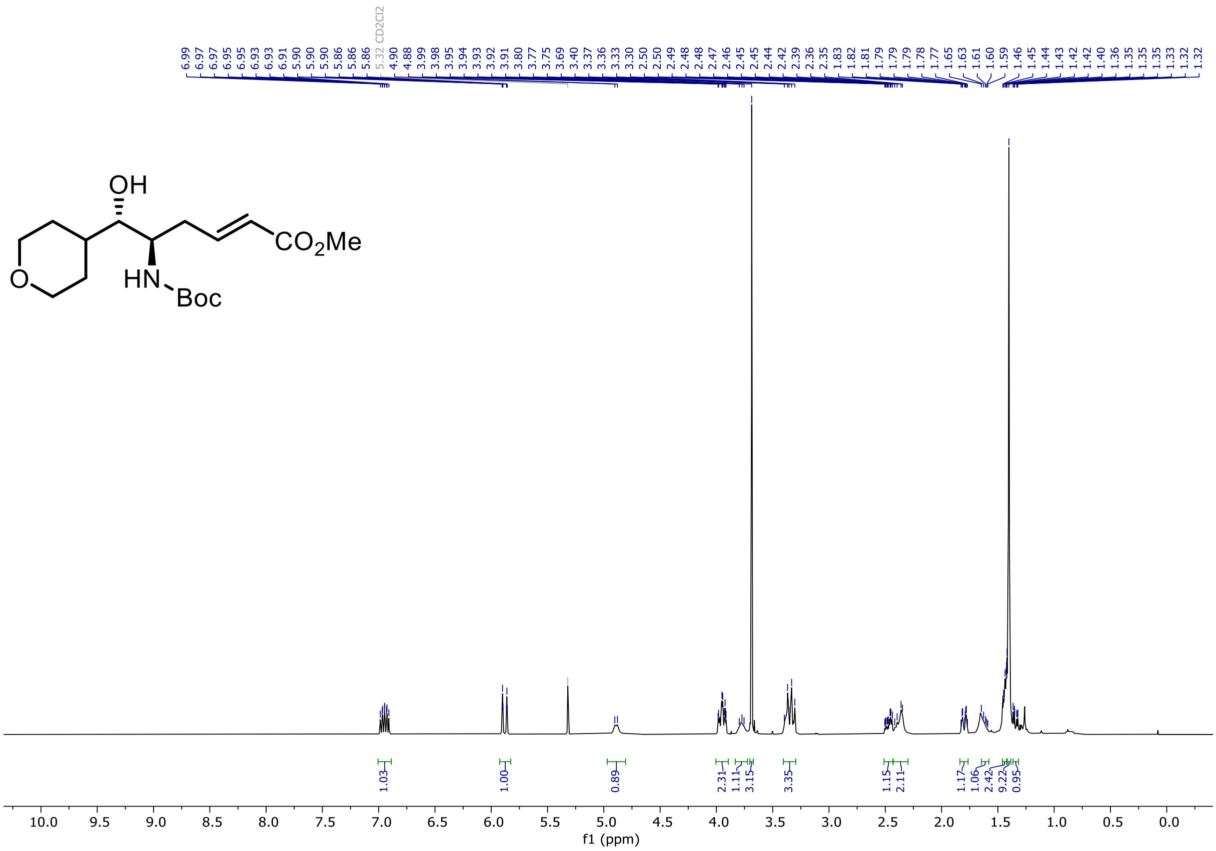

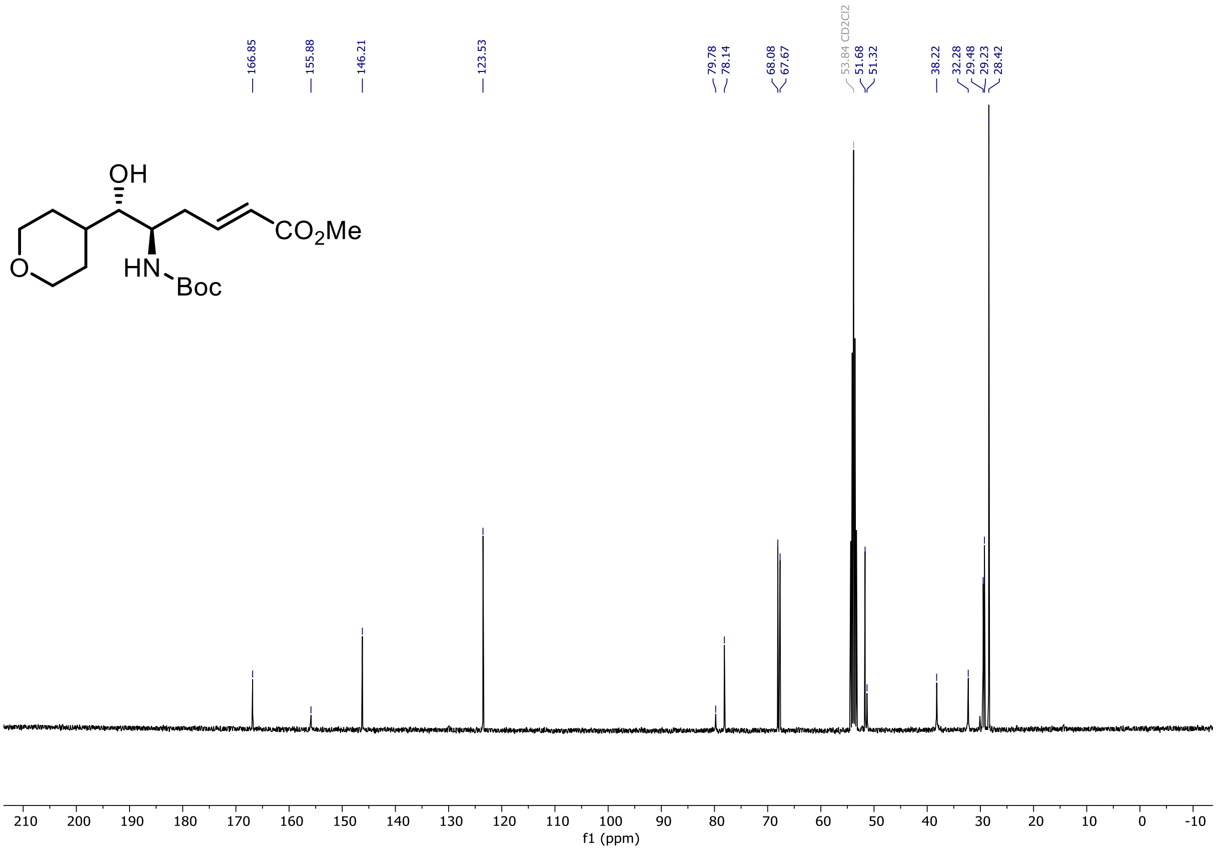

^{1}H NMR (400 MHz, CD_2Cl_2; top) and ^{13}C NMR (101 MHz, CD_2Cl_2; bottom) of compound **43a**

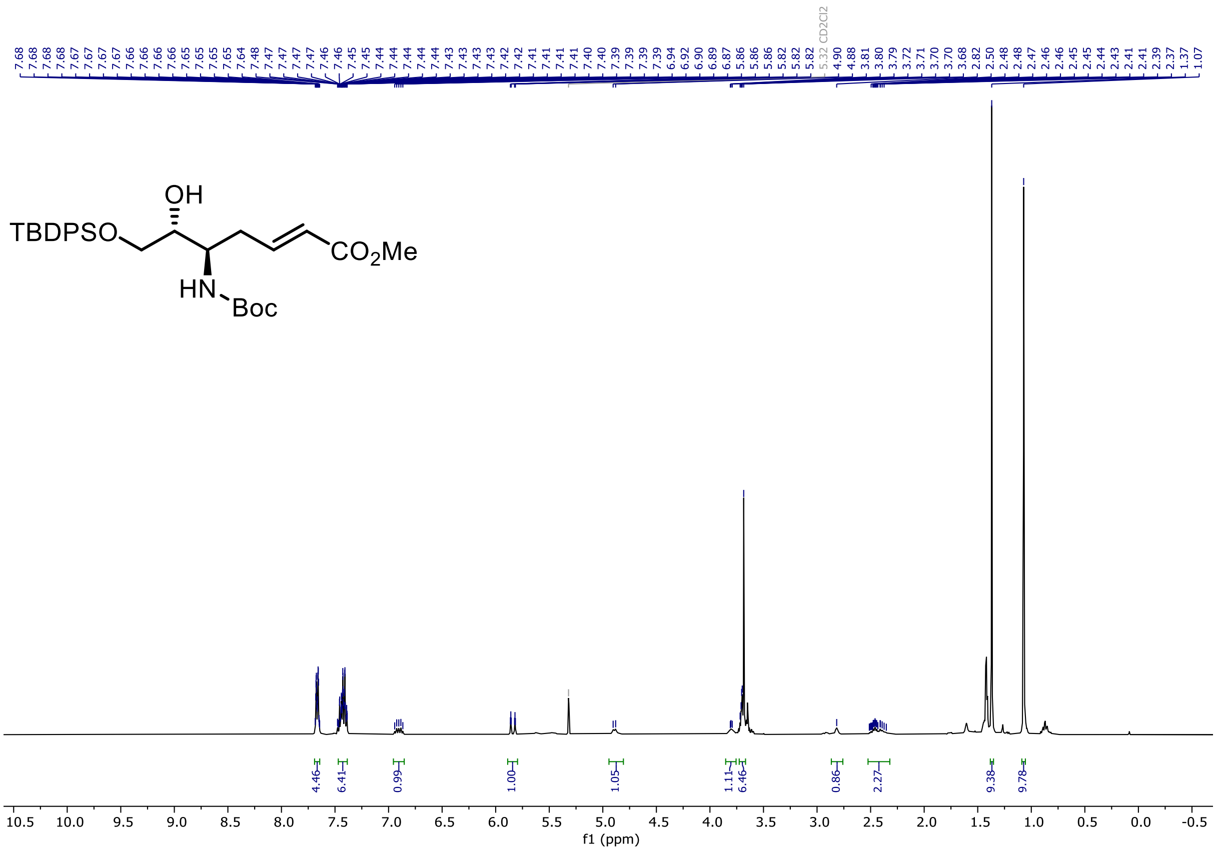

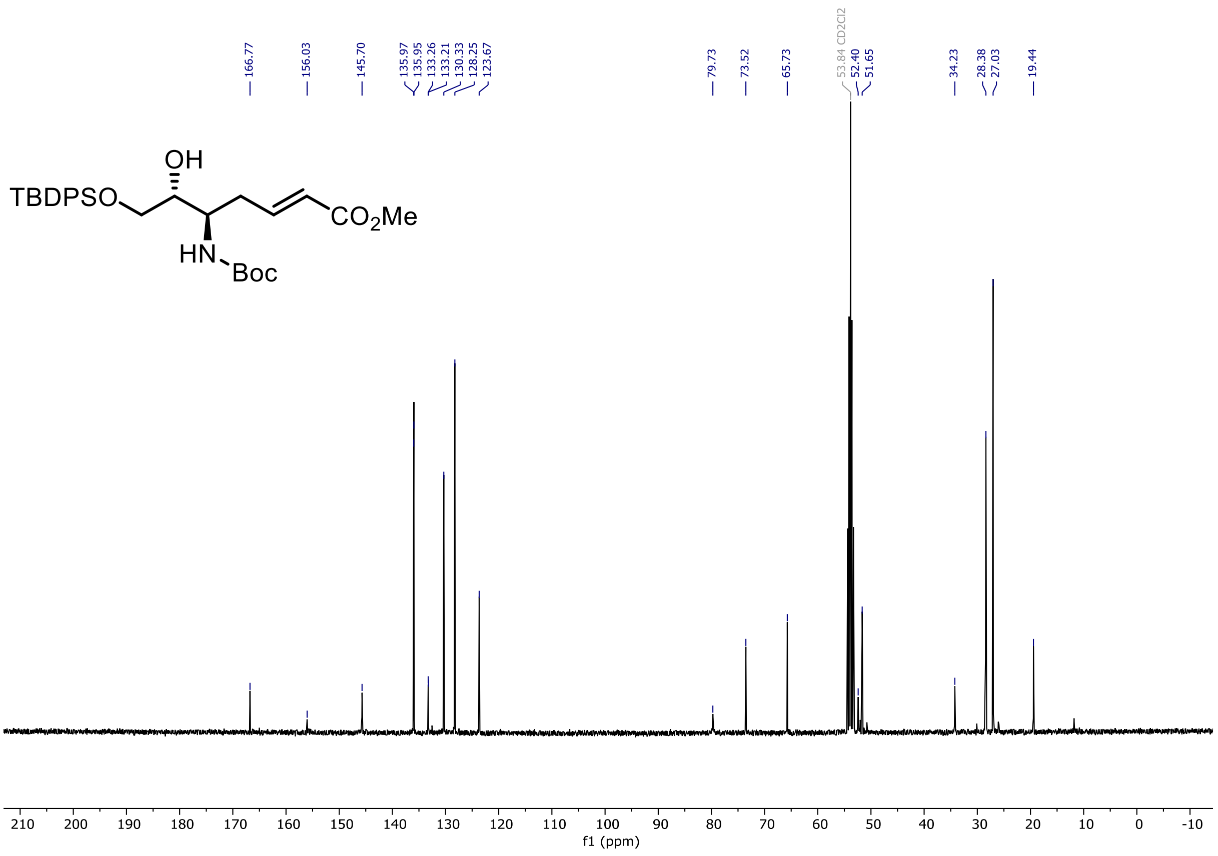

^{1}H NMR (400 MHz, CD_2Cl_2; top) and ^{13}C NMR (101 MHz, CD_2Cl_2; bottom) of compound **43b**

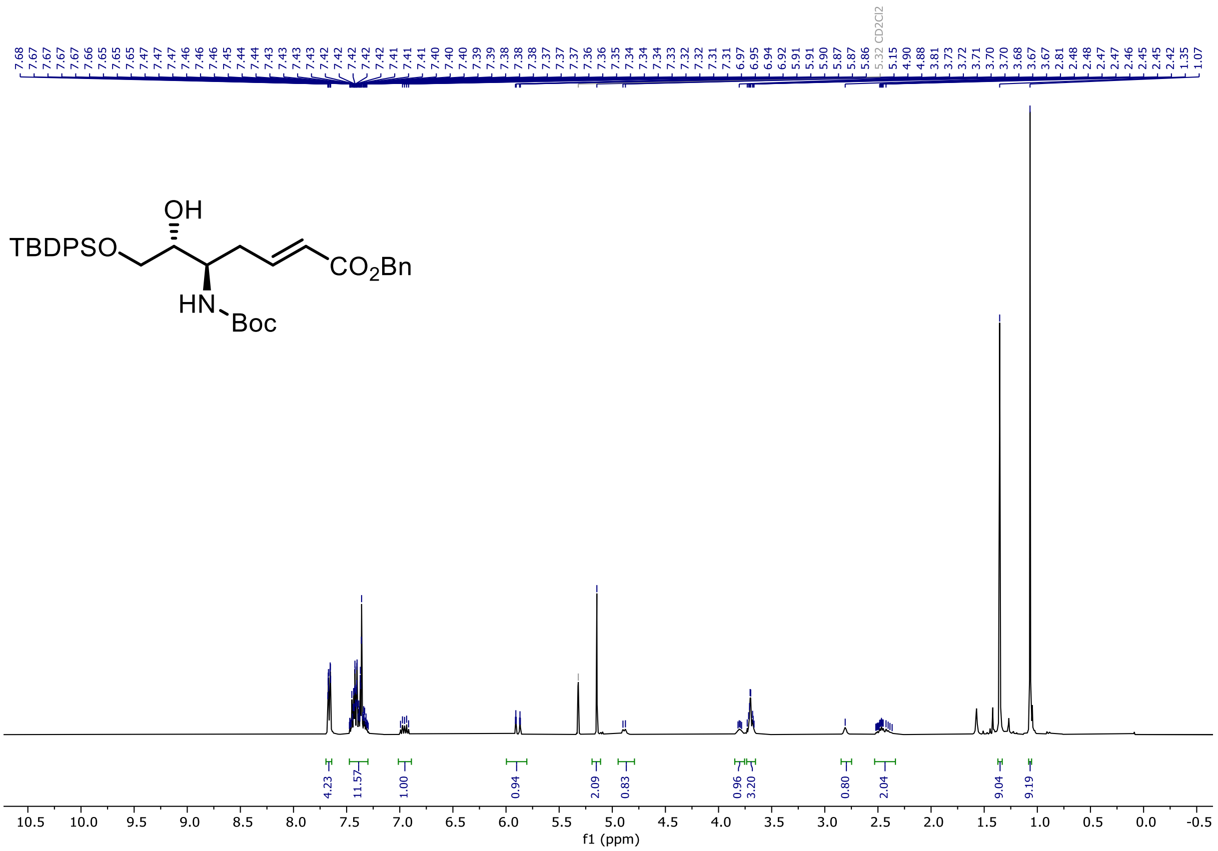

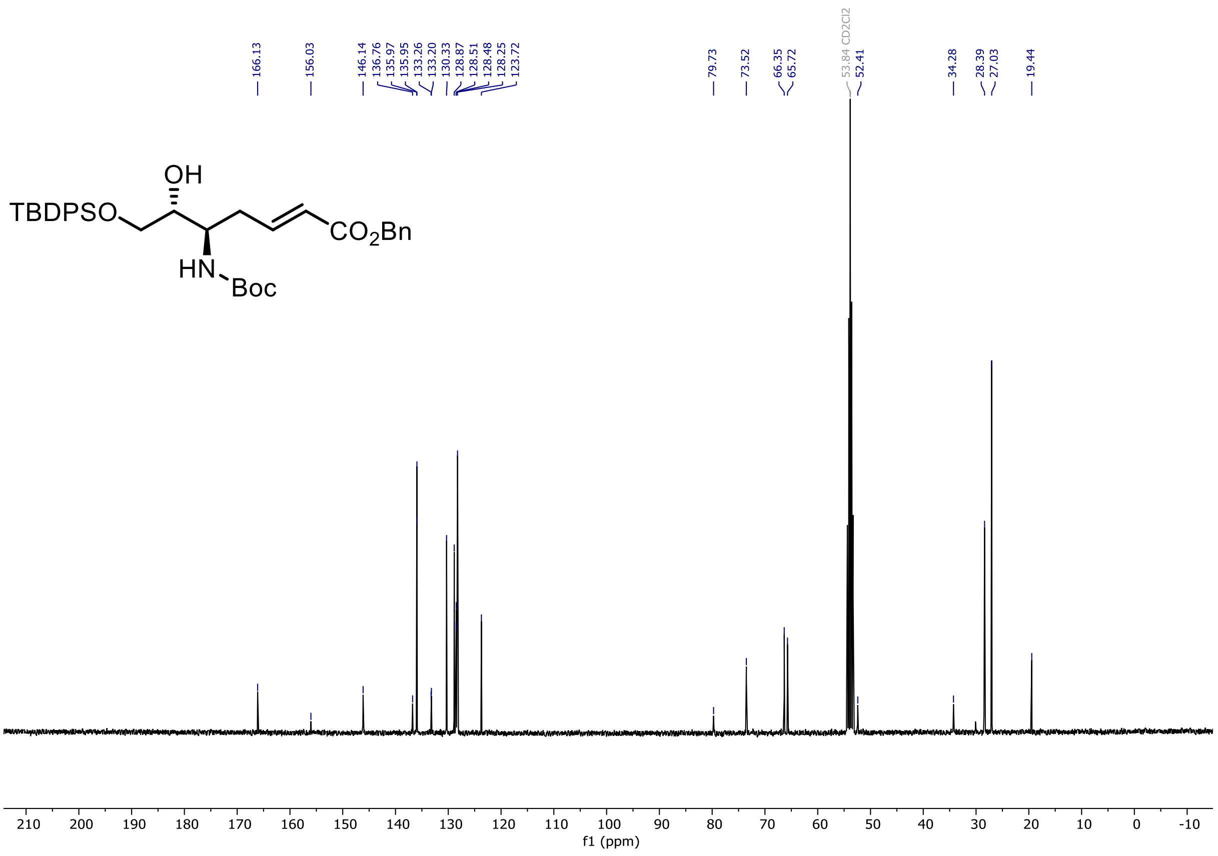

^{1}H NMR (400 MHz, CD_2Cl_2; top) and ^{13}C NMR (101 MHz, CD_2Cl_2; bottom) of compound **44**

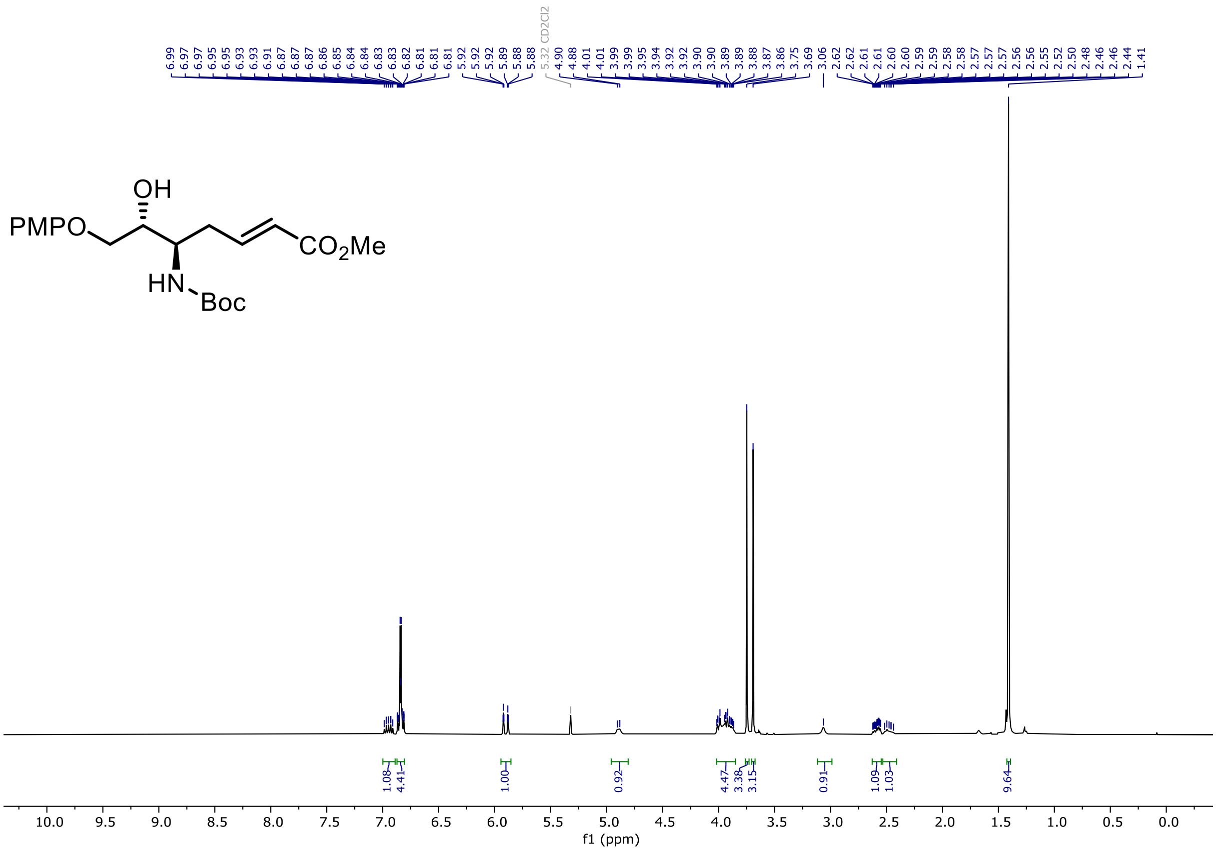

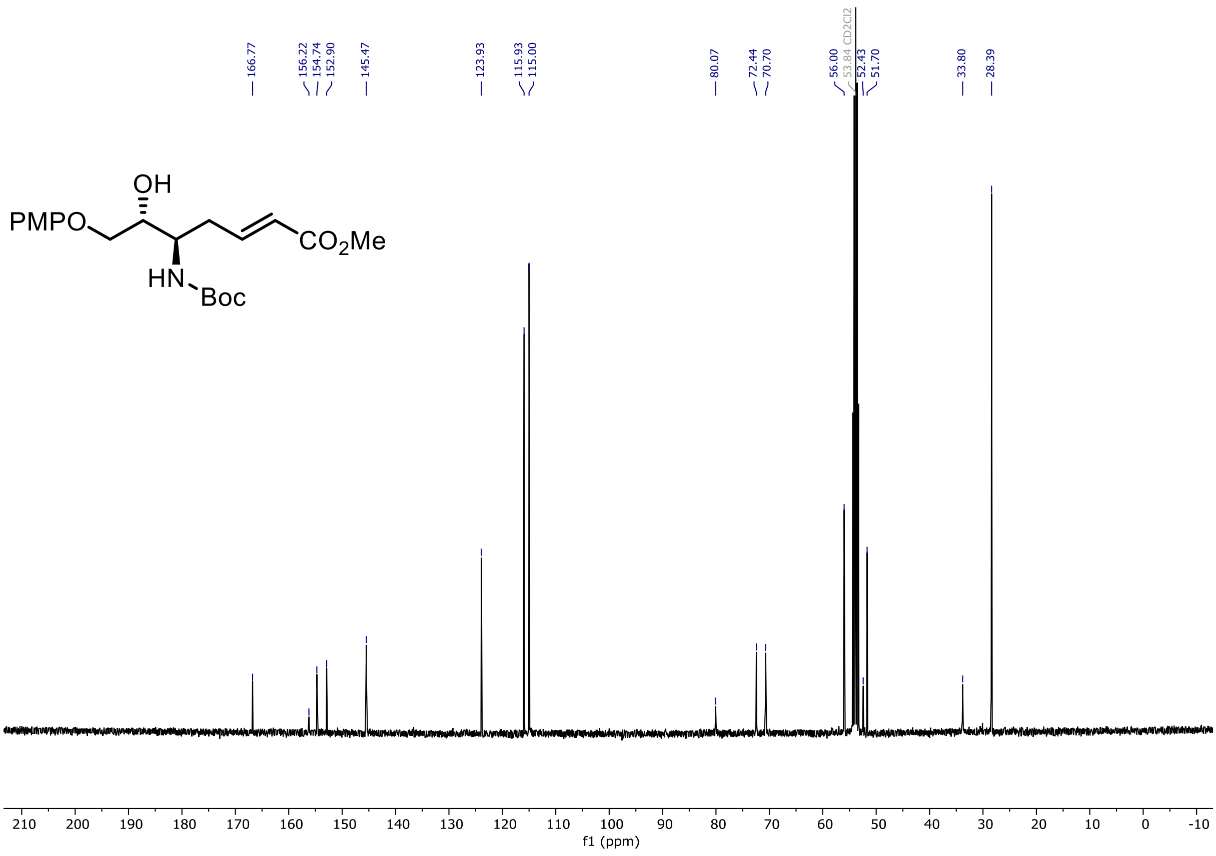

^{1}H NMR (400 MHz, CD_2Cl_2; top) and ^{13}C NMR (101 MHz, CD_2Cl_2; bottom) of compound **45** (dr ≈ 20:1)

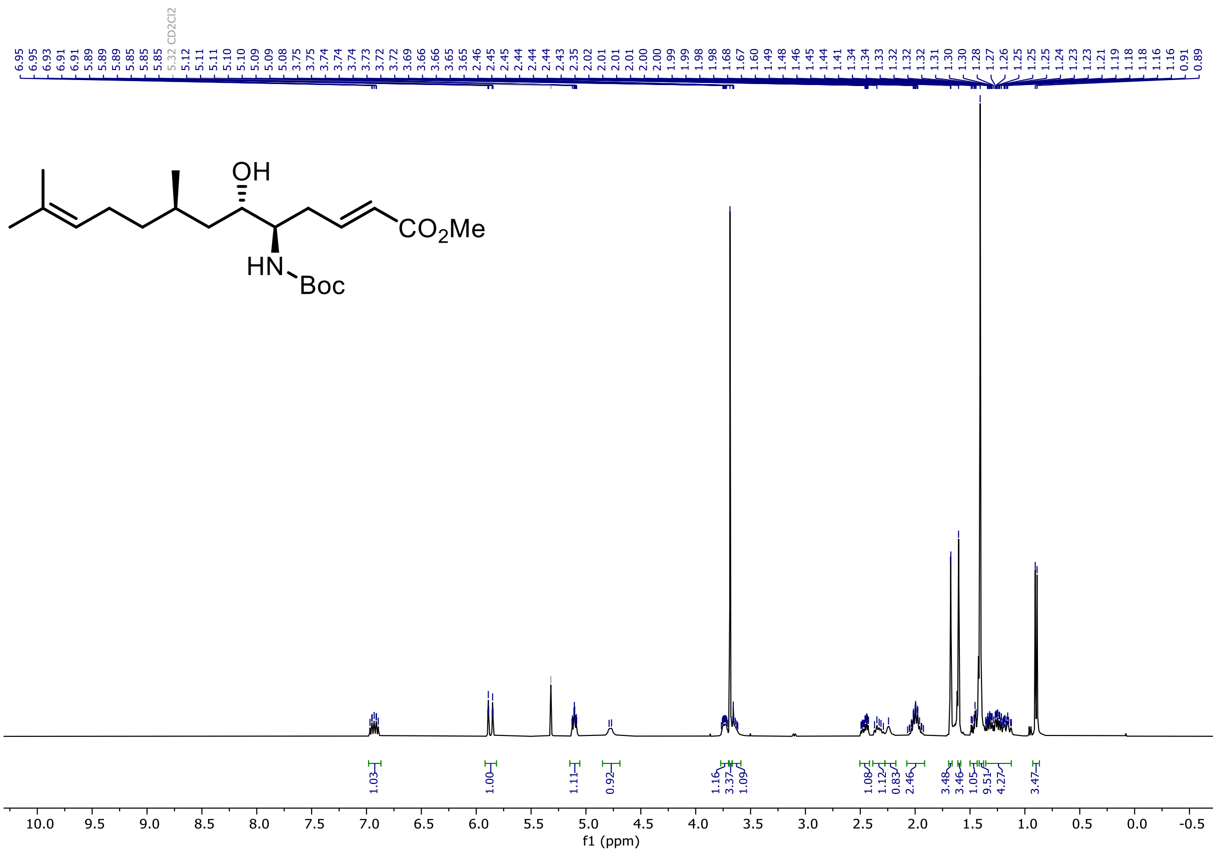

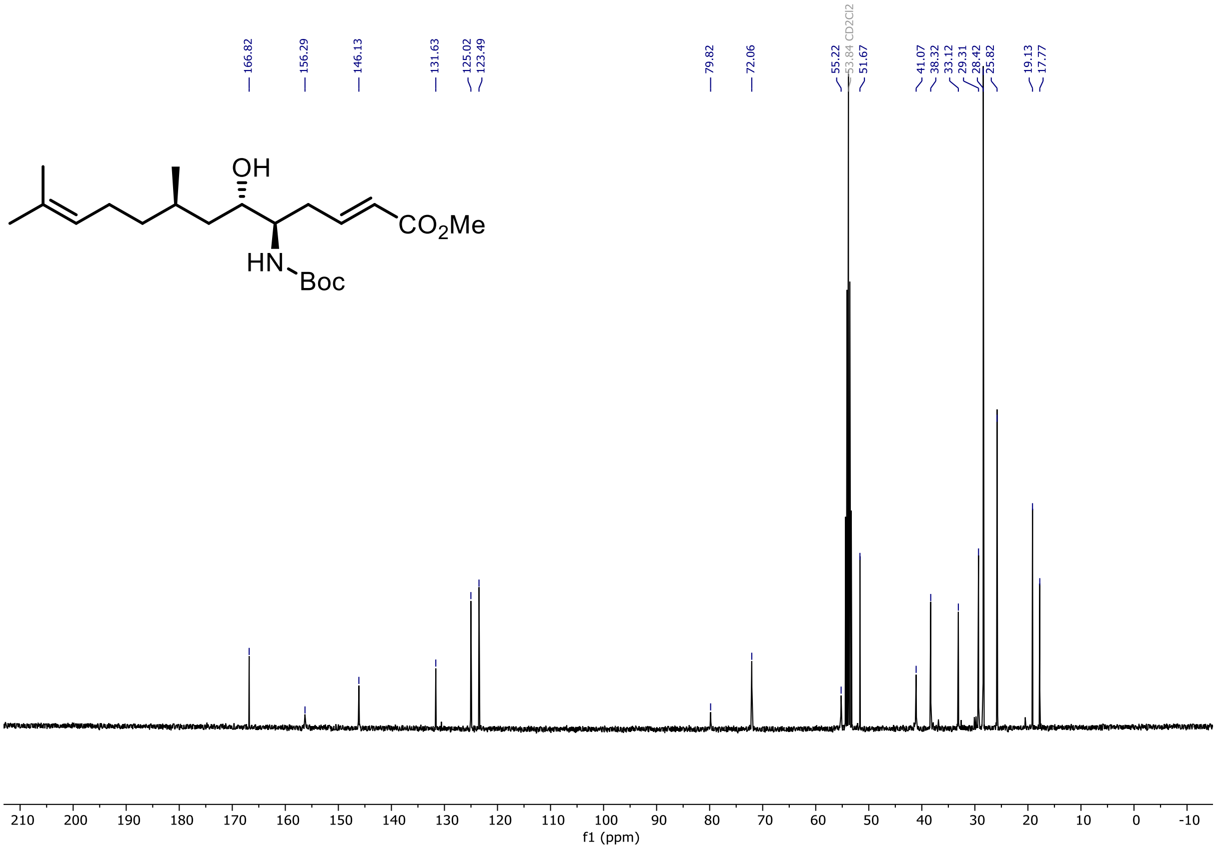

^{1}H NMR (400 MHz, CD_2Cl_2; top) and ^{13}C NMR (101 MHz, CD_2Cl_2; bottom) of compound **46**

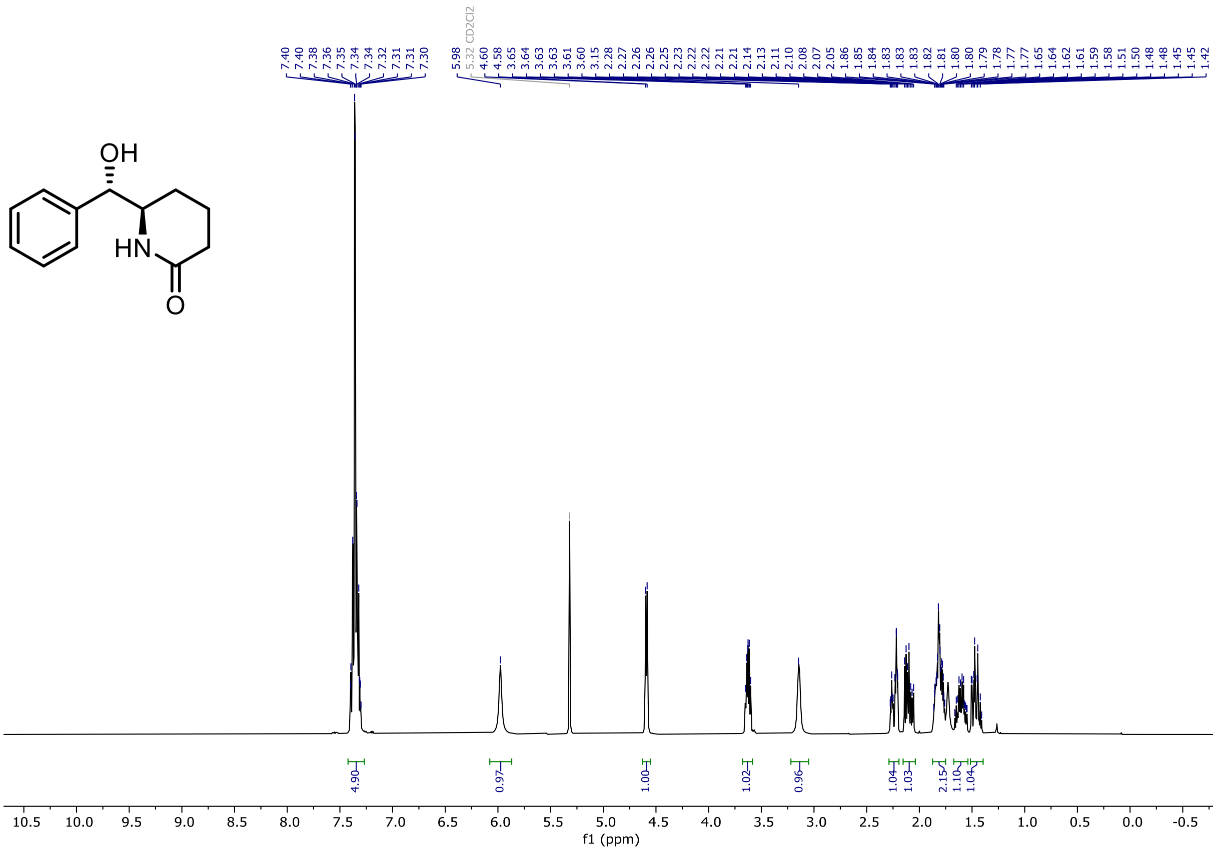

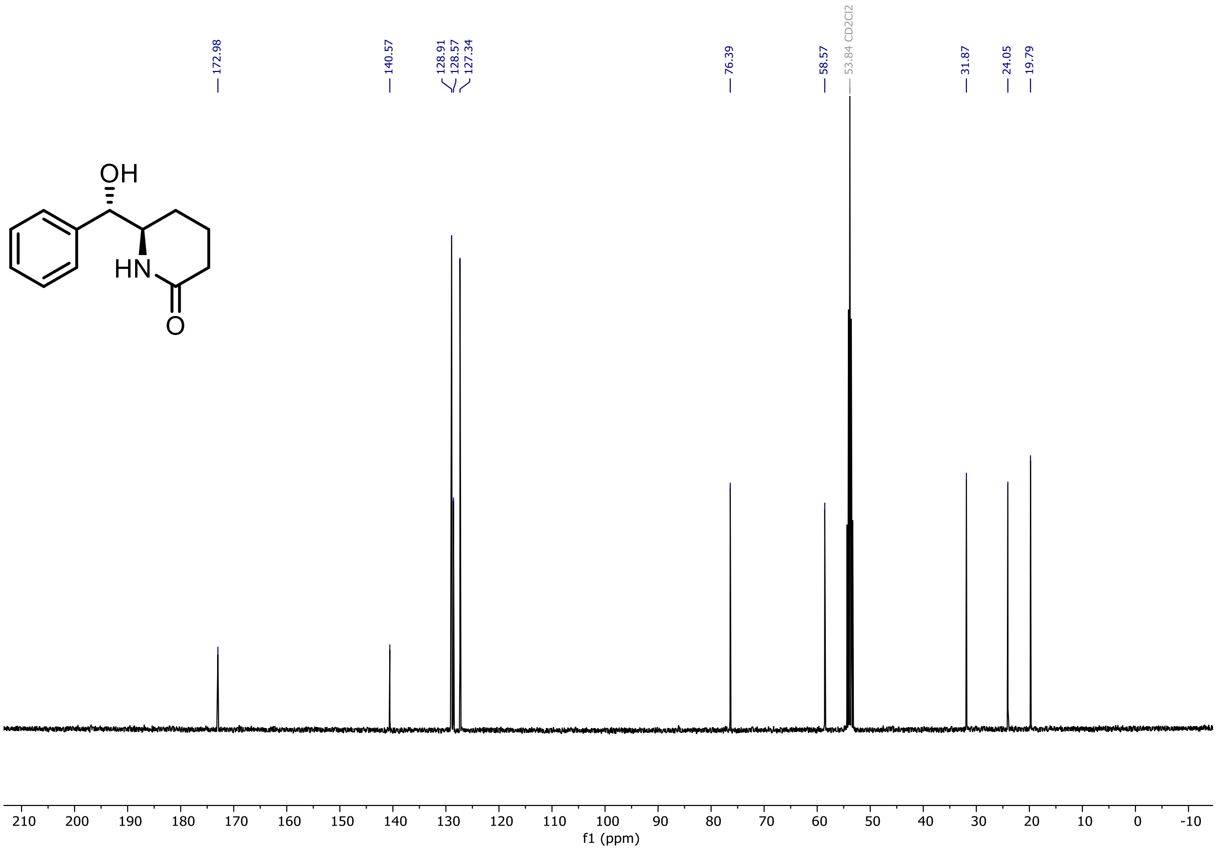

^{1}H NMR (400 MHz, CD_2Cl_2; top) and ^{13}C NMR (101 MHz, CD_2Cl_2; bottom) of compound **47**

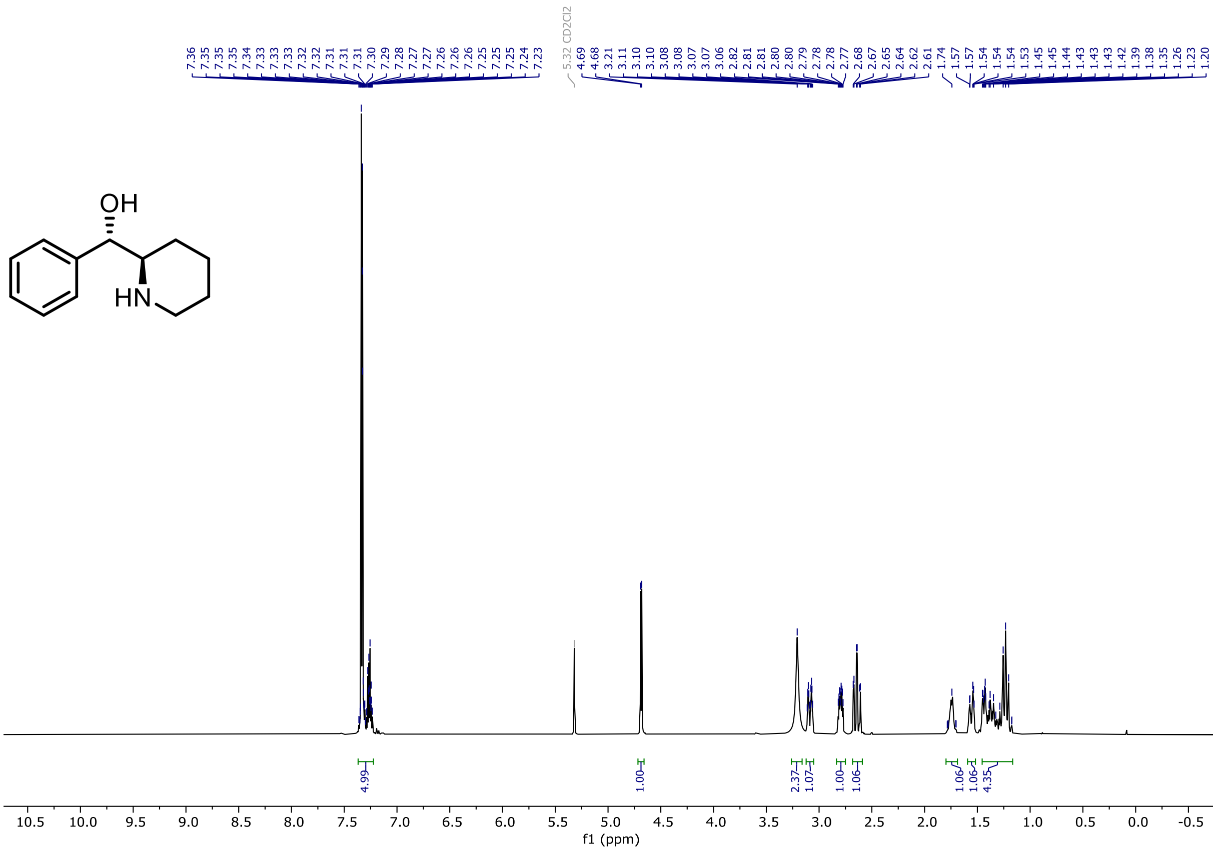

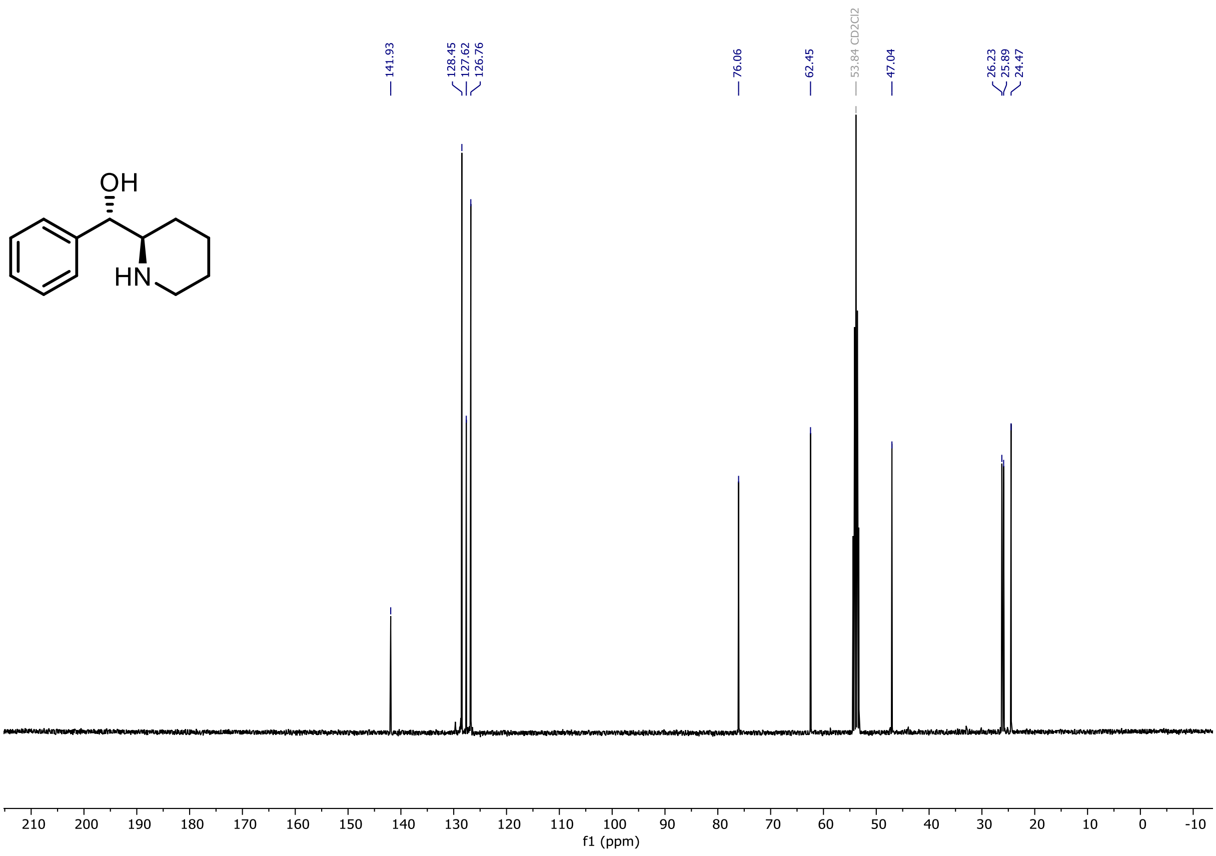

^{1}H NMR (400 MHz, $CDCl_3$; top) and ^{13}C NMR (101 MHz, $CDCl_3$; bottom) of compound **48**

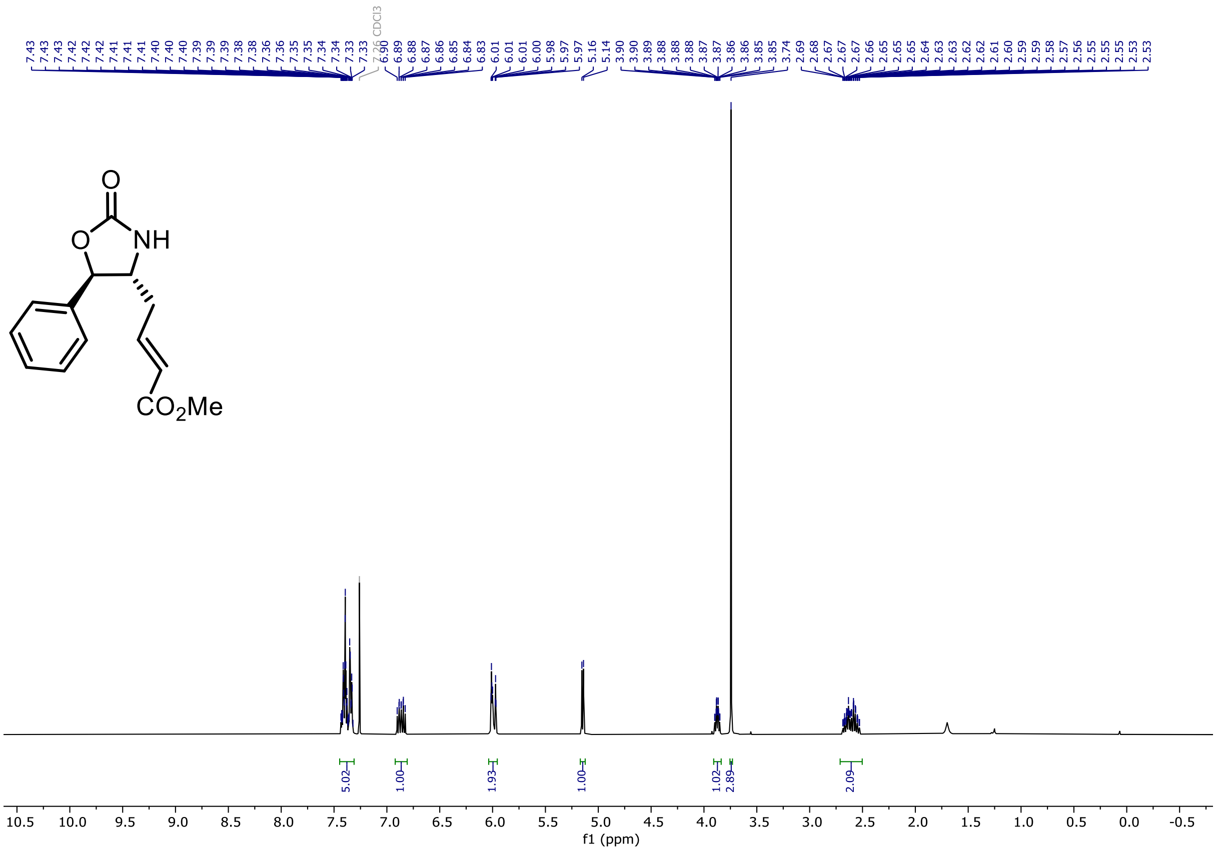

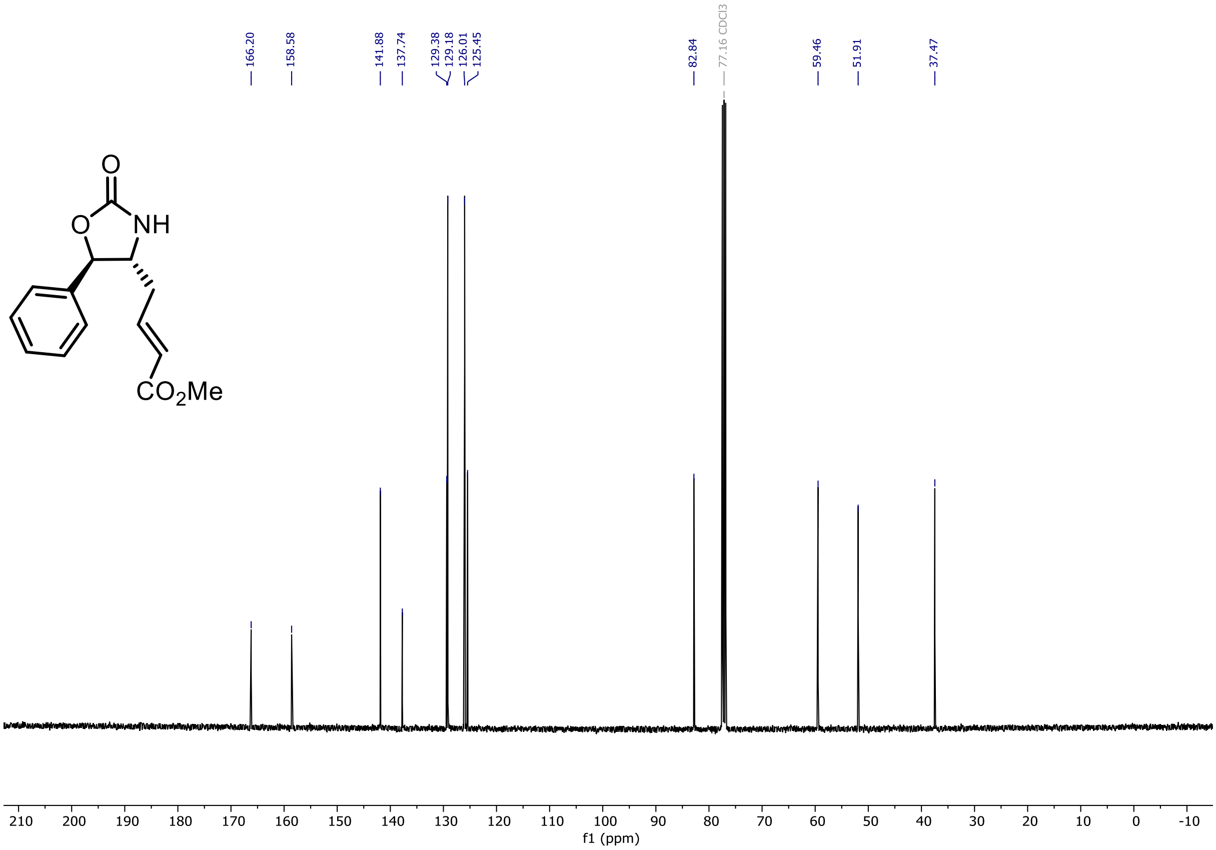

^{1}H NMR (600 MHz, $CDCl_3$, 263K; top) and ^{13}C NMR (151 MHz, $CDCl_3$, 263 K; bottom) of compound **49**

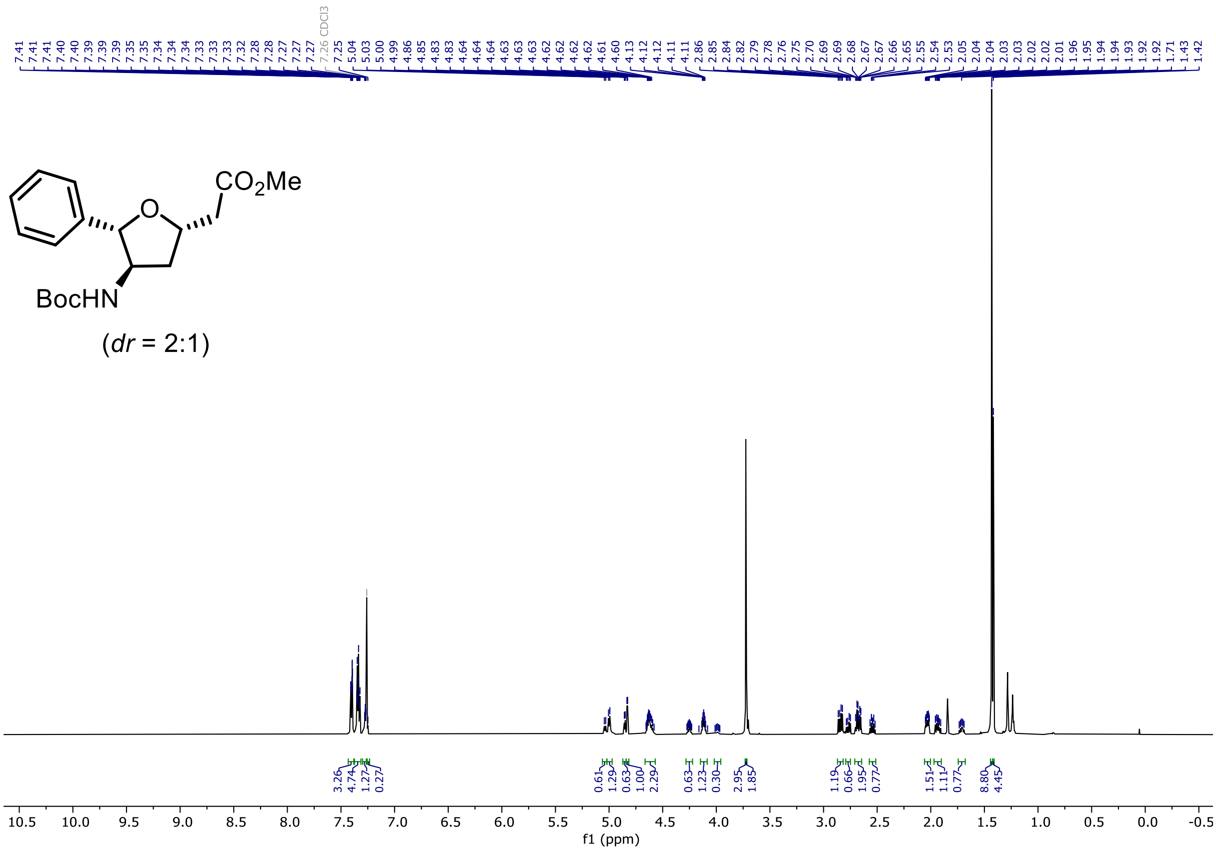

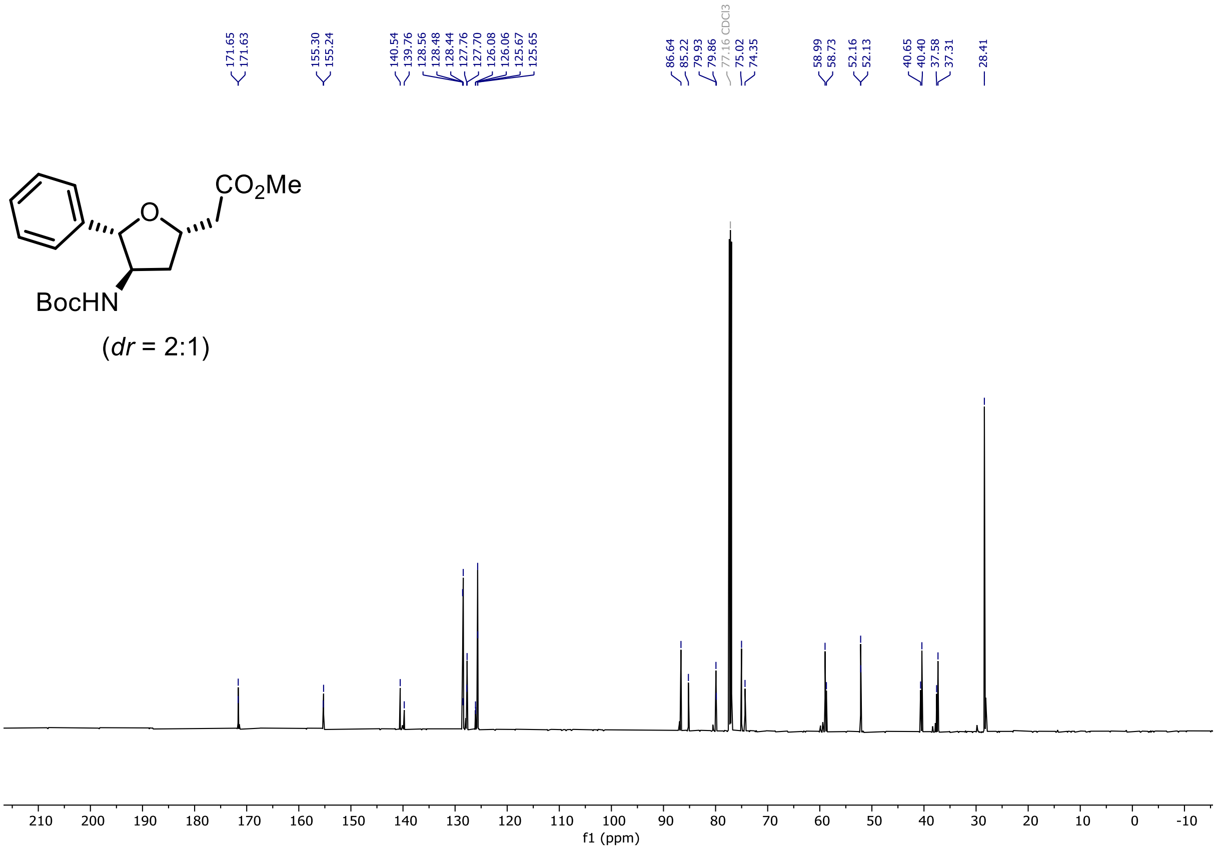

^{1}H NMR (400 MHz, CD_2Cl_2; top) and ^{13}C NMR (101 MHz, CD_2Cl_2; bottom) of compound **50**

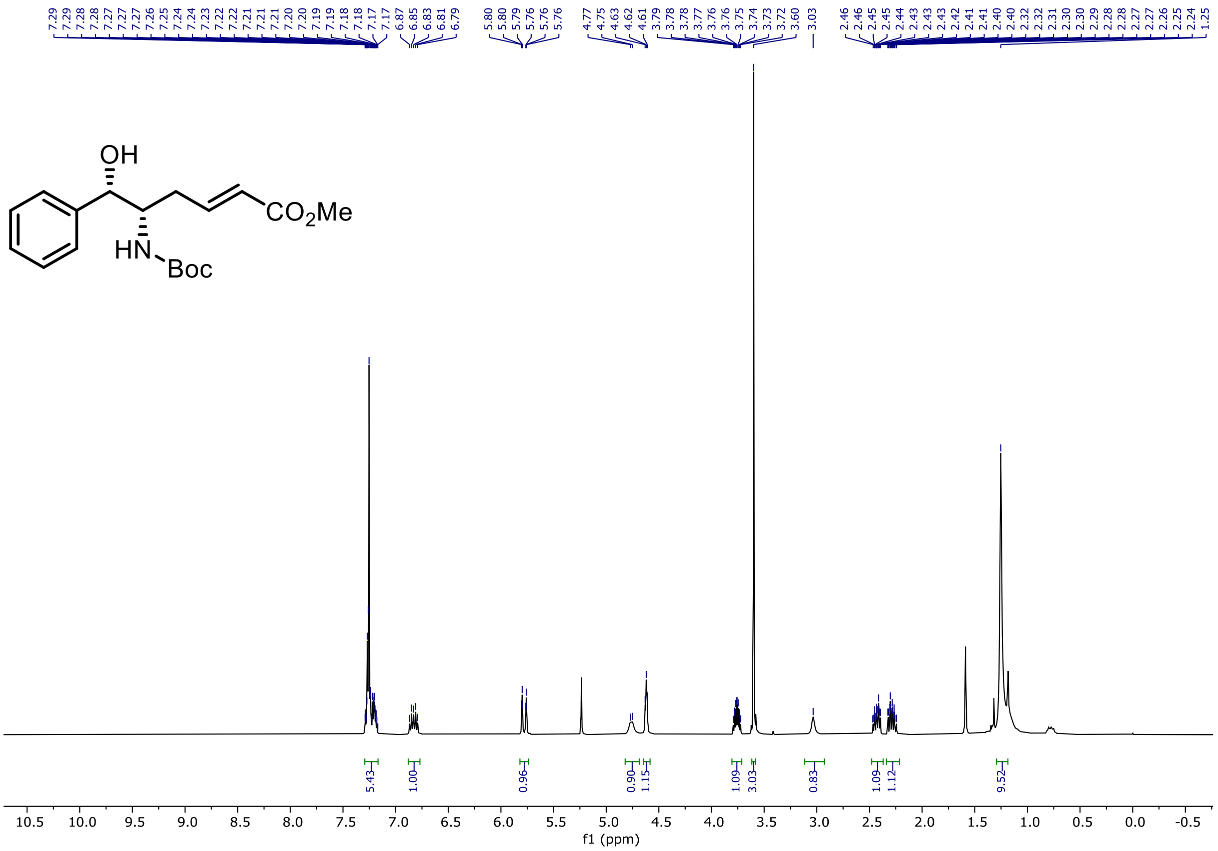

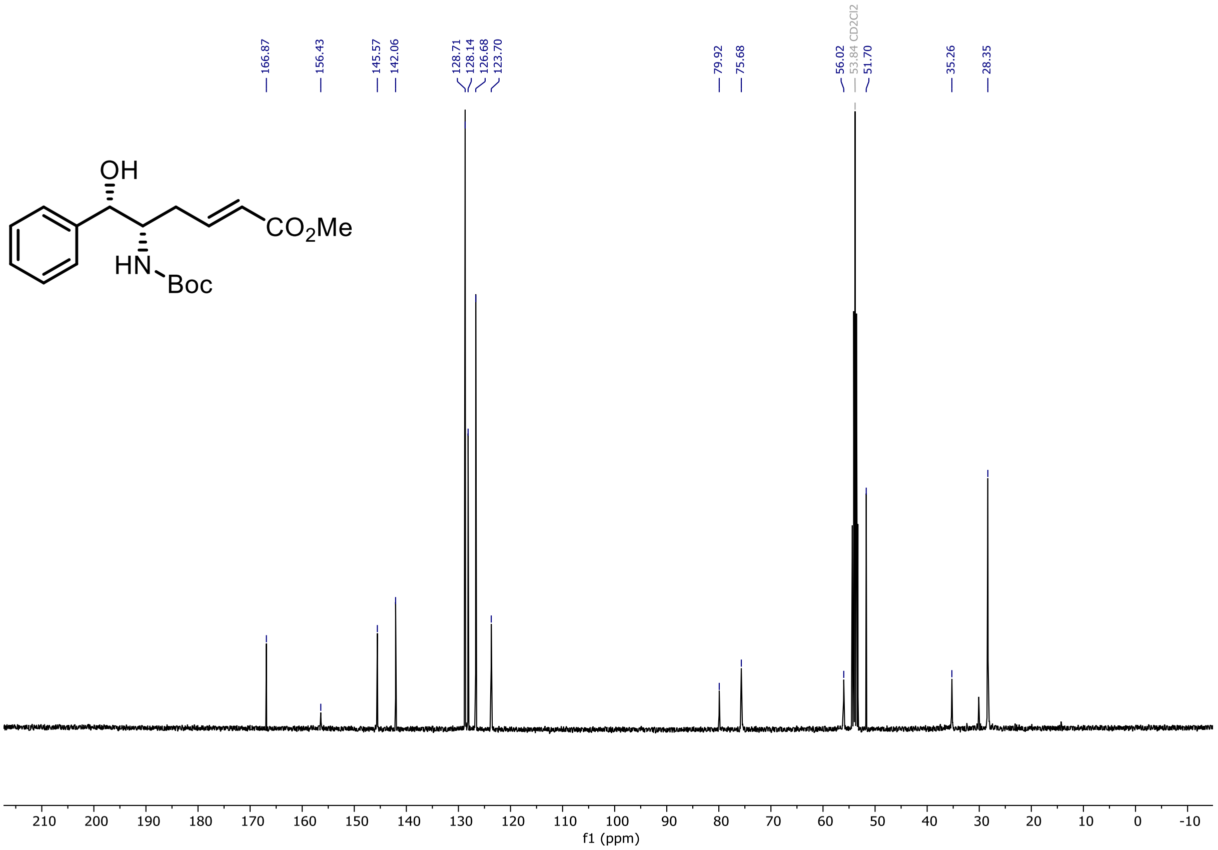

Supplement: Supplementary file 1 — ja4c12002_si_001.pdf [file ja4c12002_si_001.pdf]
